# Flipping the GPCR Switch: Structure-Based Development of Selective Cannabinoid Receptor 2 Inverse Agonists

**DOI:** 10.1021/acscentsci.3c01461

**Published:** 2024-03-11

**Authors:** Miroslav Kosar, Roman C. Sarott, David A. Sykes, Alexander E. G. Viray, Rosa Maria Vitale, Nataša Tomašević, Xiaoting Li, Rudolf L. Z. Ganzoni, Bilal Kicin, Lisa Reichert, Kacper J. Patej, Uxía Gómez-Bouzó, Wolfgang Guba, Peter J. McCormick, Tian Hua, Christian W. Gruber, Dmitry B. Veprintsev, James A. Frank, Uwe Grether, Erick M. Carreira

**Affiliations:** † Laboratorium für Organische Chemie, Eidgenössische Technische Hochschule Zürich, Vladimir-Prelog-Weg 3, 8093 Zürich, Switzerland; ‡ Faculty of Medicine & Health Sciences, University of Nottingham, Nottingham NG7 2UH, U.K.; § Centre of Membrane Proteins and Receptors (COMPARE), University of Birmingham and University of Nottingham, https://www.birmingham-nottingham.ac.uk/compare; ∥ Department of Chemical Physiology & Biochemistry, Oregon Health & Science University, Portland, Oregon 97239-3098, United States; ⊥ Institute of Biomolecular Chemistry, National Research Council, Via Campi Flegrei 34, 80078 Pozzuoli, Italy; # Center for Physiology and Pharmacology, Medical University of Vienna, Schwarzspanierstrasse 17, 1090 Vienna, Austria; ∇ iHuman Institute, ShanghaiTech University, Shanghai 201210, China; ○ Roche Pharma Research & Early Development, Roche Innovation Center Basel, F. Hoffmann-La Roche Ltd., 4070 Basel, Switzerland; ◆ Department of Pharmacology and Therapeutics, University of Liverpool, Ashton Street, Liverpool L69 3GE, U.K.; ¶ Vollum Institute, Oregon Health & Science University, Portland, Oregon 97239-3098, United States

## Abstract

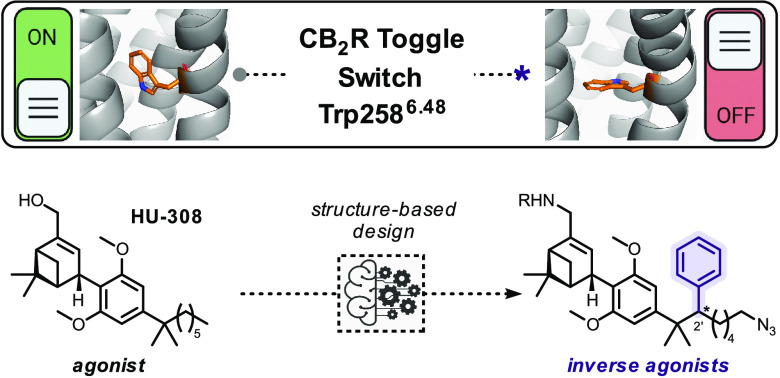

We report a blueprint for the rational design of G protein coupled receptor (GPCR) ligands with a tailored functional response. The present study discloses the structure-based design of cannabinoid receptor type 2 (CB_2_R) selective inverse agonists (*S*)-**1** and (*R*)-**1**, which were derived from privileged agonist HU-308 by introduction of a phenyl group at the *gem*-dimethylheptyl side chain. Epimer (*R*)-**1** exhibits high affinity for CB_2_R with *K*_d_ = 39.1 nM and serves as a platform for the synthesis of a wide variety of probes. Notably, for the first time these fluorescent probes retain their inverse agonist functionality, high affinity, and selectivity for CB_2_R independent of linker and fluorophore substitution. Ligands (*S*)-**1**, (*R*)-**1**, and their derivatives act as inverse agonists in CB_2_R-mediated cAMP as well as G protein recruitment assays and do not trigger β-arrestin–receptor association. Furthermore, no receptor activation was detected in live cell ERK_1/2_ phosphorylation and Ca^2+^-release assays. Confocal fluorescence imaging experiments with (*R*)-**7** (Alexa488) and (*R*)-**9** (Alexa647) probes employing BV-2 microglial cells visualized CB_2_R expressed at endogenous levels. Finally, molecular dynamics simulations corroborate the initial docking data in which inverse agonists restrict movement of toggle switch Trp258^6.48^ and thereby stabilize CB_2_R in its inactive state.

## Abstract 2

We report a generalizable strategy for structure-based agonist-to-inverse-agonist functional transformation and probe development by ligand modification that modulates the GPCR toggle switch of CB_2_R.

## Introduction

The endocannabinoid system is present in all vertebrates and comprises endogenous ligands, enzymes mediating ligand metabolism, transporters, and the two prominent cannabinoid receptors type 1 and type 2 (CB_1_R and CB_2_R).^1,2^ Exploitation of the therapeutic potential of CB_2_R has primarily focused on receptor activation with agonists and showed promise to ameliorate a plethora of diseases, such as autoimmune^3^ and metabolic disorders,^4,5^ chronic pain,^6^ and multiple sclerosis.^7^ By contrast, CB_2_R antagonists and inverse agonists remain vastly underexplored despite encouraging results in models of arthritis^8^ and neuroinflammation.^9−11^ Notably, CB_2_R antagonist TT-816 is currently being investigated in a phase II clinical trial as an immune checkpoint inhibitor for the treatment of solid tumors.^12^

Despite the current considerable endeavors to deliver selective CB_2_R therapeutics,^12−19^ to date there are no such drugs available on the market. Poor understanding of CB_2_R localization, expression, and signaling on the molecular level are key factors responsible for this absence.^20^ Elucidation of CB_2_R pharmacology has been hampered by the insufficient specificity of monoclonal antibodies^21−24^ and the scarcity of reliable chemical probes.^25^ Although some potent, selective, and validated fluorescent probes have been reported,^26−28^ these function as agonists that disturb cellular homeostasis by triggering downstream signaling and β-arrestin association, followed by agonist-mediated receptor internalization.^29,30^ These limitations may be addressed by implementation of inverse agonist based fluoroprobes that do not prompt receptor endocytosis. Additionally, inverse agonists tend to possess greater affinity for receptors in the more populous inactive G protein coupled receptor (GPCR) conformation yielding improved specificity and signal-to-noise ratio of probes compared to agonists.^31^

Historically, development of high-affinity, selective fluorescent CB_2_R inverse agonists has proven arduous. In the cases reported, fluorophore conjugation completely ablated^32^ or materially reduced^33^ affinity. In one example, a study of a series of agonists led to the identification of a specific linker–fluorophore construct endowing inverse agonism in a cAMP assay.^34^ Development of a potent, selective, and versatile CB_2_R-targeting inverse agonist scaffold that can be conjugated to a variety of fluorophores and functionalities remains an unmet challenge.

**Scheme 1:**
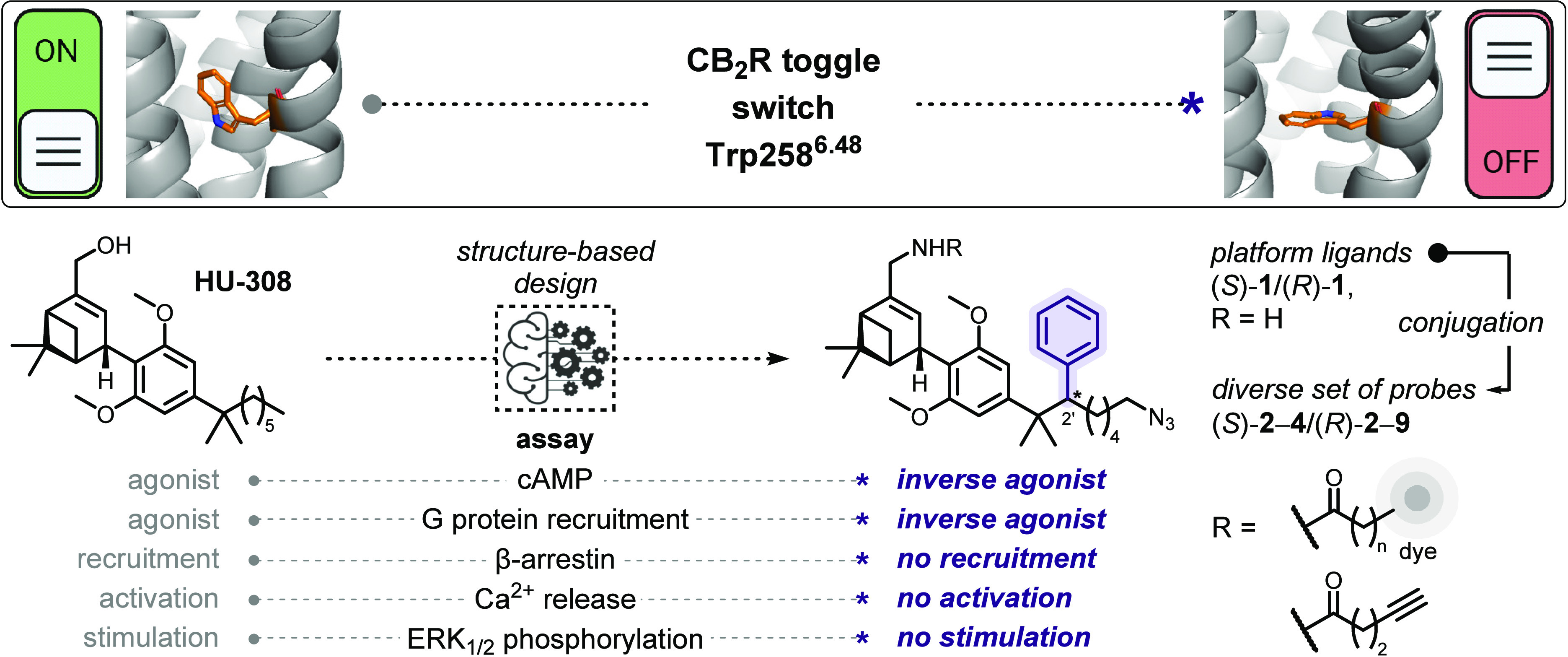
Novel Structure-Based Design of HU-308-Derived CB_2_R-Selective Inverse Agonists That Actuate Trp258^6.48^ Toggle Switch

Since its discovery in 1999,^35^ CB_2_R-selective agonist HU-308 (Scheme 1) has enjoyed privileged status for the study of CB_2_R pharmacology.^36^ HU-308 has been extensively applied to unravel effects of CB_2_R activation in animal models of pain,^37^ osteoporosis,^38^ Parkinson’s disease,^39^ and amyotrophic lateral sclerosis^40^ and is currently investigated in phase I clinical trials for mitigation of inflammation.^19^ The pharmacophore embedded in HU-308 has served in the development of photoswitchable,^41^ fluorescent,^28,42^ and ligand-directed covalent probes.^26^ On the basis of our prior work with HU-308, we focused on this scaffold with the intent of transforming its functional profile from agonist to inverse agonist with minimal structural modification. In this respect, Schapira and Jones have independently discussed the conceptual benefits of working with a set of molecules closely related in structure to enable in-depth understanding of receptor pharmacology.^43,44^

The past two decades witnessed the exponential rise of reported GPCR structures; hence, structure-based ligand design is at present ideally positioned to capitalize on the ongoing revolution.^45^ Since more than a third of all approved drugs exert their action by GPCR modulation, it is vital to comprehensively investigate and understand receptor pharmacology with functionally orthogonal chemical probes.^46^ GPCRs of the most populous class A family are distinguished by high homology of the CWxP motif. In particular, the toggle switch of CWxP that modulates receptor activation, Trp258^6.48^, is conserved within 78% of nonolfactory GPCRs.^47^ Examination of the X-ray structure of CB_2_R in its inactive conformation revealed a secondary binding pocket that hosts Trp258^6.48^. Further investigation by in silico docking suggested that addition of a substituent at C(2′) of HU-308 might constrain Trp258^6.48^ and hence modulate CB_2_R activation (Scheme 1).

We report novel inverse agonists that demonstrate avid binding at CB_2_R with excellent selectivity over the closely related CB_1_R. The compounds were profiled for their functional response in a comprehensive panel of in vitro (β-arrestin and G protein recruitment) as well as cellular (cAMP, ERK_1/2_ phosphorylation, Ca^2+^ signaling) assays. Remarkably, none of the probes activate CB_2_R-mediated signaling in any of the tested pathways. Fluorescent probes demonstrated excellent specificity and visualized CB_2_R expressed at endogenous levels in live-cell confocal microscopy experiments. Finally, molecular dynamics simulations investigated structural determinants that prevent receptor activation upon ligand binding and corroborate movement restriction of Trp258^6.48^. The workflow and key considerations described herein may be used to successfully drive future structure-based switch of functionality involving ligands and proteins beyond HU-308 and CB_2_R.

## Results and Discussion

### In Silico Probe Design

**Figure 1:**
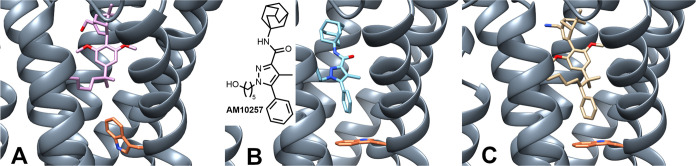
Comparison of active (A, PDB 8GUS, ligand HU-308)^48^ and inactive (B, PDB 5ZTY, ligand AM10257)^49^ CB_2_R conformations. (C) Docking study of HU-308-derived putative inverse agonist (*R*)-**1** in the inactive CB_2_R conformation (PDB 5ZTY). (*R*)-**1** reaches into the secondary pocket occupied by the toggle switch responsible for CB_2_R activation, Trp258^6.48^ (orange), and shares binding interactions virtually identical with those of AM10257.

We have investigated the recently published active (PDB 8GUS, Figure 1A)^48^ and inactive (PDB 5ZTY, Figure 1B)^49^ conformations of CB_2_R crystallized with agonist HU-308 and antagonist/inverse agonist AM10257, respectively. Close examination of the two receptor conformations revealed that AM10257 reaches into a secondary binding pocket that features a highly conserved CWxP motif in class A GPCRs^50^ and moreover hosts Trp258^6.48^, the recently designated single residue toggle switch of CB_2_R activation.^51^

**Figure 2:**
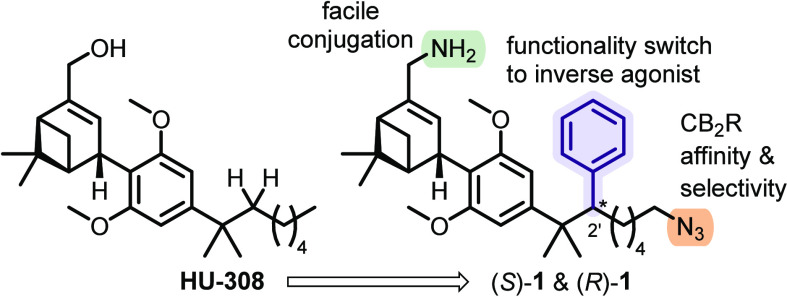
Design of inverse agonists (*S*)-**1** and (*R*)-**1**.

Comparison of the two receptor conformations combined with in silico docking suggested that a phenyl substituent introduced α to the *gem*-dimethyl group of HU-308 might occupy the same lipophilic subpocket as the phenyl of AM10257. The phenyl substitution creates a new C(2′) stereocenter at the pendent side chain; accordingly the explicit (*S*) and (*R*) designations preceding compound labels denote its absolute configuration. Additionally, structural features were incorporated that proved critical in our prior works to bestow excellent pharmacological profiles, yielding ligands (*S*)-**1** and (*R*)-**1** (Figure 2).^26,28,42^ Namely, terminal azide was inserted and the allylic alcohol was substituted by an amine to allow facile, stable conjugation to fluorophores and confer improved affinity and selectivity for CB_2_R. The novel putative HU-308-derived inverse agonist (*R*)-**1** showed binding interactions virtually identical with those of AM10257 in the inactive CB_2_R conformation (Figure 1C). In particular, the C(2′) phenyl group of (*R*)-**1** oriented toward Trp258^6.48^ and attained a favorable edge-to-face π-interaction similar to the phenyl of AM10257. The nearly identical interactions are essential as we have hypothesized that the impediment of the upward movement of Trp258^6.48^ may effectively prevent receptor activation.

### Synthesis

Access to (*S*)-**1** and (*R*)-**1** that feature a phenyl group at the homobenzylic position of cannabinoid scaffolds α to a sterically demanding *gem*-dimethyl group is synthetically challenging and unprecedented. Prior structure–activity relationship studies on cannabinoid ligands focused almost exclusively on the easily accessible benzylic or ω-position of the pendent side chain.^52^ To the best of our knowledge, there is only a single report of substitution at the homobenzylic position with a methyl group in a structure of Δ^8^-THC that lacks the sterically congesting *gem*-dimethyl motif.^53^

**Scheme 2:**
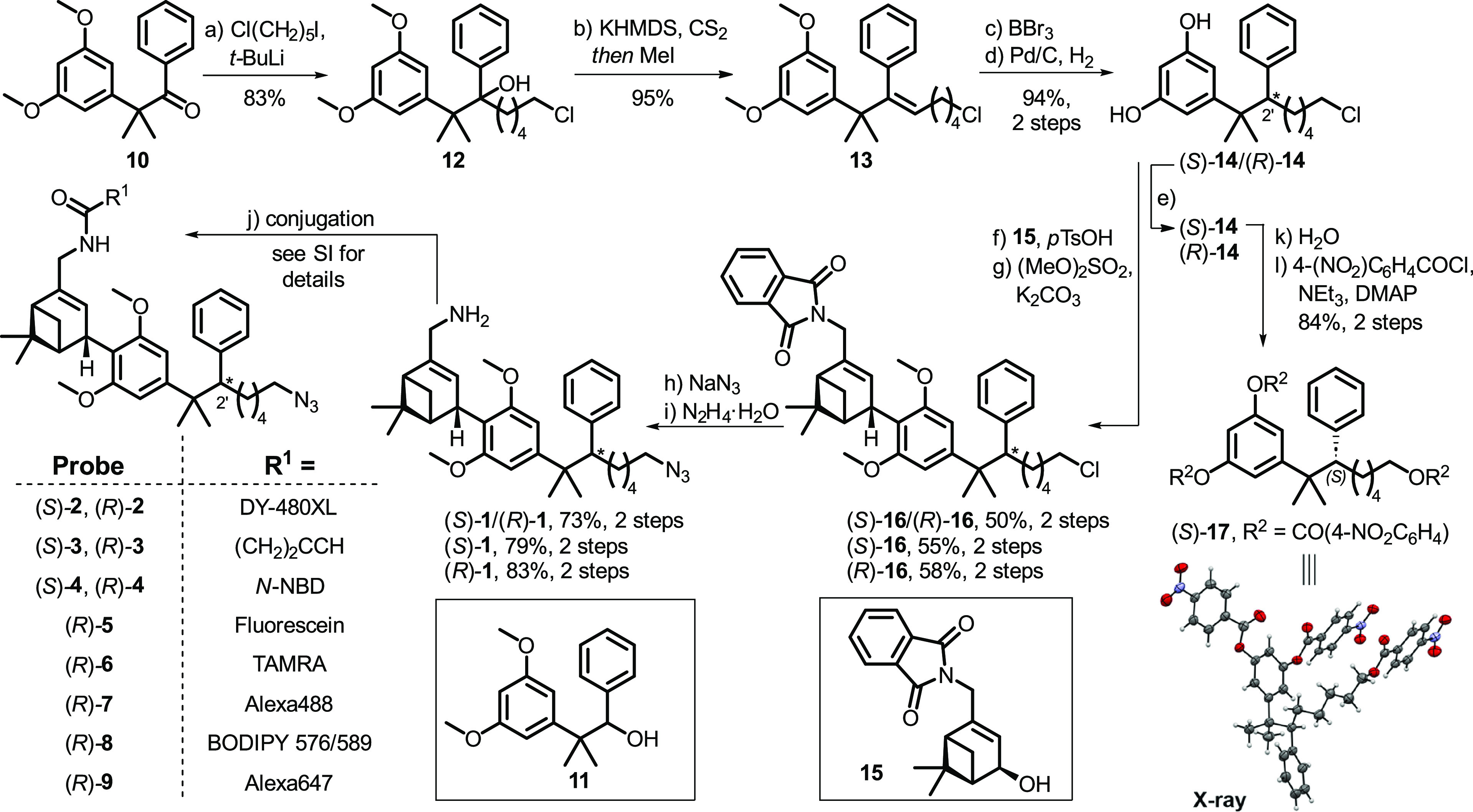
Synthesis of Novel CB_2_R-Selective HU-308-Derived Inverse Agonists Reagents and conditions: (a) 1-chloro-5-iodopentane, *t*-BuLi, *n*-pentane, Et_2_O, −78 °C to rt, 83%; (b) KHMDS, CS_2_, THF, −78 °C to rt and then MeI, 40 °C, 95%; (c) BBr_3_, CH_2_Cl_2_, 0 °C, 97%; (d) Pd/C, H_2_, EtOAc, rt, 97%; (e) semipreparative SFC, (*S*)-**14**, 25%, >99% ee, (*R*)-**14**, 20%, 96% ee; (f) **15**, *p*TsOH·H_2_O, CH_2_Cl_2_, rt, 64–71%; (g) (MeO)_2_SO_2_, K_2_CO_3_, acetone, rt, 78–82%; (h) NaN_3_, DMF, 50 °C, 88–96%; (i) N_2_H_4_·H_2_O, (*E*)/(*Z*)-crotyl alcohol, EtOH, 75 °C, 76–94%; (j) for conjugation conditions and details, see Supporting Information; (k) H_2_O, microwave irradiation, 150 °C, 99%; (l) 4-nitrobenzoyl chloride, NEt_3_, DMAP, CH_2_Cl_2_, rt, 85%.

The synthesis of (*S*)-**1**/(*R*)-**1** commenced with **10**, which was prepared by methylation of 3,5-dimethoxyphenylacetonitrile and subsequent treatment with phenyl lithium (Scheme 2).^54,55^ Introduction of the alkyl side chain to ketone **10** proved a formidable challenge due to steric hindrance. Initial attempts using established phosphonium ylide routes yielded no reaction even at elevated temperatures.^56,57^ An extensive screening of Grignard reagents either yielded no reaction or afforded exclusively Grignard reduction product **11**. Finally, using a modified procedure for the preparation of alkyl lithiums by Punzalan,^58^ we employed, for the first time, 5-chloropentyl lithium to forge the tertiary alcohol **12** in 83% yield.^59^ Subsequent Chugaev elimination of the benzylic alcohol under mild conditions yielded **13** in 95% yield. BBr_3_-mediated demethylation followed by high-pressure hydrogenation over Pd/C afforded (*S*)-**14**/(*R*)-**14** as a racemic mixture in 94% yield over two steps. The synthesis was continued with the racemate to rapidly access material for initial pharmacological evaluation. To this end, Friedel–Crafts allylation with verbenol derivative **15** followed by treatment with (MeO)_2_SO_2_ furnished methylated epimeric mixture (*S*)-**16**/(*R*)-**16** in 50% yield over the two steps. Subsequent substitution of the primary alkyl chloride with NaN_3_ and hydrazine-mediated phthalimide deprotection revealed allylic amines (*S*)-**1**/(*R*)-**1** in 73% yield. Finally, the synthesis was concluded by functionalization of the diastereomeric mixture (*S*)-**1**/(*R*)-**1** with DY-480XL or 4-pentynoic acid to yield mixtures of epimers (*S*)-**2**/(*R*)-**2** and (*S*)-**3**/(*R*)-**3**, respectively.

We then investigated access to epimers (*S*)-**1** and (*R*)-**1** separately. Following introduction of verbenol fragment **15**, screening of conditions to separate the resulting epimers ((*S*)-**16**/(*R*)-**16**) by silica gel chromatography, HPLC, and supercritical fluid chromatography (SFC) proved unsuccessful. Gratifyingly, we found that enantiomers (*S*)-**14**/(*R*)-**14** could be separated by semipreparative SFC using a chiral stationary phase to yield (*S*)-**14** and (*R*)-**14** in >99% ee and 96% ee, respectively. Resorcinols (*S*)-**14** and (*R*)-**14** were then functionalized to yield enantio- and diastereomerically pure (*S*)-**1**–(*S*)-**4** and (*R*)-**1**–(*R*)-**9**. To assign the absolute configuration at the C(2′) stereocenter, (*S*)-**14** was converted to a *p*-nitrobenzoate (*S*)-**17**, whose structure was elucidated by X-ray crystallography.

### Pharmacological Profiling

#### Saturation Binding Assays

We assessed whether the phenyl substitution in (*S*)-**1**, (*R*)-**1**, and their derivatives (*S*)-**2**–(*S*)-**4** and (*R*)-**2**–(*R*)-**9** impedes interaction with CB_2_R. To this end, time-resolved Förster resonance energy transfer (TR-FRET) binding assay was employed to determine the affinities of the new probes at room temperature.^28^ HEK293 membrane preparations of SNAP-Lumi4-Tb labeled hCB_2_R were incubated with a fluorescent probe in the presence or absence of a validated inverse agonist, SR-144,528,^36^ to determine its binding parameters. Gratifyingly, the epimeric mixture (*S*)-**2**/(*R*)-**2** demonstrated good affinity for CB_2_R (*K*_d_ = 67.9 nM), suggesting that the C(2′) functionalization was well tolerated and validated the in silico guided design.

Encouraged by the promising result, we studied the impact of configuration at the C(2′) stereocenter on the pharmacological properties by examining each epimer individually. A 12-fold greater binding affinity for CB_2_R was shown by (*R*)-**1** (*K*_d_ = 39.1 nM) in comparison to (*S*)-**1** (*K*_d_ = 476 nM). Functionalization of (*S*)-**1** and (*R*)-**1** with 4-pentynoic acid was well tolerated, and the resulting compounds, (*S*)-**3** and (*R*)-**3**, retained the stereoisomeric preference with *K*_d_ = 2.10 and 0.42 nM, respectively. Conjugation of (*S*)-**1** and (*R*)-**1** with DY-480XL and *N*-NBD yielded probes (*S*)-**2** and (*R*)-**2** and (*S*)-**4** and (*R*)-**4**, respectively. Fluoroprobes (*S*)-**2** and (*S*)-**4** displayed inferior CB_2_R affinity (*K*_d_ = 162 and 158 nM, respectively) compared to the excellent binding potencies of (*R*)-**2** and (*R*)-**4** (*K*_d_ = 10.2 and 12.3 nM, respectively). Furthermore, strong agreement was observed between CB_2_R *K*_d_ and *K*_i_ values obtained by independent TR-FRET and radioligand binding assays for (*R*)-**2** (*K*_d_ = 10.2 nM and *K*_i_ = 8.26 nM) and (*R*)-**3** (*K*_d_ = 0.42 nM and *K*_i_ = 0.66 nM). Collectively, the results further validate the TR-FRET assay and imply that the orthosteric binding pocket of CB_2_R shows preference for the *R*-epimer of the parent compound and its derivatives.

**Figure 3:**
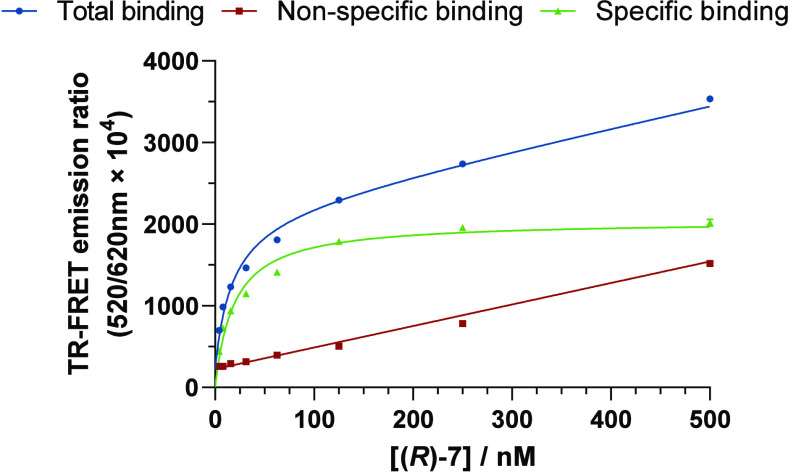
TR-FRET-based saturation binding profile of (*R*)-**7** (Alexa488) at CB_2_R determined at 37 °C. Nonspecific binding was determined in the presence of SR-144,528 (10 μM). Data shown as a mean ± SEM, *N* = 3.

We then set out to investigate whether the excellent CB_2_R affinities of (*R*)-**1**–(*R*)-**4** are impacted by linker and fluorophore substitution. To this end, probes (*R*)-**5**–(*R*)-**9** were prepared that feature a variety of linker lengths and fluorophores, spanning a wide range of size, lipophilicity, and membrane permeability. When tested by TR-FRET at 37 °C, fluorescein, tetramethylrhodamine (TAMRA), and Alexa488 bearing probes (*R*)-**5**, (*R*)-**6**, and (*R*)-**7** all emerged as high affinity binders for CB_2_R with excellent *K*_d_ values of 30.3, 2.78, and 24.9 nM, respectively (Table 1). BODIPY 576/589 conjugate (*R*)-**8** showed good binding potency with CB_2_R, *K*_d_ = 44.7 nM. Particularly remarkable was the retention of high affinity displayed by probe (*R*)-**9** (*K*_d_ = 25.9 nM) functionalized with Alexa647. These results illustrate substantial improvement over previous work with agonists where functionalization with the highly polar Alexa488 and sterically demanding Alexa647 led to 64-fold and 611-fold drops in affinity, respectively.^28^ Importantly, fluoroprobes (*R*)-**5**, (*R*)-**6**, (*R*)-**7**, and (*R*)-**9** emit robust fluorescence signals with exquisite specific binding windows when tested at the physiologically relevant temperature, 37 °C (see Figure 3 and Figure S1).

**Table 1:** TR-FRET-Based Profiling of Binding Affinity^a^

		*K*_d_ [nM]		
probe	dye	CB_2_R	CB_1_R	*K*_d_ ratio (CB_1_R/CB_2_R)
(*R*)-**2**	DY-480XL	18.9	1740	92
(*R*)-**5**	fluorescein	30.3	1280	42
(*R*)-**6**	TAMRA	2.78	396	142
(*R*)-**7**	Alexa488	24.9	3300	133
(*R*)-**9**	Alexa647	25.9	7050	272

a Saturation binding data (*K*_d_) were determined in a TR-FRET assay at 37 °C with membrane preparations from either hCB_2_R-HEK293 or hCB_1_R-HEK293 cells. Data shown as a mean, *N* = 3.

The binding selectivity of fluorescent probes was tested against the closely related CB_1_R in a saturation binding assay at 37 °C using membrane preparations derived from HEK293 cells expressing hCB_1_R (Table 1). Fluoroprobes (*R*)-**2**, (*R*)-**5**, (*R*)-**6**, and (*R*)-**7** displayed 42–142-fold selectivity for CB_2_R over CB_1_R. Notably, (*R*)-**9** demonstrated an exceptional 272-fold preference for CB_2_R over CB_1_R. Collectively, the excellent affinity and selectivity of a range of physicochemically distinct substituents and fluorophores highlight the versatility of novel platform ligand (*R*)-**1**.

#### Kinetic Binding TR-FRET Assay

We were intrigued by the performances of our probes in the saturation binding assay and leveraged TR-FRET to study ligand binding kinetics at a physiologically relevant temperature, 37 °C (Table 2). The results suggest that all compounds, except (*S*)-**1**, possess dramatically slower receptor dissociation rates, *k*_off_, in comparison to the control inverse agonist, SR-144,528. Therefore, high CB_2_R affinity of the probes once bound stems from slow receptor dissociation rates. The probes are thus endowed with long receptor residence times, τ, an attribute that has been argued particularly important for GPCRs^60^ as a better suited determinant, compared to *K*_d_, of ligand–protein interactions in living systems.^61,62^ Importantly, excellent agreement was found between *K*_d_ values obtained in saturation and kinetic binding experiments.

**Table 2:** TR-FRET-Based Kinetic Profiling at CB_2_R^a^

probe	*k*_on_ [10^6^ M^–1^ min^–1^]	*k*_off_ [10^–2^ min^–1^]	τ [min]	kinetic *K*_d_ [nM]
(*S*)-**1**	9.39	115	0.87	122
(*R*)-**1**	12.0	13.9	7.19	11.6
(*S*)-**3**	44.3	4.96	20.2	1.12
(*R*)-**3**	88.7	2.29	43.7	0.26
(*R*)-**6**	3.60	1.08	92.6	3.00
(*R*)-**7**	1.49	2.37	42.2	15.9
(*R*)-**9**	1.75	1.71	58.5	9.77
SR-144,528	240	122	0.82	5.08

a Kinetic *K*_d_ data were measured at 37 °C in a TR-FRET assay using hCB_2_R-HEK293 membrane preparations. Data shown as a mean, *N* = 3.

### Functional Profiling: cAMP, G Protein Recruitment, and β-Arrestin

HU-308 is a potent full agonist at CB_2_R in the [^35^S]-GTPγS assay, triggers inhibition of cAMP production, promotes recruitment of β-arrestin, stimulates ERK_1/2_ phosphorylation, and facilitates release of Ca^2+^ from intracellular stores.^36,41,63^ Compounding evidence indicates that distinct CB_2_R agonists favor discrete receptor conformations, leading to preferential activation of one specific signaling pathway over another, a phenomenon known as biased agonism.^36,64−66^ Accordingly, we have dedicated substantial efforts to comprehensively profile the pharmacological responses elicited by the new probes across known CB_2_R signaling pathways.

One of the canonical signaling pathways of CB_2_R involves association with Gα_i/o_ proteins, which elicit reversible inhibition of adenylyl cyclase resulting in a decrease of cellular cAMP levels and suppression of protein kinase A activity.^67^ Consequently, we have investigated the change in cAMP levels upon probe addition using homogeneous time-resolved fluorescence (HTRF) cAMP assay (Table 3).

**Table 3:** Functional Characterization in a CB_2_R cAMP Assay^a^

probe	pEC_50_	*E*_max_ [%]
(*S*)-**1**	5.57	–44
(*R*)-**1**	6.95	–44
(*S*)-**2**	6.47	–37
(*R*)-**2**	7.15	–31
(*S*)-**3**	7.39	–49
(*R*)-**3**	7.48	–55
(*S*)-**4**^b^	4.98	+44
(*R*)-**4**	5.91	–40
**ago-3**	8.47	+112

a Potency (pEC_50_) and *E*_max_ data were obtained in a cAMP HTRF assay using hCB_2_R-CHO cells. Data were normalized to agonist CP-55,940 response (100%) and basal level (0%), unless noted otherwise.

b Data were normalized to the response of inverse agonist AM10257 (0%) and basal level (100%). Data shown as a mean, *N* = 3.

All compounds behaved as inverse agonists, with efficacy (*E*_max_) ranging between −31 and −55%. Both epimers of the parent amine ligand inhibited cAMP production with the (*R*)-**1** stereoisomer demonstrating greater potency (pEC_50_ = 6.95) than (*S*)-**1** (pEC_50_ = 5.57). DY-480XL and alkyne functionalized probes, (*R*)-**2** (pEC_50_ = 7.15) and (*R*)-**3** (pEC_50_ = 7.48), were favored over (*S*)-**2** (pEC_50_ = 6.47) and (*S*)-**3** (pEC_50_ = 7.39) with respect to potency, albeit to a lesser degree. Interestingly, the *N*-NBD probes (*S*)-**4** and (*R*)-**4** behaved as inverse agonists only at high concentration (pEC_50_ = 4.98 and 5.91, respectively). As a control, we prepared HU-308-derived agonist probe **ago-3** from **ago-1** that features the same scaffold as (*S*)-**3** and (*R*)-**3** except that it lacks the C(2′) phenyl substituent (eq 1). In the cAMP assay **ago-3** displayed potent receptor activation (pEC_50_ = 8.47, *E*_max_ = 112%). These results provide direct experimental evidence as to the critical role of the phenyl substituent in facilitating the switch in ligand functionality from agonist to inverse agonist.
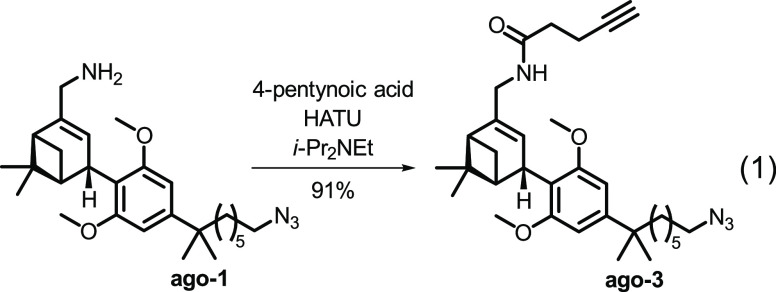
1

**Figure 4:**
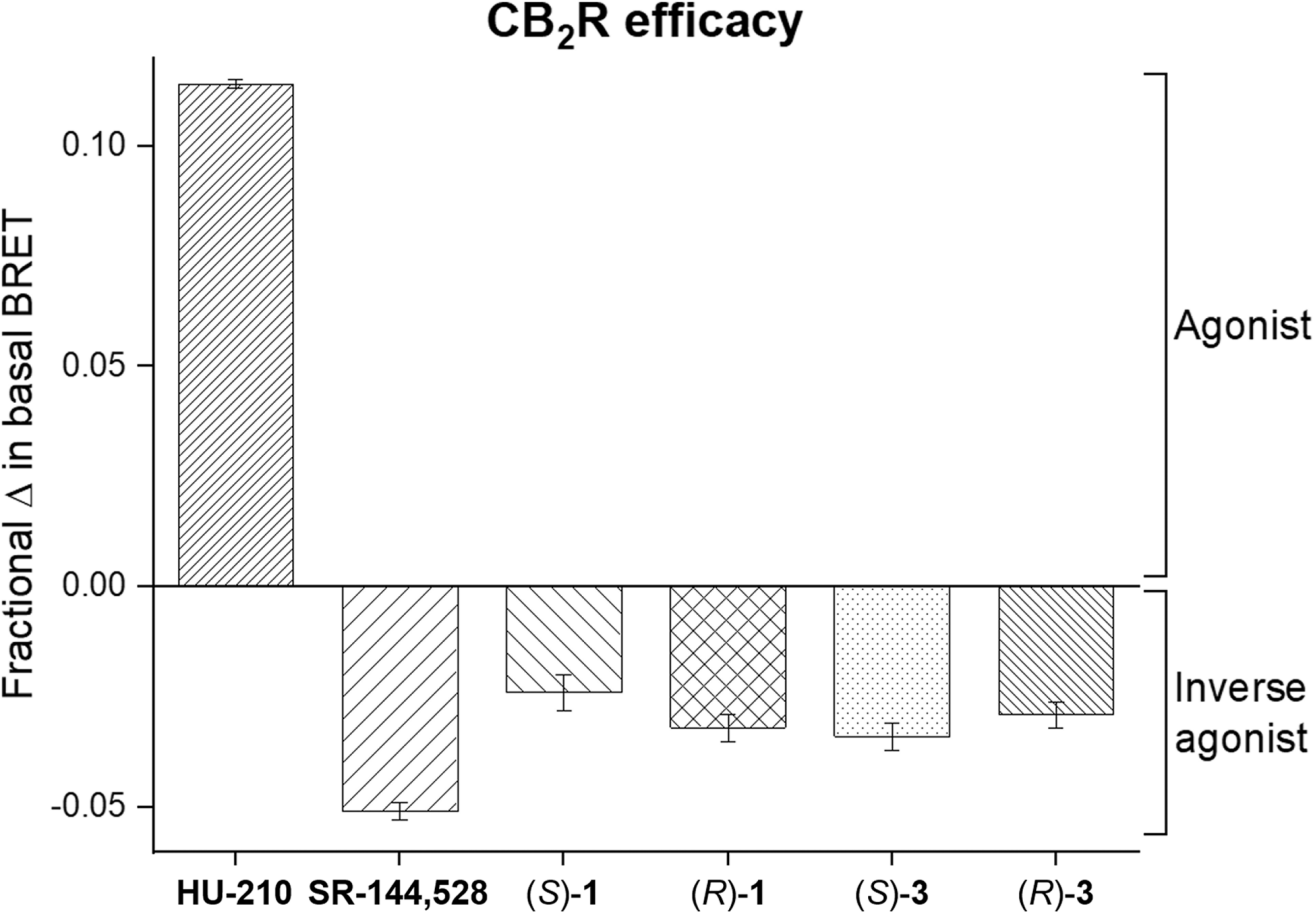
BRET-based Gi-CASE membrane assay to characterize G protein recruitment at CB_2_R. Efficacy, *E*_max_, of the compounds is shown as a mean ± SEM, *N* = 3–4.

To complement the functional response elicited in the cAMP assay, we tested whether the probes trigger association of Gα_i_ protein with CB_2_R using our recently reported bioluminescence resonance energy transfer (BRET) Gi-CASE assay.^68^ Membrane preparations harvested from hCB_2_R-HEK293 T-REx cells that genetically incorporate fluorescent NanoLuciferase donor and Venus acceptor proteins to the Gα and Gγ subunits, respectively, were incubated with a probe, and the change in BRET signal was detected. Agonist binding triggers CB_2_R activation and dissociation of the Gα and Gβγ subunits resulting in BRET signal reduction. Conversely, inverse agonists elicit increase in BRET intensity by stabilization of inactive CB_2_R conformation and G protein accumulation beyond the basal level. Compounds (*S*)-**1**, (*R*)-**1**, (*S*)-**3**, and (*R*)-**3** were selected as representatives to circumvent interference among fluorophores in the BRET assay as previously reported.^34^ The results indicate that all tested compounds behave as potent inverse agonists with respect to G protein recruitment at CB_2_R (Figure 4 and Table 4).

**Table 4:** Functional Characterization of G Protein Recruitment at CB_2_R in a BRET Gi-CASE Assay^a^

probe	pEC_50_	*E*_max_ [%]
(*S*)-**1**	6.72	–21
(*R*)-**1**	6.80	–28
(*S*)-**3**	7.51	–30
(*R*)-**3**	7.22	–25
SR-144,528	8.23	–45

a Potency (pEC_50_) and *E*_max_ data were obtained in a Gi-CASE BRET-based assay using membrane preparations from hCB_2_R-HEK293 T-REx cells. Data were normalized to agonist HU-210 response (100%) and basal level (0%). Data are shown as mean, *N* = 3–4.

Alkyne functionalized probes (*S*)-**3** and (*R*)-**3** (pEC_50_ = 7.51 and 7.22, respectively) have shown superior potency in comparison to free amines (*S*)-**1** and (*R*)-**1** (pEC_50_ = 6.72 and 6.80, respectively). Control agonist HU-210 and inverse agonist SR-144,528 demonstrated potency consistent with previously reported [^35^S]-GTPγS binding assay values, further validating the experimental results (pEC_50_ = 8.83 and 8.23, respectively).^36,69^ With respect to efficacy (*E*_max_), probes (*S*)-**1**, (*R*)-**1**, (*S*)-**3**, and (*R*)-**3** induced functional responses between −21 and −30%. Remarkably, comparison of the effects elicited by (*S*)-**1** in the cAMP and Gi-CASE assays (pEC_50_ = 5.57 and 6.72, respectively) suggests 14-fold increased potency of G protein recruitment over adenylyl cyclase inhibition, a striking bias within a CB_2_R–Gα_i_-mediated pathway.

**Figure 5:**
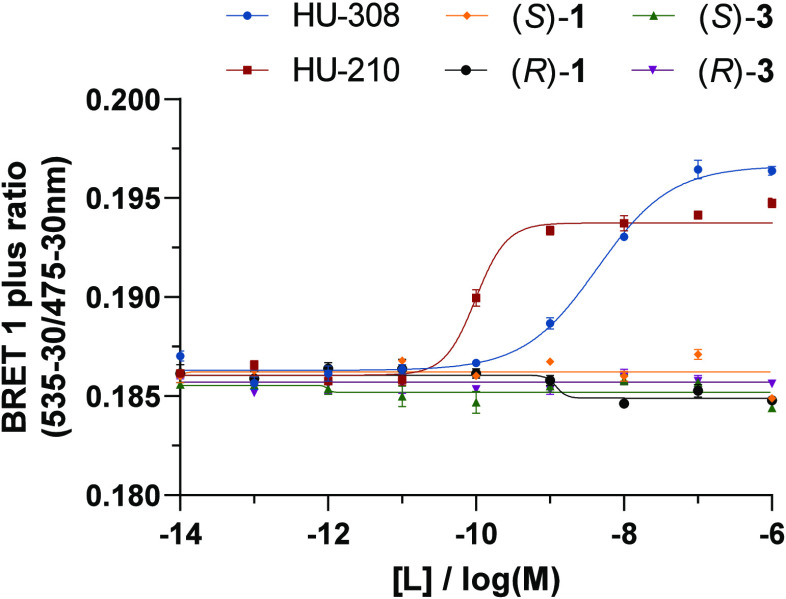
BRET-based assay to characterize β-arrestin recruitment at CB_2_R. L = ligand. Data are a representative of *N* = 3.

Among the best studied G protein independent signaling pathways of CB_2_R is the β-arrestin cascade. β-Arrestins bind activated CB_2_R following receptor phosphorylation, block further G protein mediated signaling, and destine the receptor for internalization.^30^ Representative compounds were profiled for β-arrestin recruitment in a BRET assay where an increase of the BRET ratio corresponds to recruitment of β-arrestin. Baseline BRET signal was retained by (*S*)-**1**, (*R*)-**1**, (*S*)-**3**, and (*R*)-**3** (Figure 5). In contrast, control agonists HU-308 and HU-210 showed expected recruitment of β-arrestin to CB_2_R as indicated by increases in BRET intensity (pEC_50_ = 8.37 and 10.0, respectively). These results imply that (*S*)-**1**, (*R*)-**1**, (*S*)-**3**, and (*R*)-**3** do not activate CB_2_R toward β-arrestin recruitment.

### Phosphorylation of ERK

**Figure 6:**
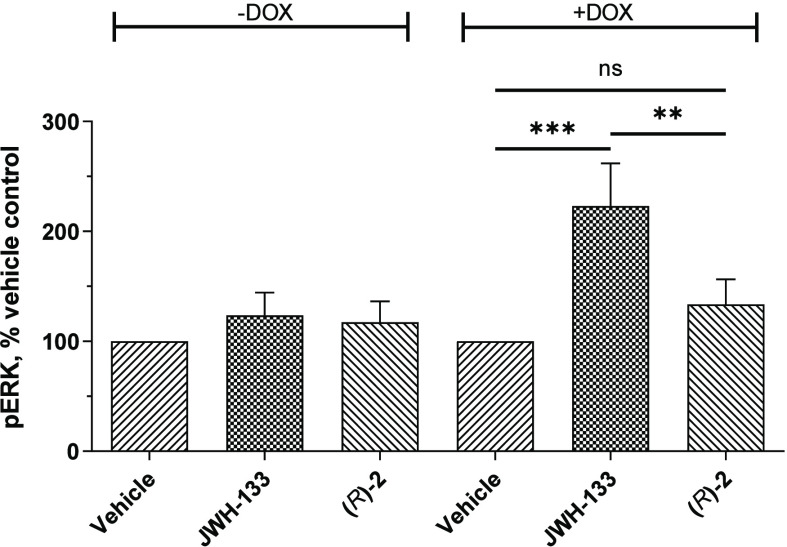
Live cell AlphaScreen SureFire phospho-ERK assay with CB_2_R inducible breast cancer HCC1954 cell line. Cells were optionally induced with doxycycline (DOX) for 24 h to stimulate expression of CB_2_R followed by incubation with a vehicle (0.1% DMSO), agonist JWH133^72^ (1 μM), or (*R*)-**2** (1 μM) for 30 min. Statistical significance was examined by one-way ANOVA followed by Tukey’s *post hoc* test. ns = nonsignificant, ∗∗, *p* < 0.01; ∗∗∗, *p* < 0.001. Data are an average of three independent biological replicates.

Activation of CB_2_R is associated with downstream stimulation of mitogen-activated protein kinases, such as ERK_1/2_, mediated via either Gβγ or β-arrestins.^70,71^ We have tested representative high-affinity fluorescent probe (*R*)-**2** for CB_2_R-mediated phosphorylation of endogenous ERK_1/2_ in a CB_2_R inducible breast cancer HCC1954 cell line using the AlphaScreen SureFire phospho-ERK assay (Figure 6). Expression of CB_2_R was optionally induced with doxycycline (DOX), and after 24 h the cells were incubated with a vehicle (0.1% DMSO), CB_2_R selective agonist JWH133^72^ (1 μM), or (*R*)-**2** (1 μM) for 30 min. Following cell lysis, lysates were incubated with a mixture containing donor and acceptor beads for 2 h at room temperature and the luminescence emission signal was measured.

In the absence of CB_2_R expression inducer (DOX), the phosphorylation levels of ERK_1/2_ remained the same for cells treated with a vehicle, agonist JWH133, and (*R*)-**2**. In cells induced to express CB_2_R with DOX (1 μg/mL), JWH133 effectively stimulated ERK_1/2_ phosphorylation mediated by CB_2_R activation, in agreement with previously reported findings.^73^ Addition of (*R*)-**2** had no effect on the level of phosphorylated ERK_1/2_, which remained the same as for a vehicle. Data of the phospho-ERK cellular assay imply that (*R*)-**2** does not induce phosphorylation of ERK_1/2_ by either CB_2_R-mediated Gβγ or β-arrestin signaling (or by non-CB_2_R-mediated pathways).

### Ca^2+^ Signaling

Upon activation of CB_2_R, Ca^2+^ is often released from intracellular reservoirs.^74−76^ Our previous work reported that HU-308 and its photoswitchable derivative, *azo*-HU-308, increase intracellular Ca^2+^ in the mouse AtT-20 cell line.^41^ Naturally, we were intrigued to investigate the response elicited by our probes. The epimeric mixture (*S*)-**3**/(*R*)-**3** was chosen to avoid fluorophore interference with the Fluo-4AM Ca^2+^ dye and test both diastereomers simultaneously.

**Figure 7:**
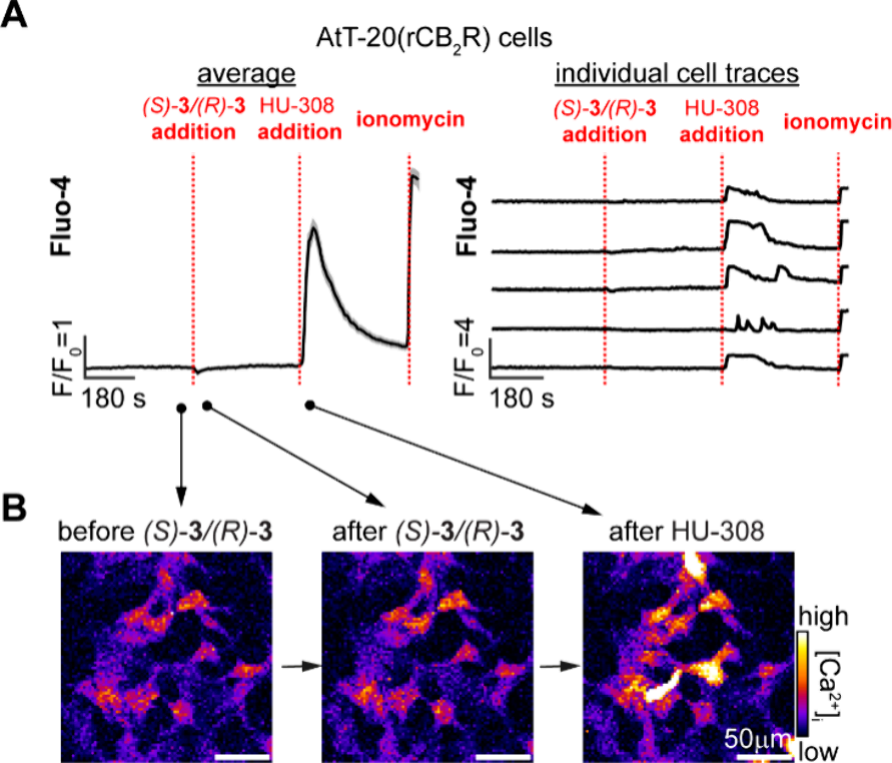
Live cell fluorescent Ca^2+^ imaging in rat CB_2_R overexpressing AtT-20 cells [AtT-20(rCB_2_R)] loaded with Fluo-4AM (2 μM). After initial equilibration, (*S*)-**3**/(*R*)-**3** (20 μM) was added, followed by HU-308 (20 μM) and ionomycin (10 μM). Shown are the average responses of 200 cells (A, left), individual traces of five representative cells (A, right), and representative fluorescence images from different time points (B). Averaged data plotted as mean ± SEM, *T* = 4.

Live AtT-20 cells overexpressing rat CB_2_R [AtT-20(rCB_2_R)] were treated with Fluo-4AM Ca^2+^ dye and imaged by confocal microscopy (Figure 7). Addition of (*S*)-**3**/(*R*)-**3** (20 μM) did not elicit increase in Fluo-4AM fluorescence, whereas subsequent addition of agonist HU-308 (20 μM) triggered a robust fluorescence spike. Ionomycin was added at the end of the experiment to saturate Ca^2+^ levels. This result indicates that neither (*S*)-**3** nor (*R*)-**3** induces Ca^2+^ release via CB_2_R activation and suggests that the probes can be displaced by HU-308.

### Fluorescence Confocal Microscopy in Live Cells

Having validated that the probes do not trigger CB_2_R signaling at multiple downstream pathways, we employed (*R*)-**7** and (*R*)-**9** to visualize CB_2_R by confocal fluorescence microscopy. Probes (*R*)-**7** and (*R*)-**9** were selected due to their bright, photostable, and extensively applied fluorophores Alexa488 and Alexa647. Additionally, the green- and red-shifted fluorescence spectra of (*R*)-**7** and (*R*)-**9** provide flexibility and potential for synergy with additional fluorescent proteins and small molecule dyes for multiplexed imaging studies.

**Figure 8:**
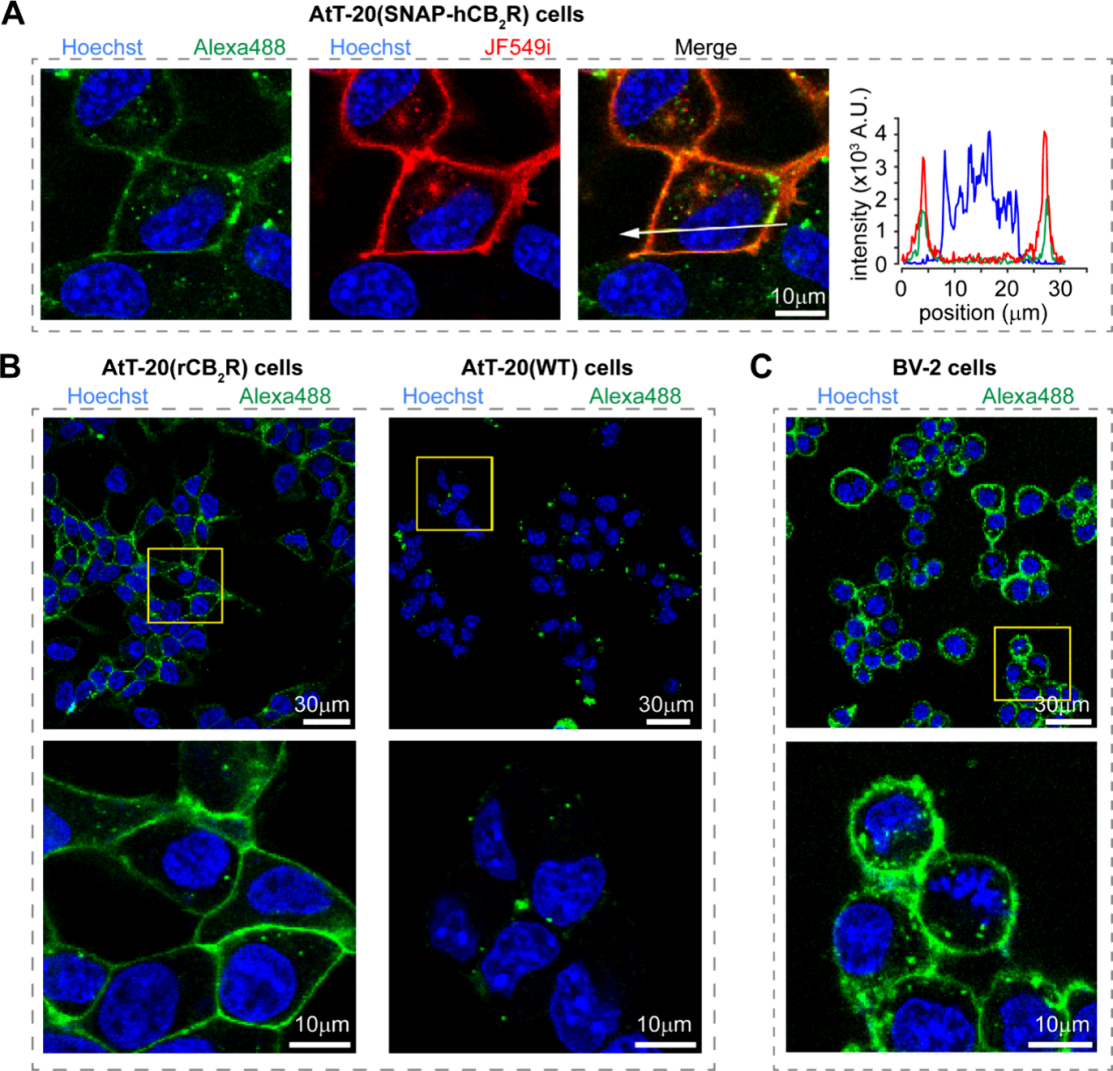
Confocal imaging of (*R*)-**7** in live cell lines. (A) AtT-20(SNAP-hCB_2_R) cells were labeled for 15 min with (*R*)-**7** (Alexa488, 625 nM, green), SNAP-JF549i (JF549i, 500 nM, red), and Hoechst33342 (Hoechst, 1 μM, blue) to visualize CB_2_R, SNAP-tags, and nuclei, respectively. Fluorescence intensity profiles across the white line for Alexa488, JF549i, and Hoechst33342 are shown on the right. (B) AtT-20(rCB_2_R) cells (left) and AtT-20(WT) cells (right) were treated with (*R*)-**7** (625 nM, green) and Hoechst33342 (1 μM, blue) for 15 min and imaged by confocal microscopy. (C) Live BV-2 microglial cells that endogenously express CB_2_R were incubated with (*R*)-**7** (2.5 μM, green) and Hoechst33342 (1 μM, blue) for 15 min and imaged by confocal microscopy.

First, AtT-20 cells stably expressing *N*-terminal SNAP-tagged human CB_2_R [AtT-20(SNAP-hCB_2_R)] were coincubated with (*R*)-**7**, Janelia Fluor SNAP-549i (JF549i), and Hoechst33342 to label CB_2_R, SNAP-tags, and nuclei, respectively. Confocal microscopy revealed bright fluorescence of Alexa488 and JF549i delineating the plasma membranes of AtT-20 cells (Figure 8A). Analysis of the corresponding intensity plot showed virtually identical colocalization overlap between the Alexa488 ((*R*)-**7**) and JF549i (SNAP-hCB_2_R) signals (Figure 8A).

Since many cannabinoid ligands tend to accumulate in plasma membranes due to their lipophilic nature, specificity of (*R*)-**7** and (*R*)-**9** for CB_2_R was evaluated using AtT-20(rCB_2_R) and AtT-20 wild-type (WT) cells, which do not express CB_2_R.^77^ AtT-20(rCB_2_R) and AtT-20(WT) cells were incubated with (*R*)-**7** and Hoechst33342 and imaged by confocal microscopy. A robust Alexa488 fluorescence signal was detected at the plasma membrane of AtT-20(rCB_2_R) cells (Figure 8B, left). In stark contrast, AtT-20(WT) cells showed only minimal background fluorescence with no signal stemming from the cellular membrane (Figure 8B, right). These results confirm that (*R*)-**7** specifically labels CB_2_R at the plasma membrane.

Encouraged by the promising results with (*R*)-**7** in cells overexpressing CB_2_R, we proceeded to investigate the probes’ ability to detect CB_2_R at endogenous expression levels. To this end, the murine derived BV-2 microglial cell line was selected due to its extensive use as a high fidelity, primary microglia culture model^78^ that was applied in the study of neurodegeneration and neuroinflammation.^79−81^ Importantly, BV-2 cells endogenously express CB_2_R.^82,83^ Following incubation of BV-2 cells with (*R*)-**7** and Hoechst33342, an intense Alexa488 signal was observed at the plasma membrane across BV-2 cells (Figure 8C). These results confirm that (*R*)-**7** can visualize CB_2_R at endogenous expression levels. Importantly, when (*R*)-**9** (Alexa647) was subjected to analogous experiments, it demonstrated equal specificity for CB_2_R in AtT-20 cells (see Figure S2A,B) combined with strong signal intensity in the BV-2 microglial cell line (see Figure S2C). Finally, these data imply that the performances of (*R*)-**7** and (*R*)-**9** remain uncompromised by interspecies differences and the probes can be employed to investigate both human and murine orthologs of CB_2_R.

### Molecular Dynamics Simulations Unravel Pharmacophore Determinants of Receptor Activation

Molecular dynamics (MD) studies were performed in a membrane environment with inverse agonists (*S*)-**3** and (*R*)-**3** and their agonist counterpart **ago-3** to contrast their interactions with CB_2_R at a molecular level and elucidate their orthogonal functional profiles. To this end, the X-ray structure of CB_2_R in an inactive state in complex with AM10257 (PDB 5ZTY) was selected as a starting point for 1 μs MD simulations to assess ligand stability and identify rearrangements within the binding site.

**Figure 9:**
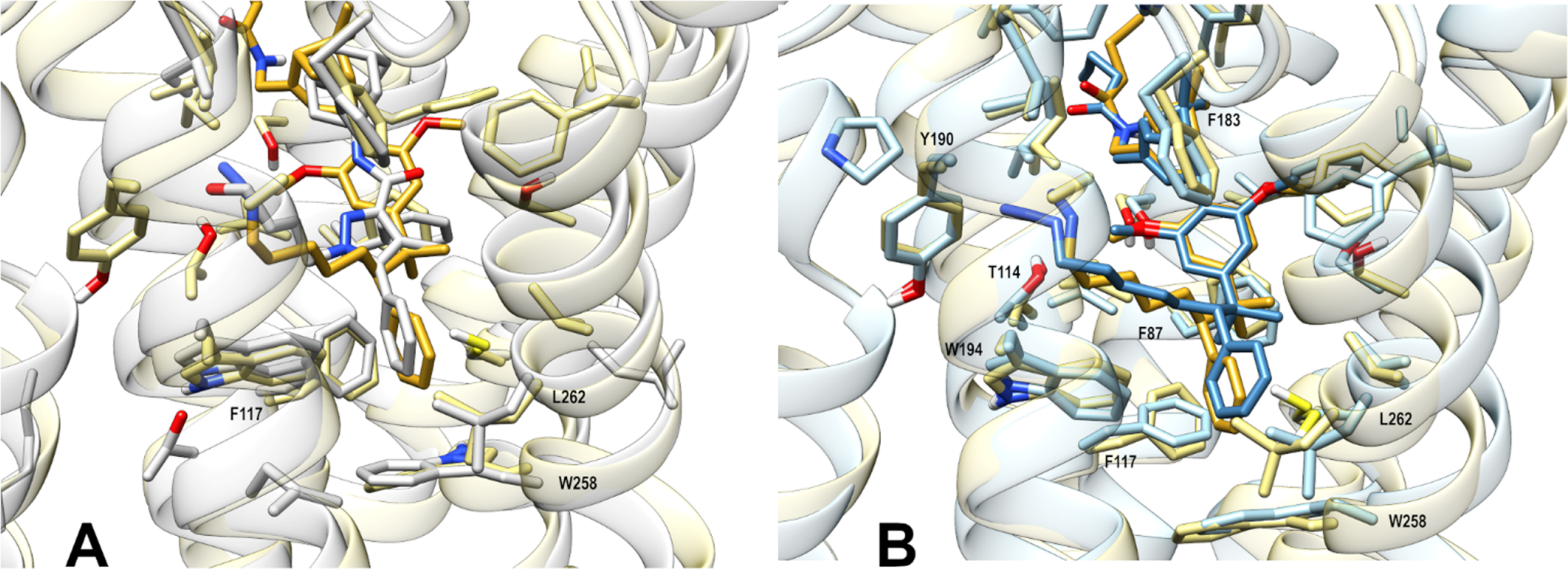
Representative frames from molecular dynamics (MD) simulations of CB_2_R (PDB 5ZTY) in complex with (*S*)-**3** or (*R*)-**3**. Superimposition at level of protein backbone of (A) CB_2_R X-ray structure with AM10257 (light gray) and (*R*)-**3** MD complex (ligand in gold and protein in light yellow) and of (B) the two inverse agonist complexes (*S*)-**3** (ligand in blue and protein light blue) and (*R*)-**3** (ligand in gold and protein in light yellow).

All three ligands adopt an L-shape conformation with the pendent alkyl chain hosted in a cleft formed by Phe183^ECL2^, Tyr190^5.39^, Trp194^5.43^, and Thr114^3.33^ (see Figure 9 and Figures S3 and S4). The resorcinol engages in π–π interactions with Phe183^ECL2^, while the pinene core is surrounded by aromatic residues (Phe183^ECL2^, Phe91^2.61^, and Phe94^2.64^). An rmsd plot of **ago-3** following a best fit of protein backbone shows an initial rearrangement followed by a periodic “breathinglike” motion of the resorcinol and alkyl chain that oscillates between bent and flat conformations (see Figures S3 and S5). Furthermore, in accord with our earlier work,^42^ in the CB_2_R–**ago-3** complex the amide group of **ago-3** forms a stable hydrogen bond with the carbonyl of Ser90^2.60^.

In agreement with the in silico docking (Figure 1), the MD simulations suggest the C(2′) phenyl rings of (*S*)-**3** and (*R*)-**3** mirror that of AM10257 and engage in π–π contacts with Phe117^3.36^ and Trp258^6.48^ (see Figure 9A and Figure S6). In contrast to **ago-3**, no initial rearrangement in the binding poses of (*S*)-**3** and (*R*)-**3** was observed in their rmsd plots (see Figure S7). Ligands (*S*)-**3** and (*R*)-**3** share similar binding modes of the pinene-resorcinol core; however, significant differences are observed in the orientation adopted by *gem*-dimethyl groups and the C(2′) phenyl rings (see Figure 9B and Figure S8). In particular, the *gem*-dimethyl group of (*S*)-**3** is rotated clockwise compared to that of (*R*)-**3** and the C(2′) phenyl of (*S*)-**3** is rotated toward Leu262^6.44^, inducing a minor displacement of helix H6, while that of (*R*)-**3** protrudes deeper toward Trp258^6.48^. In both cases, the conformation of the Trp258^6.48^ toggle switch is restricted and CB_2_R is thus stabilized in its inactive state, in stark contrast to the interactions observed with the agonist complex.

Superimposition of CB_2_R protein backbone employing the X-ray structure with AM10257 and the representative MD frames in complex with either (*S*)-**3** (see Figure S6) or (*R*)-**3** (Figure 9A) imply that (*R*)-**3** more closely resembles the binding mode of AM10257 in the crystallized complex. In particular, the secondary binding pocket featuring Trp258^6.48^ and the surrounding residues Phe117^3.36^ and Leu262^6.44^ are in excellent agreement between AM10257 and (*R*)-**3**.

Finally, the difference in free energy of binding (ΔΔ*G*) was determined for (*R*)-**3** and (*S*)-**3** using molecular mechanics/Poisson–Boltzmann (generalized Born) surface area (MM/PB(GB)SA) calculations (see Table S1). The data imply that binding of (*R*)-**3** is more stable by −0.76 kcal mol^–1^ (MM/GBSA) and −0.40 kcal mol^–1^ (MM/PBSA) in comparison to (*S*)-**3**. Notably, the calculated ΔΔ*G* values are in agreement with the experimental difference of ΔΔ*G* = −0.9 kcal mol^–1^ for epimers (*S*)-**3** and (*R*)-**3**.

## Conclusion

This study describes the in silico guided, structure-based switch of functionality from agonist to inverse agonist of HU-308, a ligand extensively applied to unravel CB_2_R pharmacology and currently investigated in clinical trials. The novel inverse agonist platform ligands (*S*)-**1** and (*R*)-**1** demonstrated high binding affinity for CB_2_R and selectivity against CB_1_R that was retained upon functionalization with a range of chemically distinct substituents and fluorophores. The functional response exerted on CB_2_R by (*S*)-**1**, (*R*)-**1**, and their derivatives was evaluated by HTRF and BRET and implied an inverse agonist profile in cAMP as well as G protein recruitment assays, and no induction of β-arrestin–receptor association. Live cell experiments with (*R*)-**2** and (*S*)-**3**/(*R*)-**3** demonstrated that the probes do not activate CB_2_R toward ERK_1/2_ phosphorylation and Ca^2+^ signaling pathways, respectively. Fluorescence microscopy experiments with (*R*)-**7** and (*R*)-**9** in AtT-20 cells expressing human and rat CB_2_R isoforms demonstrated excellent target specificity and species translatability. Treatment of the BV-2 microglial cell line with (*R*)-**7** and (*R*)-**9** allowed imaging of endogenous CB_2_R in live cells. Finally, MD simulations with (*S*)-**3**, (*R*)-**3**, and **ago-3** corroborate the critical role of the C(2′) phenyl substituent in conferring the functional profile by modulating the CB_2_R toggle switch Trp258^6.48^ of the CWxP motif.

More broadly, this work discloses the first ligand platform for CB_2_R that retains its inverse agonist functional profile, affinity, and selectivity independent of its conjugation to a range of diverse functional groups. The probes introduce a long-sought-after complementarity to an agonist-dominated toolkit to study, elucidate and unlock the full therapeutic potential of CB_2_R. Moreover, the platform ligands promise broad application and synergy with previously published work which awaited discovery of an inverse agonist.^26^ The exponential rise in resolved structures of class A GPCRs, many of which are available in inactive, intermediate, and fully active states, has enabled unprecedented insight into the mechanism of receptor activation.^50,84,85^ Thus, the strategy and experimental framework disclosed herein may aid in the structure-based design of agonists, antagonists, and inverse agonists for GPCRs beyond CB_2_R.

## Supporting Information Available

Experimental procedures and characterization data for all compounds (PDF)
