# Supplementary material for: Flipping the GPCR Switch: Structure-Based Development of Selective Cannabinoid Receptor 2 Inverse Agonists
Source: ACS Cent Sci. 2024 Mar 11;10(5):956–68. doi: 10.1021/acscentsci.3c01461 (PMC11117691; doi:10.1021/acscentsci.3c01461)
Supplement: Supplementary file 1 — oc3c01461_si_001.pdf [file oc3c01461_si_001.pdf]

Flipping the GPCR Switch: Structure–Based Development of Selective Cannabinoid Receptor 2 Inverse Agonists

Miroslav Kosar,[a] Roman C. Sarott,[a] David A. Sykes,[b] Alexander E. G. Viray,[c] Rosa Maria Vitale,[d] Nataša Tomašević,[e] Xiaoting Li,[f] Rudolf L. Z. Ganzoni,[a] Bilal Kicin,[a] Lisa Reichert,[a] Kacper J. Patej,[a] Uxía Gómez-Bouzó,[a] Wolfgang Guba,[g] Peter J. McCormick,[h] Tian Hua,[f] Christian W. Gruber,[e] Dmitry B. Veprintsev,[b] James A. Frank,*[c,i] Uwe Grether,*[g] and Erick M. Carreira*[a]

[a]Laboratorium für Organische Chemie, Eidgenössische Technische Hochschule Zürich, Vladimir-Prelog-Weg 3, 8093 Zürich, Switzerland
[b]Faculty of Medicine & Health Sciences, University of Nottingham, Nottingham NG7 2UH, UK; Centre of Membrane Proteins and Receptors (COMPARE), University of Birmingham and University of Nottingham, Midlands, UK
[c]Department of Chemical Physiology & Biochemistry, Oregon Health & Science University, Portland, Oregon 97239-3098, United States
[d]Institute of Biomolecular Chemistry, National Research Council, Via Campi Flegrei 34, 80078 Pozzuoli, Italy
[e]Center for Physiology and Pharmacology, Medical University of Vienna, Schwarzspanierstr. 17, 1090 Vienna, Austria
[f]iHuman Institute, ShanghaiTech University, Shanghai 201210, China
[g]Roche Pharma Research & Early Development, Roche Innovation Center Basel, F. Hoffmann-La Roche Ltd., 4070 Basel, Switzerland
[h]Department of Pharmacology and Therapeutics, University of Liverpool, Ashton street, Liverpool, L69 3GE, UK
[i]Vollum Institute, Oregon Health & Science University, Portland, Oregon 97239-3098, United States

*E-mail: erickm.carreira@org.chem.ethz.ch
*E-mail: uwe.grether@roche.com
*E-mail: frankja@ohsu.edu

SUPPORTING INFORMATION

Table of Contents

SUPPLEMENTARY FIGURES 4

SUPPLEMENTARY TABLES 12

MOLECULAR MODELING 13

MOLECULAR DYNAMICS: COMPUTATIONAL METHODS 13

RADIOLIGAND BINDING ASSAY 15

Cell culture 15

Assay procedure 15

TR-FRET-BASED CB_2R BINDING ASSAYS 16

Cell culture 16

Terbium labeling of SNAP-tagged CB_2R HEK293-TR cells 16

Fluorescent ligand-binding assays 16

Signal detection and data analysis 18

FUNCTIONAL ASSAYS 21

Homogeneous Time-Resolved Fluorescence (HTRF) cAMP assay 21

β–arrestin recruitment assay 21

G_iCASE assay 22

$ERK_{1/2}$ PHOSPHORYLATION ASSAY 23

Cell culture 23

Assay procedure 23

FLUORESCENCE CONFOCAL MICROSCOPY 24

Media and solutions 24

Cell culture 24

Confocal microscopy 24

REFERENCES 26

GENERAL SYNTHETIC METHODS 28

COMPOUND SYNTHESIS AND CHARACTERIZATION 30

NMR SPECTRA 67

SFC TRACES 103

CRYSTALLOGRAPHIC DATA 105

SUPPLEMENTARY FIGURES

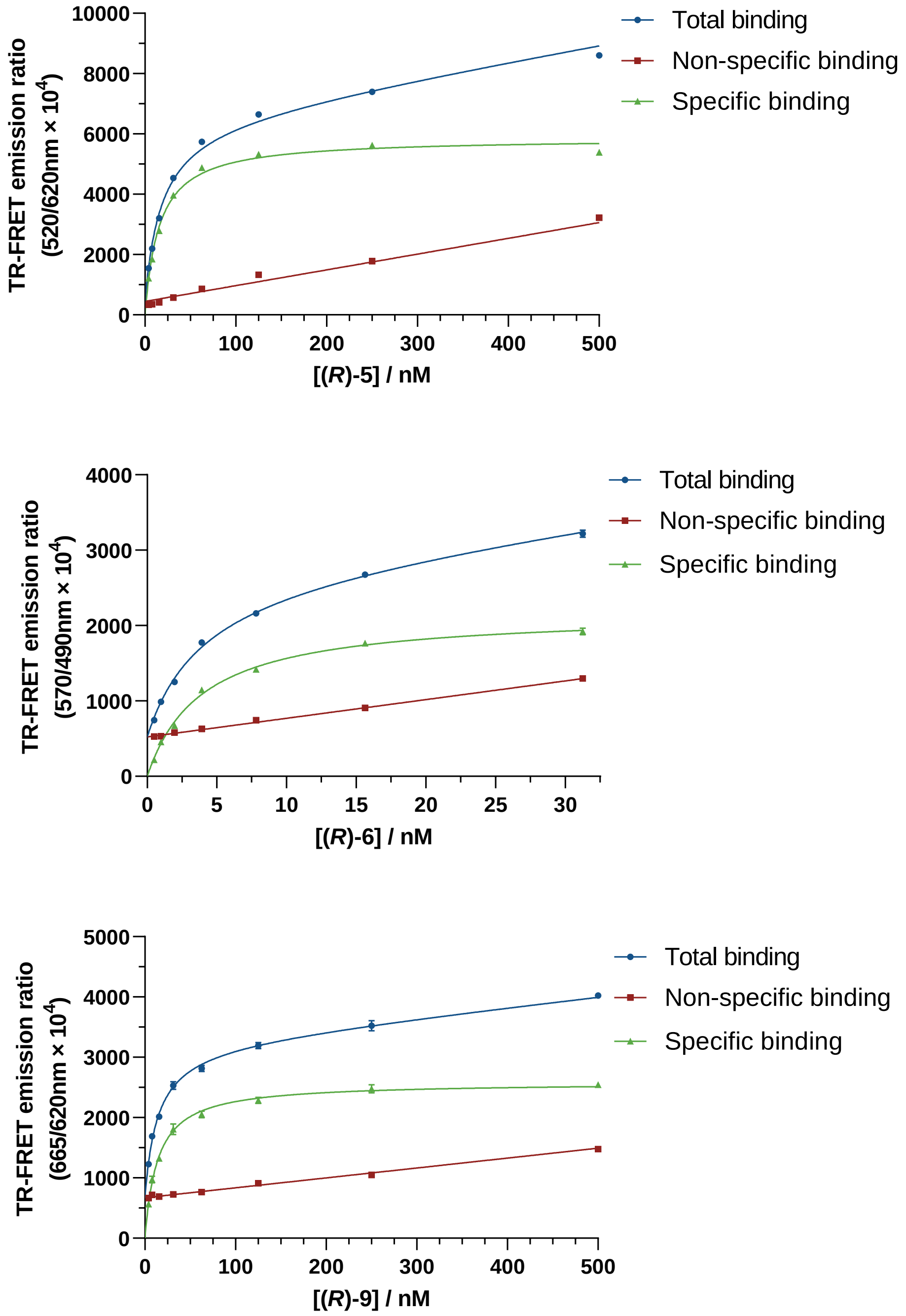

Figure S1. TR-FRET-based saturation binding profile of (*R*)-**5**, (*R*)-**6**, and (*R*)-**9** at hCB$_2$R at 37 °C. Non-specific binding was determined in presence of SR-144,528 (10 µM). Data shown as a mean ± SEM, *N* = 3.

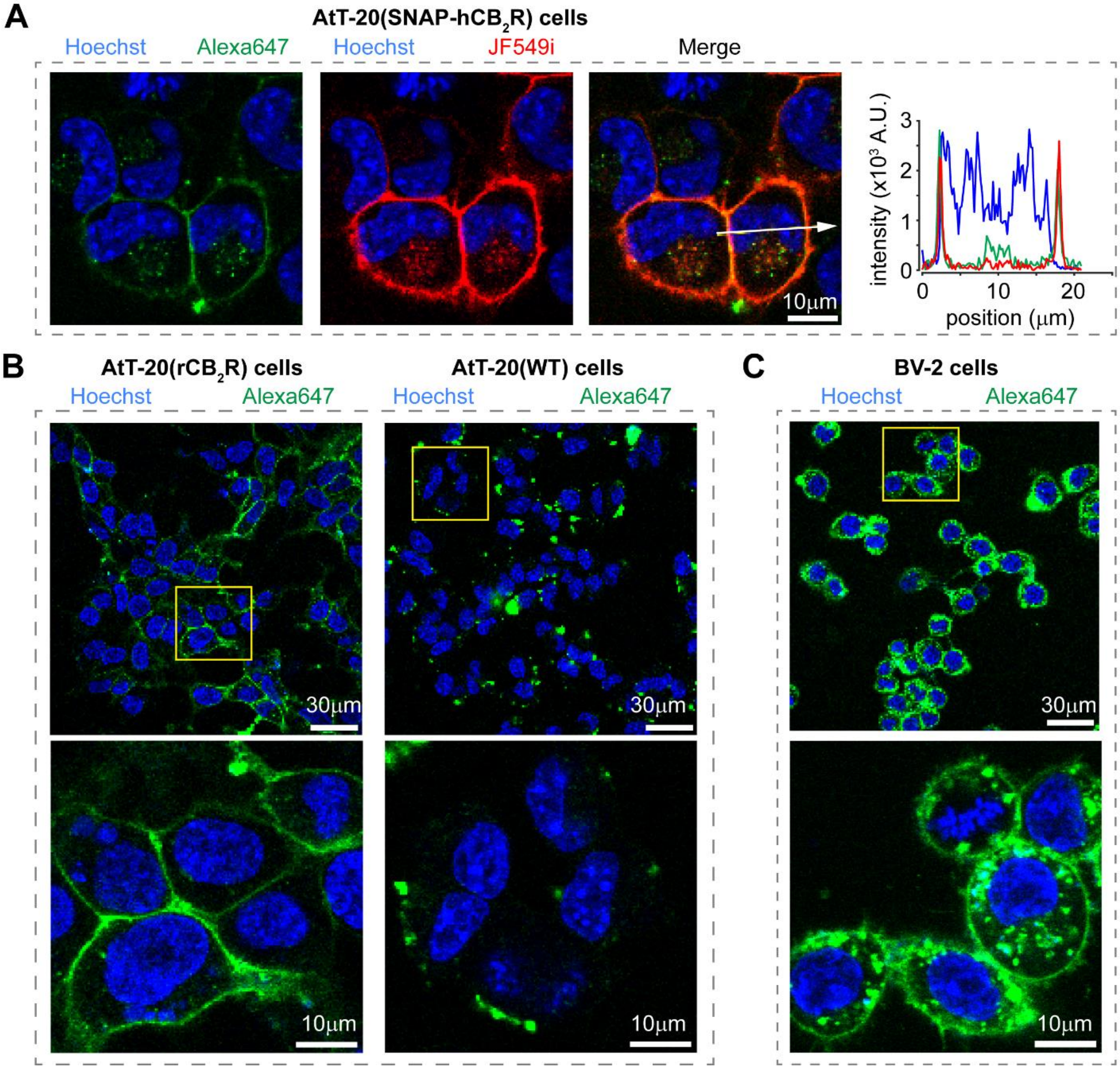

Figure S2. Confocal imaging of (*R*)-**9** in live cell lines; (**A**) AtT-20(SNAP-hCB$_2$R) cells were labelled for 15 min with (*R*)-**9** (Alexa647, 625 nM, green), SNAP-JF549i (JF549i, 500 nM, red), and Hoechst33342 (1 μM, blue) to visualize CB$_2$R, SNAP-tags, and nuclei, respectively. Fluorescence intensity profiles across the white line for Alexa647, JF549i, and Hoechst33342 are shown on the right. (**B**) AtT-20(rCB$_2$R) cells (left) and AtT-20(WT) cells (right) were treated with (*R*)-**9** (625 nM, green) and Hoechst33342 (1 μM, blue) for 15 min and imaged by confocal microscopy. (**C**) Live BV-2 microglial cells that endogenously express mouse CB$_2$R were incubated with (*R*)-**9** (10 μM, green) and Hoechst33342 (1 μM, blue) for 15 min and imaged by confocal microscopy.

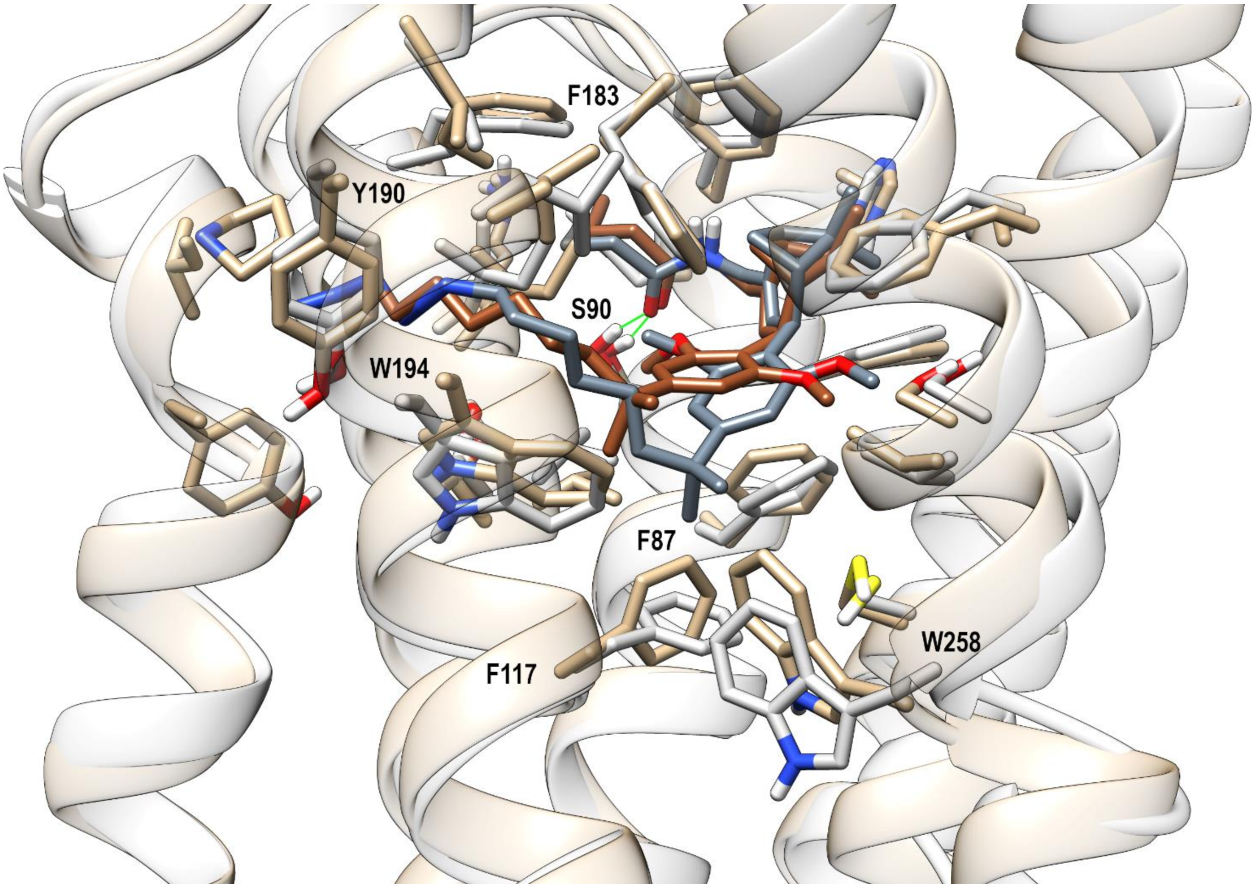

Figure S3 Representative frames from MD of CB_2R–**ago-3** complex for the two conformations (bent–A and flat–B) of **ago-3** discussed in the main text. A stick representation is used for heavy atoms of the ligand (colored in dark gray (A) and sienna (B)) and for protein sidechains within 5 Å of the ligand (colored in light gray (A) and tan (B)). Protein backbone atoms are represented as ribbons colored accordingly to the sidechains, using half-transparency. Hydrogen, nitrogen, oxygen, and sulfur atoms are painted white, blue, red, and yellow, respectively. A green wire representation is adopted for H-bonds.

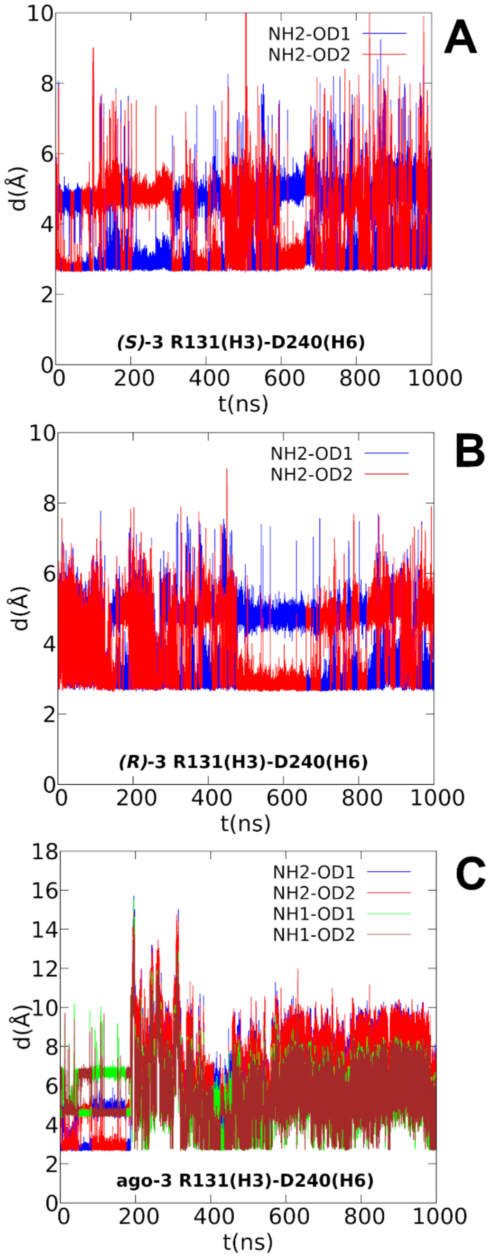

Figure S4. Distance plot between Arg131$^{3.50}$ guanidine nitrogen and Asp240$^{6.30}$ carboxylate oxygen atoms for CB_2R in complex with epimers (*S*)-**3** (panel **A**), (*R*)-**3** (panel **B**) and **ago-3** (panel **C**) over 1 μs of MD. For clarity, only the couple atoms featuring the shortest distance were shown for the epimers. Distance plot was smoothed with a five-point window running average. In both CB_2R–(*S*)-**3** and CB_2R–(*R*)-**3** complexes a stable interaction of the ionic lock Arg131$^{3.50}$ and Asp240$^{6.30}$ has been observed for the whole simulated period (panels **A** and **B**). In comparison, the analysis of MD trajectory of CB_2R in complex with the agonist **ago-3** shows destabilization of the ionic lock (panel **C**).

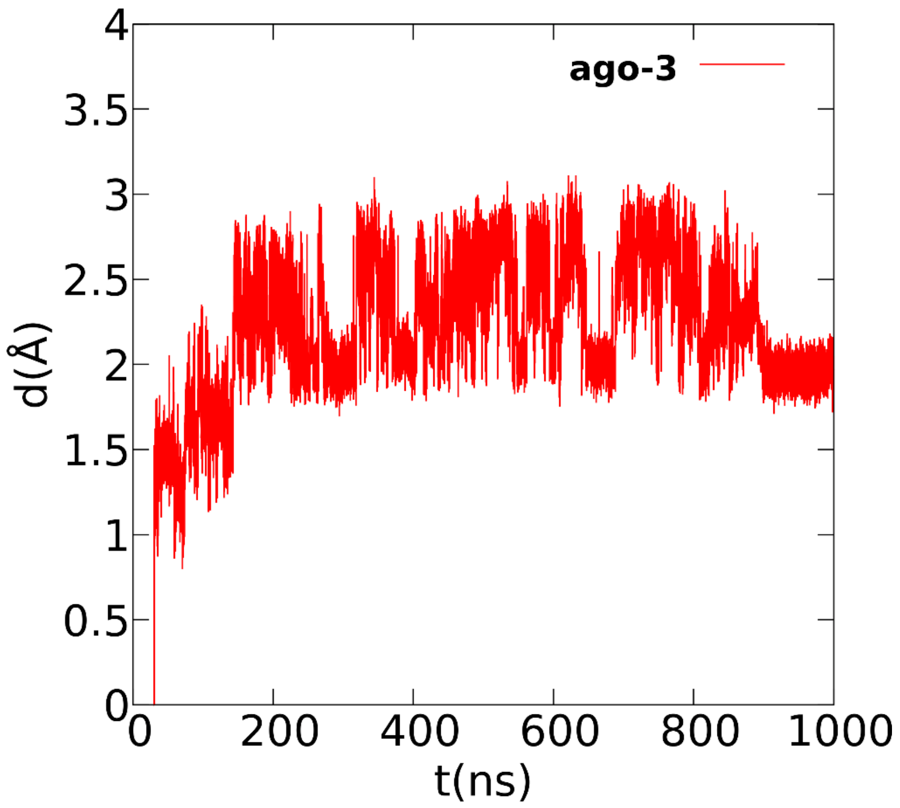

Figure S5. Ligand atom root-mean-square deviation (rmsd) plot calculated for CB_2R–**ago-3** ligand complex, after best fit of the protein backbone atoms (except the flexible *N*-terminus and ICL3 loop) over 1 μs of MD after discarding the first 30 ns of ligand rearrangement. The rmsd plot was smoothed with a five-point window running average. Interestingly, the rmsd plot shows a periodic trend, with the ligand swinging back and forth between two-states.

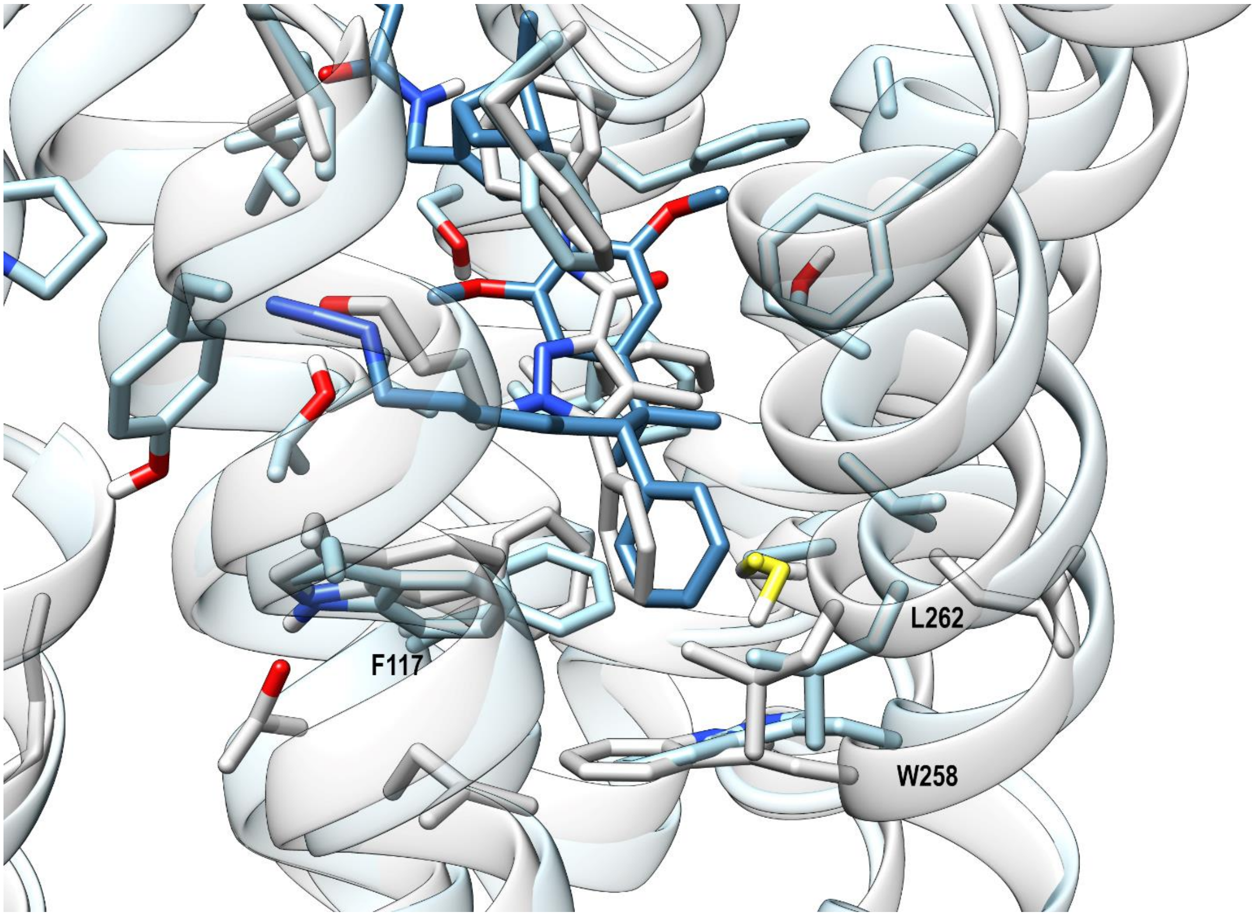

Figure S6. Protein backbone best fit of the representative frames from MD of CB_2R in complex with (*S*)–**3** (colored in blue) overlaid with the CB_2R X–ray structure (PDB:5ZTY, light gray). Protein backbone atoms are represented as ribbons colored accordingly to the sidechains, using half-transparency.

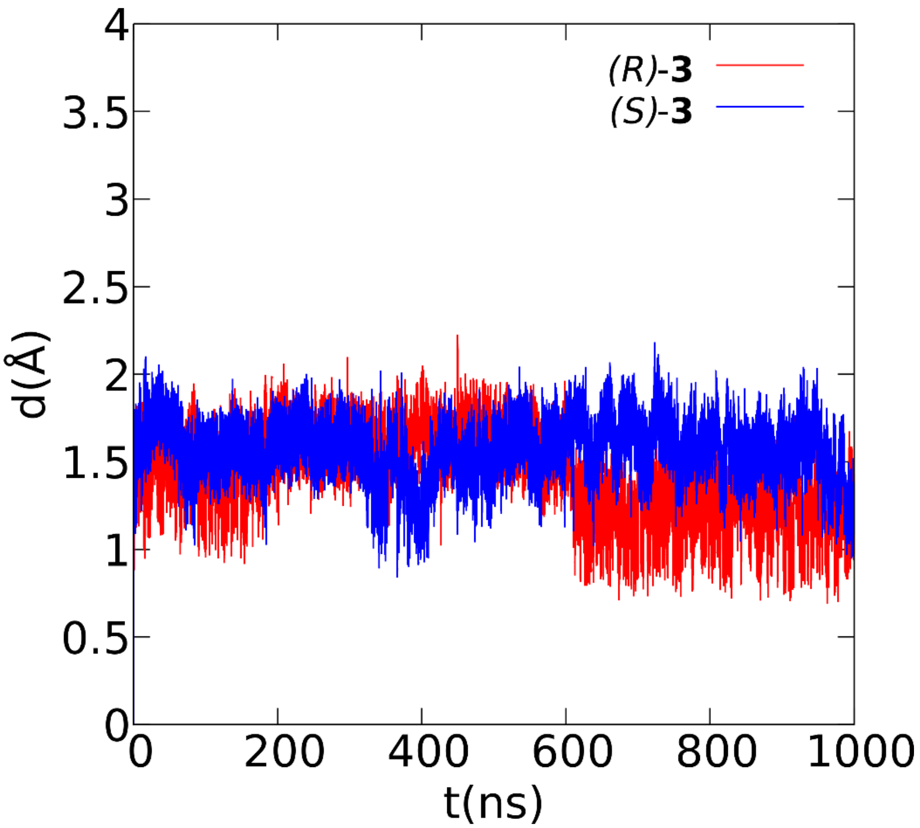

Figure S7. Ligand atom root-mean-square deviation (rmsd) plot for CB_2R in complex with epimers (*S*)–**3** and (*R*)–**3**, after best fitting of the protein backbone atoms (except the flexible *N*-terminus and ICL3 loop) calculated over 1 μs of MD. The rmsd plot was smoothed with a five-point window running average.

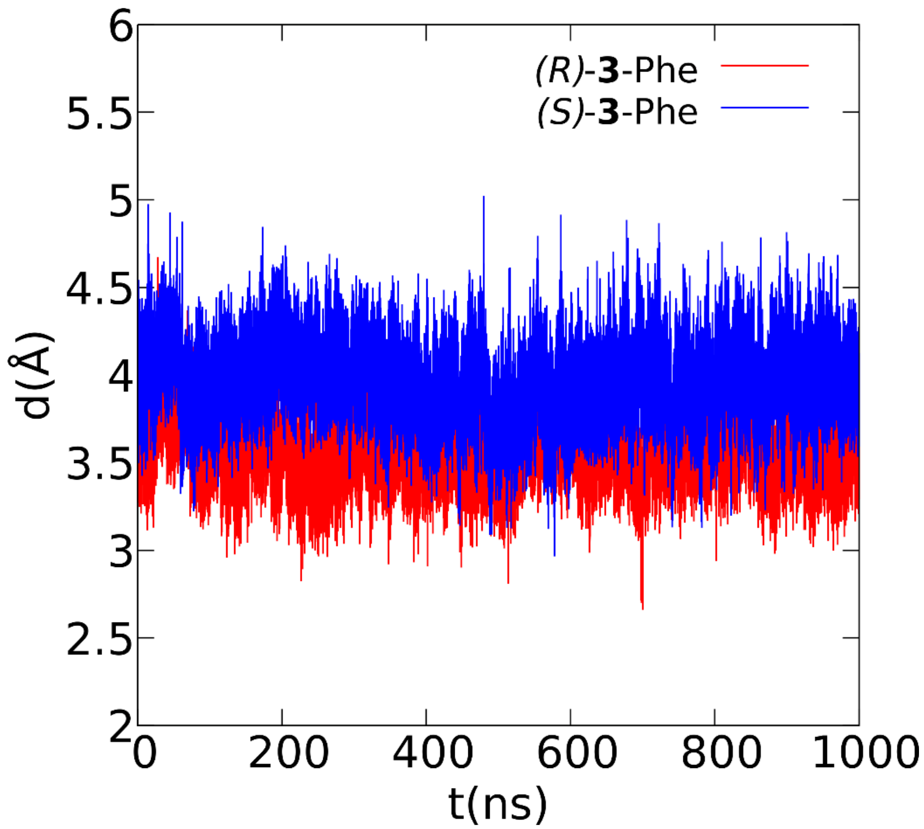

Figure S8. Distance plot for epimers (*S*)–**3** and (*R*)–**3** between the center of mass of their C(2') phenyl ring and the plane formed by Cα atoms of Thr118$^{3.37}$, Ile198$^{5.47}$, Trp258$^{6.48}$, Leu287$^{7.41}$, corresponding to the bottom of the binding pocket, calculated over 1 μs of MD. Distance plot was smoothed with a five-point window running average. The different orientation of the C(2') phenyl ring also determines a shorter distance between its center of mass and the plane formed by Cα atoms of Thr118$^{3.37}$, Ile198$^{5.47}$, Trp258$^{6.48}$, Leu287$^{7.41}$ (corresponding to the bottom of the binding pocket) in the complex of the (*R*)–**3**.

SUPPLEMENTARY TABLES

Table S1. Comparison of free energy of binding (ΔΔG) of CB_2R–ligand bound complexes obtained by molecular mechanics and calculated using experimental K_d values.

**Method**	ΔG (*S*)–**3** / kcal mol^{-1}	ΔG (*R*)–**3** / kcal mol^{-1}	ΔΔG (*R*)–**3** / kcal mol^{-1}
MM/GBSA[#]	–79.10 ± 0.14	–79.86 ± 0.12	–0.76
MM/PBSA[†]	–8.36 ± 0.16	–8.76 ± 0.15	–0.40
Experimental	–12.7	–13.6	–0.90

[#]Data obtained using Molecular Mechanics/Generalized Born Surface Area (MM/PBSA)

[†]Data obtained using Molecular Mechanics/Poisson–Boltzmann Surface Area (MM/PBSA)

MOLECULAR MODELING

The X-ray structure of CB_2R complexed with the antagonist/inverse agonist AM10257 (PDB:5ZTY)[1] was used as a template to dock CB_2R ligands. The docking experiments were performed with the software GOLD[2] (CCDC) with default settings. The best 10 docking poses for each compound were energy-minimized within the binding pocket using MOE (CCG, Montreal)[3] and examined visually to select the most reasonable docking pose with respect to molecular interactions and internal conformational strain. The final selection was based on checking consistency with the available structure-activity relationship information.

MOLECULAR DYNAMICS: COMPUTATIONAL METHODS

Ligands were built with UCSF Chimera 1.17[4] followed by initial energy minimization (EM) at the molecular mechanics level, using AM1-BC charges. The molecules were then fully optimized using the GAMESS program[5] at the Hartree–Fock level with the STO-3G basis set and subjected to HF/6-31G*/STO-3G single-point calculations to derive the partial atomic charges using the RESP procedure.[6] Docking studies were performed with AutoDock 4.2[7] by using CB_2R X-ray structure in complex with an antagonist (PDB: 5ZTY). Both proteins and ligands were processed with AutoDock Tools (ADT) package version 1.5.6rc1[7] to merge non-polar hydrogens and calculate Gasteiger charges. Grids for docking evaluation with a spacing of 0.375 Å and 50 × 50 × 50 points, centered on the ligand binding site, were generated using the program AutoGrid 4.2 included in Autodock 4.2 distribution, following the docking protocol already published.[8] The complexes, selected on the basis of binding energy, cluster population and visual inspection, were completed by addition of all hydrogen atoms, and they underwent energy minimization (EM) and then molecular dynamics (MD) simulations with Amber20 pmemd.cuda module,[9] using the 14SB version of the AMBER force field for the protein, gaff2[10] for the ligand and lipid 14 force field parameter for the lipid. The inactivating mutations present in the PDB entry 5ZTY were back-mutated to wild type residues before MD simulations. The energy-minimized complexes were embedded in a pre-equilibrated palmitoyl-oleoyl-phosphatidyl-choline (POPC) lipid bilayer and solvated in an aqueous medium using the charmmgui web-interface (http://www.charmm-gui.org). Potassium and chloride ions were added to ensure electric neutrality and 0.15 M ionic strength. The system underwent 10,000 steps of energy minimization keeping the solute atoms harmonically restrained to their starting positions (Kr = 10 kcal mol^{-1} $Å^{-1}$). The system was gradually heated to

100 K for 500 ps keeping fixed solute and lipid atoms using a Langevin thermostat, followed by a subsequent heating up to the final temperature of 310 K for 1 ns, keeping fixed the solute and lipid atoms, using an anisotropic Berendsen weak-coupling barostat to equilibrate the pressure. The system then underwent equilibration with positional restraints on the solute: 25 ns with restraints on all solute atoms (Kr = 5 kcal mol^{-1} $Å^{-1}$), followed by 5 ns with restraints on Cα protein atoms alone (Kr = 5 kcal mol^{-1} $Å^{-1}$). The production run was carried out for 1 μs. The ligand cluster analysis was done with the hierarchical agglomerative method after best fit of protein backbone. The experimental binding free energies of the ligands were calculated according to the equation ΔG exp =RT ln(K_d), where T = 310 K, R is the gas constant and K_d are the values reported in the main text of the manuscript. Python scripts ante-MMPBSA.py and MMPBSA.py[11] were used to compute MM/PB(GB)SA, selecting the GBn model (igb=7).[12] 1000 frames were taken evenly from the last 500 ns of MD trajectories. A LCPO algorithm[13] was used to estimate solvent accessible area. The Cpptraj module and the UCSF Chimera 1.17 program were used to perform MD analysis and to draw the figures, respectively.

RADIOLIGAND BINDING ASSAY

Cell culture

For bindings experiments CHO cells stably expressing the human CB_2R were used.[14]

Assay procedure

Radioligand binding assays were performed with [^{3}H]-CP55,940 (Perkin Elmer, Austria). Membrane preparation was carried out according to previously published protocols.[15] Briefly, competition binding experiments were carried out in duplicates at a final volume of 300 µL containing binding buffer (50 mM Tris-HCl, 2.5mM EDTA, 5 mM $MgCl_2$, 0.5 mg/mL fatty acid-free BSA; pH 7.4), [^{3}H]-CP55,940 (0.3 nM final concentration), compounds (semi logarithmically-spaced concentrations ranging from 0.1 nM to 10 µM) and membranes (2 µg/assay) in silanized glass vials. The reaction mixture was incubated for 2 h at 30 °C. Nonspecific binding was determined in the presence of AM630 (10 µM). Bound radioligand was separated from free radioligand by rapid filtration using 0.1% polyethlyenimine-presoaked GF/C glass fiber filter and a Skatron cell harvester. The radioactivity retained on the filters was measured by liquid scintillation. IC_{50} and K_i values obtained from radioligand competition binding assays were determined by fitting the data to a three-parameter logistic Hill equation and applying the Cheng and Prusoff approximation. Data were normalized to specific binding of [^{3}H]-CP55,940 in absence of compounds as maximum percentage (100%), which refers to an average of 4500-5000 fmoles/mg protein for CB_2R. Data are from three independent biological replicates.

TR-FRET-BASED CB_2R BINDING ASSAYS

Cell culture

SNAP-tagged human CB_2R HEK293-TR cells were maintained in a humidified environment at 37 °C and 5% CO_2 in Dulbecco's modified Eagle's medium (DMEM) with 10% fetal bovine serum (FBS) containing blasticidin (5 µg/ml; Invitrogen) and (Zeocin; 20 µg/ml; Invitrogen). For receptor-inducible expression, cells were seeded into t175 cm^2 flasks, grown to 70% confluence and DMEM containing 1 µg/ml tetracycline was added. 48 h later cells were labelled with SNAP-Lumi4-Tb (CisBio) and membranes prepared as described in detail below.

Terbium labeling of SNAP-tagged CB_2R HEK293-TR cells

Cell culture medium was removed from the t175 cm^2 flasks containing confluent adherent CB_2R HEK293-TR cells. Cells were washed 1× in PBS (GIBCO Carlsbad, CA) followed by 1× Tag-lite labeling medium (LABMED, CisBio) to remove the excess cell culture media, then 10 mL of LABMED containing SNAP-Lumi4-Tb (100 nM) was added to the flask and incubated for 1 h at 37 °C under 5% CO_2. Cells were washed 1× in PBS (GIBCO Carlsbad, CA) to remove the excess of SNAP-Lumi4-Tb then detached using 5 mL of GIBCO enzyme-free Hank's-based cell dissociation buffer (GIBCO, Carlsbad, CA) and collected in a vial containing 5 ml of DMEM (Sigma-Aldrich) supplemented with 10% fetal calf serum. Cells were pelleted by centrifugation (5 min at 1500 rpm) and the pellets were frozen to −80 °C. To prepare membranes, homogenization steps were conducted at 4 °C (to avoid receptor degradation). Following homogenization in ice cold buffer 10 mM HEPES containing 10 mM EDTA pH 7.4, an initial centrifugation was performed at 1200 g for 3 min to remove the nuclear fraction. Subsequent centrifugation steps were then performed as described by Herenbrink et al.[16]

Fluorescent ligand-binding assays

All fluorescent ligand binding experiments were conducted in white 384-well Optiplate plates, in assay binding buffer, consisting of Hanks Balanced Salt Solution (HBSS), 5 mM HEPES, 0.5% BSA pH 7.4 at 37 °C, unless noted otherwise. In all cases, non-specific binding was determined by the presence of 10 µM SR-144,528.

Saturation binding TR-FRET assay

To determine equilibrium affinity values, at least six different concentrations of fluorescent ligand were used. Increasing concentrations of fluorescent ligand were incubated with human CB_2R HEK293-TR cell membranes (1 μg per well) in assay binding buffer (final assay volume, 50 μL). The degree of fluorescent ligand bound to the receptor was assessed by TR-FRET detection. Saturation analysis was performed at equilibrium, by simultaneously fitting total and non-specific (NSB) binding data (Eq. 1) and allowed the determination of fluorescent ligand binding affinity.

Kinetic binding TR-FRET assay

To assess the kinetics of fluorescent ligand binding to CB_2R, at least six different concentrations of fluorescent ligand were used. Increasing concentrations of fluorescent ligand were incubated with human CB_2R HEK293-TR cell membranes (1 μg per well) in assay binding buffer (final assay volume, 50 μL). The degree of fluorescent ligand bound to the receptor was assessed by TR-FRET detection. The degree of fluorescent ligand bound to the receptor was assessed at multiple time points by TR-FRET detection to allow construction of association binding curves, which were fitted to (Eq. 2). Non-specific binding was determined as the amount of TR-FRET signal detected in the presence of SR-144,528 (10 μM) and was subtracted from each time point, meaning that $t = 0$ was always equal to zero. Each time point was conducted on the same 384-well plate incubated at room temperature with orbital mixing (1 sec of 100 RPM/cycle).

Competition-kinetic binding TR-FRET assay

To determine the association and dissociation rates of CB_2R-specific ligands, we used a competition-association binding assay. This approach involves the simultaneous addition of both fluorescent ligand and competitor to the CB_2R preparation. Binding experiments were conducted in white 384-well Optiplate plates, in assay binding buffer, either Hanks Balanced Salt Solution (HBSS), 5 mM HEPES, 0.5% BSA, 0.02% pluronic F-127 pH 7.4, and 100 μM GppNHp.

To achieve this aim HEK293 cell membranes containing the human CB_2R (1 μg per well) were added to wells containing increasing concentrations of unlabelled ligands, and a fixed concentration of the fluorescent ligand RO7568997 (900 nM), a concentration approximately 2 × its K_d in 50 μL of assay buffer in a 384-well plate incubated at 37 ^oC with orbital mixing. The

degree of fluorescent ligand bound to the receptor was assessed at multiple time points by HTRF detection.

The kinetic parameters of RO7568997 plus those of unlabeled compounds were determined using a start time of ~60 sec and an interval time of 60 sec. Nonspecific binding was determined as the amount of HTRF signal detected in the presence of (SR144,528, 10 μM) and was subtracted from total binding, to calculate specific binding, meaning that t = 0 was always equal to zero. The association and dissociation rates of RO7568997 was fixed at 1.13 min^{-1} and $3.5x10^6$ M^{-1} min^{-1} and determined by fitting association binding data to Eq. 2.

Competition association binding data were globally fitted using (Eq. 3) to simultaneously calculate k_{on} and k_{off} of unlabeled compound.

Signal detection and data analysis

Signal detection was performed on a Pherastar FSX (BMG Labtech, Offenburg, Germany). The terbium donor was always excited with four laser flashes at a wavelength of 337 nm. TR-FRET signals were collected at both 570 nm (acceptor) and 490 nm (donor), when using the DY-480XL and TAMRA-based fluorescent ligands (e.g. (*R*)-**2** and (*R*)-**6**) and at 520 nm (acceptor) and 620 nm (donor), when using the green NBD, fluorescein, and Alexa488 fluorescent ligands (e.g. (*R*)-**4**, (*R*)-**5** and (*R*)-**7**), and at 665 nm (acceptor) and 620 nm (donor surrogate), when using the red Alexa647 based fluorescent ligand ((*R*)-**9**). TR-FRET ratios were obtained by dividing the acceptor signal by the donor signal and multiplying this value by 10,000.

Saturation binding data were analysed using GraphPad Prism 10.0 by non-linear regression according to a one-site equation by globally fitting total and NSB. Individual estimates for the fluorescent ligand dissociation constant (K_d) were calculated using the following equations where L is the fluorescent ligand concentration:

$$\text{Total binding} = \text{Specific}+\text{NSB}=(\text{Bmax}*[\text{L}])/(\text{Kd}+[\text{L}])+\text{slope}*[\text{L}]+\text{Background}$$

$$\text{NSB}=\text{slope}*[\text{L}]+\text{Background} \qquad \text{(Eq. 1)}$$

Fitting the total and NSB data sets globally (simultaneously), sharing the value of slope, provides one best-fit value for both the K_d and the B_{max}.

Fluorescent ligand association data were fitted as follows to a global fitting model using GraphPad Prism 10.0 to simultaneously calculate k_{on} and k_{off} using the following equation,

$$k_{ob} = [L]*k_{on} + k_{off} \quad \text{(Eq. 2)}$$

$$Y = Y_{max}*(1-\exp(-1*k_{ob}*X))$$

Where, k_{ob} equals the observed rate of ligand association and k_{on} and k_{off} are the association and dissociation-rate constants respectively of the fluorescent ligand. In this globally fitted model of tracer binding, tracer concentrations [L] are fixed, k_{on} and k_{off} are shared parameters whilst k_{obs} is allowed to vary. Here, Y is the level of receptor-bound tracer, Y_{max} is the level of tracer binding at equilibrium, X is in units of time (eg. min) and k_{obs} is the rate in which equilibrium is approached (eg. min^{-1}).

Association and dissociation rates for unlabeled antagonists were calculated using the following equations first described by Motulsky and Mahan[17] and modified by Schiele[18] to account for photobleaching.

$$K_A = k_1[L] + k_2$$

$$K_B = k_3[I] + k_4$$

$$S = \sqrt{((K_A - K_A)^2 + 4 \cdot k_1 \cdot k_3 \cdot L \cdot I \cdot 10^{-18})}$$

$$K_F = 0.5 * (K_A + K_B + S)$$

$$K_S = 0.5 * (K_A + K_B - S)$$

$$Q = \frac{B_{max} * K_1 * L * 10^{-9}}{K_F - K_S}$$

$$Mot = Q \cdot \left(\frac{k_4 \cdot (K_F - K_S)}{K_F \cdot K_S} + \frac{k_4 - K_F}{K_F} exp^{(-K_F \cdot X)} - \frac{k_4 \quad K_S}{K_S} exp^{(-K_S \cdot X)} \right)$$

$$Bleaching = B_{max} * exp^{(-K_{drift} * X)}$$

$$Y = Mot * Bleaching \quad \text{(Eq. 3)}$$

Where: X = Time (min), Y = Specific binding (e.g. CPM or HTRF units e.g. HTRF ratio 520nm/620nm x 10'000), k_1 = k_{on} tracer (M^{-1} min^{-1}), k_2 = k_{off} tracer (min^{-1}), L = Concentration of

tracer used (nM), I = Concentration unlabeled ligand (nM). Fixing the above parameters allows the following to be calculated: k_3 = Association-rate constant of unlabeled ligand (M^{-1} min^{-1}), k_4 = Dissociation-rate constant of unlabeled ligand (min^{-1}), B_{max} = Maximal specific binding of the system at equilibrium binding (e.g. CPM or HTRF units, e.g. HTRF ratio 520nm/620nm x 10’000), K_{drift} = Signal drift.

FUNCTIONAL ASSAYS

Homogeneous Time-Resolved Fluorescence (HTRF) cAMP assay

The homogeneous time-resolved fluorescence (HTRF) cAMP assay was conducted according to the manufacturer's protocol for the cAMP-Gs Dynamic kit (Cisbio). Briefly, the CHO cell line stably overexpressing CB_2R was maintained in Ham's F12 supplemented with 10% FBS, penicillin (100 U/mL, Gibco-ThermoFisher), streptomycin (100 µg/mL, Gibco-ThermoFisher) and G418 (400 µg/mL, ThermoFisher). For CB_2R compounds test, the cells were harvested with Cell Dissociation Buffer, resuspended in F-12 K at 0.4×10^6 cells/mL, and dispensed into 384-well low volume plates at 2000 cells/5 µL per well. The cells were stimulated with compounds diluted in Stimulation Buffer (2.5 µL/well) for 15 min at room temperature, followed by the addition of forsklin (2.5 µL 25 µM). After 15 mins incubation, reactions were stopped by 5 µL/well cAMP-d2 conjugate working solution followed by 5 µL/well of anti-cAMP cryptate working solution. After incubation for 1 h at room temperature, the plates were read in a PerkinElmer Envision reader for Time-Resolved Förster Resonance Energy Transfer (TR-FRET) at 620 nm and 665 nm.

β–arrestin recruitment assay

SNAP-tagged human CB_2R HEK293-TR cells were maintained in a humidified environment at 37 °C and 5% CO_2 in Dulbecco's modified Eagle's medium (DMEM) with 10% fetal bovine serum (FBS) containing blasticidin (5 µg/mL; Invitrogen) and (Zeocin; 20 µg/mL; Invitrogen). For receptor-inducible expression, cells were seeded into t175 cm^2 flasks, grown to 70% confluence and DMEM containing 1 µg/ml tetracycline added.

HEK293TR-CB_2R-nLuc cells expressing a fluorescently labelled β-arrestin protein were maintained in a humidified environment at 37 °C and 5% CO_2 in Dulbecco's modified Eagle's medium (DMEM) with 10% fetal bovine serum (FBS) containing blasticidin (5 µg/mL; Invitrogen), Zeocin (20 µg/mL; Invitrogen) and G418 (0.2 mg/mL; Invitrogen) and used to assess compound stimulated β-arrestin recruitment to the human CB_2R. Cultured cells were harvested upon reaching 70% confluency and plated at a seeding density of 50,000 cells per well. The cells were grown for 48 h until they reached confluency and then stimulated with 1 µg/mL tetracycline for a further 48 h to induce CB_2R expression. Cell culture media was then aspirated from the wells and the cells were washed with assay buffer, 100 µL/well HBSS containing 0.5% BSA and 5 mM

HEPES. Following the wash, assay buffer containing 10 µM furimazine, 90 µL/well was added to the wells. The plate was then incubated at 37 °C for 15 minutes to allow the nanoLuc substrate furimazine to enter the cells. Three BRET cycles of 1-minute intervals were performed to assess basal BRET levels, following which 10 µL of each compound, diluted in assay buffer was added to the assay plate, which was read at 1-minute intervals for 30 minutes.

Compounds included serial dilutions of the synthetic CB_2R agonists HU-210, HU-308 and the novel inverse agonists. Buffer containing 10% DMSO (1% final) served as the vehicle control and HU-210 (100 nM) was the positive control to which all responses were normalised.

G_iCASE assay

The assay buffer used to profile compounds in the CB_2R G_i-CASE system consisted of HBSS containing 0.02% pluronic F127, 0.5% BSA and 5 mM HEPES. The reference compounds HU-210, SR-144,528 and the test compounds were initially serially diluted in DMSO in a 96-well polypropylene plate. 0.5 µL of the reference and test compound serial dilutions was then added to a white 384 well Optiplate^{TM} (PerkinElmer) containing 39.5 µL of assay buffer. Finally, the CB_2R membranes containing the G_i-CASE biosensor were thawed and added into assay buffer containing 50 µM furimazine. Membranes (10 µL) were added to the assay plate at a final concentration of 5 µg/well. The total assay volume was 50 µL. The assay plates were read using a BMG Pherastar FSX reader at 28°C. The duration of data collection on the PHERAstar FSX using the BRET1 plus module (535-30LP/475-30BP) was 60 minutes at 1-minute intervals (60 cycles) in the case of membrane-based experiments.

$ERK_{1/2}$ PHOSPHORYLATION ASSAY

Cell culture

For ERK phosphorylation assay an inducible HCC1954 breast cancer cell line expressing CB_2R was used.

Assay procedure

$ERK_{1/2}$ phosphorylation was measured using AlphaScreen® SureFire® p-$ERK_{1/2}$ assay kit according to manufacturer instructions (PerkinElmer)**.** Briefly, HCC1954 cells were seeded at a density of 100 000 cells/well in poly-D-lysine coated 96-well plates and incubated in absence or presence of 1 µg/mL doxycycline for 24 h. On the next day prior to assay, cells were serum-starved for 2 h at 37°C. Compounds (1 µM final concentration) were prepared in serum-free medium, added to cells, and incubated for 30 min at room temperature. Following the incubation, cells were washed twice with PBS before the addition of lysis buffer and lysed on a plate shaker (~350 rpm) for 10 min at room temperature. Lysates were transferred into white ProxiPlate 384-well microplates and assay beads were prepared and added according to the kit protocol. The plate was incubated for 2 h and $ERK_{1/2}$ phosphorylation was measured on CLARIOstar® Plus Multimode Plate Reader (BMG Labtech, Germany), using a ratio of 620/570 nm. For ERK phosphorylation assay, data were normalized to pERK levels of vehicle-treated control (0.1% DMSO, 100%). Statistical significance was determined by one-way ANOVA followed by Tukey's post hoc test. Data are from three independent biological replicates; p-value is displayed as **$p < 0.01$; ***$p < 0.001$.

FLUORESCENCE CONFOCAL MICROSCOPY

Media and solutions

Culture media (D10): Contains high glucose DMEM (450 mL, Gibco, #11965-092), FBS (50 mL, HyClone's USDA tested, #SH30910), antibiotic Penicillin-strep (2.5 mL, Gibco, #15070). Sterile filtered and aliquoted.

Culture media (D10) with hygromycin-B selection: Contains high glucose DMEM (450 mL, Gibco, #11965-092), FBS (50 mL, HyClone's USDA tested, #SH30910), antibiotic Penicillin-strep (2.5 mL, Gibco, #15070). Sterile filtered and aliquoted. Selection maintained with Hygromycin-B (200 µg/mL, Thermo Fisher Scientific, #10687010).

Imaging buffer: Contains (in mM): 115 NaCl, 1.2 $CaCl_2$, 1.2 $MgCl_2$, 1.2 K_2HPO_4, 20 HEPES, 20 D-glucose. Adjusted to pH 7.4 with NaOH. Sterile filtered and aliquoted.

Cell culture

AtT-20 wild type (WT) and overexpressing rat CB_2R cells (AtT-20(rCB_2R)) were cultured in D10 culture media. AtT-20 cells stably expressing SNAP-hCB_2R [AtT-20(SNAP-hCB_2R) cells] were prepared as previously described[19] and cultured in D10 media with hygromycin-B at 37 °C and 5% CO_2. In preparation for microscopy experiments, 8-well Ibidi glass bottom chambered coverslips (Ibidi, #0827-90) were coated with poly-L-lysine (25 µL per well, Sigma-Aldrich, #P4832) for 20 min at 37 °C and 5% CO_2, followed by two washes with PBS. Cells were detached from their culture flask using trypsin (Gibco, #25300) and plated on the chambered coverslips to achieve 70-80% confluence 24-48 h later for imaging experiments in 37 °C and 5% CO_2.

Confocal microscopy

Ca^{2+} imaging experiments:

AtT-20(rCB_2R, WT, and SNAP-hCB_2R) cells in 8-well glass bottom chambered coverslips were incubated in imaging buffer containing Fluo-4-AM (2 µM, ThermoFisher, #F14201) for 45 min at 37 °C and 5% CO_2. Cells were then washed twice with standard imaging buffer (250 µL each) and remained in imaging buffer for the duration of the experiment (250 µL). Time-lapse images were acquired with an Olympus Fluoview 1200 laser scanning confocal microscope using a 20×/0.75 objective (Olympus UPlanSApo). Fluo-4 excitation was achieved using a 488 nm laser setting at

low laser power (<2%). Time-lapse movies were acquired with a frame-time of 4 s per frame. Compounds were first dissolved at 1000x their final concentration in DMSO and incubated for 10 s with 10% Pluronic F-127 in DMSO (1:1 v:v ratio with compound solution, Tocris #6253). The compound mixture was then diluted 1000x into the imaging well.

Localization experiments with (*R*)-7 and (*R*)-9:

Fluorescence images were acquired with an Olympus Fluoview 1200 laser scanning confocal microscope using a 63× objective. AtT-20 (SNAP-hCB_2R, rCB_2R, and wild type) cells in 8-well glass bottom chambered coverslips were incubated in D10 media containing (*R*)-**7** or (*R*)-**9** (625 nM), Janelia Fluor SNAP 549i (500 nM), and Hoechst-33342 (1 µM) for 15 min. BV-2 cells were plated in 8-well glass bottom chambered coverslips were incubated in D10 media containing (*R*)-**7** or (*R*)-**9** (2.5 µM and 10 µM, respectively), Janelia Fluor SNAP 549i (500 nM), and Hoechst-33342 (1 µM) for 15 min. The cells were then washed twice with imaging buffer (each 250 µL) and placed on the microscope stage for equilibration before imaging. For a direct comparison of the AtT-20(rCB_2R) and wild type cells, one well of each genotype was labelled in parallel, and the identical settings on the confocal microscope were used between wells to accurately compare the dye fluorescence intensities.

REFERENCES

1. Li, X.; Hua, T.; Vemuri, K.; Ho, J. H.; Wu, Y.; Wu, L.; Popov, P.; Benchama, O.; Zvonok, N.; Locke, K.; Qu, L.; Han, G. W.; Iyer, M. R.; Cinar, R.; Coffey, N. J.; Wang, J.; Wu, M.; Katritch, V.; Zhao, S.; Kunos, G.; Bohn, L. M.; Makriyannis, A.; Stevens, R. C.; Liu, Z. J. Crystal Structure of the Human Cannabinoid Receptor CB2. *Cell* **2019**, *176*, 459-467
2. Jones, G.; Willett, P.; Glen, R. C.; Leach, A. R.; Taylor, R. Development and validation of a genetic algorithm for flexible docking. *J. Mol. Biol.* **1997**, *267*, 727-748
3. ULC, C. C. G. *Molecular Operating Environment (MOE), 2022.02*, 1010 Sherbrooke St. West, Suite #910, Montreal, QC, Canada, 2022.
4. Pettersen, E. F.; Goddard, T. D.; Huang, C. C.; Couch, G. S.; Greenblatt, D. M.; Meng, E. C.; Ferrin, T. E. UCSF Chimera - a visualization system for exploratory research and analysis. *J. Comput. Chem.* **2004**, *25*, 1605-1612
5. Michael W. Schmidt, K. K. B., Jerry A. Boatz, Steven T. Elbert, Mark S. Gordon, Jan H. Jensen, Shiro Koseki, Nikita Matsunaga, Kiet A. Nguyen, Shyjun SU, Theresa L. Windus, Michel Dupuis and John A. Montgomery General Atomic and Molecular Electronic Structure System. *J. Comput. Chem.* **1993**, *14*, 1347-1363
6. Kollman, T. F. a. P. A. Application of the RESP Methodology in the Parametrization of Organic Solvents. *J. Phys. Chem. B* **1998**, *102*, 8070-8079
7. Morris, G. M.; Huey, R.; Lindstrom, W.; Sanner, M. F.; Belew, R. K.; Goodsell, D. S.; Olson, A. J. AutoDock4 and AutoDockTools4: Automated docking with selective receptor flexibility. *J. Comput. Chem.* **2009**, *30*, 2785-2791
8. Vitale, R. M.; Thellung, S.; Tinto, F.; Solari, A.; Gatti, M.; Nuzzo, G.; Ioannou, E.; Roussis, V.; Ciavatta, M. L.; Manzo, E.; Florio, T.; Amodeo, P. Identification of the hydantoin alkaloids parazoanthines as novel CXCR4 antagonists by computational and in vitro functional characterization. *Bioorg. Chem.* **2020**, *105*, 104337-104347
9. Case, D. A.; Aktulga, H. M.; Belfon, K.; Ben-Shalom, I. Y.; Berryman, J. T.; Brozell, S. R.; Cerutti, D. S.; Cheatham, I., T.E.; Cisneros, G. A.; Cruzeiro, V. W. D.; Darden, T. A.; Duke, R. E.; Giambasu, G.; Gilson, M. K.; Gohlke, H.; Goetz, A. W.; Harris, R.; Izadi, S.; Izmailov, S. A.; Kasavajhala, K.; Kaymak, M. C.; King, E.; Kovalenko, A.; Kurtzman, T.; Lee, T. S.; LeGrand, S.; Li, P.; Lin, C.; Liu, J.; Luchko, T.; Luo, R.; Machado, M.; Man, V.; Manathunga, M.; Merz, K. M.; Miao, Y.; Mikhailovskii, O.; Monard, G.; Nguyen, H.; O'Hearn, K. A.; Onufriev, A.; Pan, F.; Pantano, S.; Qi, R.; Rahnamoun, A.; Roe, D. R.; Roitberg, A.; Sagui, C.; Schott-Verdugo, S.; Shajan, A.; Shen, J.; Simmerling, C. L.; Skrynnikov, N. R.; Smith, J.; Swails, J.; Walker, R. C.; Wang, J.; Wang, J.; Wei, H.; Wolf, R. M.; Wu, X.; Xiong, Y.; Xue, Y.; York, D. M.; Zhao, S.; Kollman, P. A. *Amber* **2022**, *University of California, San Francisco*,
10. He, X.; Man, V. H.; Yang, W.; Lee, T. S.; Wang, J. A fast and high-quality charge model for the next generation general AMBER force field. *J. Chem. Phys.* **2020**, *153*, 114502-114513
11. Miller, B. R., 3rd; McGee, T. D., Jr.; Swails, J. M.; Homeyer, N.; Gohlke, H.; Roitberg, A. E. MMPBSA.py: An Efficient Program for End-State Free Energy Calculations. *J. Chem. Theory Comput.* **2012**, *8*, 3314-3321
12. Mongan, J.; Simmerling, C.; McCammon, J. A.; Case, D. A.; Onufriev, A. Generalized Born model with a simple, robust molecular volume correction. *J. Chem. Theory Comput.* **2007**, *3*, 156-169

13. Weiser, J.; Shenkin, P. S.; Still, W. C. Approximate atomic surfaces from linear combinations of pairwise overlaps (LCPO). *J. Comput. Chem.* **1999**, *20*, 217-230
14. Gertsch, J.; Leonti, M.; Raduner, S.; Racz, I.; Chen, J. Z.; Xie, X. Q.; Altmann, K. H.; Karsak, M.; Zimmer, A. Beta-caryophyllene is a dietary cannabinoid. *Proc. Natl. Acad. Sci. U. S. A.* **2008**, *105*, 9099-9104
15. Muratspahic, E.; Tomasevic, N.; Koehbach, J.; Duerrauer, L.; Hadzic, S.; Castro, J.; Schober, G.; Sideromenos, S.; Clark, R. J.; Brierley, S. M.; Craik, D. J.; Gruber, C. W. Design of a Stable Cyclic Peptide Analgesic Derived from Sunflower Seeds that Targets the kappa-Opioid Receptor for the Treatment of Chronic Abdominal Pain. *J. Med. Chem.* **2021**, *64*, 9042-9055
16. Klein Herenbrink, C.; Sykes, D. A.; Donthamsetti, P.; Canals, M.; Coudrat, T.; Shonberg, J.; Scammells, P. J.; Capuano, B.; Sexton, P. M.; Charlton, S. J.; Javitch, J. A.; Christopoulos, A.; Lane, J. R. The role of kinetic context in apparent biased agonism at GPCRs. *Nat. Commun.* **2016**, *7*, 10842-10856
17. Motulsky, H. J.; Mahan, L. C. The kinetics of competitive radioligand binding predicted by the law of mass action. *Mol. Pharmacol.* **1984**, *25*, 1-9
18. Schiele, F.; Ayaz, P.; Fernandez-Montalvan, A. A universal homogeneous assay for high-throughput determination of binding kinetics. *Anal. Biochem.* **2015**, *468*, 42-9
19. Kosar, M.; Sykes, D. A.; Viray, A. E. G.; Vitale, R. M.; Sarott, R. C.; Ganzoni, R. L.; Onion, D.; Tobias, J. M.; Leippe, P.; Ullmer, C.; Zirwes, E. A.; Guba, W.; Grether, U.; Frank, J. A.; Veprintsev, D. B.; Carreira, E. M. Platform Reagents Enable Synthesis of Ligand-Directed Covalent Probes: Study of Cannabinoid Receptor 2 in Live Cells. *J. Am. Chem. Soc.* **2023**, *145*, 15094-15108

GENERAL SYNTHETIC METHODS

Procedure

Unless otherwise noted, all reactions were carried out under nitrogen atmosphere.

Chemicals

All chemicals and solvents were purchased from commercial suppliers and were used without further purification. DMF, DMSO, THF, Et_2O, and CH_2Cl_2 were dried using 4 Å molecular sieves or using an LC Technology Solutions solvent purification system (SP-1) under an atmosphere of dry nitrogen. *i*-Pr_2NEt was distilled from KOH under an atmosphere of dry nitrogen.

Chromatography

Analytical thin-layer chromatography (TLC) was performed on Merck silica gel 60 F_{254} TLC glass plates. Purification of reaction products was carried out by flash column chromatography (FCC) using Sigma Aldrich silica 230-400 mesh particle size, 60 Å under 0.3–0.5 bar overpressure or Büchi Pure Chromatography System C-805 Flash with FlashPure silica cartridges.

Nuclear magnetic resonance spectroscopy

NMR spectra were acquired on Bruker AVIII HD 600 MHz, 500 MHz and 400 MHz spectrometers operating at the denoted spectrometer frequency given in MHz for the specified nucleus. ^{1}H NMR spectra are reported with the solvent resonance as the reference ($CDCl_3$ at 7.26 ppm, CD_3OD at 3.31 ppm, CD_2Cl_2 at 5.32 ppm, $(CD_3)_2SO$ at 2.50 ppm). Peaks are reported as (s = singlet, bs = broad singlet, d = doublet, bt = broad triplet, t = triplet, q = quartet, m = multiplet or unresolved, coupling constant(s) in Hz, integral). ^{13}C NMR spectra were recorded with ^{1}H-decoupling and are reported in ppm with the solvent resonance as the reference ($CDCl_3$ at 77.16 ppm, CD_3OD at 49.00 ppm, CD_2Cl_2 at 54.00 ppm, $(CD_3)_2SO$ at 39.52 ppm). Service measurements were performed by the NMR service team of the Laboratorium für Organische Chemie at ETH Zürich.

High-resolution mass spectrometry

High-resolution mass spectrometric data were obtained at ETH Zürich mass spectrometry service on Bruker Daltonics maXis ESI-QTOF, Thermo Q Exactive EI-Trace 1310 Analyser or a Bruker Daltonics maXiS II ESI-QTOF spectrometers and are reported as (*m/z*).

Infrared spectroscopy

Infrared (IR) spectra were measured neat on a Perkin-Elmer UATR Two FT-IR Spectrometer and the band maxima are reported in wavenumbers (cm^{-1}).

Optical rotation

Optical rotations ($[\alpha]_D^T$) were determined using a Jasco P-2000 Polarimeter (10 cm, 1.5 mL cell).

X-ray diffraction

The X-ray diffraction was measured on a XtaLAB Synergy R, HyPix-Arc 150 diffractometer and analyzed by Dr. Nils Trapp and Mr. Michael Solar.

Semi-preparative SFC

The separations were performed using a Prep-SFC-100 semi preparative system with columns (20 × 250 mm, 5 µm) supplied by Daicel, Chiral Technology and 90 mL/min flow of solvent A (CO_2) and co-solvent B, specified by the method below:

Method 1. Column: Chiralcel OD-H, B = 25% (EtOH:*n*-heptane, 1:1)

COMPOUND SYNTHESIS AND CHARACTERIZATION

Synthesis of 12

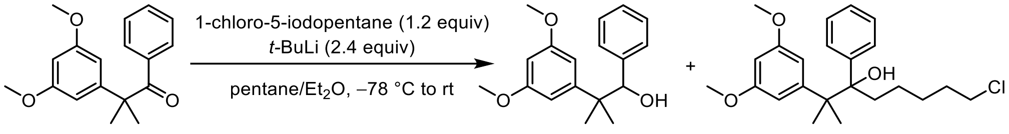

Solution of 1-chloro-5-iodopentane (1.42 mL, 10.1 mmol, 1.2 equiv) in 3:2 pentane–Et_2O (100 mL) was cooled to –78 °C under argon atmosphere and *t*-BuLi (1.7 M in pentane, 11.9 mL, 20.3 mmol, 2.4 equiv) was added dropwise. The solution was stirred at –78 °C for 20 min. To this solution, ketone **10** (2.40 g, 8.44 mmol, 1.0 equiv) in Et_2O (2.0 mL) was added rapidly in one portion. The mixture was stirred for 5 min. at –78 °C, then the cooling bath was removed and the mixture was stirred for additional 30 min. The reaction was quenched by addition of sat. aq. NH_4Cl (50 mL). The layers were separated and the aqueous phase was extracted with Et_2O (3 × 100 mL). Combined organic extracts were washed with brine, dried over $MgSO_4$ and concentrated *in vacuo*. Purification by flash column chromatography (SiO_2; using 5 – 35% Et_2O in hexanes) afforded the reduced alcohol **11** as a white amorphous solid (310 mg, 13%) and product **12** as a colorless viscous liquid (2.75 g, 83%).

11 – side product

^{1}H NMR (400 MHz, $CDCl_3$) δ 7.35 – 7.25 (m, 3H), 7.24 – 7.18 (m, 2H), 6.58 (d, *J* = 2.3 Hz, 2H), 6.40 (t, *J* = 2.2 Hz, 1H), 4.74 (s, 1H), 3.79 (s, 6H), 2.06 (s, 1H), 1.31 (s, 3H), 1.27 (s, 3H). **^{13}C NMR** (101 MHz, $CDCl_3$) δ 160.6, 149.1, 140.8, 127.9, 127.3, 127.3, 105.7, 97.8, 81.8, 55.2, 43.5, 25.9, 22.3. **IR** (neat, ν_{max}/cm^{-1}): 3495, 2936, 2836, 1592, 1453, 1422, 1203, 1152, 701. **HRMS (ESI)**: *m/z* = 309.1456 [M+Na]$^+$ (calc. for $C_{18}H_{22}NaO_3$ *m/z* = 309.1461).

12 – product

^{1}H NMR (400 MHz, $CDCl_3$) δ 7.31 – 7.18 (m, 5H), 6.43 (d, *J* = 2.3 Hz, 2H), 6.36 (t, *J* = 2.2 Hz, 1H), 3.74 (s, 6H), 3.42 (t, *J* = 6.7 Hz, 2H), 2.21 (ddd, *J* = 13.9, 12.0, 4.3 Hz, 1H), 1.81 (s, 1H), 1.70 – 1.53 (m, 3H), 1.37 (s, 3H), 1.36 – 1.28 (m, 2H), 1.24 (s, 3H), 1.22 – 1.09 (m, 1H), 0.95 – 0.78 (m, 1H). **^{13}C NMR** (101 MHz, $CDCl_3$) δ 160.1, 148.2, 142.2, 127.9, 127.1, 126.5, 107.5, 98.0, 80.4, 55.4, 46.7, 45.2, 35.7, 32.7, 27.6, 25.1, 24.8, 23.5. **IR** (neat, ν_{max}/cm^{-1}): 3558, 2937,

2868, 2836, 1594, 1456, 1421, 1386, 1204, 1154, 707. **HRMS (ESI)**: *m/z* = 413.1850 $[M+Na]^+$ (calc. for $C_{23}H_{31}ClNaO_3$ *m/z* = 413.1854).

Synthesis of 13

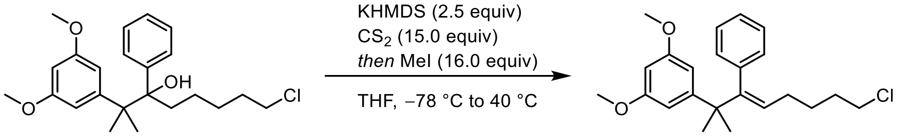

12 13

To a solution of **12** (2.70 g, 6.91 mmol, 1.0 equiv) in anhydrous THF (40 mL) at –78 °C was added KHMDS (1.0 M in THF, 17.3 mL, 17.3 mmol, 2.5 equiv) and CS_2 (6.26 mL, 104 mmol, 15.0 equiv). The yellow solution was stirred at –78 °C for 10 min, the cooling bath was removed and the mixture stirred for another 30 min. MeI (6.88 mL, 111 mmol, 16.0 equiv) was added and the mixture was stirred at rt for 1 h and then at 40 °C overnight. The mixture was diluted with Et_2O (40 mL) and aq. sat. $NaHCO_3$ (40 mL) was added. The layers were separated and the aqueous phase was extracted with Et_2O (2 × 40 mL). Combined organic extracts were washed with brine, dried over $MgSO_4$ and concentrated *in vacuo*. Purification by flash column chromatography (SiO_2; using 0 – 2% Et_2O in hexanes) afforded the product **13** as an amber orange liquid (2.45 g, 95%).

^{1}H NMR (400 MHz, $CDCl_3$) δ 7.17 – 7.11 (m, 3H), 6.70 – 6.63 (m, 2H), 6.50 (d, *J* = 2.3 Hz, 2H), 6.33 (t, *J* = 2.3 Hz, 1H), 5.65 (t, *J* = 7.2 Hz, 1H), 3.78 (s, 6H), 3.42 (t, *J* = 6.8 Hz, 2H), 1.82 – 1.74 (m, 2H), 1.72 – 1.64 (m, 2H), 1.50 – 1.41 (m, 2H), 1.37 (s, 6H). **^{13}C NMR** (101 MHz, $CDCl_3$) δ 160.4, 150.9, 149.5, 140.2, 129.7, 127.5, 126.3, 125.6, 105.8, 97.4, 55.4, 45.0, 44.3, 32.2, 29.0, 28.5, 27.2. **IR** (neat, ν_{max}/cm^{-1}): 2947, 2933, 2835, 1598, 1457, 1422, 1204, 1155, 1059, 706. **HRMS (ESI)**: *m/z* = 395.1745 $[M+Na]^+$ (calc. for $C_{23}H_{29}ClNaO_2$ *m/z* = 395.1748).

Synthesis of SI-1

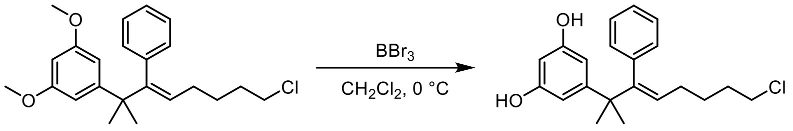

13 SI-1

To a solution of **13** (2.50 g, 6.70 mmol, 1.0 equiv) in anhydrous CH_2Cl_2 (50 mL) at 0 °C was added BBr_3 (1.0 M in CH_2Cl_2, 20.1 mL, 20.1 mmol, 3.0 equiv) dropwise and the green solution was stirred at 0 °C for 5.5 h. The mixture was diluted with CH_2Cl_2 (40 mL) and carefully quenched with aq. sat. $NaHCO_3$ (40 mL). The layers were separated and the aqueous phase was extracted with CH_2Cl_2 (2 × 40 mL). Combined organic extracts were dried over $MgSO_4$ and concentrated *in vacuo*. Purification by flash column chromatography (SiO_2; using 0 – 50% EtOAc in hexanes) afforded the product **SI-1** as a golden oil (2.25 g, 97%).

^{1}H NMR (400 MHz, $CDCl_3$) δ 7.18 – 7.10 (m, 3H), 6.76 – 6.66 (m, 2H), 6.46 (d, *J* = 2.2 Hz, 2H), 6.25 (t, *J* = 2.1 Hz, 1H), 5.90 (bs, 2H), 5.64 (t, *J* = 7.2 Hz, 1H), 3.42 (t, *J* = 6.7 Hz, 2H), 1.82 – 1.71 (m, 2H), 1.71 – 1.61 (m, 2H), 1.51 – 1.38 (m, 2H), 1.33 (s, 6H). **^{13}C NMR** (101 MHz, $CDCl_3$) δ 156.0, 151.8, 149.0, 140.1, 129.6, 127.4, 126.2, 125.7, 107.1, 100.5, 45.2, 43.8, 32.0, 28.8, 28.3, 26.9. **IR** (neat, ν_{max}/cm^{-1}): 3356, 2966, 2944, 2867, 1599, 1440, 1323, 1151, 993, 705. **HRMS (ESI)**: *m/z* = 367.1432 $[M+Na]^+$ (calc. for $C_{21}H_{25}ClNaO_2$ *m/z* = 367.1435).

Synthesis of (*S*)-14 and (*R*)-14

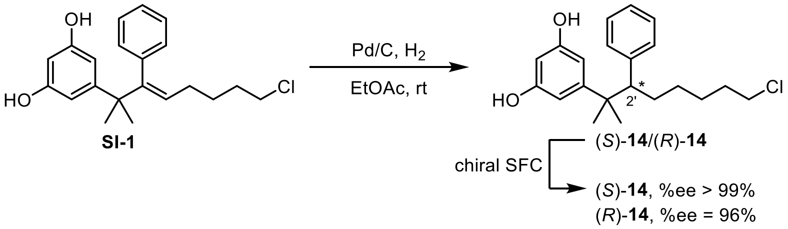

To a solution of **SI-1** (1.80 g, 5.22 mmol) in dry EtOAc (30 mL) was added Pd (10 wt% on C, 5.55 g, 5.22 mmol, 1.0 equiv) and the resulting mixture was hydrogenated with H_2 at 60 bar, rt for 48 h. Then the mixture was filtered over a pad of Celite and the crude material was purified by flash column chromatography (SiO_2, 15 – 30% EtOAc in hexanes) to afford the product (*S*)-**14**/(*R*)-**14** as a dark red oil (1.75 g, 97%). The racemate was resolved by semi-preparative SFC Method 1. to afford enantiomer (*S*)-**14** (450 mg, 25%, %*ee* > 99%) and enantiomer (*R*)-**14** (360 mg, 20%, %*ee* = 96%).

^{1}H NMR (400 MHz, $CDCl_3$) δ 7.30 – 7.17 (m, 3H), 7.11 – 7.03 (m, 2H), 6.42 (d, *J* = 2.2 Hz, 2H), 6.22 (t, *J* = 2.2 Hz, 1H), 5.18 (bs, 2H), 3.38 (td, *J* = 6.7, 2.0 Hz, 2H), 2.74 (dd, *J* = 12.2, 2.9 Hz, 1H), 1.70 – 1.59 (m, 1H), 1.58 – 1.49 (m, 2H), 1.45 – 1.34 (m, 1H), 1.32 – 1.18 (m, 2H), 1.22 (s, 3H), 1.06 (s, 3H), 1.02 – 0.83 (m, 2H). **^{13}C NMR** (101 MHz, $CDCl_3$) δ 156.2, 153.2, 141.5, 130.1, 127.6, 126.3, 106.8, 100.3, 56.8, 45.4, 41.6, 32.4, 29.2, 29.1, 27.5, 26.7, 23.2. **IR** (neat, ν_{max}/cm^{-1}): 3345, 2936, 2867, 1599, 1453, 1320, 1151, 992, 705. **HRMS (ESI)**: *m/z* = 369.1590 $[M+Na]^+$ (calc. for $C_{21}H_{27}ClNaO_2$ *m/z* = 369.1592). (*S*)-**14** **$[\alpha]^{25}_{D}$** = –34.683 ± 0.163 (c = 1.0, $CHCl_3$). (*R*)-**14** **$[\alpha]^{25}_{D}$** = +33.913 ± 0.053 (c = 1.0, $CHCl_3$).

Synthesis of (*S*)-SI-2/(*R*)-SI-2

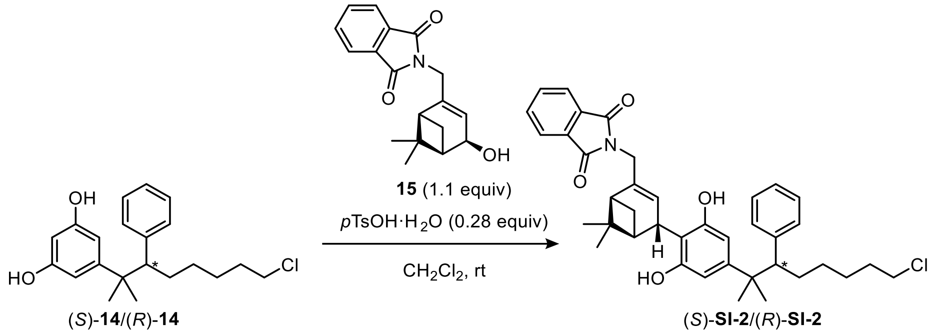

To a solution of resorcinol (*S*)-**14/**(*R*)-**14** (69.3 mg, 200 µmol, 1.0 equiv) and *p*TsOH·H_2O (10.6 mg. 56.0 µmol, 0.28 equiv) in CH_2Cl_2 (10 mL) was added allylic alcohol **15** (65.3 mg, 220 µmol, 1.1 equiv) and the light-yellow solution was stirred at rt for 1.5 h. The reaction was stopped by addition of sat. aq. $NaHCO_3$ (5 mL), the layers were separated and the aqueous layer was extracted with Et_2O (3 × 10 mL). Combined organic extracts were dried over $MgSO_4$ and concentrated *in vacuo*. Purification by flash column chromatography (SiO_2; 0 – 20% EtOAc in hexanes) afforded the product (*S*)-**SI-2/**(*R*)-**SI-2** as a colourless foam (80.0 mg, 64%).

1:1 mixture of diastereomers – all signals reported: **^{1}H NMR** (400 MHz, $CDCl_3$) δ 7.95 – 7.85 (m, 2H), 7.80 – 7.67 (m, 2H), 7.29 – 7.14 (m, 3H), 7.12 – 7.03 (m, 2H), 6.55 – 5.14 (bs, 2H), 6.40 (bs, 2H), 6.02 (s, 1H), 4.47 (ddd, *J* = 15.5, 3.0, 1.9 Hz, 1H), 4.29 – 4.18 (m, 1H), 4.01 (s, 1H), 3.42 – 3.31 (m, 2H), 2.71 (dd, *J* = 12.1, 3.5 Hz, 1H), 2.37 – 2.20 (m, 3H), 1.74 – 1.34 (m, 5H), 1.32 (s, 3H), 1.30 – 1.20 (m, 2H), 1.17 (s, 3H), 1.01 (s, 3H), 1.00 (s, 3H), 0.96 – 0.80 (m, 2H). **^{13}C NMR** (101 MHz, $CDCl_3$) δ 168.5, 154.9, 150.2, 148.2, 141.8, 134.3, 132.1, 130.2, 127.6, 126.3, 123.7, 120.7, 111.9, 107.3, 56.9, 47.4, 45.3, 44.3, 43.3, 41.3, 41.2, 37.9, 32.5, 29.8, 29.2, 28.2, 27.5, 26.8, 26.0, 22.7, 20.7. **IR** (neat, ν_{max}/cm^{-1}): 3460, 2931, 2867, 1771, 1714, 1624, 1574, 1426, 1393, 1341, 1025, 729, 705. **HRMS (ESI)**: *m/z* = 648.2834 $[M+Na]^+$ (calc. for $C_{39}H_{44}ClNNaO_4$ *m/z* = 648.2851). **$[\alpha]^{25}_D$** = +72.790 ± 0.118 (c = 1.0, $CHCl_3$).

Synthesis of (*S*)-16/(*R*)-16

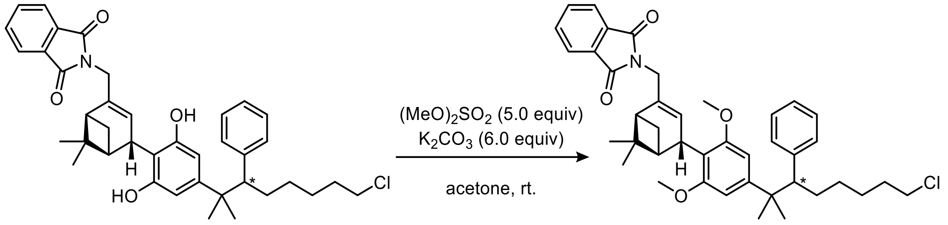

To a suspension of (*S*)-**SI-2**/(*R*)-**SI-2** (30.0 mg, 47.9 µmol, 1.0 equiv) and K_2CO_3 (39.7 mg. 287 µmol, 6.0 equiv) in acetone (0.4 mL) was added $(MeO)_2SO_2$ (23 µL. 240 µmol, 5.0 equiv) and the solution was stirred at rt overnight. The reaction mixture was diluted with Et_2O (5 mL), filtered and concentrated *in vacuo*. Purification by flash column chromatography (SiO_2; 0 – 20% acetone in hexanes) afforded the product (*S*)-**16**/(*R*)-**16** as a pale-yellow oil (24.5 mg, 78%).

1:1 mixture of diastereomers – all signals reported: ^{1}**H NMR** (400 MHz, $CDCl_3$) δ 7.89 – 7.81 (m, 2H), 7.75 – 7.67 (m, 2H), 7.24 – 7.13 (m, 3H), 7.05 – 6.99 (m, 2H), 6.39 (s, 2H), 5.62 – 5.57 (m, 1H), 4.34 – 4.29 (m, 1H), 4.27 – 4.20 (m, 1H), 3.98 – 3.94 (m, 1H), 3.68 (s, 6H), 3.39 – 3.32 (m, 2H), 2.70 (dd, J = 12.1, 2.8 Hz, 1H), 2.21 – 2.12 (m, 2H), 2.05 – 1.98 (m, 1H), 1.72 (d, J = 7.9 Hz, 1H), 1.66 – 1.35 (m, 4H), 1.30 – 1.21 (m, 2H), 1.27 (s, 3H), 1.24 (s, 3H), 1.11 (s, 3H), 0.94 (s, 3H), 0.98 – 0.84 (m, 2H). ^{13}**C NMR** (101 MHz, $CDCl_3$) δ 168.3, 158.3, 148.4, 141.8, 136.1, 133.9, 132.4, 130.2, 127.5, 126.2, 124.0, 123.3, 118.0, 103.6, 57.2, 55.9, 47.6, 45.2, 44.4, 42.6, 41.8, 41.2, 37.5, 32.5, 29.2, 29.0, 27.7, 27.6, 26.8, 26.4, 23.9, 20.9. **IR** (neat, ν_{max}/cm^{-1}): 2932, 2864, 1771, 1714, 1603, 1572, 1465, 1451, 1389, 1239, 1116, 956, 730, 704. **HRMS (ESI)**: *m/z* = 676.3144 $[M+Na]^+$ (calc. for $C_{41}H_{48}ClNNaO_4$ *m/z* = 676.3164). $[\alpha]^{25}_D$ = +57.601 ± 0.343 (c = 0.75, $CHCl_3$).

Synthesis of (*S*)-SI-3/(*R*)-SI-3

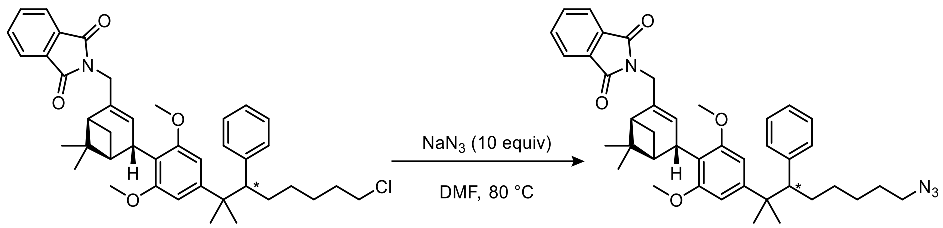

(*S*)-**16**/(*R*)-**16** (*S*)-**SI-3**/(*R*)-**SI-3**

To solution of (*S*)-**16**/(*R*)-**16** (15.2 mg, 23.2 µmol, 1.0 equiv) in DMF (0.10 mL) was added NaN_3 (15.1 mg, 232 µmol, 10 equiv) and the reaction mixture was stirred at 80 °C for 24 h. The mixture was allowed to cool down to rt and diluted with 5% aq. LiCl (5 mL) and Et_2O (5 mL). The layers were separated and the aqueous phase was extracted with Et_2O (4 × 5 mL). Combined organic extracts were dried over $MgSO_4$, filtered and concentrated *in vacuo*. Purification by flash column chromatography (SiO_2, 0 – 10% EtOAc in hexanes) afforded the product (*S*)-**SI-3**/(*R*)-**SI-3** as a pale pink foam (14.7 mg, 96%).

1:1 mixture of diastereomers – all signals reported: **^{1}H NMR** (400 MHz, $CDCl_3$) δ 7.92 – 7.82 (m, 2H), 7.76 – 7.68 (m, 2H), 7.24 – 7.14 (m, 3H), 7.06 – 6.98 (m, 2H), 6.38 (s, 2H), 5.66 – 5.55 (m, 1H), 4.35 – 4.28 (m, 1H), 4.27 – 4.20 (m, 1H), 4.00 – 3.93 (m, 1H), 3.68 (s, 6H), 3.09 (t, *J* = 6.9 Hz, 2H), 2.78 – 2.66 (m, 1H), 2.26 – 2.11 (m, 2H), 2.08 – 1.97 (m, 1H), 1.72 (d, *J* = 7.7 Hz, 1H), 1.68 – 1.54 (m, 1H), 1.50 – 1.29 (m, 3H), 1.27 (s, 3H), 1.26 – 1.23 (m, 3H), 1.23 – 1.13 (m, 2H), 1.12 (s, 3H), 0.94 (s, 3H), 1.02 – 0.82 (m, 2H). **^{13}C NMR** (101 MHz, $CDCl_3$) δ 168.3, 158.3, 148.5, 141.7, 136.2, 133.9, 132.4, 130.1, 127.5, 126.2, 123.9, 123.3, 117.9, 103.6, 57.2, 55.9, 51.5, 47.6, 44.4, 42.6, 41.8, 41.2, 37.5, 29.3, 29.0, 28.7, 27.9, 27.7, 26.7, 26.4, 23.9, 20.9. **IR** (neat, ν_{max}/cm^{-1}): 2931, 2864, 2094, 1772, 1715, 1603, 1572, 1466, 1451, 1389, 1239, 1117, 956, 729, 704. **HRMS (ESI)**: *m/z* = 683.3563 $[M+Na]^+$ (calc. for $C_{41}H_{48}N_4NaO_4$ *m/z* = 683.3568). **$[\alpha]^{25}_D$** = +43.225 ± 0.114 (c = 1.0, $CHCl_3$).

Synthesis of (*S*)-1/(*R*)-1

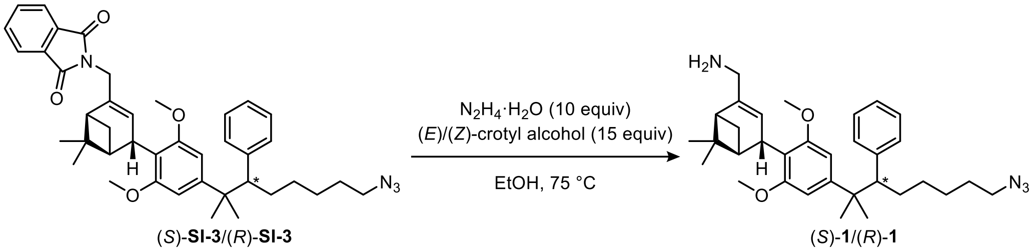

To a solution of (*S*)-**SI-3**/(*R*)-**SI-3** (44.0 mg, 66.5 µmol, 1.0 equiv) in EtOH (1.3 mL) was added $N_2H_4{\cdot}H_2O$ (33 µL, 666 µmol, 10 equiv) and (*E*)/(*Z*)-crotyl alcohol (85 µL, 1.00 mmol, 15 equiv) and the solution was heated to 75 °C and stirred for 3 h. Precipitation of a white solid occurred, and the mixture was filtered over cotton, which was rinsed with EtOH. The filtrate was concentrated *in vacuo*. Purification by flash column chromatography (SiO_2; 1% 7.0 M NH_3 in MeOH, 1 – 4% MeOH in CH_2Cl_2) afforded the product (*S*)-**1**/(*R*)-**1** as a white foam (27.0 mg, 76%).

1:1 mixture of diastereomers – all signals reported: ^{1}**H NMR** (400 MHz, $CDCl_3$) δ = 7.28 – 7.14 (m, 3H), 7.03 (dt, *J* = 7.8, 1.6 Hz, 2H), 6.43 (s, 2H), 5.57 (s, 1H), 4.02 – 3.97 (m, 1H), 3.70 (s, 6H), 3.24 (t, *J* = 1.7 Hz, 2H), 3.11 (td, *J* = 6.9, 1.1 Hz, 2H), 2.72 (dd, *J* = 12.1, 2.9 Hz, 1H), 2.20 (dtd, *J* = 8.3, 5.4, 1.2 Hz, 1H), 2.16 – 2.12 (m, 1H), 2.07 (tq, *J* = 5.5, 1.9 Hz, 1H), 1.71 (dd, *J* = 8.3, 5.9 Hz, 1H), 1.67 – 1.57 (m, 1H), 1.50 – 1.43 (m, 1H), 1.39 (dtd, *J* = 8.8, 6.2, 5.7, 1.3 Hz, 2H), 1.31 (s, 3H), 1.27 (s, 3H), 1.24 – 1.17 (m, 2H), 1.14 (d, *J* = 1.2 Hz, 3H), 1.00 – 0.88 (m, 2H), 0.97 (s, 3H). ^{13}**C NMR** (101 MHz, $CDCl_3$) δ = 158.4, 148.4, 148.4, 143.5, 143.5, 141.7, 130.1, 127.5, 127.5, 126.3, 121.1, 118.5, 118.5, 104.0, 104.0, 57.3, 56.0, 56.0, 51.5, 47.7, 47.6, 44.7, 41.8, 41.0, 37.6, 29.3, 29.3, 28.9, 28.7, 28.1, 27.9, 27.9, 26.7, 26.5, 24.0, 24.0, 21.2. **IR** (neat, ν_{max}/cm^{-1}) 2934, 2865, 2095, 1604, 1572, 1452, 1411, 1240, 1120. **HRMS (ESI)**: *m/z* = 553.3510 $[M+Na]^+$ (calc. for $C_{33}H_{46}N_4NaO_2$ *m/z* = 553.3513). $[\alpha]^{25}_D$ = +71.916 ± 0.163 (c = 0.7, $CHCl_3$).

Synthesis of (*S*)-2/(*R*)-2

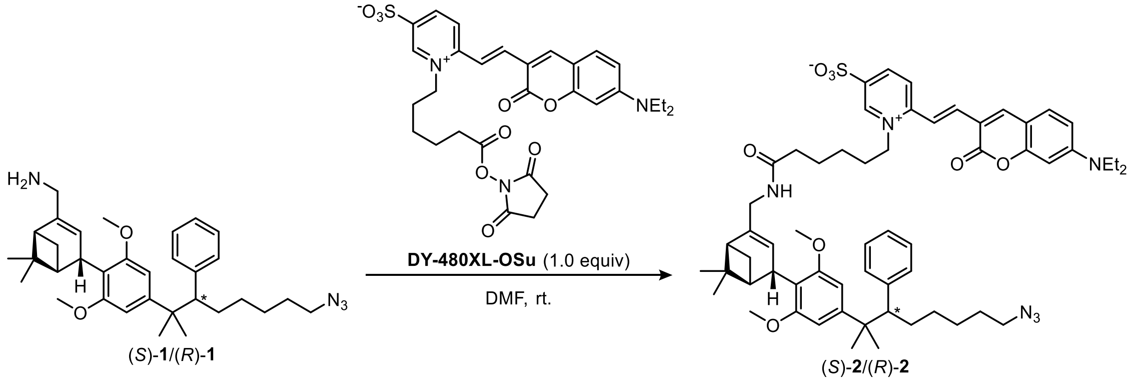

To a solution of (*S*)-**1**/(*R*)-**1** (9.0 mg, 16.9 µmol, 1.0 equiv) in DMF (50 µL) was added **DY-480XL-OSu** (10.4 mg, 16.9 µmol, 1.0 equiv) in DMF (30 µL). The deep red solution was stirred at ambient temperature and under exclusion of light overnight, after which the mixture was concentrated *in vacuo*. Purification by flash column chromatography (superneutral SiO_2, 1 – 5% MeOH in CH_2Cl_2) afforded the product (*S*)-**2**/(*R*)-**2** as dark red waxy solid (15.0 mg, 86%).

1:1 mixture of diastereomers – all signals reported: **^{1}H NMR** (500 MHz, CD_2Cl_2) δ = 9.02 (d, *J* = 1.8 Hz, 1H), 8.62 (dd, *J* = 8.6, 1.7 Hz, 1H), 8.14 (d, *J* = 8.6 Hz, 1H), 8.10 (d, *J* = 15.3 Hz, 1H), 7.94 (s, 1H), 7.51 (d, *J* = 15.2 Hz, 1H), 7.44 (d, *J* = 9.0 Hz, 1H), 7.25 – 7.19 (m, 2H), 7.20 – 7.15 (m, 1H), 7.09 – 7.05 (m, 2H), 6.69 (dd, *J* = 9.0, 2.5 Hz, 1H), 6.50 (d, *J* = 2.4 Hz, 1H), 6.44 (s, 2H), 6.12 (t, *J* = 5.7 Hz, 1H), 5.61 (ddd, *J* = 4.5, 3.0, 1.5 Hz, 1H), 4.64 – 4.56 (m, 2H), 3.94 (q, *J* = 2.1 Hz, 1H), 3.89 – 3.79 (m, 2H), 3.68 (s, 6H), 3.68 (s, 4H), 3.46 (q, *J* = 7.1 Hz, 4H), 3.09 (t, *J* = 6.9 Hz, 2H), 2.76 (dt, *J* = 11.8, 2.3 Hz, 1H), 2.28 (t, *J* = 7.5 Hz, 2H), 2.18 – 2.13 (m, 1H), 2.12 – 2.04 (m, 3H), 2.00 (dtt, *J* = 5.8, 3.9, 1.9 Hz, 1H), 1.76 (q, *J* = 7.5 Hz, 2H), 1.68 (t, *J* = 8.2 Hz, 2H), 1.64 – 1.56 (m, 2H), 1.47 – 1.39 (m, 1H), 1.38 – 1.31 (m, 2H), 1.27 (s, 3H), 1.25 (s, 3H), 1.23 (t, *J* = 7.1 Hz, 6H), 1.20 – 1.12 (m, 2H), 1.09 (s, 3H), 0.97 – 085 (m, 2H), 0.94 (s, 3H). **^{13}C NMR** (126 MHz, CD_2Cl_2) δ = 172.7, 172.6, 172.6, 160.6, 158.7, 157.4, 153.4, 153.1, 149.2, 147.8, 145.0, 143.0, 142.2, 141.3, 139.1, 131.2, 130.5, 127.8, 126.4, 124.8, 123.7, 118.4, 115.9, 113.8, 110.7, 109.4, 104.2, 97.1, 59.2, 57.3, 56.2, 56.2, 51.8, 48.0, 45.6, 44.8, 44.7, 44.5, 42.1, 41.1, 37.9, 36.3, 36.3, 29.8, 29.6, 29.1, 29.0, 28.2, 27.9, 26.9, 26.9, 26.5, 26.2, 25.9, 25.1, 23.9, 21.2, 12.7. **IR** (neat, ν_{max}/cm^{-1}): 2933, 2866, 2095, 1713, 1578, 1504, 1417, 1272, 1134, 1041. **HRMS (ESI)**: *m/z* = 1027.5356 $[M+H]^+$ (calc. for $C_{59}H_{75}N_6O_8S$ *m/z* = 1027.5362).

Synthesis of (*S*)-3/(*R*)-3

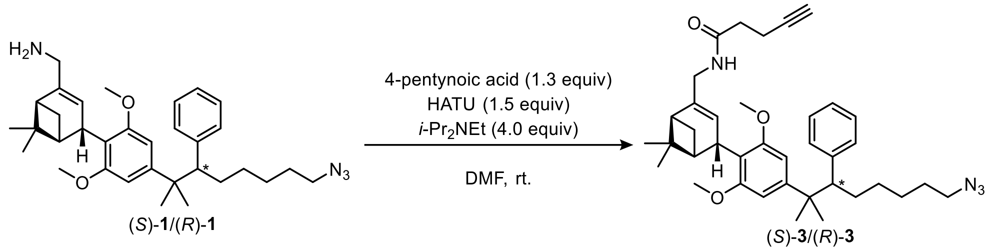

To a solution of 4-pentynoic acid (0.6 mg, 6.3 µmol, 1.3 equiv) in DMF (50 µL) was added HATU (2.8 mg, 7.2 µmol, 1.5 equiv) and *i*-Pr_2NEt (3.5 µL, 20 µmol, 4.0 equiv) and the reaction mixture was stirred at rt for 5 min before being added to a mixture of **(*S*)-1/(*R*)-1** (2.6 mg, 4.8 µmol, 1.0 equiv) in DMF (50 µL). The reaction mixture was stirred at rt for 1 h and concentrated *in vacuo*. The crude product was purified by preparative TLC (SiO_2, 35% EtOAc in hexanes) to afford the title product **(*S*)-3/(*R*)-3** as a colourless waxy solid (2.7 mg, 90%).

1:1 mixture of diastereomers – all signals reported: **^{1}H NMR** (500 MHz, CD_2Cl_2) δ 7.26 – 7.16 (m, 3H), 7.11 – 7.05 (m, 2H), 6.46 (s, 2H), 5.63 (ddt, *J* = 5.3, 2.9, 1.5 Hz, 1H), 5.53 (bt, *J* = 5.4 Hz, 1H), 4.00 – 3.94 (m, 1H), 3.87 – 3.81 (m, 2H), 3.70 (s, 3H), 3.69 (s, 3H), 3.11 (t, *J* = 6.9 Hz, 2H), 2.80 – 2.75 (m, 1H), 2.54 – 2.48 (m, 2H), 2.39 (t, *J* = 7.1 Hz, 2H), 2.18 (dddd, *J* = 8.4, 5.5, 4.6, 1.3 Hz, 1H), 2.10 (td, *J* = 5.6, 1.4 Hz, 1H), 2.05 – 1.99 (m, 2H), 1.72 – 1.57 (m, 3H), 1.48 – 1.30 (m, 4H), 1.29 (s, 3H), 1.26 (s, 3H), 1.12 (s, 3H), 0.96 (s, 3H), 0.93 (m, 2H). **^{13}C NMR** (126 MHz, CD_2Cl_2) δ 170.9, 158.8, 149.4, 142.3, 139.0, 130.6, 127.9, 126.6, 124.2, 118.4, 104.3, 83.8, 69.4, 57.5, 56.3, 52.0, 48.0, 44.9, 42.2, 41.3, 38.1, 36.0, 30.3, 29.7, 29.2, 29.1, 28.3, 28.1, 27.1, 26.6, 24.0, 21.3, 15.4. **IR** (neat, ν_{max}/cm^{-1}) 3310, 2927, 2857, 2094, 1650, 1604, 1572, 1452, 1411, 1365, 1302, 1261, 1239, 1184, 1120. **HRMS (ESI)**: *m/z* = 633.3767 $[M+Na]^+$ (calc. for $C_{38}H_{50}N_4NaO_3$ *m/z* = 633.3775).

Synthesis of (*S*)-SI-2

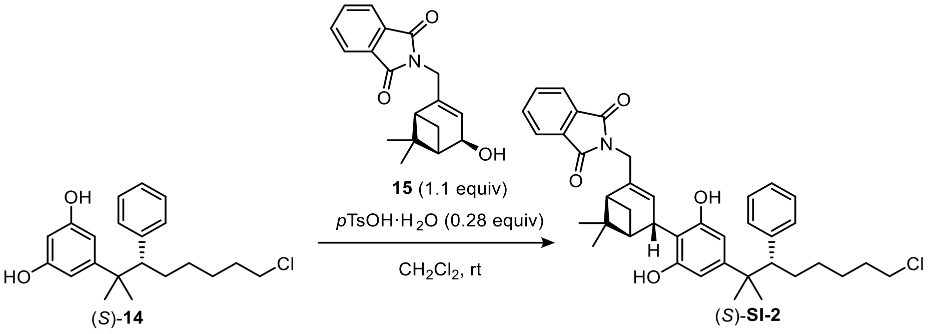

To a solution of resorcinol (*S*)-**14** (51.0 mg, 147 µmol, 1.0 equiv) and *p*TsOH·H_2O (7.8 mg, 41.1 µmol, 0.28 equiv) in CH_2Cl_2 (6.0 mL) was added allylic alcohol **15** (48.0 mg, 162 µmol, 1.1 equiv) and the light-yellow solution was stirred at rt for 1.5 h. Additional allylic alcohol **15** (10.9 mg, 36.7 µmol, 0.25 equiv) was added and the reaction was stirred for 1 h. The reaction was stopped by addition of sat. aq. $NaHCO_3$ (3 mL), the layers were separated and the aqueous layer was extracted with Et_2O (3 × 5 mL). Combined organic extracts were dried over $MgSO_4$ and concentrated *in vacuo*. Purification by flash column chromatography (SiO_2; 5 – 30% EtOAc in hexanes) afforded the product (*S*)-**SI-2** as a colourless foam (62.0 mg, 67%).

^{1}H NMR (400 MHz, $CDCl_3$) δ 7.93 – 7.86 (m, 2H), 7.76 – 7.70 (m, 2H), 7.28 – 7.14 (m, 3H), 7.11 – 7.03 (m, 2H), 6.49 – 6.31 (s, 2H), 6.05 – 5.98 (m, 1H), 5.90 (bs, 2H), 4.47 (ddd, *J* = 15.5, 3.0, 1.9 Hz, 1H), 4.27 – 4.19 (m, 1H), 4.05 – 3.98 (m, 1H), 3.35 (t, *J* = 6.8 Hz, 2H), 2.71 (dd, *J* = 12.1, 2.9 Hz, 1H), 2.37 – 2.21 (m, 3H), 1.66 – 1.15 (m, 7H), 1.32 (s, 3H), 1.17 (s, 3H), 1.01 (s, 3H), 1.00 (s, 3H), 0.98 – 0.82 (m, 2H). **^{13}C NMR** (101 MHz, $CDCl_3$) δ 168.5, 154.9, 149.9, 148.1, 141.8, 134.3, 132.0, 130.1, 127.6, 126.2, 123.7, 120.7, 111.9, 107.4, 56.8, 47.3, 45.3, 44.3, 43.3, 41.3, 41.2, 37.8, 32.4, 29.6, 29.2, 28.2, 27.5, 26.7, 26.0, 22.6, 20.7. **IR** (neat, ν_{max}/cm^{-1}): 3454, 2933, 1770, 1711, 1623, 1574, 1426, 1393, 754, 729, 705. **HRMS (ESI)**: *m/z* = 648.2844 $[M+Na]^+$ (calc. for $C_{39}H_{44}ClNNaO_4$ *m/z* = 648.2851). $[\alpha]^{25}_D$ = +72.790 ± 0.118 (c = 1.0, $CHCl_3$).

Synthesis of (*R*)-SI-2

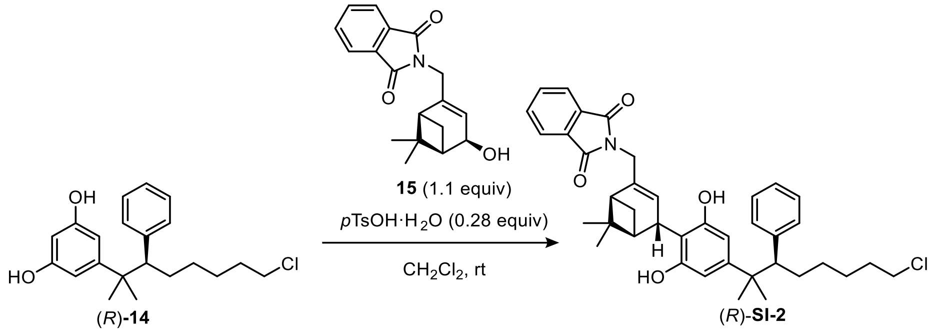

To a solution of resorcinol (*R*)-**14** (56.2 mg, 162 µmol, 1.0 equiv) and *p*TsOH·H_2O (8.6 mg. 45.3 µmol, 0.28 equiv) in CH_2Cl_2 (7.0 mL) was added allylic alcohol **15** (53.0 mg, 178 µmol, 1.1 equiv) and the light-yellow solution was stirred at rt. for 1.5 h. More allylic alcohol **15** (12.0 mg, 40.5 µmol, 0.25 equiv) was added and the reaction was stirred for 1 h. The reaction was stopped by addition of sat. aq. $NaHCO_3$ (3 mL), the layers were separated and the aqueous layer was extracted with Et_2O (3 × 5 mL). Combined organic extracts were dried over $MgSO_4$ and concentrated *in vacuo*. Purification by flash column chromatography (SiO_2; using 5 – 30% EtOAc in hexanes) afforded the product (*R*)-**SI-2** as a colourless foam (72.0 mg, 71%).

^{1}H NMR (400 MHz, $CDCl_3$) δ 7.92 – 7.86 (m, 2H), 7.76 – 7.71 (m, 2H), 7.27 – 7.16 (m, 3H), 7.12 – 7.06 (m, 2H), 6.41 (s, 2H), 6.05 – 5.99 (m, 1H), 5.88 (bs, 2H), 4.47 (ddd, *J* = 15.5, 3.0, 1.9 Hz, 1H), 4.27 – 4.18 (m, 1H), 4.05 – 3.99 (m, 1H), 3.36 (t, *J* = 6.7 Hz, 2H), 2.72 (dd, *J* = 12.1, 2.9 Hz, 1H), 2.36 – 2.22 (m, 3H), 1.70 – 1.44 (m, 4H), 1.40 – 1.13 (m, 3H), 1.32 (s, 3H), 1.17 (s, 3H), 1.00 (s, 3H), 0.99 (s, 3H), 0.97 – 0.81 (m, 2H). **^{13}C NMR** (101 MHz, $CDCl_3$) δ 168.5, 155.0, 150.1, 148.1, 141.7, 134.3, 132.1, 130.1, 127.6, 126.2, 123.7, 120.8, 111.9, 107.3, 56.7, 47.4, 45.3, 44.3, 43.3, 41.3, 41.2, 37.8, 32.4, 29.6, 29.1, 28.2, 27.5, 26.7, 26.0, 22.5, 20.7. **IR** (neat, ν_{max}/cm^{-1}): 3454, 2931, 2867, 1770, 1710, 1623, 1574, 1426, 1392, 1340, 1112, 1024, 946, 764, 728, 705. **HRMS (ESI)**: *m/z* = 626.3030 $[M+H]^+$ (calc. for $C_{39}H_{45}ClNO_4$ *m/z* = 626.3032). **$[\alpha]^{25}_D$** = +114.140 ± 0.504 (c = 1.0, $CHCl_3$).

Synthesis of (*S*)-16

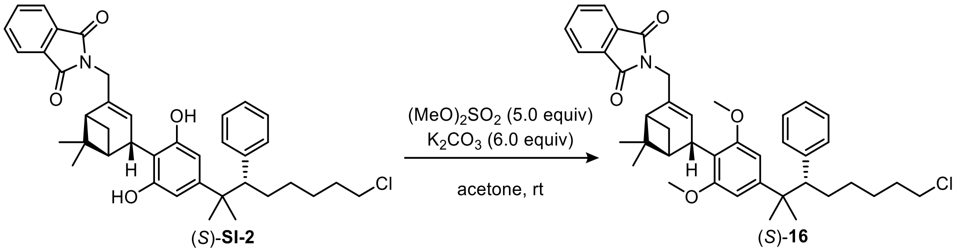

To a suspension of (*S*)-**SI-2** (57.0 mg, 91.0 µmol, 1.0 equiv) and K_2CO_3 (75.5 mg. 546 µmol, 6.0 equiv) in acetone (0.9 mL) was added $(MeO)_2SO_2$ (43 µL, 455 µmol, 5.0 equiv) and the solution was stirred at rt overnight. The reaction mixture was diluted with Et_2O (5 mL), filtered and concentrated *in vacuo*. Purification by flash column chromatography (SiO_2; 0 – 15% acetone in hexanes) afforded the product (*S*)-**16** as a white foam (49.0 mg, 82%).

^{1}H NMR (400 MHz, $CDCl_3$) δ 7.89 – 7.82 (m, 2H), 7.75 – 7.67 (m, 2H), 7.25 – 7.12 (m, 3H), 7.05 – 6.98 (m, 2H), 6.39 (s, 2H), 5.62 – 5.56 (m, 1H), 4.35 – 4.28 (m, 1H), 4.24 (ddd, *J* = 15.4, 2.3, 1.6 Hz, 1H), 3.98 – 3.94 (m, 1H), 3.68 (s, 6H), 3.36 (t, *J* = 6.7 Hz, 2H), 2.71 (dd, *J* = 12.0, 2.9 Hz, 1H), 2.23 – 2.12 (m, 2H), 2.05 – 1.97 (m, 1H), 1.74 (d, *J* = 8.0 Hz, 1H), 1.68 – 1.38 (m, 4H), 1.34 – 1.14 (m, 2H), 1.27 (s, 3H), 1.25 (s, 3H), 1.12 (s, 3H), 1.02 – 0.83 (m, 2H), 0.94 (s, 3H). **^{13}C NMR** (101 MHz, $CDCl_3$) δ 168.3, 158.3, 148.4, 141.7, 136.2, 133.9, 132.4, 130.1, 127.5, 126.2, 123.9, 123.3, 117.9, 103.7, 57.2, 55.9, 47.6, 45.2, 44.4, 42.6, 41.8, 41.2, 37.5, 32.5, 29.2, 29.0, 27.6, 27.6, 26.8, 26.4, 23.9, 20.9. **IR** (neat, ν_{max}/cm^{-1}): 2930, 2863, 1772, 1715, 1603, 1572, 1390, 1239, 1117, 729, 704, 671. **HRMS (ESI)**: *m/z* = 676.3152 [M+Na]$^+$ (calc. for $C_{41}H_{48}ClNNaO_4$ *m/z* = 676.3164). **[α]$^{25}_D$** = +33.551 ± 0.235 (c = 1.0, $CHCl_3$).

Synthesis of (*R*)-16

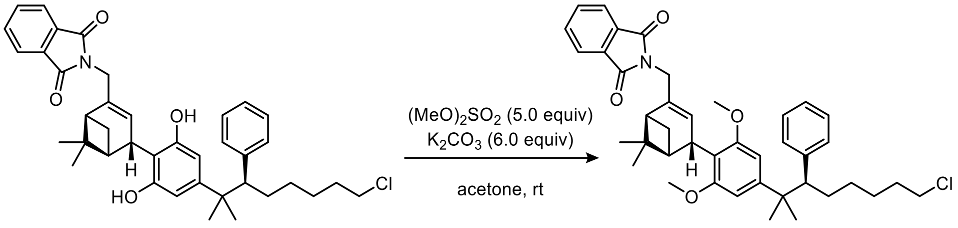

To a suspension of (*R*)-**SI-2** (70.0 mg, 112 µmol, 1.0 equiv) and K_2CO_3 (92.7 mg. 671 µmol, 6.0 equiv) in acetone (1.0 mL) was added $(MeO)_2SO_2$ (53 µL. 559 µmol, 5.0 equiv) and the solution was stirred at rt overnight. The reaction mixture was diluted with Et_2O (5 mL), filtered and concentrated *in vacuo*. Purification by flash column chromatography (SiO_2; 0 – 15% acetone in hexanes) afforded the product (*R*)-**16** as a white foam (59.0 mg, 81%).

^{1}H NMR (400 MHz, $CDCl_3$) δ 7.88 – 7.83 (m, 2H), 7.74 – 7.68 (m, 2H), 7.25 – 7.15 (m, 3H), 7.06 – 7.00 (m, 2H), 6.39 (s, 2H), 5.62 – 5.58 (m, 1H), 4.32 (ddd, *J* = 15.4, 2.2, 1.6 Hz, 1H), 4.24 (ddd, *J* = 15.4, 2.3, 1.6 Hz, 1H), 3.99 – 3.94 (m, 1H), 3.68 (s, 6H), 3.37 (t, *J* = 6.8, 2H), 2.71 (dd, *J* = 12.1, 2.9 Hz, 1H), 2.22 – 2.11 (m, 2H), 2.05 – 1.99 (m, 1H), 1.72 (d, *J* = 8.1 Hz, 1H), 1.66 – 1.38 (m, 4H), 1.34 – 1.15 (m, 2H), 1.27 (s, 3H), 1.25 (s, 3H), 1.11 (s, 3H), 1.02 – 0.84 (m, 2H), 0.94 (s, 3H). **^{13}C NMR** (101 MHz, $CDCl_3$) δ 168.3, 158.3, 148.5, 141.7, 136.1, 133.9, 132.4, 130.1, 127.5, 126.2, 123.9, 123.3, 117.9, 103.6, 57.1, 55.8, 47.6, 45.2, 44.3, 42.6, 41.8, 41.2, 37.5, 32.4, 29.2, 29.0, 27.6, 27.6, 26.8, 26.4, 23.8, 20.9. **IR** (neat, ν_{max}/cm^{-1}): 2933, 2865, 1772, 1715, 1603, 1572, 1390, 1239, 1117, 730, 704. **HRMS (ESI)**: *m/z* = 676.3163 $[M+Na]^+$ (calc. for $C_{41}H_{48}ClNNaO_4$ *m/z* = 676.3164). **$[\alpha]^{25}_D$** = +71.187 ± 0.169 (c = 1.0, $CHCl_3$).

Synthesis of (*S*)-SI-3

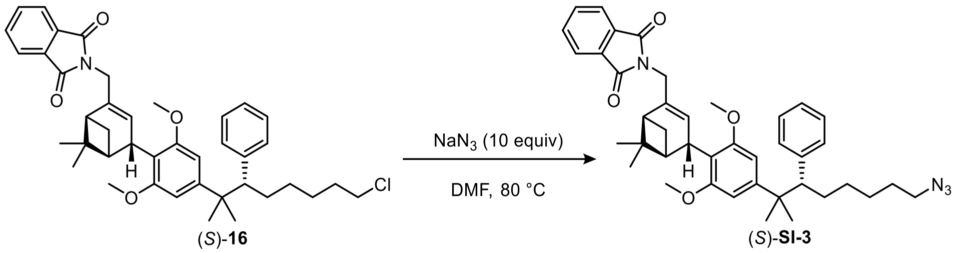

To solution of (*S*)-**16** (42.0 mg, 64.1 µmol, 1.0 equiv) in DMF (0.35 mL) was added NaN_3 (41.7 mg, 642 µmol, 10 equiv) and the reaction mixture was stirred at 80 °C for 24 h. The mixture was allowed to cool down to rt and diluted with 5% aq. LiCl (3 mL) and Et_2O (3 mL). The layers were separated and the aqueous phase was extracted with Et_2O (3 × 3 mL). Combined organic extracts were dried over $MgSO_4$, filtered and concentrated *in vacuo*. Purification by flash column chromatography (SiO_2, 0 – 10% EtOAc in hexanes) afforded the product (*S*)-**SI-3** as a white foam (38.0 mg, 90%).

^{1}H NMR (400 MHz, $CDCl_3$) δ 7.88 – 7.82 (m, 2H), 7.74 – 7.66 (m, 2H), 7.25 – 7.13 (m, 3H), 7.05 – 6.98 (m, 2H), 6.39 (s, 2H), 5.63 – 5.56 (m, 1H), 4.36 – 4.28 (m, 1H), 4.24 (ddd, *J* = 15.4, 2.3, 1.6 Hz, 1H), 3.99 – 3.94 (m, 1H), 3.68 (s, 6H), 3.09 (t, *J* = 6.9 Hz, 2H), 2.70 (dd, *J* = 12.0, 2.9 Hz, 1H), 2.24 – 2.11 (m, 2H), 2.04 – 1.98 (m, 1H), 1.74 (d, *J* = 8.0 Hz, 1H), 1.68 – 1.08 (m, 6H), 1.27 (s, 3H), 1.25 (s, 3H), 1.12 (s, 3H), 1.02 – 0.83 (m, 2H), 0.94 (s, 3H). **^{13}C NMR** (101 MHz, $CDCl_3$) δ 168.3, 158.3, 148.4, 141.7, 136.2, 133.9, 132.4, 130.1, 127.5, 126.2, 123.9, 123.3, 117.9, 103.6, 57.2, 55.8, 51.5, 47.6, 44.3, 42.6, 41.8, 41.2, 37.5, 29.3, 29.0, 28.7, 27.9, 27.6, 26.6, 26.4, 23.9, 20.9. **IR** (neat, ν_{max}/cm^{-1}): 2933, 2865, 2094, 1772, 1716, 1603, 1572, 1390, 1240, 1119. **(ESI)**: *m/z* = 661.3746 [M+H]$^+$ (calc. for $C_{41}H_{49}N_4O_4$ *m/z* = 661.3748). **$[\alpha]^{25}_D$** = +32.189 ± 0.150 (c = 1.0, $CHCl_3$).

Synthesis of (*R*)-SI-3

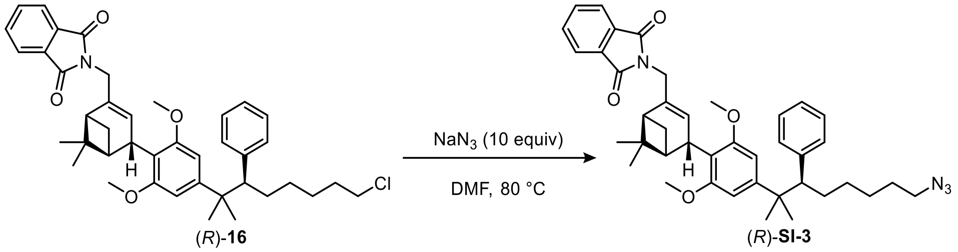

To solution of (*R*)-**16** (57.0 mg, 87.1 µmol, 1.0 equiv) in DMF (0.40 mL) was added NaN_3 (56.6 mg, 871 µmol, 10 equiv) and the reaction mixture was stirred at 80 °C for 24 h. The mixture was allowed to cool down to rt and diluted with 5% aq. LiCl (3 mL) and Et_2O (3 mL). The layers were separated and the aqueous phase was extracted with Et_2O (3 × 3 mL). Combined organic extracts were dried over $MgSO_4$, filtered and concentrated *in vacuo*. Purification by flash column chromatography (SiO_2, 0 – 10% EtOAc in hexanes) afforded the product (*R*)-**SI-3** as a white foam (50.5 mg, 88%).

^{1}H NMR (400 MHz, $CDCl_3$) δ 7.89 – 7.81 (m, 2H), 7.75 – 7.67 (m, 2H), 7.25 – 7.15 (m, 3H), 7.06 – 6.99 (m, 2H), 6.39 (s, 2H), 5.63 – 5.5 (m, 1H), 4.35 – 4.29 (m, 1H), 4.28 – 4.20 (m, 1H), 4.00 – 3.93 (m, 1H), 3.68 (s, 6H), 3.09 (t, *J* = 6.9 Hz, 2H), 2.71 (dd, *J* = 12.1, 2.9 Hz, 1H), 2.23 – 2.10 (m, 2H), 2.06 – 1.98 (m, 1H), 1.72 (d, *J* = 8.1 Hz, 1H), 1.68 – 1.53 (m, 1H), 1.47 – 1.09 (m, 5H), 1.27 (s, 3H), 1.25 (s, 3H), 1.11 (s, 3H), 1.03 – 0.85 (m, 2H), 0.94 (s, 3H). **^{13}C NMR** (101 MHz, $CDCl_3$) δ 168.3, 158.3, 148.5, 141.7, 136.1, 133.9, 132.4, 130.1, 127.5, 126.2, 124.0, 123.3, 117.9, 103.6, 57.2, 55.8, 51.5, 47.6, 44.3, 42.6, 41.8, 41.2, 37.5, 29.2, 29.0, 28.7, 27.9, 27.6, 26.6, 26.4, 23.8, 20.9. **IR** (neat, ν_{max}/cm^{-1}): 2933, 2864, 2094, 1772, 1715, 1603, 1572, 1424, 1410, 1390, 1239, 1118, 730, 711. **HRMS (ESI)**: *m/z* = 683.3565 $[M+Na]^+$ (calc. for $C_{41}H_{48}N_4NaO_4$ *m/z* = 683.3568). **$[\alpha]^{25}_D$** = +73.648 ± 0.128 (c = 1.0, $CHCl_3$).

Synthesis of (*S*)-1

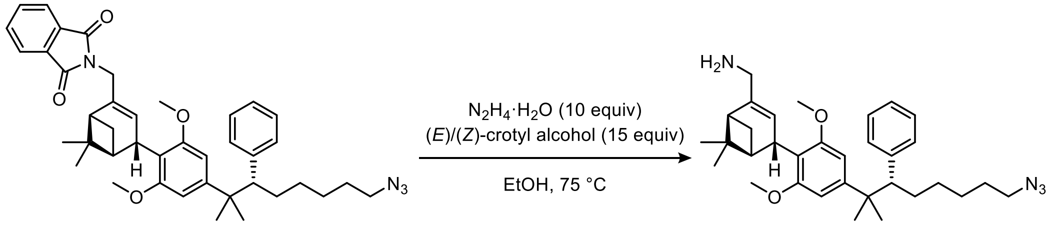

(*S*)-**SI-3** (*S*)-**1**

To a solution of (*S*)-**SI-3** (36.0 mg, 54.4 µmol, 1.0 equiv) in EtOH (1.1 mL) was added $N_2H_4{\cdot}H_2O$ (27 µL, 545 µmol, 10 equiv) and (*E*)/(*Z*)-crotyl alcohol (70 µL, 817 µmol, 15 equiv) and the solution was heated to 75 °C and stirred for 2 h. Precipitation of a white solid occurred, and the mixture was filtered over cotton, which was rinsed with EtOH. The filtrate was concentrated *in vacuo*. Purification by flash column chromatography (SiO_2; 1% 7.0 M NH_3 in MeOH, 1 – 4% MeOH in CH_2Cl_2) afforded the product (*S*)-**1** as a white foam (25.5 mg, 88%).

^{1}H NMR (400 MHz, $CDCl_3$) δ 7.25 – 7.15 (m, 3H), 7.06 – 7.00 (m, 2H), 6.43 (s, 2H), 5.58 – 5.54 (m, 1H), 4.03 – 3.97 (m, 1H), 3.70 (s, 6H), 3.24 (t, *J* = 1.8 Hz, 2H), 3.11 (t, *J* = 6.9 Hz, 2H), 2.72 (dd, *J* = 12.1, 2.9 Hz, 1H), 2.23 – 2.17 (m, 1H), 2.14 (td, *J* = 5.6, 1.4 Hz, 1H), 2.06 (ddt, *J* = 5.8, 3.9, 1.9 Hz, 1H), 1.72 (d, *J* = 8.4 Hz, 1H), 1.69 – 1.32 (m, 4H), 1.31 (s, 3H), 1.27 (s, 3H), 1.24 – 1.16 (m, 2H), 1.14 (s, 3H), 1.02 – 0.88 (m, 2H), 0.97 (s, 3H). **^{13}C NMR** (101 MHz, $CDCl_3$) δ 158.4, 148.4, 143.6, 141.7, 130.1, 127.5, 126.3, 121.0, 118.5, 104.0, 57.3, 56.0, 51.5, 47.7, 47.6, 44.7, 41.8, 41.0, 37.6, 29.3, 28.9, 28.7, 28.1, 27.9, 26.7, 26.5, 24.0, 21.2. **IR** (neat, ν_{max}/cm^{-1}): 2932, 2864, 2094, 1604, 1572, 1452, 1410, 1240, 1120. **HRMS (ESI)**: *m/z* = 531.3690 $[M+H]^+$ (calc. for $C_{33}H_{47}N_4O_2$ *m/z* = 531.3694). **$[\alpha]^{25}_D$** = +44.526 ± 0.423 (c = 1.0, $CHCl_3$).

Synthesis of (*R*)-1

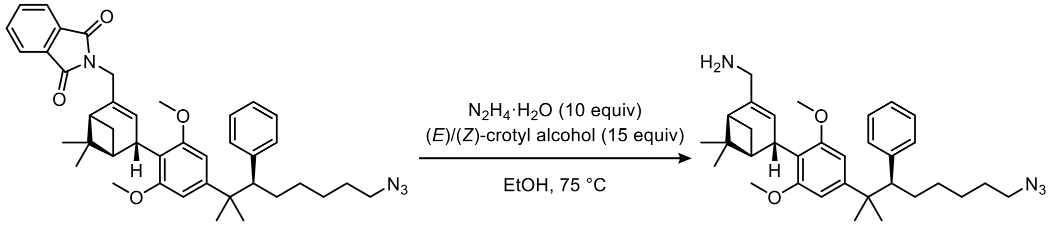

To a solution of (*R*)-**SI-3** (37.0 mg, 55.9 µmol, 1.0 equiv) in EtOH (1.1 mL) was added $N_2H_4·H_2O$ (28 µL, 560 µmol, 10 equiv) and (*E*)/(*Z*)-crotyl alcohol (72 µL, 840 µmol, 15 equiv) and the solution was heated to 75 °C and stirred for 2 h. Precipitation of a white solid occurred, and the mixture was filtered over cotton, which was rinsed with EtOH. The filtrate was concentrated *in vacuo*. Purification by flash column chromatography (SiO_2; 1% 7.0 M NH_3 in MeOH, 1 – 4% MeOH in CH_2Cl_2) afforded the product (*R*)-**1** as a white foam (28.0 mg, 94%).

^{1}H NMR (400 MHz, $CDCl_3$) δ 7.26 – 7.15 (m, 3H), 7.03 (dt, *J* = 6.0, 1.6 Hz, 2H), 6.43 (s, 2H), 5.58 – 5.55 (m, 1H), 4.01 – 3.98 (m, 1H), 3.71 (s, 6H), 3.23 (t, *J* = 1.8 Hz, 2H), 3.11 (t, *J* = 6.9 Hz, 2H), 2.72 (dd, *J* = 12.1, 2.9 Hz, 1H), 2.20 (dt, *J* = 8.3, 5.5 Hz, 1H), 2.13 (td, *J* = 5.6, 1.4 Hz, 1H), 2.10 – 2.05 (m, 1H), 1.70 (d, *J* = 8.3 Hz, 1H), 1.68 – 1.57 (m, 1H), 1.53 – 1.33 (m, 3H), 1.31 (s, 3H), 1.27 (s, 3H), 1.25 – 1.17 (m, 2H), 1.14 (s, 3H), 1.01 – 0.86 (m, 2H). 0.97 (s, 3H). **^{13}C NMR** (101 MHz, $CDCl_3$) δ 158.4, 148.4, 143.7, 141.7, 130.1, 127.5, 126.2, 120.9, 118.5, 104.0, 57.2, 56.0, 51.5, 47.7, 47.7, 44.7, 41.8, 41.0, 37.5, 29.3, 28.9, 28.7, 28.1, 27.9, 26.7, 26.5, 23.9, 21.2. **IR** (neat, ν_{max}/cm^{-1}): 2933, 2864, 2094, 1604, 1572, 1452, 1410, 1240, 1120. **HRMS (ESI)**: *m/z* = 531.3690 $[M+H]^+$ (calc. for $C_{33}H_{47}N_4O_2$ *m/z* = 531.3694). **$[\alpha]^{25}_D$** = +101.994 ± 0.218 (c = 1.0, $CHCl_3$).

Synthesis of (*S*)-2

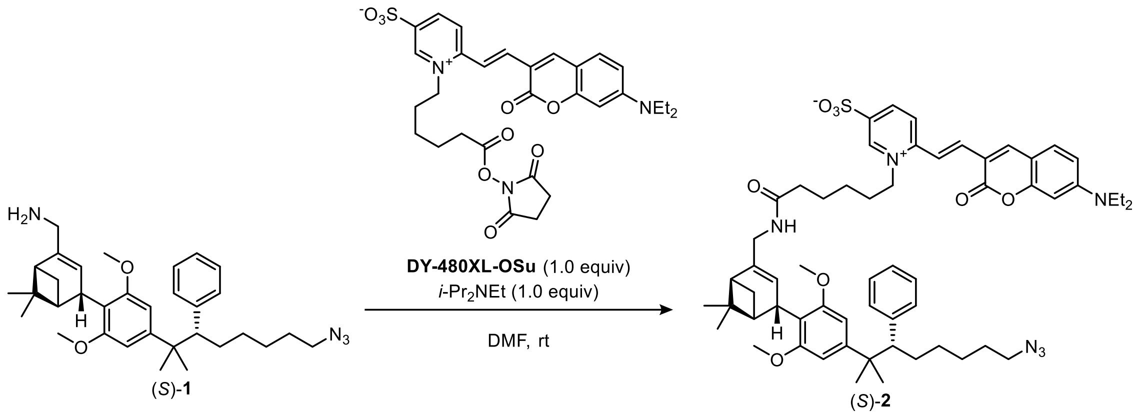

To a solution of (*S*)-**1** (2.3 mg, 4.3 µmol, 1.0 equiv) in DMF (50 µL) was added NHS ester **DY-480XL-OSu** (2.7 mg, 4.3 µmol, 1.0 equiv) in DMF (30 µL) and *i*-Pr_2NEt (0.8 µL, 4.3 µmol, 1.0 equiv). The deep red solution was stirred at rt and under exclusion of light overnight, after which the mixture was concentrated *in vacuo*. Purification by preparative TLC (SiO_2, 5% MeOH in CH_2Cl_2) afforded the product (*S*)-**2** as dark red waxy solid (4.3 mg, 97%).

^{1}H NMR (500 MHz, CD_2Cl_2) δ 8.89 (s, 1H), 8.66 (d, *J* = 8.5 Hz, 1H), 8.20 – 8.04 (m, 2H), 7.88 (s, 1H), 7.48 – 7.40 (m, 2H), 7.26 – 7.21 (m, 2H), 7.20 – 7.16 (m, 1H), 7.09 – 7.04 (m, 2H), 6.69 (dd, *J* = 9.0, 2.5 Hz, 1H), 6.51 (d, *J* = 2.4 Hz, 1H), 6.45 (s, 2H), 5.83 (t, *J* = 4.8 Hz, 1H), 5.67 – 5.56 (m, 1H), 4.58 – 4.50 (m, 2H), 3.97 – 3.94 (m, 1H), 3.91 – 3.77 (m, 2H), 3.68 (s, 6H), 3.47 (q, *J* = 7.1 Hz, 4H), 3.10 (t, *J* = 6.9 Hz, 2H), 2.77 (dd, *J* = 12.0, 2.8 Hz, 1H), 2.32 – 1.94 (m, 7H), 1.81 – 1.51 (m, 6H), 1.48 – 1.30 (m, 3H), 1.27 (s, 3H), 1.26 (s, 3H), 1.23 (t, *J* = 7.1 Hz, 6H), 1.20 – 1.12 (m, 2H), 1.11 (d, *J* = 3.5 Hz, 3H), 1.00 – 0.85 (m, 2H), 0.95 (s, 3H). **^{13}C NMR** (126 MHz, CD_2Cl_2) δ 172.3, 158.8, 157.6, 153.6, 149.4, 148.1, 142.6, 142.3, 139.3, 131.4, 130.6, 127.9, 126.6, 124.8, 123.8, 118.5, 115.9, 113.8, 110.9, 109.5, 104.3, 97.3, 59.4, 57.4, 56.4, 52.0, 48.1, 45.8, 44.9, 44.8, 42.2, 41.3, 38.1, 36.5, 30.0, 29.7, 29.2, 29.1, 28.3, 28.1, 27.0, 26.6, 26.4, 25.1, 24.0, 21.3, 12.8. **IR** (neat, ν_{max}/cm^{-1}): 2917, 2851, 2094, 1717, 1578, 1504, 1416, 1272, 1134, 1043. **HRMS (ESI)**: *m/z* = 1027.5357 $[M+H]^+$ (calc. for $C_{59}H_{75}N_6O_8S$ *m/z* = 1027.5362).

Synthesis of (*R*)-2

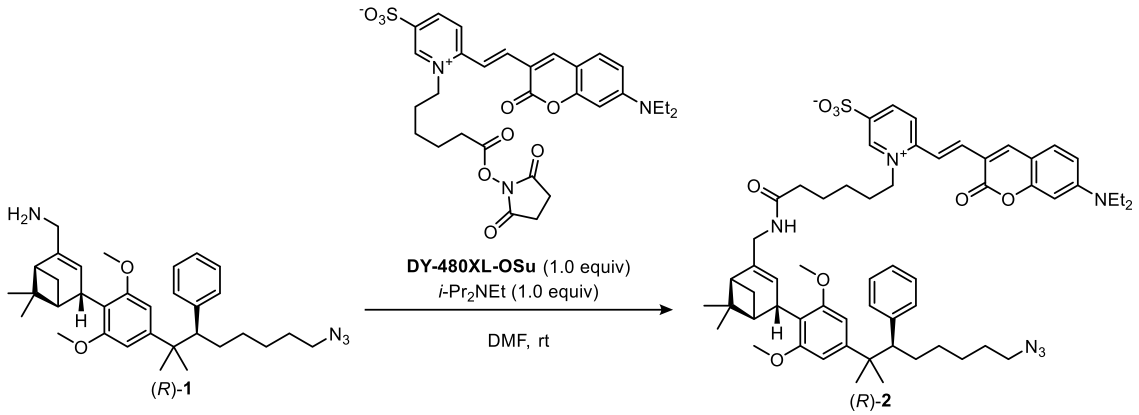

To a solution of (*R*)-**1** (2.2 mg, 4.1 µmol, 1.0 equiv) in DMF (50 µL) was added NHS ester **DY-480XL-OSu** (2.5 mg, 4.1 µmol, 1.0 equiv) in DMF (30 µL) and *i*-Pr_2NEt (0.7 µL, 4.1 µmol, 1.0 equiv). The deep red solution was stirred at rt and under exclusion of light overnight, after which the mixture was concentrated *in vacuo*. Purification by preparative TLC (SiO_2; 5% MeOH in CH_2Cl_2) afforded the product (*R*)-**2** as dark red waxy solid (3.7 mg, 87%).

^{1}H NMR (500 MHz, CD_2Cl_2) δ 8.89 (s, 1H), 8.66 (d, *J* = 8.9 Hz, 1H), 8.18 – 8.05 (m, 2H), 7.88 (s, 1H), 7.49 – 7.40 (m, 2H), 7.26 – 7.20 (m, 2H), 7.20 – 7.16 (m, 1H), 7.07 (dt, *J* = 8.0, 1.4 Hz, 2H), 6.70 (dd, *J* = 9.0, 2.5 Hz, 1H), 6.51 (d, *J* = 2.5 Hz, 1H), 6.45 (s, 2H), 5.83 (t, *J* = 5.8 Hz, 1H), 5.65 – 5.56 (m, 1H), 4.60 – 4.48 (m, 2H), 4.01 – 3.91 (m, 1H), 3.90 – 3.79 (m, 2H), 3.68 (s, 6H), 3.47 (q, *J* = 7.1 Hz, 4H), 3.10 (td, *J* = 7.0, 1.8 Hz, 2H), 2.76 (dd, *J* = 12.1, 3.0 Hz, 1H), 2.25 (t, *J* = 7.5 Hz, 2H), 2.22 – 1.98 (m, 5H), 1.80 – 1.50 (m, 6H), 1.46 – 1.30 (m, 3H), 1.28 (s, 3H), 1.26 (s, 3H), 1.23 (t, *J* = 7.1 Hz, 6H), 1.19 – 1.12 (m, 2H), 1.10 (s, 3H), 1.00 – 0.84 (m, 2H), 0.95 (s, 3H). **^{13}C NMR** (126 MHz, CD_2Cl_2) δ 172.3, 160.7, 158.8, 157.6, 153.6, 153.1, 149.4, 148.0, 142.7, 142.3, 141.4, 139.3, 131.3, 130.6, 127.9, 126.6, 124.8, 123.8, 118.4, 115.9, 113.8, 110.9, 109.5, 104.3, 97.3, 59.4, 57.4, 56.3, 52.0, 48.1, 45.8, 44.9, 44.8, 42.2, 41.3, 38.1, 36.5, 30.0, 29.7, 29.2, 29.1, 28.3, 28.1, 27.1, 26.6, 26.4, 25.1, 24.0, 21.3, 12.8. **IR** (neat, ν_{max}/cm^{-1}): 2918, 2851, 2094, 1714, 1579, 1504, 1415, 1240, 1042. **HRMS (ESI)**: *m/z* = 1027.5357 [M+H]$^+$ (calc. for $C_{59}H_{75}N_6O_8S$ *m/z* = 1027.5362).

Synthesis of (*S*)-3

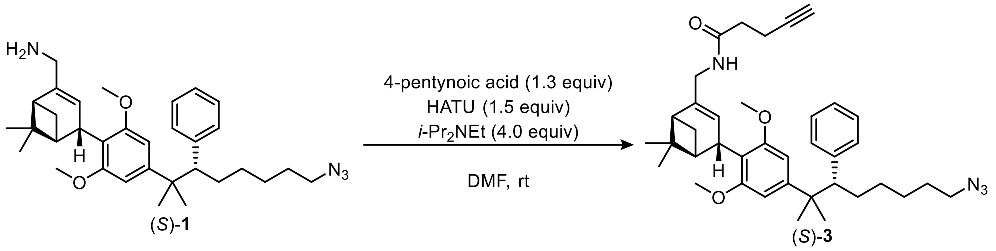

To a solution of 4-pentynoic acid (0.5 mg, 5.3 µmol, 1.3 equiv) in DMF (50 µL) was added HATU (2.4 mg, 6.2 µmol, 1.5 equiv) and *i*-Pr_2NEt (2.9 µL, 17 µmol, 4.0 equiv) and the reaction mixture was stirred at rt for 5 min before being added to **(*S*)-1** (2.2 mg, 4.1 µmol, 1.0 equiv) in DMF (50 µL). The reaction mixture was stirred at rt for 1 h and concentrated *in vacuo*. The crude product was purified by preparative TLC (SiO_2, 35% EtOAc in hexanes) to afford the title product **(*S*)-3** as a colourless waxy solid (2.3 mg, 91%).

^{1}H NMR (400 MHz, $CDCl_3$) δ 7.25 – 7.14 (m, 3H), 7.06 – 6.98 (m, 2H), 6.42 (s, 2H), 5.68 – 5.62 (m, 1H), 5.54 (t, *J* = 4.8 Hz, 1H), 4.02 – 3.95 (m, 1H), 3.89 (d, *J* = 5.4 Hz, 2H), 3.70 (s, 6H), 3.11 (t, *J* = 6.9 Hz, 2H), 2.72 (dd, *J* = 12.0, 2.9 Hz, 1H), 2.55 (tdd, *J* = 7.0, 2.7, 0.9 Hz, 2H), 2.43 (td, *J* = 7.1, 1.0 Hz, 2H), 2.24 – 2.14 (m, 1H), 2.11 (td, *J* = 5.6, 1.4 Hz, 1H), 2.10 – 2.02 (m, 1H), 1.99 (t, *J* = 2.6 Hz, 1H), 1.70 (d, *J* = 8.4 Hz, 1H), 1.69 – 1.11 (m, 6H), 1.29 (s, 3H), 1.27 (s, 3H), 1.14 (s, 3H), 1.01 – 0.79 (m, 2H), 0.97 (s, 3H). **^{13}C NMR** (101 MHz, $CDCl_3$) δ 170.8, 158.3, 148.6, 141.7, 138.3, 130.1, 127.6, 126.3, 124.2, 118.0, 103.9, 83.3, 69.5, 57.3, 56.0, 51.5, 47.5, 44.8, 44.5, 41.8, 41.0, 37.6, 35.8, 29.3, 28.9, 28.7, 27.9, 27.9, 26.7, 26.4, 24.0, 21.2, 15.1. **IR** (neat, ν_{max}/cm^{-1}) 3308, 2925, 2853, 2094, 1652, 1603, 1571, 1452, 1410, 1261, 1120. **HRMS (ESI)**: *m/z* = 611.3947 $[M+H]^+$ (calc. for $C_{38}H_{51}N_4O_3$ *m/z* = 611.3956).

Synthesis of (*R*)-3

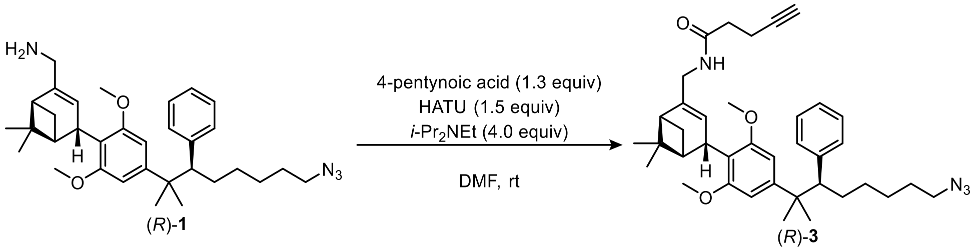

To a solution of 4-pentynoic acid (0.9 mg, 9.0 µmol, 1.3 equiv) in DMF (50 µL) was added HATU (4.0 mg, 10 µmol, 1.5 equiv) and *i*-Pr_2NEt (4.9 µL, 28 µmol, 4.0 equiv) and the reaction mixture was stirred at rt for 5 min before being added to **(*R*)-1** (3.7 mg, 6.9 µmol, 1.0 equiv) in DMF (50 µL). The reaction mixture was stirred at rt for 1 h and concentrated *in vacuo*. The crude product was purified by preparative TLC (SiO_2; 35% EtOAc in hexanes) to afford the title product **(*R*)-3** as colourless waxy solid (4.1 mg, 96%).

^{1}H NMR (400 MHz, $CDCl_3$) δ 7.25 – 7.15 (m, 3H), 7.06 – 6.99 (m, 2H), 6.42 (s, 2H), 5.66 (dt, *J* = 3.0, 1.5 Hz, 1H), 5.54 (s, 1H), 4.04 – 3.93 (m, 1H), 3.89 (d, *J* = 5.4 Hz, 2H), 3.70 (s, 6H), 3.11 (t, *J* = 6.9 Hz, 2H), 2.72 (dd, *J* = 12.1, 2.9 Hz, 1H), 2.55 (tdd, *J* = 7.0, 2.6, 1.0 Hz, 2H), 2.43 (td, *J* = 7.1, 1.0 Hz, 2H), 2.26 – 2.14 (m, 1H), 2.11 (td, *J* = 5.7, 1.4 Hz, 1H), 2.09 – 2.03 (m, 1H), 1.99 (t, *J* = 2.6 Hz, 1H), 1.69 (d, *J* = 8.4 Hz, 1H), 1.67 – 1.12 (m, 6H), 1.29 (s, 3H), 1.27 (s, 3H), 1.14 (s, 3H), 0.99 – 0.79 (m, 2H), 0.97 (s, 3H). **^{13}C NMR** (101 MHz, $CDCl_3$) δ 170.8, 158.3, 148.6, 141.7, 138.3, 130.1, 127.6, 126.3, 124.2, 118.0, 103.9, 83.3, 69.5, 57.3, 56.0, 51.5, 47.5, 44.8, 44.5, 41.8, 41.0, 37.6, 35.8, 29.3, 28.9, 28.7, 28.0, 27.9, 26.7, 26.4, 24.0, 21.2, 15.1. **IR** (neat, ν_{max}/cm^{-1}) 3308, 2925, 2853, 2095, 1652, 1603, 1572, 1452, 1411, 1261, 1120. **HRMS (ESI)**: *m/z* = 611.3947 $[M+H]^+$ (calc. for $C_{38}H_{51}N_4O_3$ *m/z* = 611.3956).

Synthesis of (*S*)-4

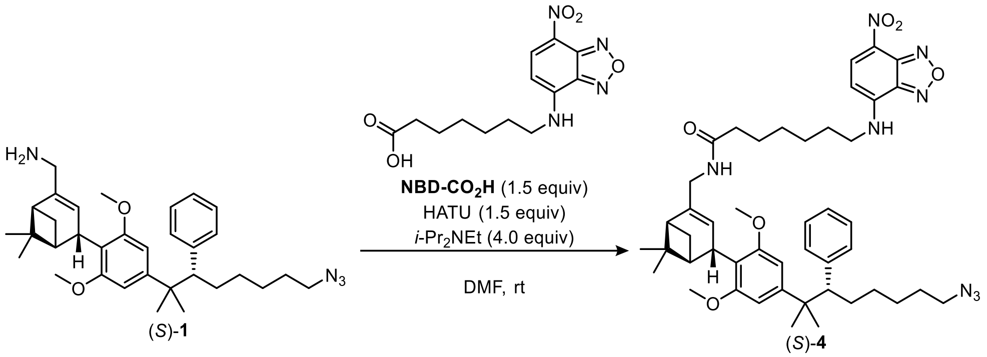

To a solution of **NBD-CO2H** (2.5 mg, 8.1 µmol, 1.5 equiv) in DMF (50 µL) was added HATU (3.1 mg, 8.1 µmol, 1.5 equiv) and *i*-Pr_2NEt (3.8 µL, 22 µmol, 4.0 equiv) and the reaction mixture was stirred at rt for 5 min before being added to **(*S*)-1** (2.9 mg, 5.4 µmol, 1.0 equiv) in DMF (50 µL). The reaction mixture was stirred at rt for 1 h and concentrated *in vacuo*. The crude product was purified by preparative TLC (SiO_2; 2% MeOH in CH_2Cl_2) to afford the title product **(*S*)-4** as yellow amorphous solid (2.8 mg, 62%).

^{1}H NMR (500 MHz, CD_3OD) δ 8.41 (d, *J* = 8.9 Hz, 1H), 7.25 – 7.19 (m, 2H), 7.19 – 7.14 (m, 1H), 7.12 – 7.03 (m, 2H), 6.50 (s, 2H), 6.25 (d, *J* = 8.9 Hz, 1H), 5.62 – 5.55 (m, 1H), 4.00 – 3.92 (m, 1H), 3.82 – 3.71 (m, 2H), 3.69 (s, 6H), 3.48 (bs, 2H), 3.09 (dt, *J* = 6.9, 1.2 Hz, 2H), 2.81 (dd, *J* = 12.0, 2.7 Hz, 1H), 2.24 (t, *J* = 7.2 Hz, 2H), 2.19 – 2.05 (m, 2H), 1.95 (td, *J* = 5.8, 2.9 Hz, 1H), 1.82 – 1.61 (m, 6H), 1.55 – 1.38 (m, 4H), 1.36 – 1.12 (m, 5H), 1.27 (s, 3H), 1.25 (s, 3H), 1.10 (s, 3H), 1.03 – 0.86 (m, 2H), 0.95 (s, 3H). **^{13}C NMR** (126 MHz, CD_3OD) δ 175.8, 159.6, 150.1, 143.0, 139.9, 131.2, 128.5, 127.2, 123.9, 119.0, 104.8, 58.1, 56.3, 52.3, 45.5, 44.9, 42.8, 41.8, 38.7, 37.1, 30.8, 30.1, 29.8, 29.5, 29.3, 28.6, 28.4, 27.8, 27.3, 27.1, 26.8, 24.0, 21.4. **IR** (neat, ν_{max}/cm^{-1}) 3303, 2919, 2852, 2094, 1650, 1579, 1300, 1121. **HRMS (ESI)**: *m/z* = 821.4703 $[M+H]^+$ (calc. for $C_{46}H_{61}N_8O_6$ *m/z* = 821.4709).

Synthesis of (*R*)-4

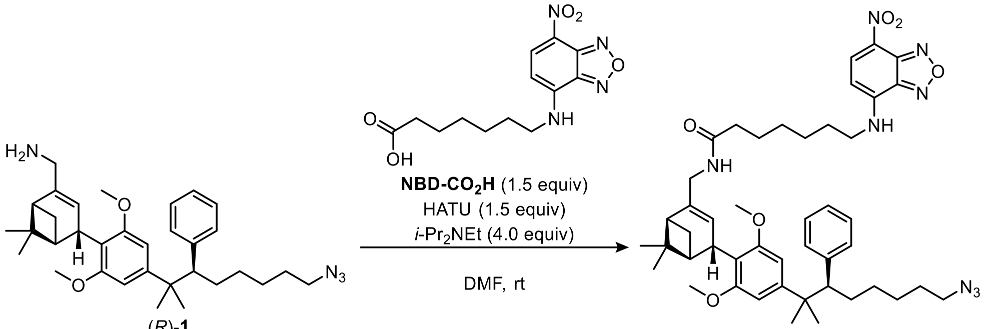

To a solution of **NBD-CO2H** (2.4 mg, 7.6 µmol, 1.5 equiv) in DMF (50 µL) was added HATU (2.9 mg, 7.6 µmol, 1.5 equiv) and *i*-Pr_2NEt (3.5 µL, 20 µmol, 4.0 equiv) and the reaction mixture was stirred at rt for 5 min before being added to **(*R*)-1** (2.7 mg, 5.0 µmol, 1.0 equiv) in DMF (50 µL). The reaction mixture was stirred at rt for 1 h and concentrated *in vacuo*. The crude product was purified by preparative TLC (SiO_2; 2% MeOH in CH_2Cl_2) to afford the title product **(*R*)-4** as yellow amorphous solid (2.5 mg, 60%).

^{1}**H NMR** (500 MHz, CD_3OD) δ 8.42 (d, *J* = 8.8 Hz, 1H), 7.27 – 7.20 (m, 2H), 7.19 – 7.14 (m, 1H), 7.12 – 7.07 (m, 2H), 6.50 (s, 2H), 6.25 (d, *J* = 8.9 Hz, 1H), 5.61 – 5.56 (m, 1H), 4.00 – 3.93 (m, 1H), 3.84 – 3.71 (m, 2H), 3.69 (s, 6H), 3.45 (bs, 2H), 3.08 (dt, *J* = 6.8, 1.0 Hz, 2H), 2.81 (dd, *J* = 12.0, 2.8 Hz, 1H), 2.24 (t, *J* = 7.2 Hz, 2H), 2.16 – 2.06 (m, 2H), 2.00 – 1.94 (m, 1H), 1.81 – 1.62 (m, 6H), 1.52 – 1.40 (m, 4H), 1.40 – 1.11 (m, 5H), 1.26 (s, 3H), 1.25 (s, 3H), 1.11 (s, 3H), 1.03 – 0.85 (m, 2H), 0.95 (s, 3H). ^{13}**C NMR** (126 MHz, CD_3OD) δ 175.8, 159.6, 150.1, 143.0, 139.9, 131.3, 128.5, 127.2, 123.8, 119.0, 104.7, 58.0, 56.2, 52.3, 45.5, 44.9, 42.8, 41.8, 38.7, 37.1, 30.1, 29.8, 29.5, 29.4, 28.6, 28.4, 27.8, 27.3, 27.1, 26.8, 24.0, 21.4. **IR** (neat, ν_{max}/cm^{-1}) 3319, 2924, 2853, 2095, 1579, 1300, 1121. **HRMS (ESI)**: *m/z* = 821.4697 $[M+H]^+$ (calc. for $C_{46}H_{61}N_8O_6$ *m/z* = 821.4709).

Synthesis of (*R*)-SI-4

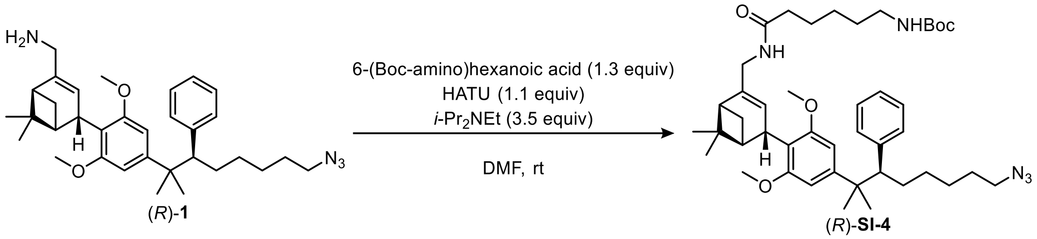

To a solution of 6-(Boc-amino)hexanoic acid (9.9 mg, 42.6 µmol, 1.3 equiv) in DMF (0.1 mL) was added HATU (13.7 mg, 36.0 µmol, 1.1 equiv) and *i*-Pr_2NEt (20 µL, 115 µmol, 3.5 equiv) and the reaction mixture was stirred at 0 °C for 5 min before being added to **(*R*)-1** (17.4 mg, 32.7 µmol, 1.0 equiv) in DMF (0.1 mL). The reaction mixture was stirred at rt for 1 h, diluted with EtOAc (15 mL) and quenched with water (10 mL). The phases were separated and the aqueous layer extracted with EtOAc (2 × 15 mL). The combined organic extracts were dried with $MgSO_4$, filtered and concentrated *in vacuo*. The crude material was purified by flash column chromatography (SiO_2; 0 – 15% (3 × CV), 15% – 25% (5 × CV), 25% (5 × CV), 25% – 40% (5 × CV) using (EtOAc:EtOH 3:1) in hexanes) to afford the title product (*R*)-**SI-4** as light yellow waxy solid (21.0 mg, 86%).

^{1}H NMR (400 MHz, $CDCl_3$) δ 7.25 – 7.14 (m, 3H), 7.08 – 6.99 (m, 2H), 6.42 (s, 2H), 5.63 (dt, *J* = 2.9, 1.4 Hz, 1H), 5.34 (t, *J* = 5.5 Hz, 1H), 4.53 (s, 1H), 4.01 – 3.94 (m, 1H), 3.94 – 3.80 (m, 2H), 3.70 (s, 6H), 3.10 (t, *J* = 6.9 Hz, 4H), 2.72 (dd, *J* = 12.1, 2.9 Hz, 1H), 2.24 – 2.14 (m, 3H), 2.13 – 2.01 (m, 2H), 1.74 – 1.57 (m, 5H), 1.55 – 1.11 (m, 8H), 1.44 (s, 9H), 1.29 (s, 3H), 1.27 (s, 3H), 1.14 (s, 3H), 1.02 – 0.87 (m, 2H), 0.96 (s, 3H). **^{13}C NMR** (101 MHz, $CDCl_3$) δ 172.7, 158.3, 156.1, 148.6, 141.7, 138.6, 130.1, 127.5, 126.3, 124.0, 118.0, 103.9, 79.2, 57.2, 56.0, 51.5, 47.5, 44.6, 44.5, 41.8, 40.9, 40.5, 37.6, 36.9, 30.0, 29.3, 28.9, 28.7, 28.6, 27.9, 27.9, 26.7, 26.6, 26.4, 25.6, 23.9, 21.2. **IR** (neat, ν_{max}/cm^{-1}) 3323, 2932, 2864, 2095, 1698, 1650, 1604, 1572, 1518, 1452, 1240, 1121. **HRMS (ESI)**: *m/z* = 744.5057 $[M+H]^+$ (calc. for $C_{44}H_{66}N_5O_5$ *m/z* = 744.5058).

Synthesis of (*R*)-5

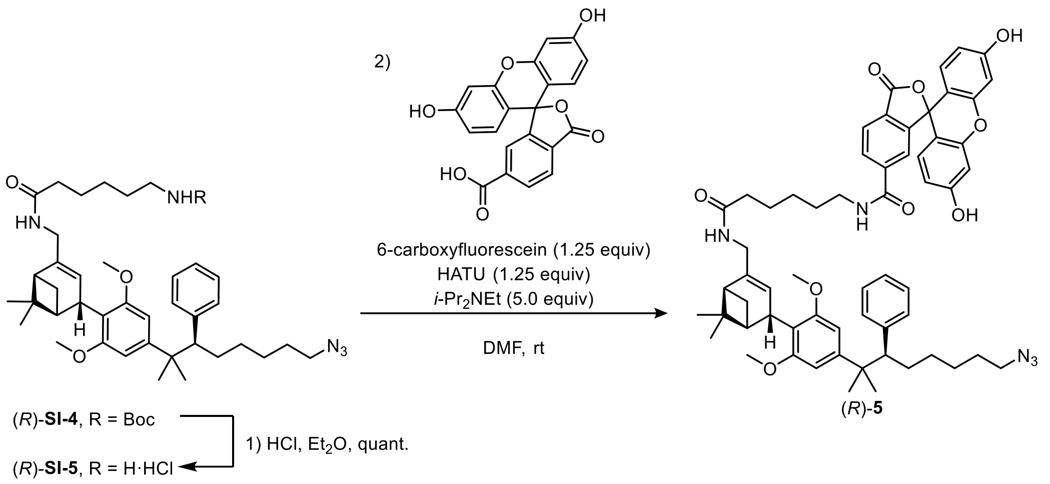

1) To a solution of (*R*)-**SI-4** (3.0 mg, 4.0 µmol, 1.0 equiv) in Et_2O (50 µL) was added HCl (2.0 M in Et_2O, 50 µL) and the solution was stirred for 1 h at rt. The mixture was concentrated *in vacuo* and the hydrochloride salt (*R*)-**SI-5** used immediately in the next step without further purification. 2) To 6-carboxyfluorescein (1.9 mg, 5.0 µmol, 1.25 equiv) was added *i*-Pr_2NEt (3.5 µL, 20 µmol, 5.0 equiv) and anhydrous DMF (20 µL) and the mixture was stirred for 5 min. Subsequently a solution of the hydrochloride (*R*)-**SI-5** (2.7 mg, 4.0 µmol, 1.0 equiv) in anhydrous DMF (50 µL) and HATU (1.9 mg, 5.0 µmol, 1.25 equiv) were added and the solution was stirred for 1 h at rt. The mixture was concentrated *in vacuo* and purified by preparative TLC (SiO_2; 1% AcOH, 7% MeOH in CH_2Cl_2) and further by preparative reverse-phase HPLC (Dr. Maisch Reprosil Gold 120 C4 150 × 20 mm, flow 26.5 mL/min, gradient of 60% – 90% acetonitrile (+ 0.1% HCOOH) in water (+ 0.1% HCOOH) over 30 min, column temperature 25 °C) to yield the title compound (*R*)-**5** as a yellow lyophilized powder (1.5 mg, 37%).

HRMS (ESI): *m/z* = 1002.5012 $[M+H]^+$ (calc. for $C_{60}H_{68}N_5O_9$ *m/z* = 1002.5012). **Preparative reverse-phase HPLC**: (Dr. Maisch Reprosil Gold 120 C4 150 × 20 mm, flow 26.5 mL/min, column temperature 25 °C, H_2O (+ 0.1% HCOOH) : MeCN (+ 0.1% HCOOH) = 40:60 (t = 0.0 min) → 40:60 (t = 1.0 min) → 10:90 (t = 24.0 min) → 10:90 (t = 26.0 min) → 40:60 (t = 30.0 min), t_R = 10.81 min. **Analytical UPLC**: (Acquity UPLC® BEH C18 1.7 µm 2.1 × 50 mm, H_2O (+ 0.1% HCOOH) : MeCN (+ 0.1% HCOOH) = 98:2 (t = 0.0 min) → 98:2 (t = 0.5 min) → 2:98 (t = 4.0 min) → 2:98 (t = 5.0 min) → 98:2 (t = 6.5 min), t_R = 4.70 min.

Synthesis of (*R*)-6

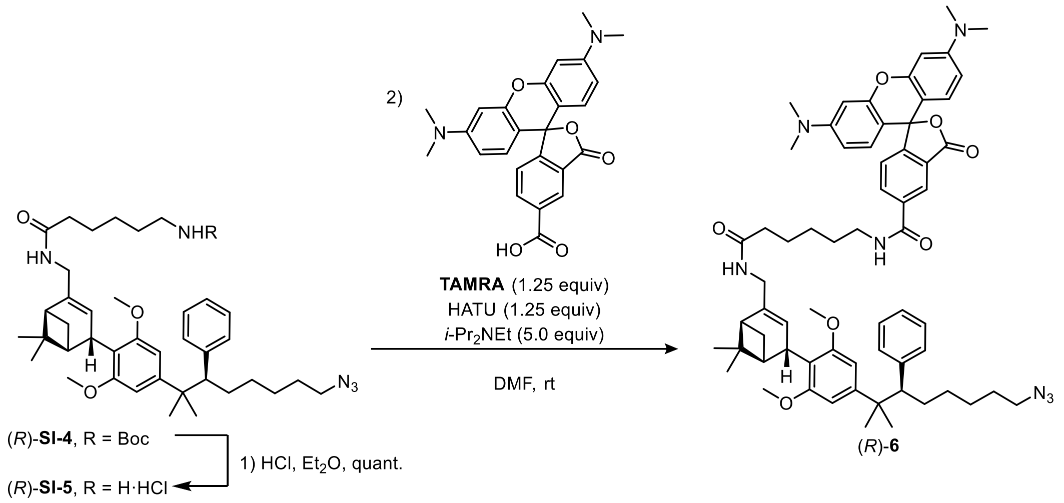

1) To a solution of **(*R*)-SI-4** (3.0 mg, 4.0 µmol, 1.0 equiv) in Et_2O (50 µL) was added HCl (2.0 M in Et_2O, 50 µL) and the solution was stirred for 1 h at rt. The mixture was concentrated *in vacuo* and the hydrochloride salt **(*R*)-SI-5** used immediately in the next step without further purification. 2) To **TAMRA** (2.2 mg, 5.0 µmol, 1.25 equiv) was added *i*-Pr_2NEt (3.5 µL, 20 µmol, 5.0 equiv) and anhydrous DMF (20 µL) and the mixture was stirred for 1 min. Subsequently a solution of the hydrochloride **(*R*)-SI-5** (2.7 mg, 4.0 µmol, 1.0 equiv) in anhydrous DMF (50 µL) and HATU (1.9 mg, 5.0 µmol, 1.25 equiv) were added and the solution was stirred for 1 h at rt. The mixture was concentrated *in vacuo* and purified by Preparative reverse-phase HPLC (Dr. Maisch Reprosil Gold 120 C4 150 × 20 mm, flow 26.5 mL min^{-1}, gradient of 50% – 90% acetonitrile (+ 0.1% HCOOH) in water (+ 0.1% HCOOH) over 30 min, column temperature 25 °C) to yield the title compound **(*R*)-6** as a red lyophilized powder (1.8 mg, 43%).

HRMS (ESI): *m/z* = 1056.5958 $[M+H]^+$ (calc. for $C_{64}H_{78}N_7O_7$ *m/z* = 1056.5957). **Preparative reverse-phase HPLC**: (Dr. Maisch Reprosil Gold 120 C4 150 × 20 mm, flow 26.5 mL/min, column temperature 25 °C, H_2O (+ 0.1% HCOOH) : MeCN (+ 0.1% HCOOH) = 50:50 (t = 0.0 min) → 50:50 (t = 1.0 min) → 10:90 (t = 24.0 min) → 10:90 (t = 26.0 min) → 50:50 (t = 30.0 min), t_R = 9.20 min. **Analytical UPLC**: (Acquity UPLC® BEH C18 1.7 µm 2.1 × 50 mm, H_2O (+ 0.1% HCOOH) : MeCN (+ 0.1% HCOOH) = 98:2 (t = 0.0 min) → 98:2 (t = 0.5 min) → 2:98 (t = 4.0 min) → 2:98 (t = 5.0 min) → 98:2 (t = 6.5 min), t_R = 4.18 min.

Synthesis of (*R*)-7

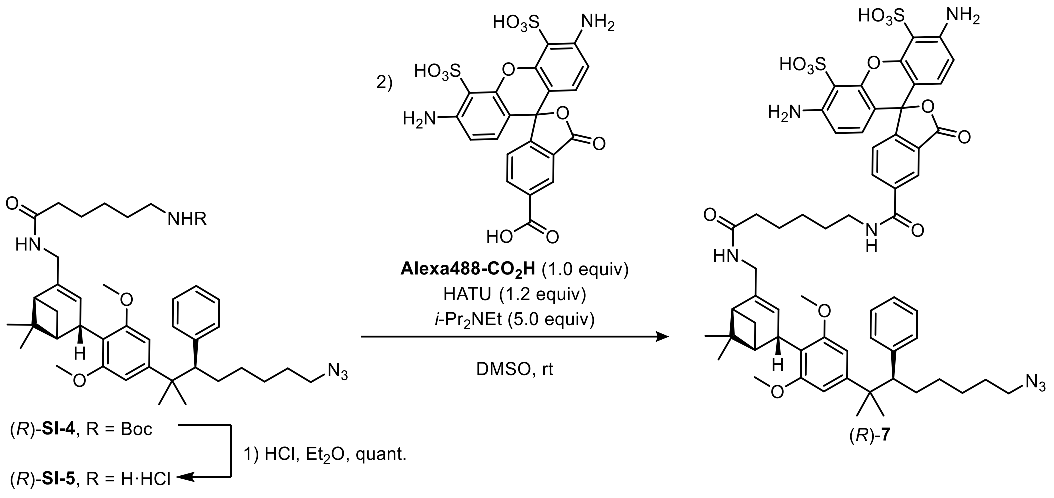

1) To a solution of (*R*)-**SI-4** (9.0 mg, 12.1 µmol, 1.3 equiv) in Et_2O (100 µL) was added HCl (2.0 M in Et_2O, 100 µL) and the solution was stirred for 1 h at rt. The mixture was concentrated *in vacuo* and the hydrochloride salt (*R*)-**SI-5** used immediately in the next step without further purification.

2) To **Alexa488-CO₂H** (5.0 mg, 9.3 µmol, 1.0 equiv) was added a solution of the hydrochloride (*R*)-**SI-5** (8.3 mg, 12.1 µmol, 1.3 equiv) in anhydrous DMSO (200 µL), followed by *i*-Pr_2NEt (8.2 µL, 47 µmol, 5.0 equiv) and HATU (4.3 mg, 11.2 µmol, 1.2 equiv) and the deep red solution was stirred at rt for 1 h. The mixture was concentrated *in vacuo* and directly purified by preparative TLC (SiO_2, 40% *i*-PrOH, 20% H_2O in EtOAc) and a second time by Preparative reverse-phase HPLC (Dr. Maisch Reprosil Gold 120 C4 150 × 20 mm, flow 26.5 mLmin^{-1}, gradient of 50% – 90% acetonitrile (+ 0.1% HCOOH) in water (+ 0.1% HCOOH) over 30 min, column temperature 25 °C) to yield the title compound (*R*)-**7** as a red lyophilized powder (2.7 mg, 25%).

^{1}H NMR (600 MHz, $(CD_3)_2SO$) δ 8.79 (s, 1H), 8.44 (bs, 1H), 8.24 (d, *J* = 7.9 Hz, 1H), 7.80 (t, *J* = 6.1 Hz, 1H), 7.34 (bs, 1H), 7.27 – 7.22 (m, 2H), 7.21 – 7.13 (m, 3H), 6.81 (bs, 2H), 6.54 (s, 2H), 6.42 (bs, 2H), 5.45 (dt, *J* = 3.3, 1.6 Hz, 1H), 3.89 – 3.83 (m, 1H), 3.74 – 3.69 (m, 1H), 3.68 (s, 6H), 3.62 (dd, *J* = 15.2, 5.8 Hz, 1H), 3.51 (t, *J* = 1.2 Hz, 2H), 3.12 (td, *J* = 6.8, 1.7 Hz, 2H), 2.87 (dd, *J* = 11.9, 2.7 Hz, 1H), 2.15 – 2.07 (m, 3H), 2.07 – 2.01 (m, 1H), 1.93 (t, *J* = 5.6 Hz, 1H), 1.63 (d, *J* = 8.1 Hz, 1H), 1.60 – 1.52 (m, 4H), 1.37 – 1.18 (m, 6H), 1.24 (s, 3H), 1.20 (s, 3H), 1.16 – 1.04 (m, 2H), 1.01 (s, 3H), 0.91 (s, 3H), 0.88 – 0.77 (m, 2H). **^{13}C NMR** (151 MHz, $(CD_3)_2SO$) δ 174.5, 171.8, 164.5, 157.8, 148.8, 141.5, 139.3, 136.1, 129.9, 127.4, 126.0, 121.1, 117.1, 103.6, 69.8, 55.9, 55.3, 50.5, 47.0, 43.4, 42.8, 41.3, 40.4, 40.2, 36.9, 35.5, 29.1, 28.9, 28.3, 27.9, 27.2,

27.1, 26.2, 26.2, 25.6, 25.3, 22.6, 20.8. **IR** (neat, ν_{max}/cm^{-1}) 3410, 3304, 3192, 2922, 2852, 2096, 1737, 1598, 1556, 1424, 1242. **HRMS (ESI)**: *m/z* = 1182.4283 [M+Na]$^+$ (calc. for $C_{60}H_{69}N_7NaO_{13}S_2$ *m/z* = 1182.4287). **Preparative reverse-phase HPLC** (Dr. Maisch Reprosil 100 C4, 5 µm, 150 × 20 mm, flow 26.5 mL/min, column temperature 25 °C, H_2O (+ 0.1% HCOOH) : MeCN (+ 0.1% HCOOH) = 50:50 (t = 0.0 min) → 50:50 (t = 1.0 min) → 10:90 (t = 24.0 min) → 10:90 (t = 26.0 min) → 50:50 (t = 30.0 min), t_R = 9.60 min. **Analytical HPLC** (Dr. Maisch Reprosil 100 C4, 5 µm, 150 × 4.6 mm, flow 1.4 mL/min, column temperature 25 °C, H_2O (+ 0.1% HCOOH) : MeCN (+ 0.1% HCOOH) = 50:50 (t = 0.0 min) → 50:50 (t = 1.0 min) → 10:90 (t = 12.0 min) → 10:90 (t = 14.0 min) → 50:50 (t = 15.0 min), t_R = 8.22 min.

Synthesis of (*R*)-8

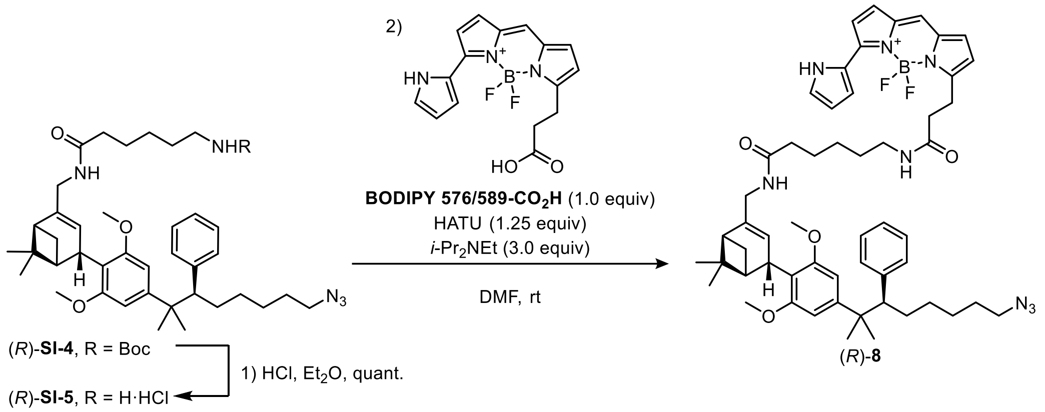

1) To a solution of **(*R*)-SI-4** (7.3 mg, 9.8 µmol, 1.2 equiv) in Et_2O (100 µL) was added HCl (2.0 M in Et_2O, 100 µL) and the solution was stirred for 1 h at rt. The mixture was concentrated *in vacuo* and the hydrochloride salt **(*R*)-SI-5** used immediately in the next step without further purification.

2) To **BODIPY 576/589-CO$_2$H** (2.7 mg, 8.2 µmol, 1.0 equiv) was added a solution of the hydrochloride **(*R*)-SI-5** (6.3 mg, 9.8 µmol, 1.2 equiv) in anhydrous DMF (100 µL), followed by *i*-Pr_2NEt (7.2 µL, 41 µmol, 5.0 equiv) and HATU (3.9 mg, 10 µmol, 1.25 equiv), and the deep purple solution was stirred at rt for 45 min. The mixture was concentrated *in vacuo* and directly purified by preparative TLC (SiO_2; 2% MeOH in CH_2Cl_2) and a second time (SiO_2; 7.5% EtOH, 22.5% EtOAc in hexanes) to yield the product **(*R*)-8** as a dark purple solid (4.7 mg, 60%).

^{1}H NMR (500 MHz, CD_2Cl_2) δ 10.38 (s, 1H), 7.27 – 7.21 (m, 2H), 7.20 – 7.16 (m, 2H), 7.11 – 7.06 (m, 3H), 7.05 (s, 1H), 7.01 (ddd, *J* = 3.9, 2.5, 1.4 Hz, 1H), 6.90 (d, *J* = 4.6 Hz, 1H), 6.89 (d, *J* = 4.0 Hz, 1H), 6.46 (s, 2H), 6.41 – 6.36 (m, 1H), 6.31 (d, *J* = 4.0 Hz, 1H), 5.70 (t, *J* = 5.5 Hz, 1H), 5.59 (dt, *J* = 2.9, 1.5 Hz, 1H), 5.39 (t, *J* = 5.7 Hz, 1H), 3.99 – 3.91 (m, 1H), 3.89 – 3.75 (m, 2H), 3.69 (s, 6H), 3.28 (t, *J* = 7.7 Hz, 2H), 3.21 (td, *J* = 7.0, 5.8 Hz, 2H), 3.10 (t, *J* = 6.9 Hz, 2H), 2.77 (dd, *J* = 11.9, 2.7 Hz, 1H), 2.59 (dd, *J* = 8.2, 7.1 Hz, 2H), 2.20 – 2.11 (m, 3H), 2.08 (td, *J* = 5.6, 1.4 Hz, 1H), 2.02 (tt, *J* = 5.8, 1.9 Hz, 1H), 1.70 – 1.20 (m, 12H), 1.67 (d, *J* = 8.3 Hz, 1H), 1.28 (s, 3H), 1.26 (s, 3H), 1.11 (s, 3H), 1.00 – 0.91 (m, 2H). 0.95 (s, 3H). **^{13}C NMR** (126 MHz, CD_2Cl_2) δ 172.8, 171.6, 158.8, 156.5, 150.8, 149.4, 142.3, 139.3, 137.8, 134.1, 132.2, 130.6, 127.9, 127.2, 126.6, 126.2, 124.1, 123.9, 120.8, 118.4, 118.2, 117.3, 112.0, 104.3, 57.4, 56.3, 52.0, 48.0, 44.9, 44.7, 42.2, 41.3, 39.7, 38.1, 37.1, 36.0, 29.8, 29.2, 29.1, 28.3, 28.1, 27.1, 26.9, 26.6, 25.8, 25.2, 24.0, 21.3. **^{19}F NMR** (471 MHz, CD_2Cl_2) δ -140.4 (q, *J* = 36.0 Hz). **^{11}B NMR** (160 MHz,

CD_2Cl_2) δ 1.54 (t, *J* = 36.3 Hz). **IR** (neat, ν_{max}/cm^{-1}) 3301, 2926, 2855, 2094, 1648, 1604, 1574, 1484, 1462, 1107. **HRMS (ESI)**: *m/z* = 955.5567 $[M+H]^+$ (calc. for $C_{55}H_{70}BF_2N_8O_4$ *m/z* = 955.5576).

Synthesis of (*R*)-9

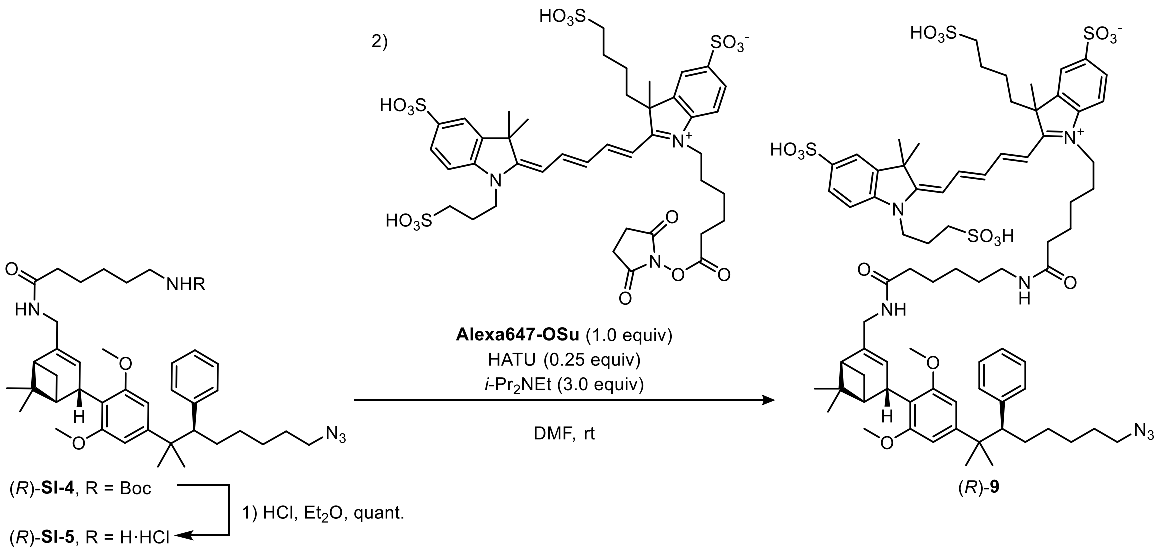

1) To a solution of (*R*)-**SI-4** (7.0 mg, 9.4 µmol, 1.8 equiv) in Et_2O (100 µL) was added HCl (2.0 M in Et_2O, 100 µL) and the solution was stirred for 1 h at rt. The mixture was concentrated *in vacuo* and the hydrochloride salt (*R*)-**SI-5** used immediately in the next step without further purification.

2) To **Alexa647-OSu** (5.0 mg, 5.2 µmol, 1.0 equiv) was added a solution of the hydrochloride (*R*)-**SI-5** (6.5 mg, 9.4 µmol, 1.8 equiv) in anhydrous DMF (100 µL) and *i*-Pr_2NEt (2.7 µL, 16 µmol, 3.0 equiv) and the deep blue solution was stirred at rt for 16 h. To reach full conversion of the hydrolyzed OSu-ester, HATU (0.5 mg, 1.3 µmol, 0.25 equiv) and *i*-Pr_2NEt (1.0 µL, 5.7 µmol, 1.1 equiv) were added and the solution was stirred for additional 30 min upon which full conversion was confirmed. The mixture was concentrated *in vacuo* and directly purified by preparative TLC (SiO_2, 20% H_2O, 40% *i*-PrOH in EtOAc) and a second time (8 – 15% MeOH in CH_2Cl_2) to remove the coeluting HOAt contaminant. The product (*R*)-**9** was furnished as a deep blue wax (5.2 mg, 70%).

^{1}H NMR (500 MHz, CD_3OD) δ 8.55 (s, 1H), 8.41 – 8.28 (m, 2H), 7.92 – 7.87 (m, 4H), 7.42 (d, *J* = 8.3 Hz, 1H), 7.33 (d, *J* = 8.3 Hz, 1H), 7.25 – 7.20 (m, 2H), 7.20 – 7.14 (m, 1H), 7.13 – 7.08 (m, 2H), 6.73 (t, *J* = 12.4 Hz, 1H), 6.54 – 6.52 (m, 1H), 6.52 (s, 2H), 6.37 (d, *J* = 13.8 Hz, 1H), 5.60 – 5.57 (m, 1H), 4.35 (t, *J* = 8.1 Hz, 2H), 4.13 (t, *J* = 7.4 Hz, 2H), 3.99 – 3.96 (m, 1H), 3.84 – 3.71 (m, 2H), 3.69 (s, 6H), 3.66 – 3.63 (m, 2H), 3.16 – 3.06 (m, 4H), 3.00 (t, *J* = 6.6 Hz, 2H), 2.83 (dd, *J* = 12.0, 2.5 Hz, 1H), 2.65 – 2.60 (m, 2H), 2.23 (t, *J* = 7.4 Hz, 4H), 2.20 – 2.12 (m, 5H), 2.09 (t, *J* = 5.6 Hz, 1H), 2.03 – 1.96 (m, 1H), 1.86 – 1.80 (m, 1H), 1.77 (s, 3H), 1.76 (s, 3H), 1.73 (s, 3H),

1.72 – 1.41 (m, 8H), 1.39 – 1.15 (m, 12H), 1.29 (s, 3H), 1.28 (s, 3H), 1.12 (s, 3H), 1.04 – 0.97 (m, 2H), 0.95 (s, 3H). **^{13}C NMR** (126 MHz, CD_3OD) δ 175.8, 170.3, 159.6, 155.2 (HSQC), 131.3, 128.5, 128.3, 127.2, 123.9, 121.4, 111.4 (HSQC), 111.1 (HSQC), 105.6 (HSQC), 104.8, 71.3, 58.0, 56.3, 52.3, 52.1 (HSQC), 48.9 (HSQC), 48.6 (HSQC), 45.5, 44.9, 44.7 (HSQC), 43.7 (HSQC), 42.8, 41.8, 40.2, 39.4, 38.7, 37.1, 36.8, 33.1, 30.8, 30.5, 30.1, 29.5, 28.6, 28.4, 28.2, 27.9, 27.6, 27.3, 26.9, 26.6, 26.0, 24.0, 23.7, 21.4. **IR** (neat, ν_{max}/cm^{-1}) 3424, 2923, 2853, 2095, 1642, 1602, 1572, 1492, 1464, 1382, 1330, 1180. **HRMS (ESI)**: *m/z* = 1520.6240 [M+Na]$^+$ (calc. for $C_{76}H_{103}N_7NaO_{16}S_4$ *m/z* = 1520.6236).

Synthesis of (*S*)-SI-6

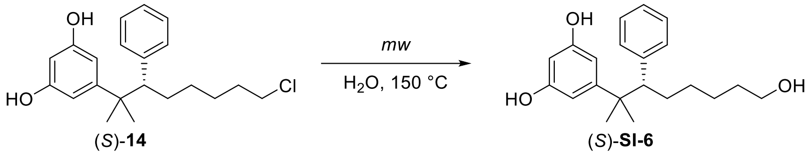

A suspension of (*S*)-**14** (32.0 mg, 92.2 μmol, 1.0 equiv) in H_2O (2.0 mL) was heated at 150 °C under microwave irradiation for 3 h. The vial was washed into a separatory funnel with EtOAc (10 mL) and CH_2Cl_2 (10 mL). The phases were separated, and the aqueous phase was extracted with CH_2Cl_2 (2 × 10 mL). The combined organic layers were dried over Na_2SO_4, filtered, and concentrated *in vacuo* to afford (*S*)-**SI-6** (30.1 mg, 91.6 μmol, 99%) as an off-white foam.

^{1}H NMR (400 MHz, $CDCl_3$) δ 7.25 – 7.16 (m, 3H), 7.15 – 7.08 (m, 2H), 6.48 (d, J = 2.0 Hz, 2H), 6.25 (t, J = 2.1 Hz, 2H), 3.52 (tdd, J = 10.5, 6.6, 4.0 Hz, 2H), 2.80 (d, J = 12.0 Hz, 1H), 1.69 – 1.52 (m, 1H), 1.40 – 1.09 (m, 10H), 0.99 (s, 3H), 0.96 – 0.81 (m, 2H). **^{13}C NMR** (101 MHz, $CDCl_3$) δ 156.5, 153.5, 141.6, 130.3, 127.7, 126.3, 106.7, 100.5, 63.3, 55.8, 41.6, 31.8, 30.1, 28.5, 27.2, 24.7, 22.2. **IR** (neat, ν_{max}/cm^{-1}): 3307, 2935, 2488, 1705, 1598, 1436, 1328, 1153. **HRMS (ESI)**: *m/z* = 351.1928 $[M+Na]^+$ (calc. for $C_{21}H_{28}NaO_3$ *m/z* = 351.1931). **$[\alpha]^{25}_D$** = –32.414 ± 0.253 (c = 0.5, $CHCl_3$).

Synthesis of (*S*)-17

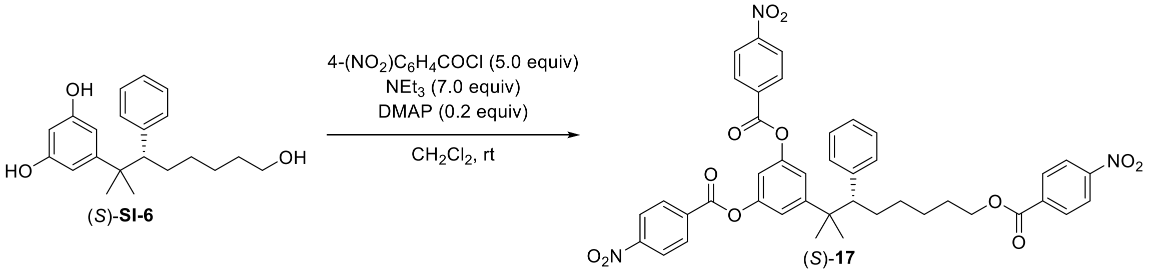

To a solution of (*S*)-**SI-6** (17.5 mg, 53.3 µmol, 1.0 equiv), NEt_3 (37.7 mg, 52.0 µL, 373.0 µmol, 7.0 equiv) and DMAP (1.3 mg, 10.7 µmol, 0.2 equiv) in dry CH_2Cl_2 (0.8 mL) was added in one portion solid 4-nitrobenzoyl chloride (49.4 mg, 266.0 µmol, 5.0 equiv). The yellow reaction mixture was allowed to stir for 14 h at rt before being loaded directly onto silica. Purification by flash column chromatography (SiO_2; 15% EtOAc in hexanes) afforded (*S*)-**17** (35.0 mg, 45.1 µmol, 85% yield) as a white solid. Slow evaporation from a solution of cyclohexane, EtOAc, Et_2O afforded needle crystals suitable for X-ray crystallography.

^{1}H NMR (500 MHz, $CDCl_3$) δ 8.41 – 8.35 (m, 8H), 8.27 – 8.23 (m, 2H), 8.15 – 8.12 (m, 2H), 7.28 – 7.17 (m, 3H), 7.13 (d, J = 2.1 Hz, 2H), 7.10 – 7.06 (m, 3H), 4.24 (t, J = 6.7 Hz, 2H), 2.80 (dd, J = 12.1, 2.8 Hz, 1H), 1.78 – 1.69 (m, 1H), 1.66 – 1.58 (m, 2H), 1.54 – 1.46 (m, 1H), 1.40 – 1.31 (m, 4H), 1.30 – 1.23 (m, 1H), 1.20 (s, 3H), 1.13 – 0.98 (m, 2H). **^{13}C NMR** (126 MHz, $CDCl_3$) δ 164.8, 163.2, 153.1, 151.2, 150.7, 140.9, 136.0, 134.8, 131.5, 130.8, 130.0, 127.9, 126.6, 124.0, 123.6, 118.1, 112.8, 66.1, 57.3, 42.1, 29.4, 28.7, 28.5, 28.0, 26.0, 23.8. **IR** (neat, ν_{max}/cm^{-1}): 3112, 2937, 2862, 1744, 1722, 1608, 1526, 1467, 1432, 1410, 1348, 1319, 1256, 1171, 1127, 1104, 1073, 1014, 959, 906, 872, 855, 839, 779, 714. **HRMS (ESI)**: *m/z* = 798.2270 $[M+Na]^+$ (calc. for $C_{42}H_{37}N_3NaO_{12}$ *m/z* = 798.2269).

Synthesis of ago-3

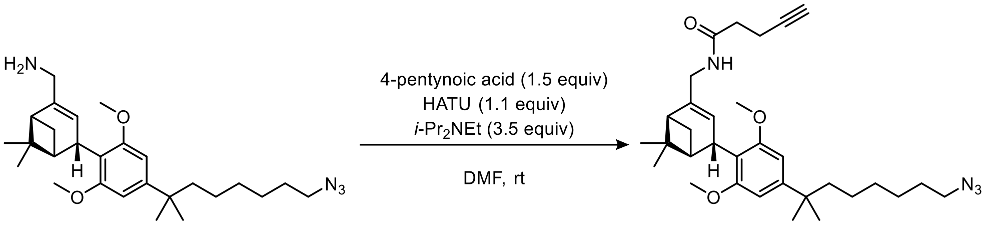

To a solution of 4-pentynoic acid (1.0 mg, 10.1 µmol, 1.5 equiv) in DMF (0.1 mL) was added HATU (2.9 mg, 7.5 µmol, 1.1 equiv) and *i*-Pr_2NEt (4 µL, 23.8 µmol, 3.5 equiv) and the reaction mixture was stirred at 0 °C for 5 min before being added to a mixture of **ago-1** (3.1 mg, 6.8 µmol, 1.0 equiv) in DMF (50 µL). The reaction mixture was stirred at rt for 1 h and concentrated *in vacuo*. The crude product was purified by preparative TLC (SiO_2, 35% EtOAc in hexanes) to afford the title product **ago-3** as a colourless waxy solid (3.3 mg, 91%).

^{1}H NMR (500 MHz, $CDCl_3$) δ 6.47 (s, 2H), 5.64 (dt, *J* = 2.9, 1.5 Hz, 1H), 5.52 (t, *J* = 5.7 Hz, 1H), 3.97 (p, *J* = 2.2 Hz, 1H), 3.90 – 3.85 (m, 2H), 3.74 (s, 6H), 3.22 (t, *J* = 6.9 Hz, 2H), 2.55 (td, *J* = 7.0, 2.6 Hz, 2H), 2.42 (t, *J* = 7.1 Hz, 2H), 2.17 (dt, *J* = 8.5, 5.5 Hz, 1H), 2.10 (td, *J* = 5.6, 1.4 Hz, 1H), 2.06 (dt, *J* = 5.7, 1.7 Hz, 1H), 1.99 (t, *J* = 2.6 Hz, 1H), 1.69 (d, *J* = 8.5 Hz, 1H), 1.58 – 1.51 (m, 4H), 1.38 – 1.20 (m, 4H), 1.28 (s, 3H), 1.27 (s, 6H), 1.16 – 1.07 (m, 2H), 0.96 (s, 3H). **^{13}C NMR** (126 MHz, $CDCl_3$) δ 170.8, 158.5, 149.4, 138.3, 124.2, 117.6, 102.8, 83.3, 69.5, 55.9, 51.6, 47.4, 44.8, 44.6, 44.5, 40.9, 38.1, 37.6, 35.8, 30.0, 29.0, 29.0, 28.0, 26.8, 26.4, 24.7, 21.2, 15.1. **IR** (neat, ν_{max}/cm^{-1}) 3311, 2930, 2858, 2097, 1651, 1572, 1411, 1122. **HRMS (ESI)**: *m/z* = 535.3645 $[M+H]^+$ (calc. for $C_{32}H_{47}N_4O_3$ *m/z* = 535.3643).

NMR SPECTRA

^{1}H NMR (400 MHz, $CDCl_3$) of **11**

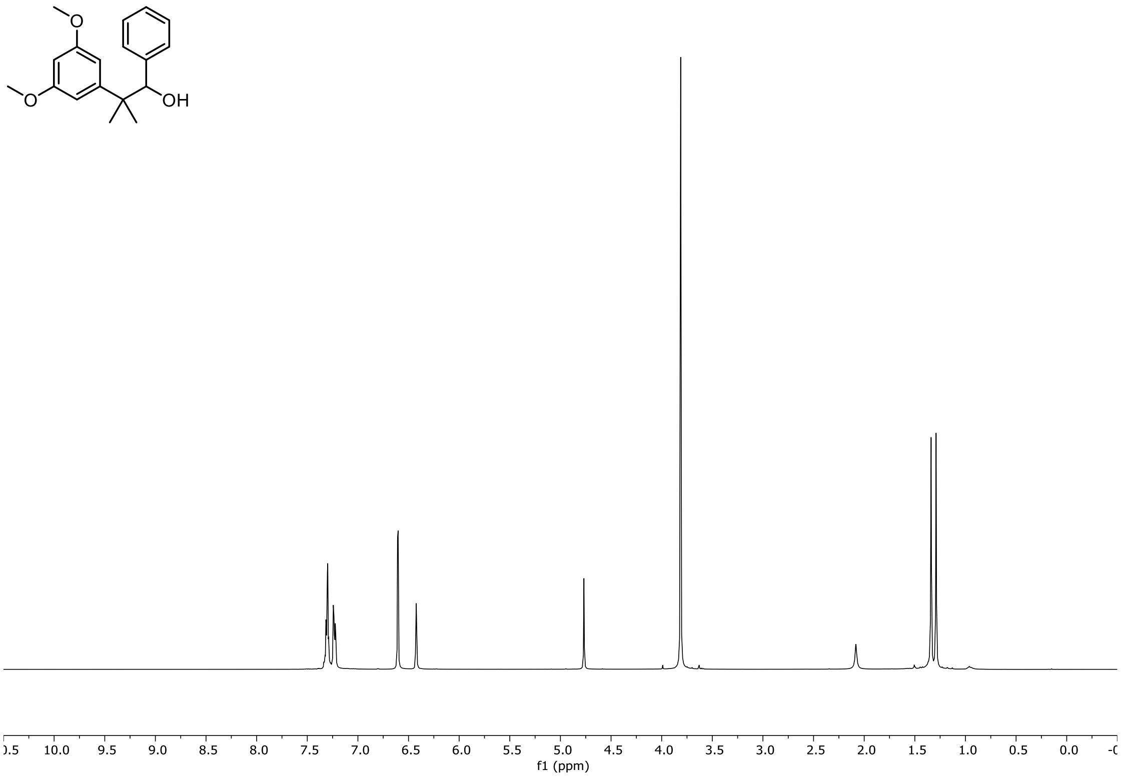

^{13}C NMR (101 MHz, $CDCl_3$) of **11**

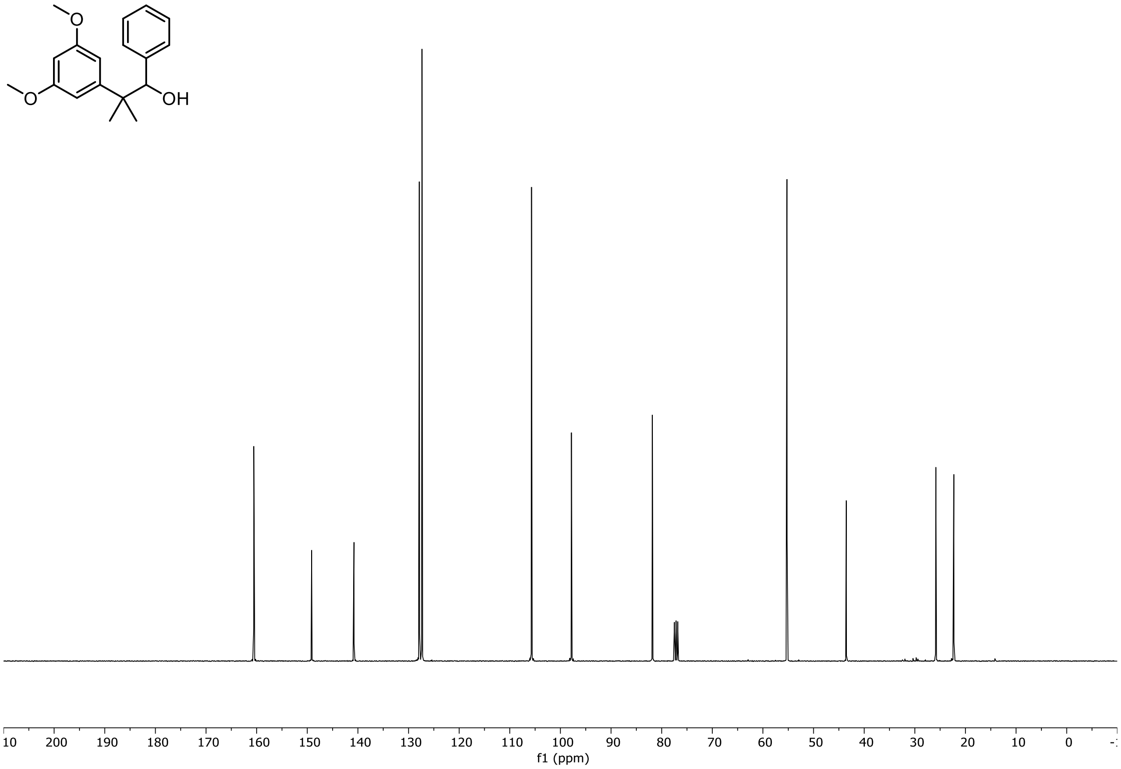

^{1}H NMR (400 MHz, $CDCl_3$) of **12**

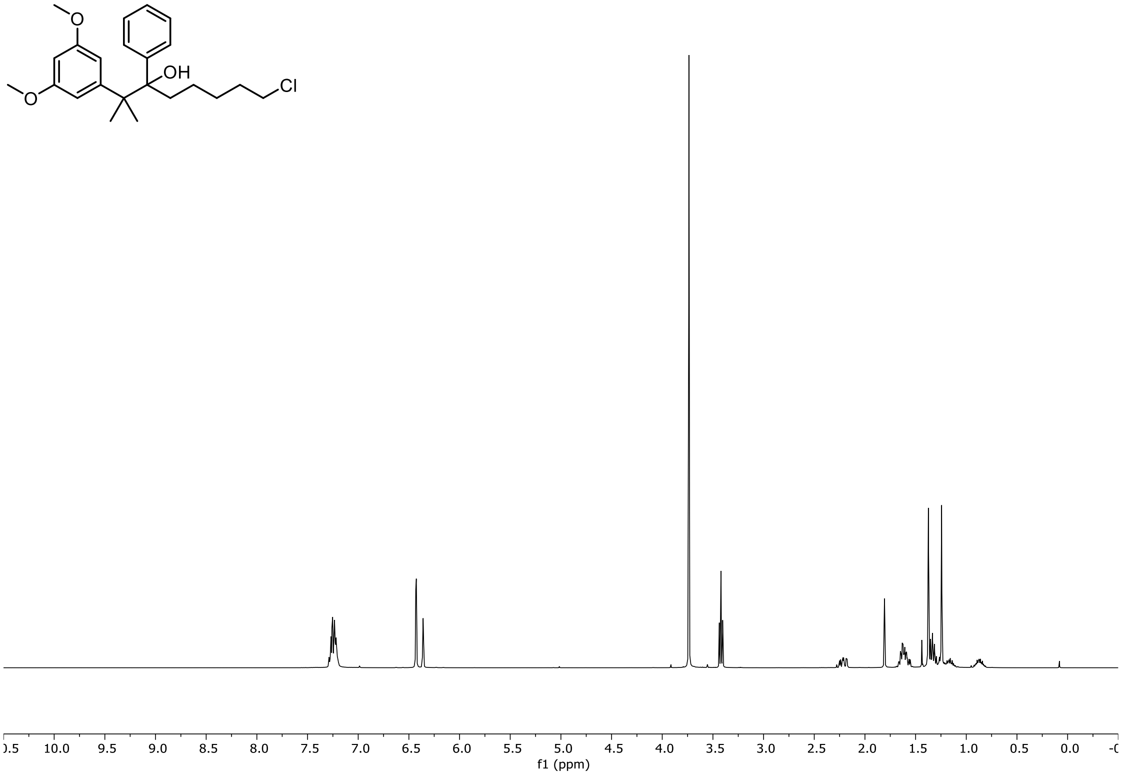

^{13}C NMR (101 MHz, $CDCl_3$) of **12**

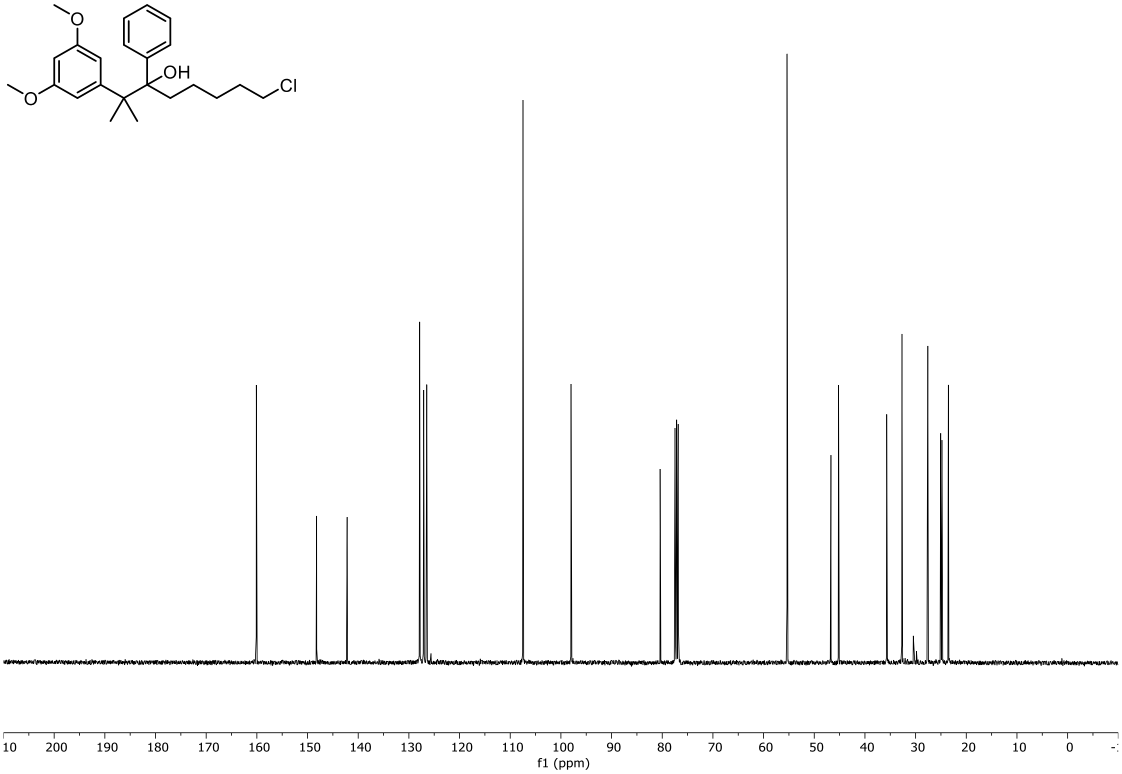

^{1}H NMR (400 MHz, $CDCl_3$) of **13**

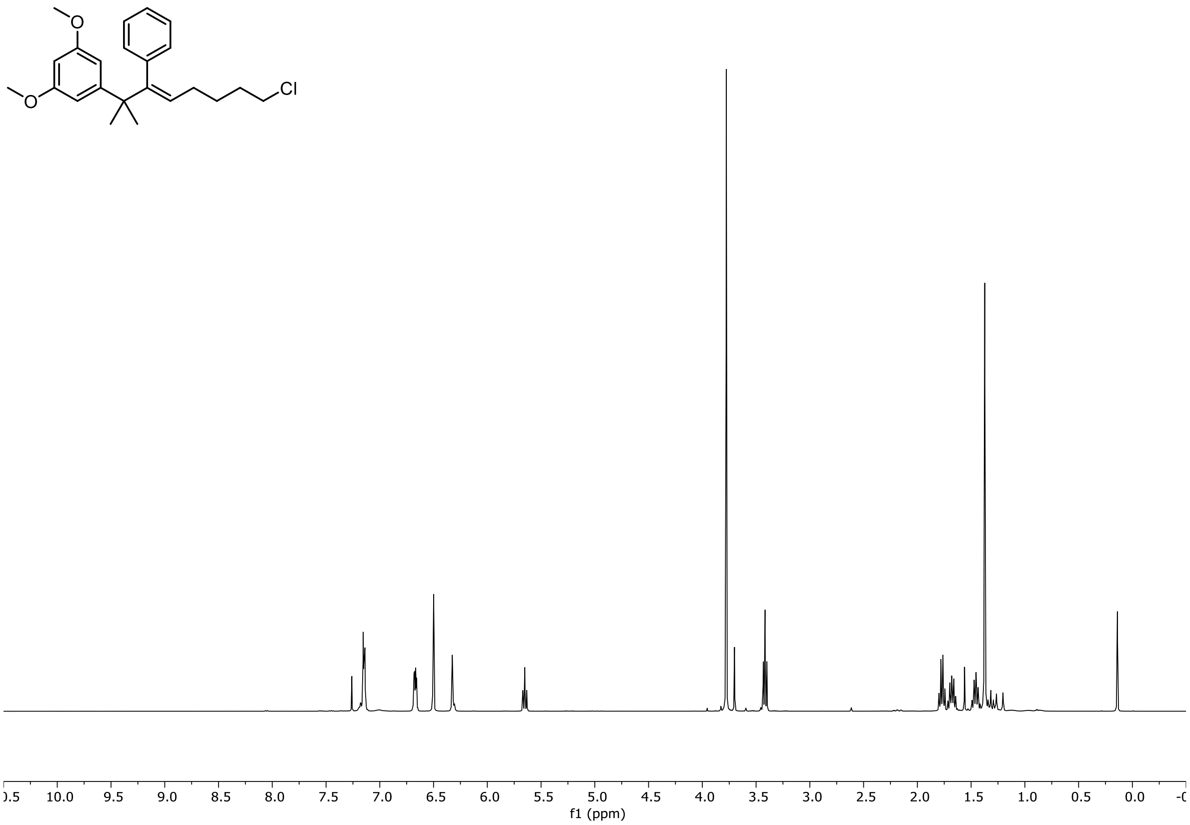

^{13}C NMR (101 MHz, $CDCl_3$) of **13**

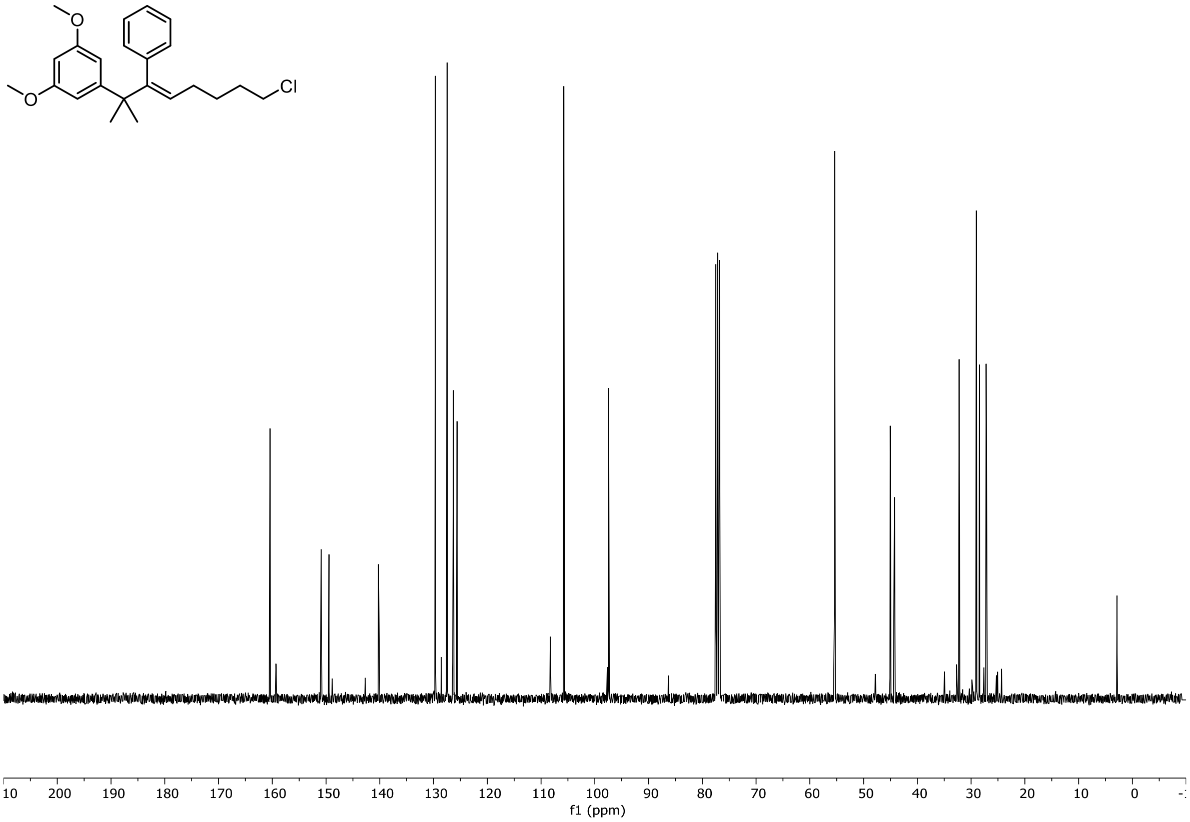

^{1}H NMR (400 MHz, $CDCl_3$) of **SI-1**

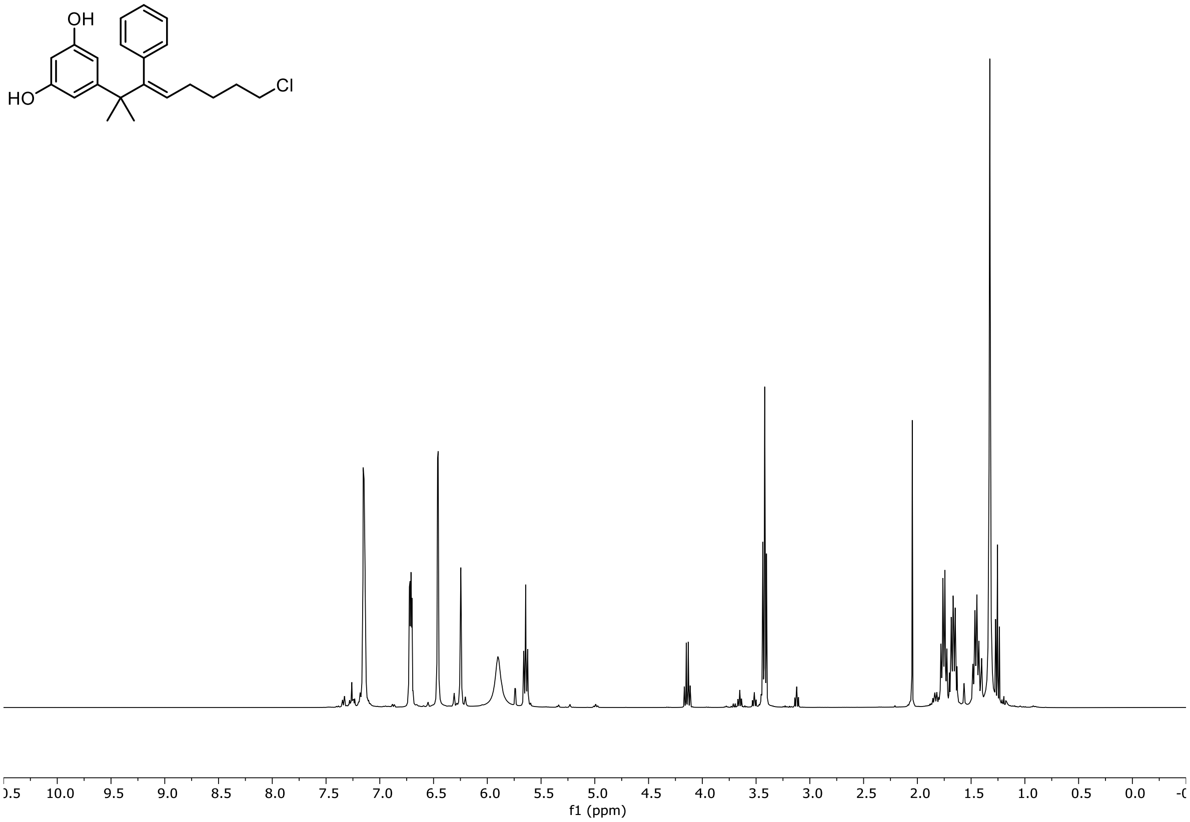

^{13}C NMR (101 MHz, $CDCl_3$) of **SI-1**

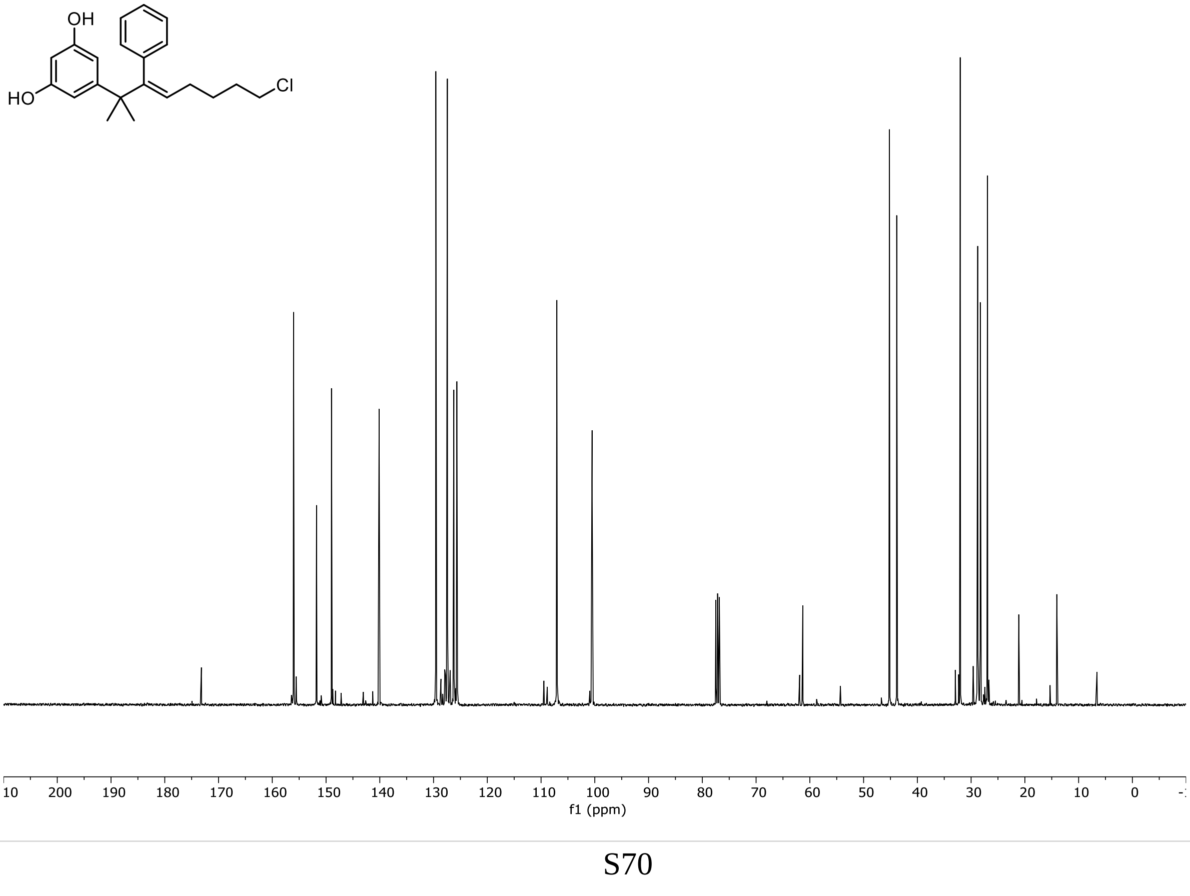

^{1}H NMR (400 MHz, $CDCl_3$) of (*S*)-**14**/(*R*)-**14**

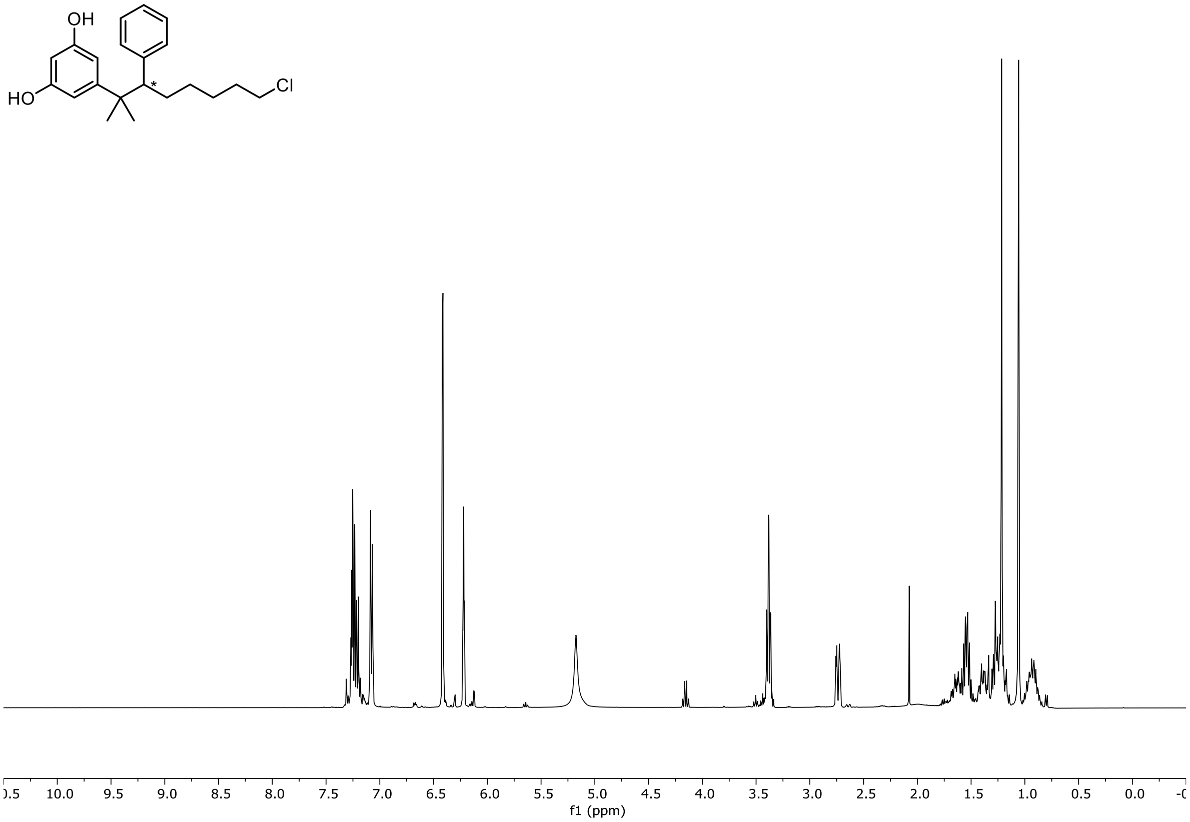

^{13}C NMR (101 MHz, $CDCl_3$) of (*S*)-**14**/(*R*)-**14**

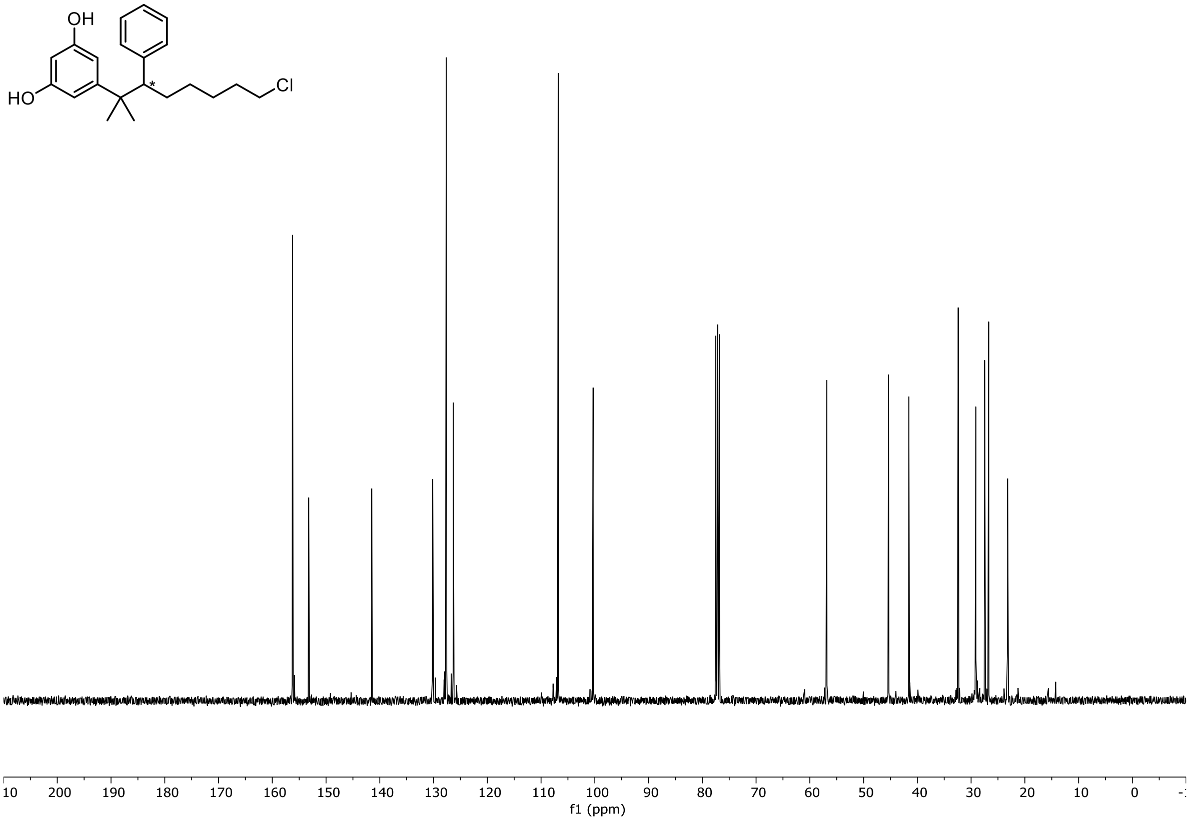

^{1}H NMR (400 MHz, $CDCl_3$) of (*S*)-**SI-2**/(*R*)-**SI-2**

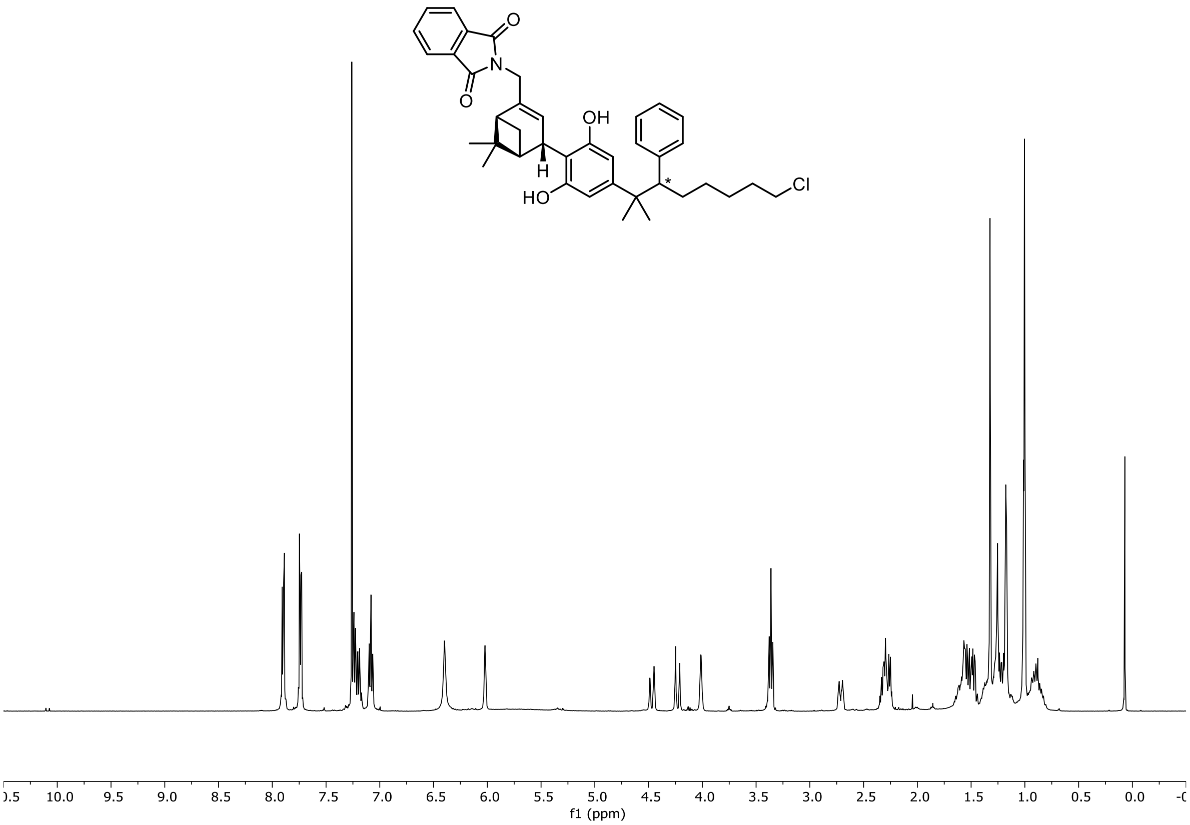

^{13}C NMR (101 MHz, $CDCl_3$) of (*S*)-**SI-2**/(*R*)-**SI-2**

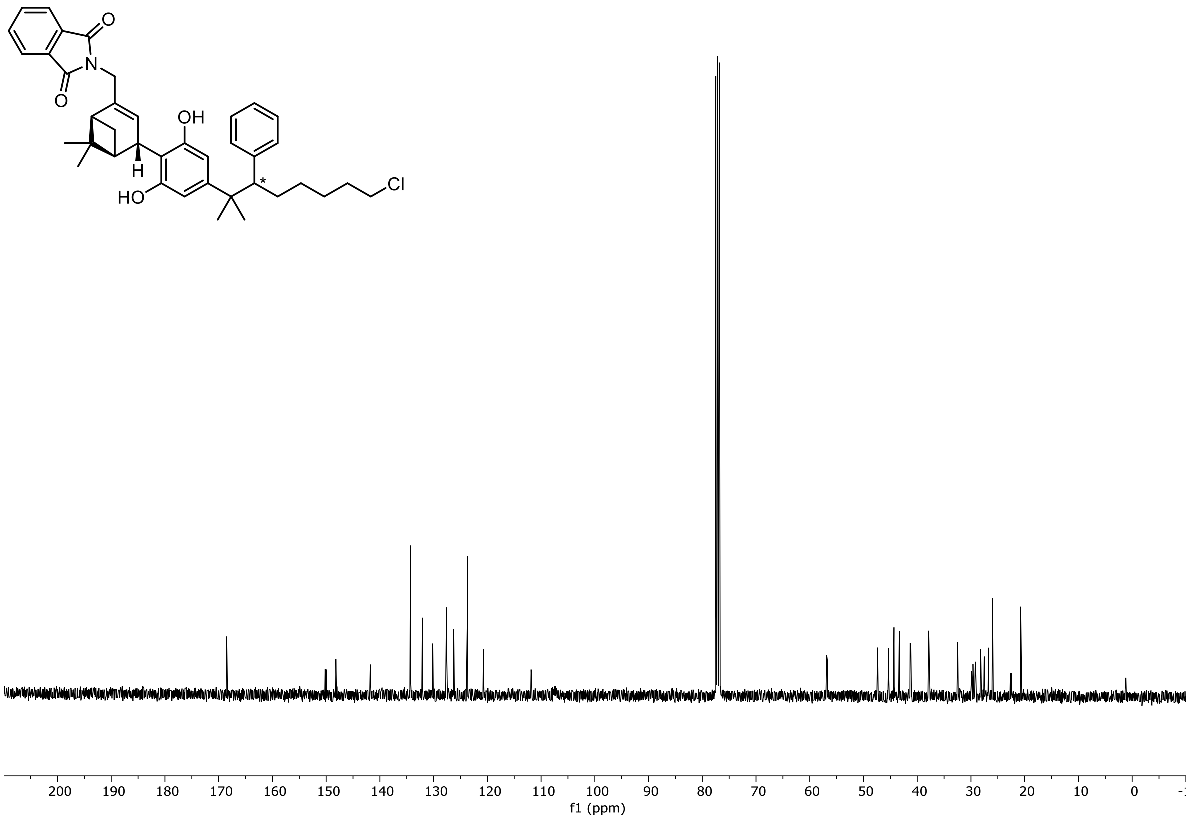

^{1}H NMR (400 MHz, $CDCl_3$) of (*S*)-**16**/(*R*)-**16**

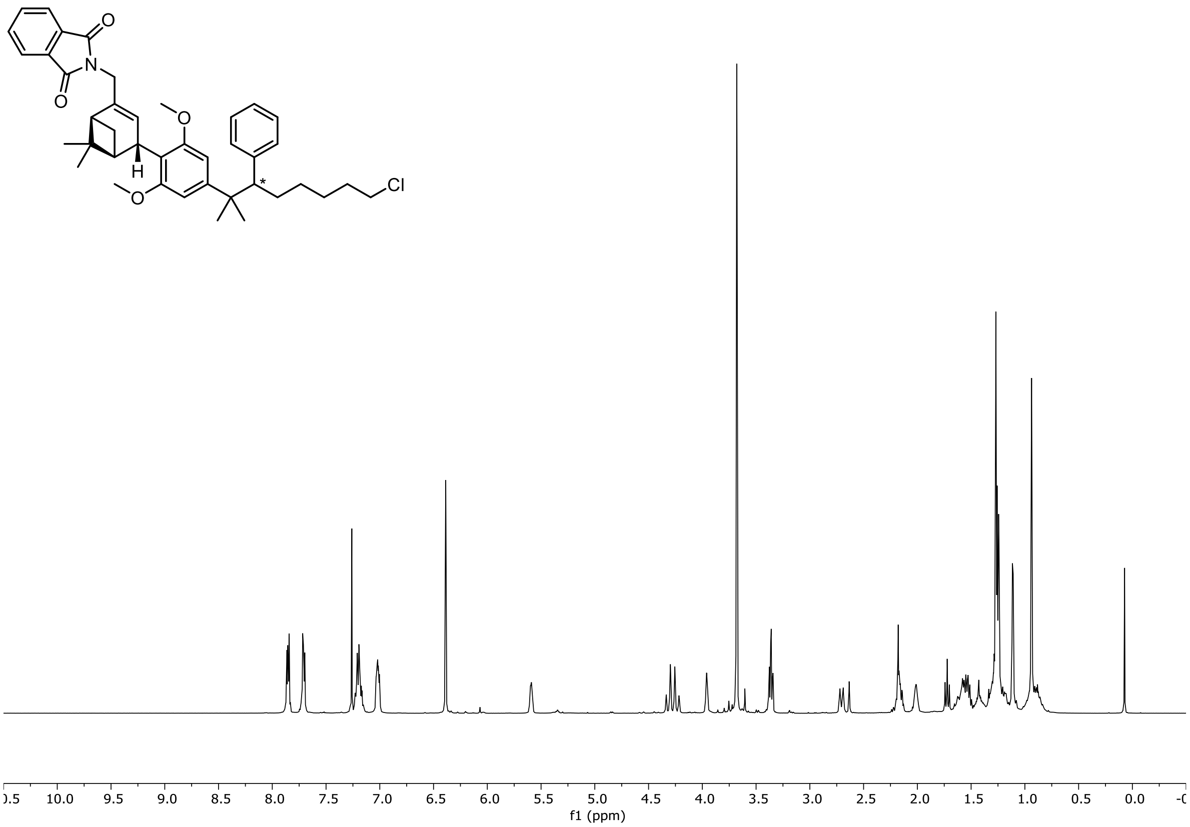

^{13}C NMR (101 MHz, $CDCl_3$) of (*S*)-**16**/(*R*)-**16**

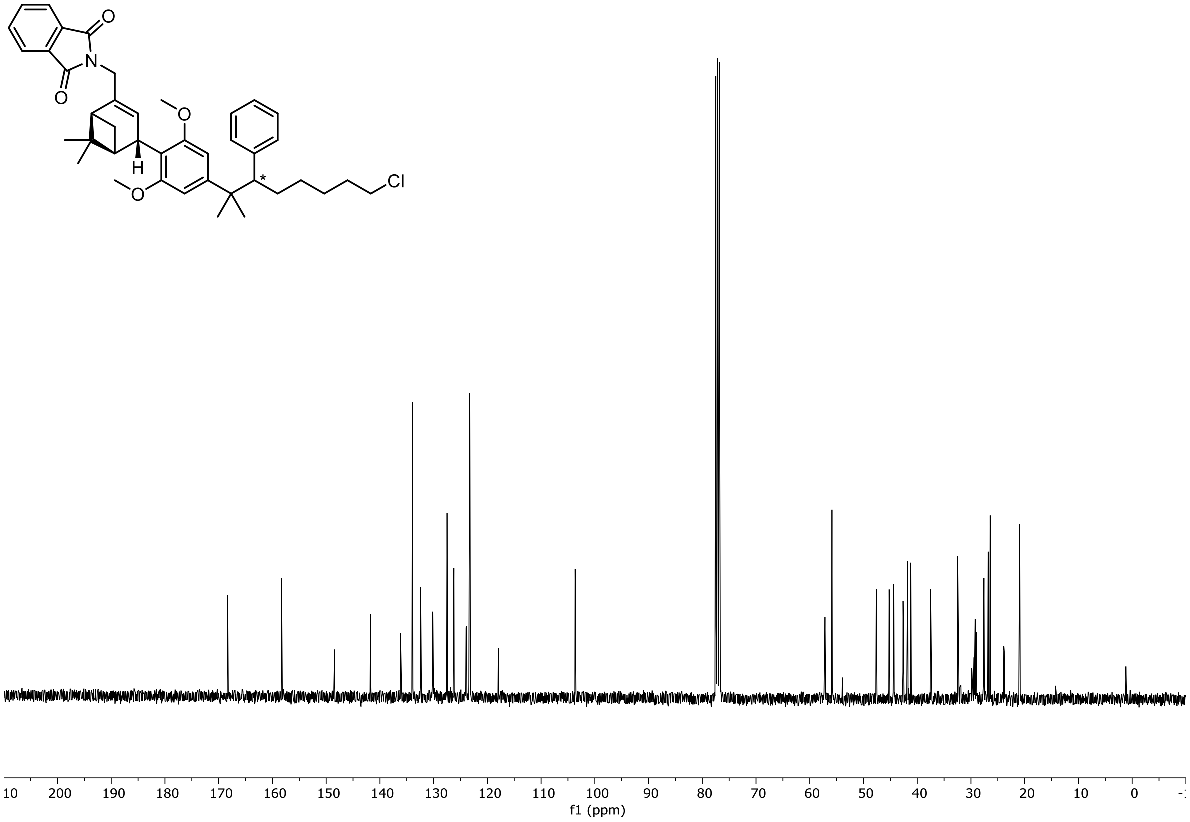

^{1}H NMR (400 MHz, $CDCl_3$) of (*S*)-**SI-3**/(*R*)-**SI-3**

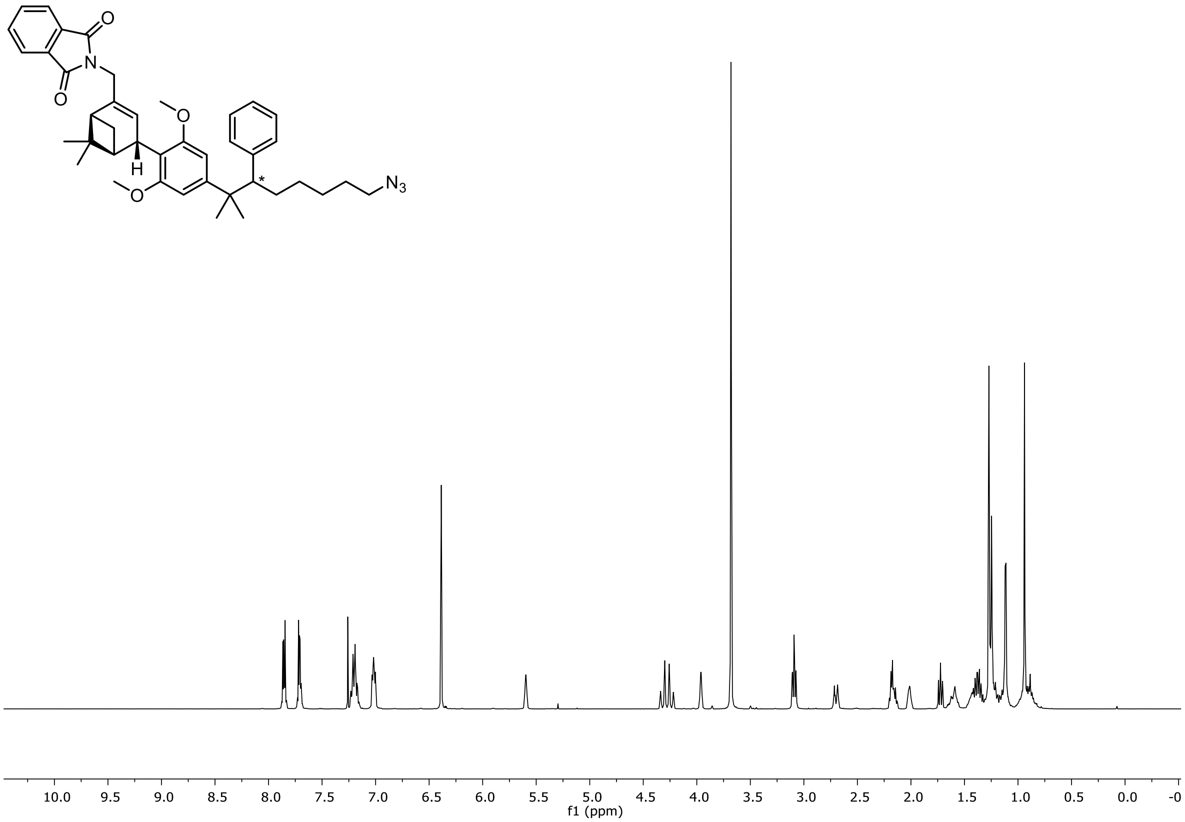

^{13}C NMR (101 MHz, $CDCl_3$) of (*S*)-**SI-3**/(*R*)-**SI-3**

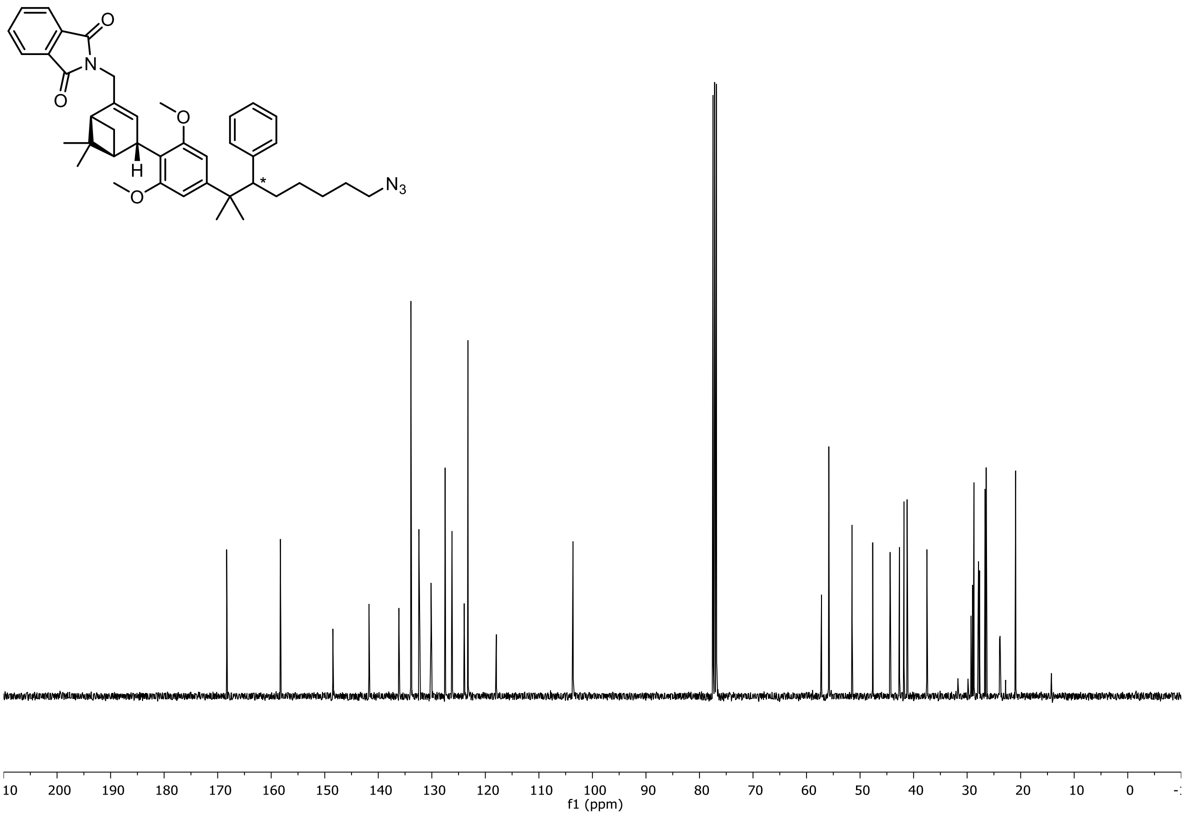

^{1}H NMR (400 MHz, $CDCl_3$) of (*S*)-**1**/(*R*)-**1**

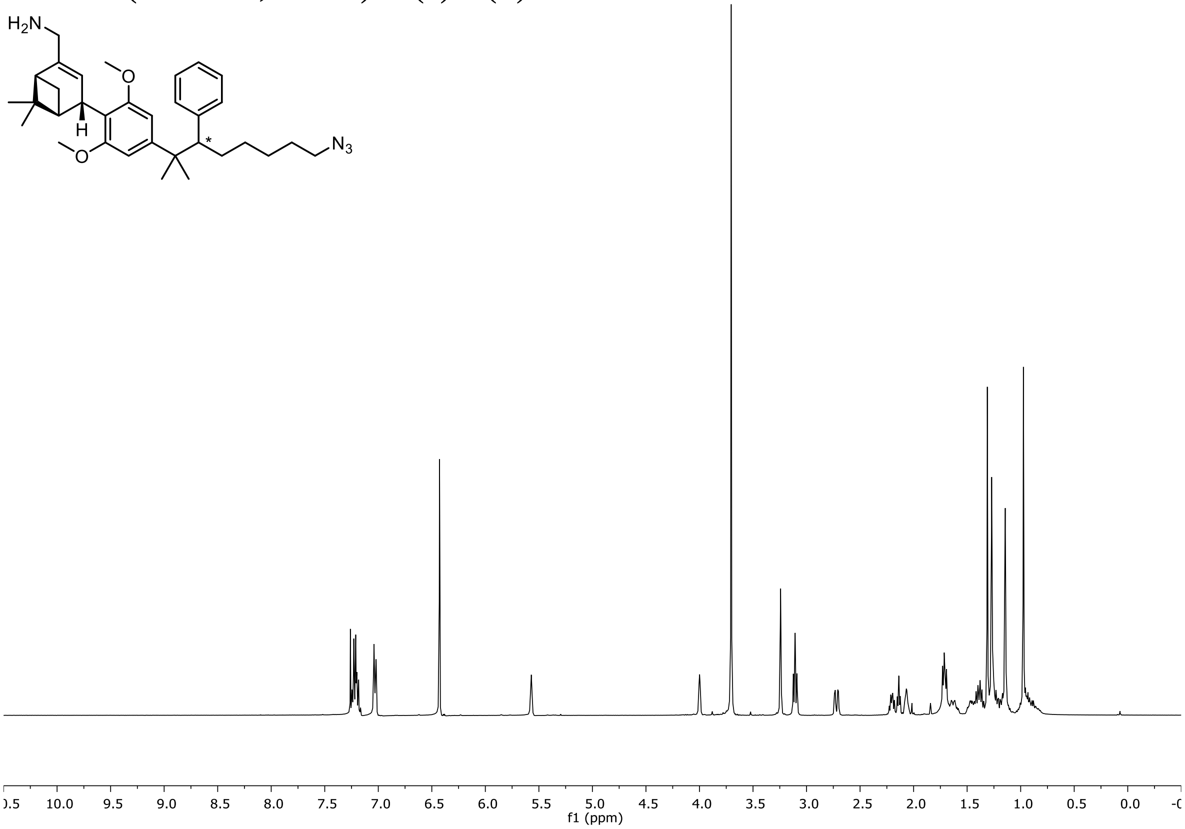

^{13}C NMR (101 MHz, $CDCl_3$) of (*S*)-**1**/(*R*)-**1**

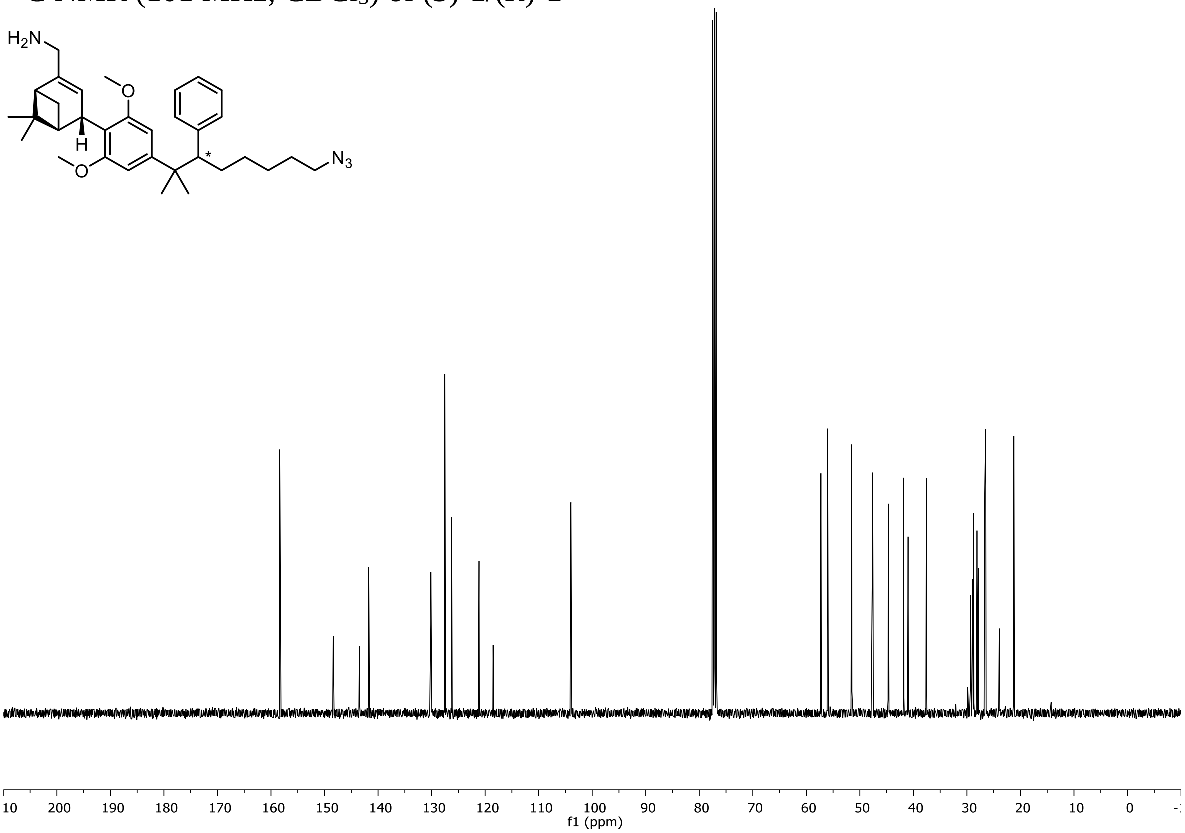

^{1}H NMR (500 MHz, CD_2Cl_2) of (*S*)-**2**/(*R*)-**2**

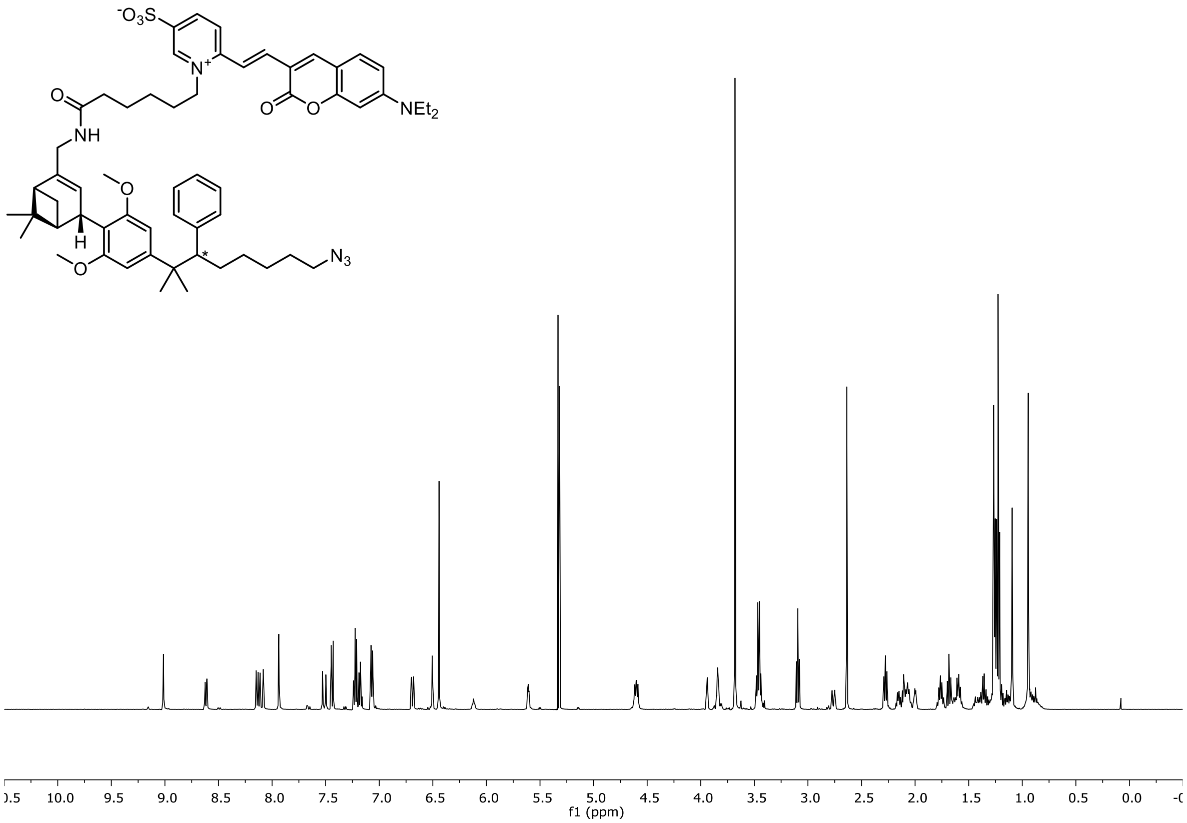

^{13}C NMR (126 MHz, CD_2Cl_2) of (*S*)-**2**/(*R*)-**2**

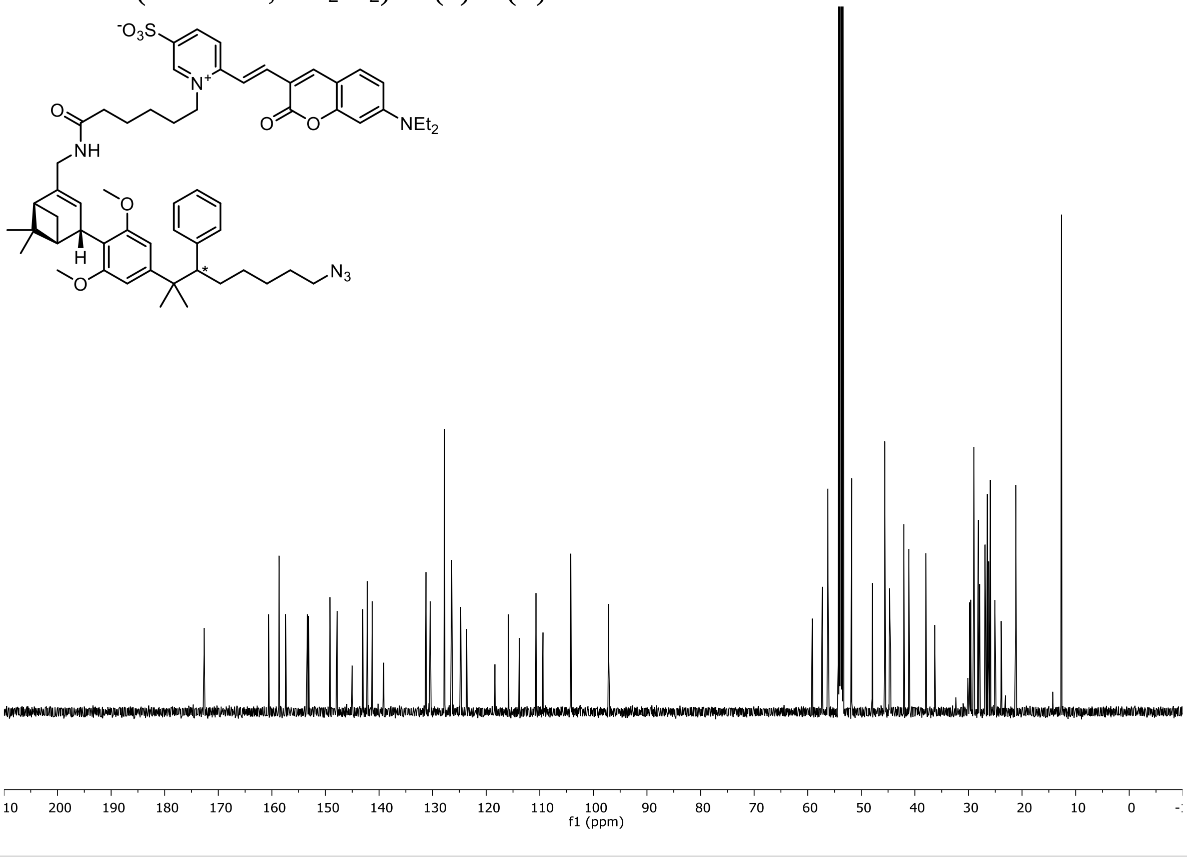

^{1}H NMR (500 MHz, CD_2Cl_2) of (*S*)-**3**/(*R*)-**3**

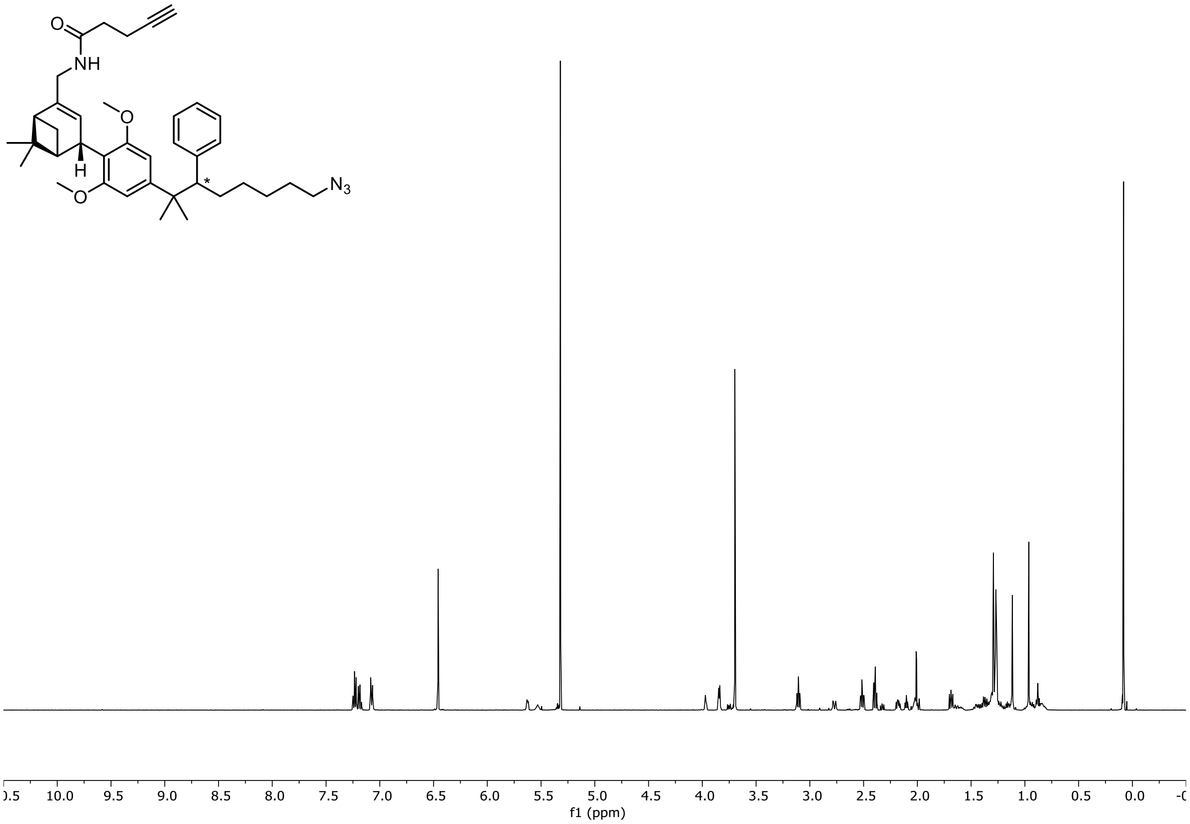

^{13}C NMR (126 MHz, CD_2Cl_2) of (*S*)-**3**/(*R*)-**3**

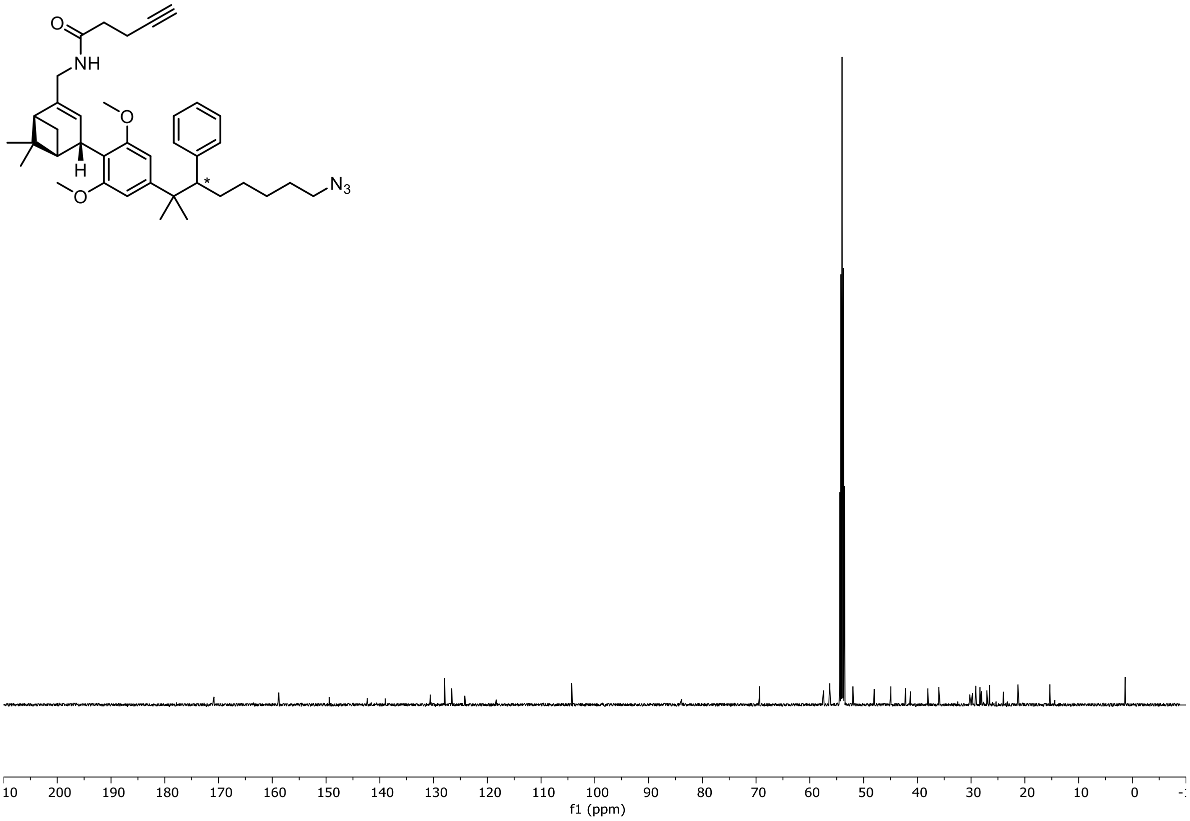

^{1}H NMR (400 MHz, $CDCl_3$) of (*S*)-**SI-2**

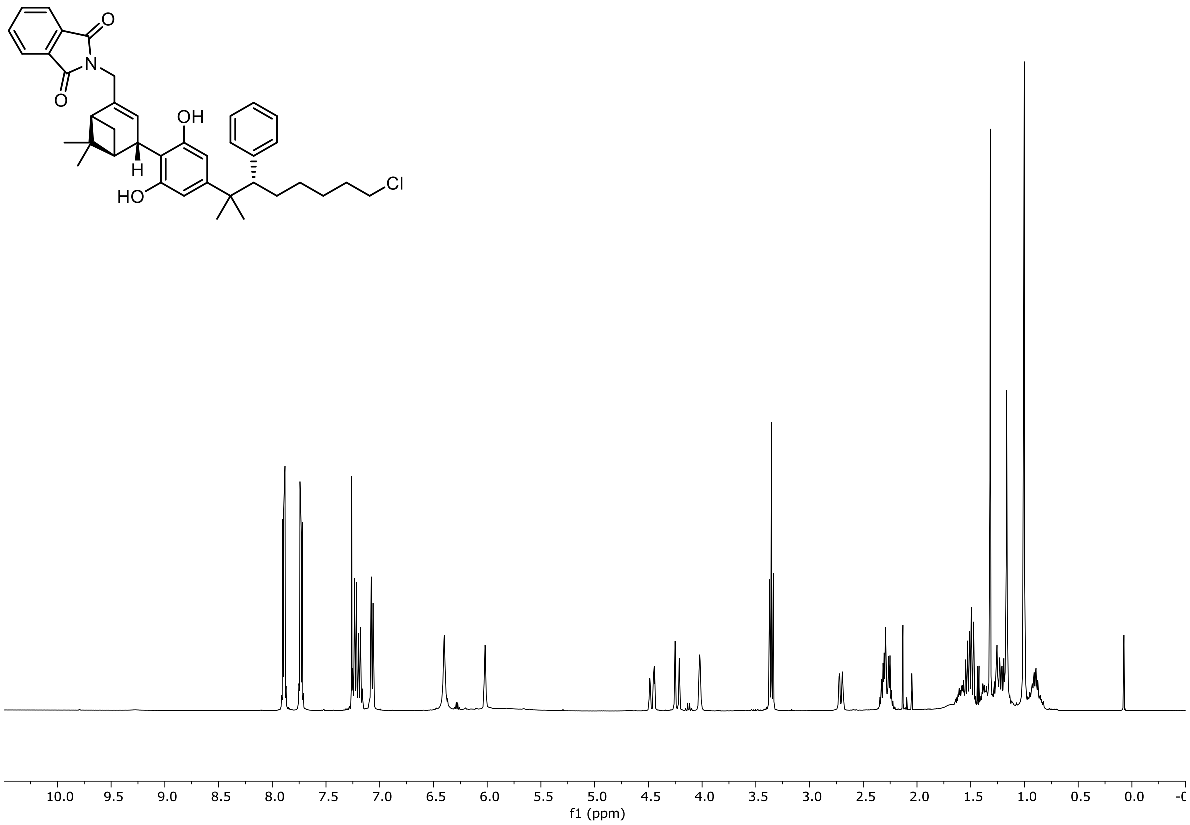

^{13}C NMR (101 MHz, $CDCl_3$) of (*S*)-**SI-2**

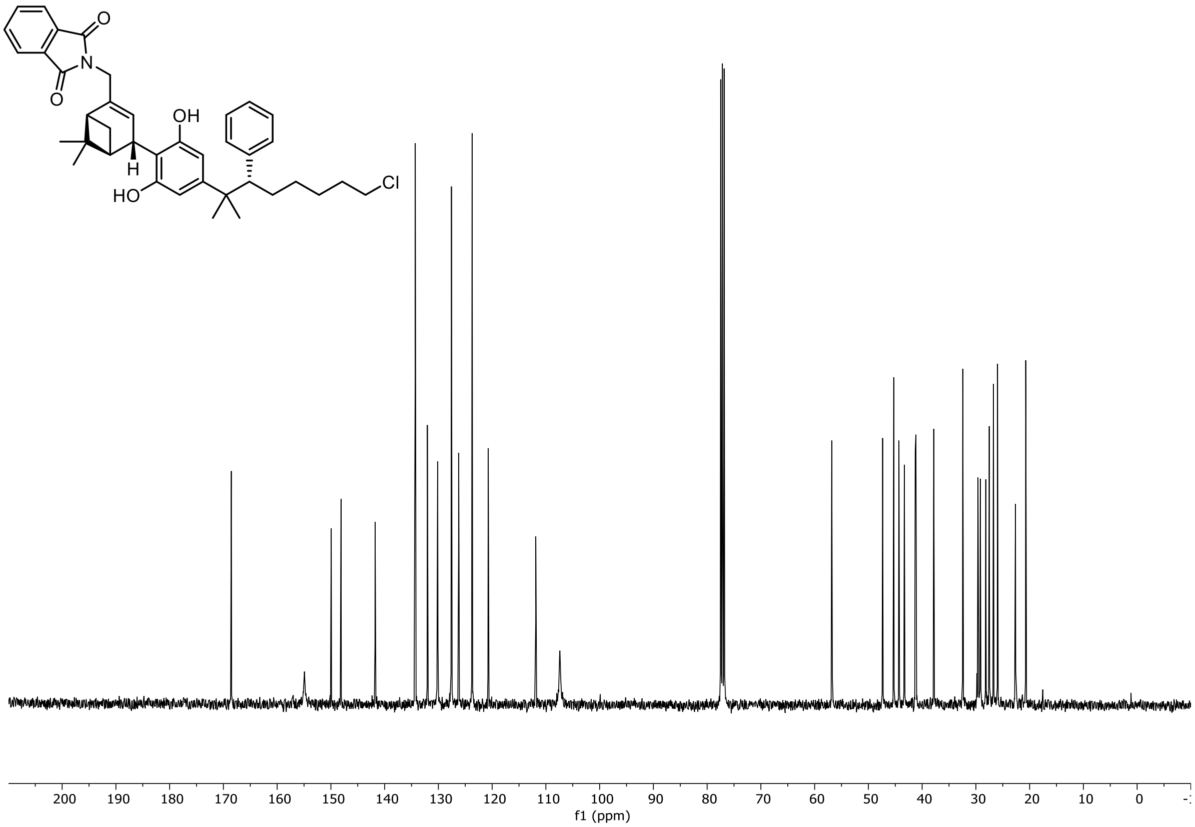

^{1}H NMR (400 MHz, $CDCl_3$) of (*R*)-**SI-2**

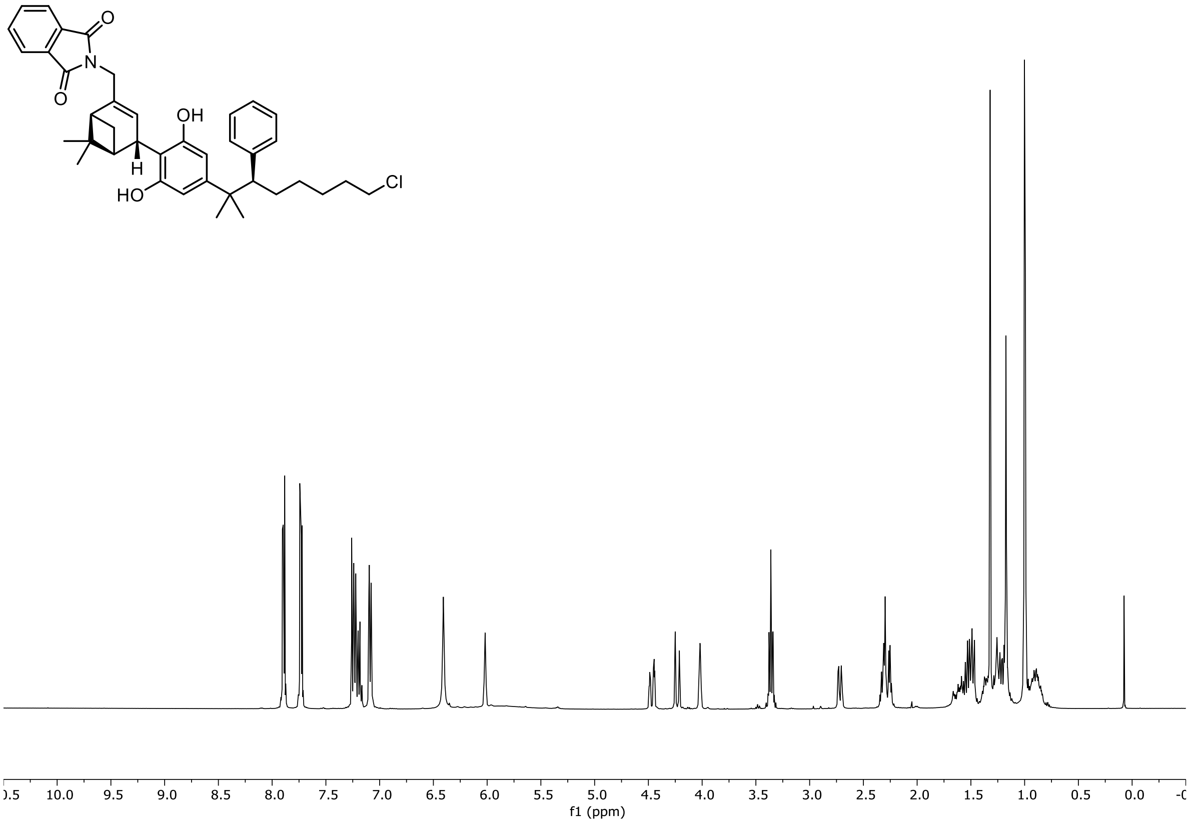

^{13}C NMR (101 MHz, $CDCl_3$) of (*R*)-**SI-2**

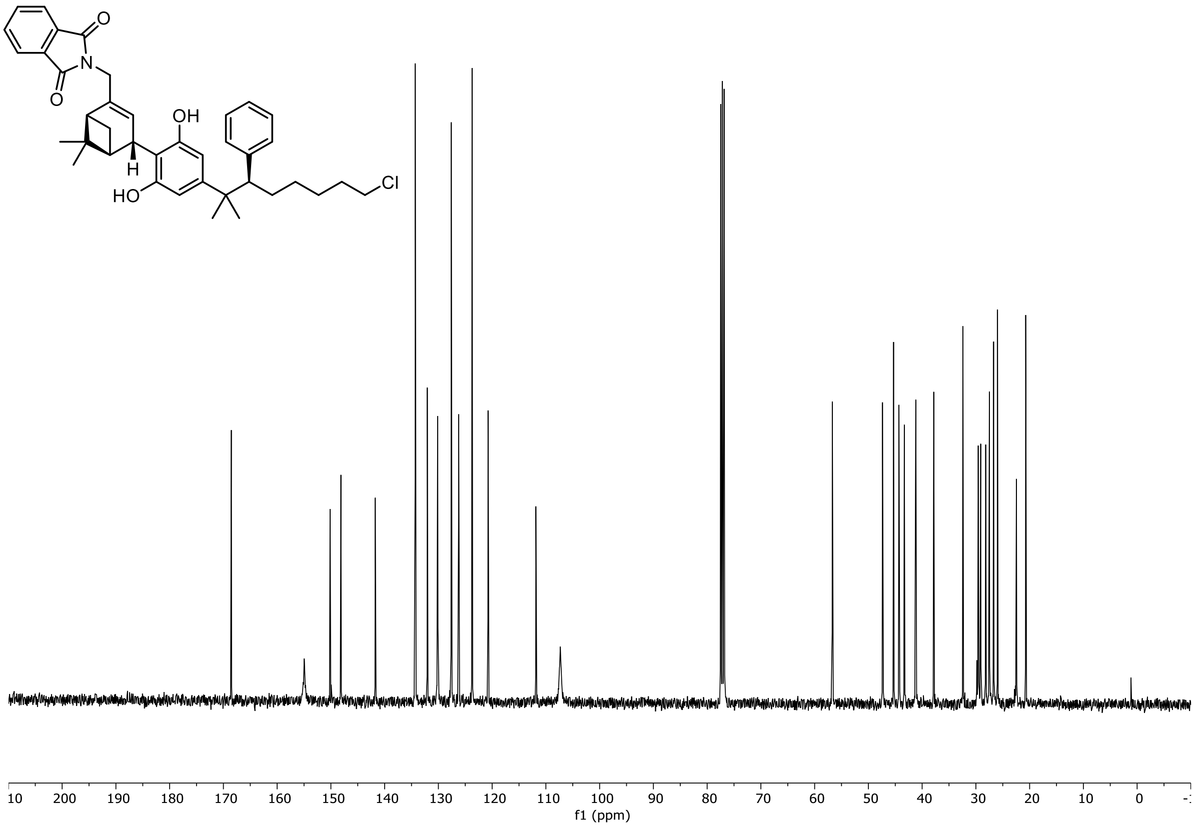

^{1}H NMR (400 MHz, $CDCl_3$) of (*S*)-**16**

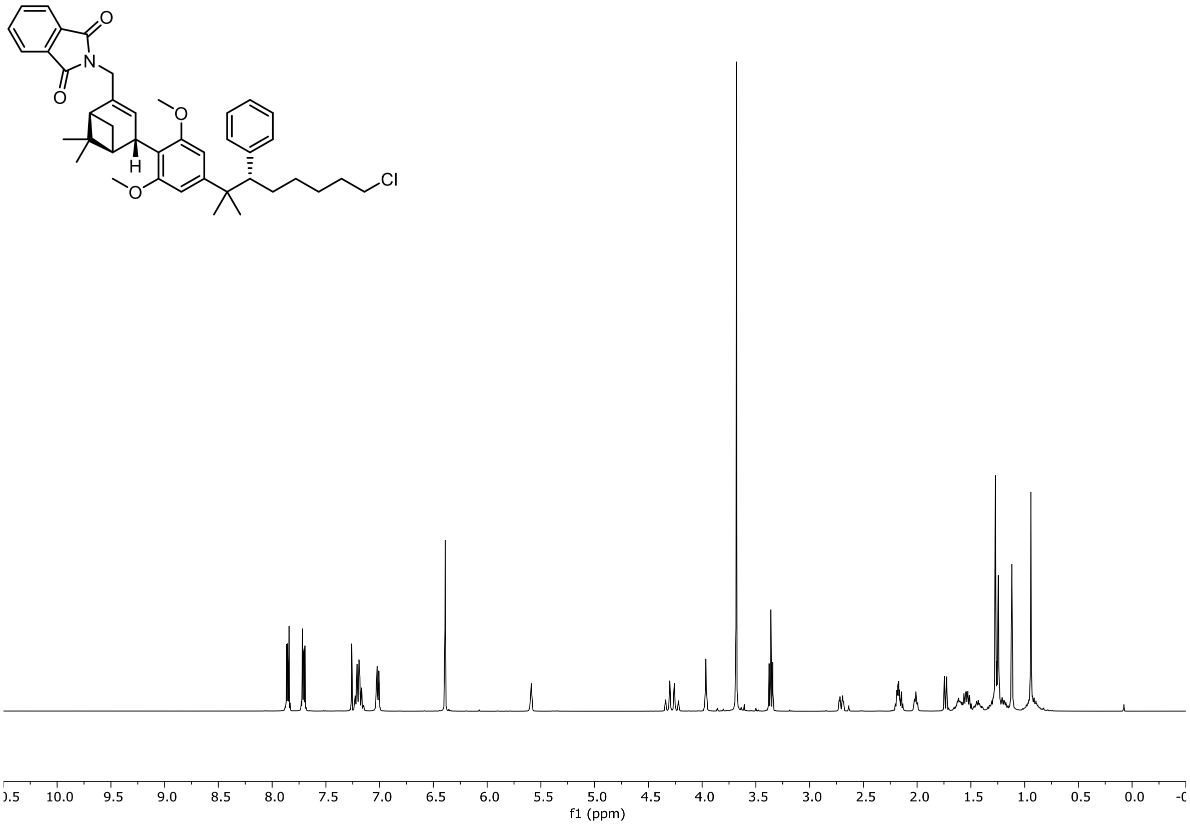

^{13}C NMR (101 MHz, $CDCl_3$) of (*S*)-**16**

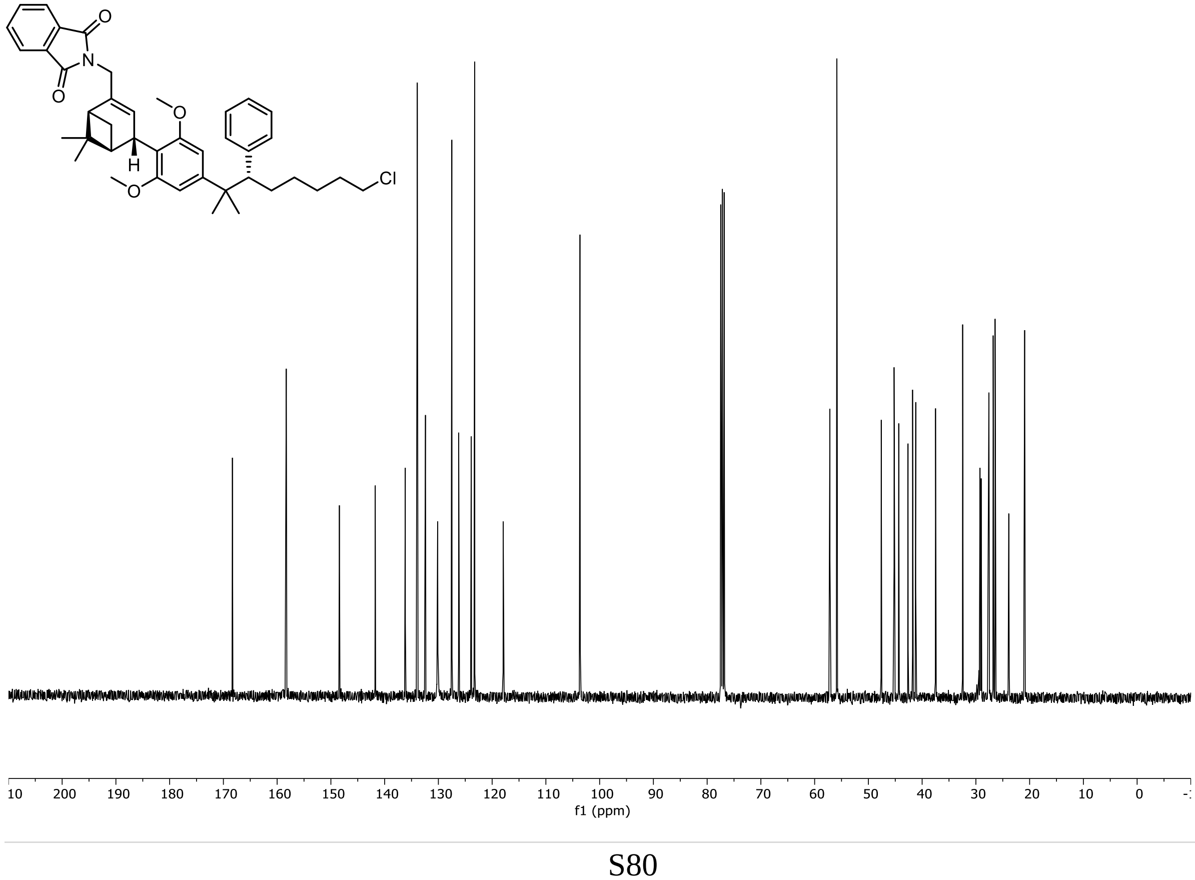

^{1}H NMR (400 MHz, $CDCl_3$) of (*R*)-**16**

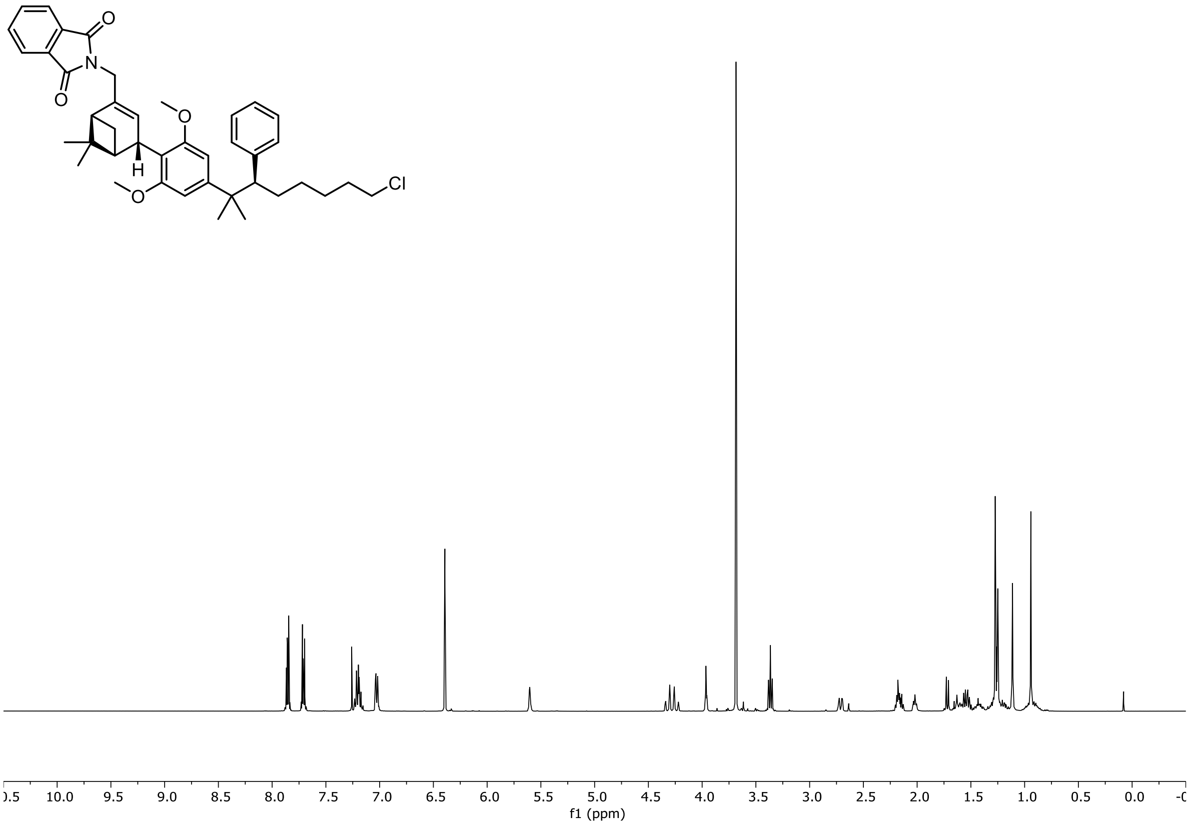

^{13}C NMR (101 MHz, $CDCl_3$) of (*R*)-**16**

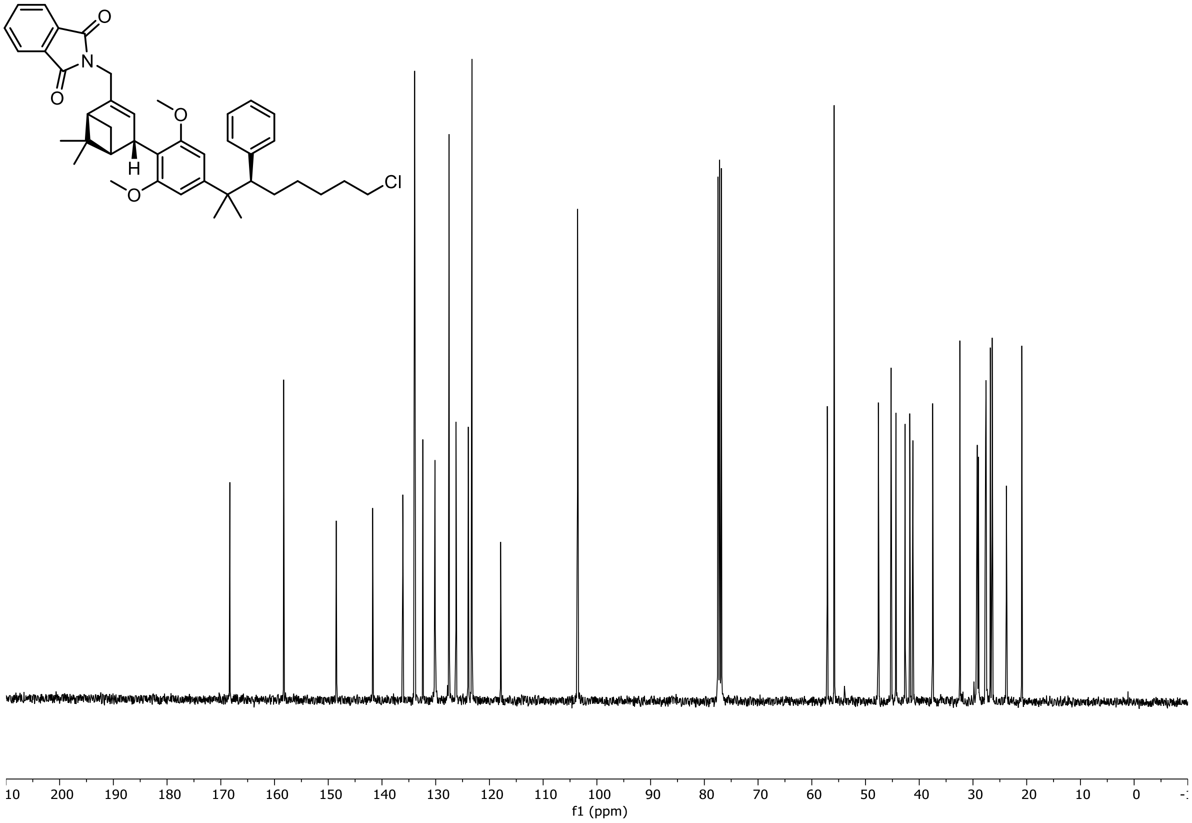

^{1}H NMR (400 MHz, $CDCl_3$) of (*S*)-**SI-3**

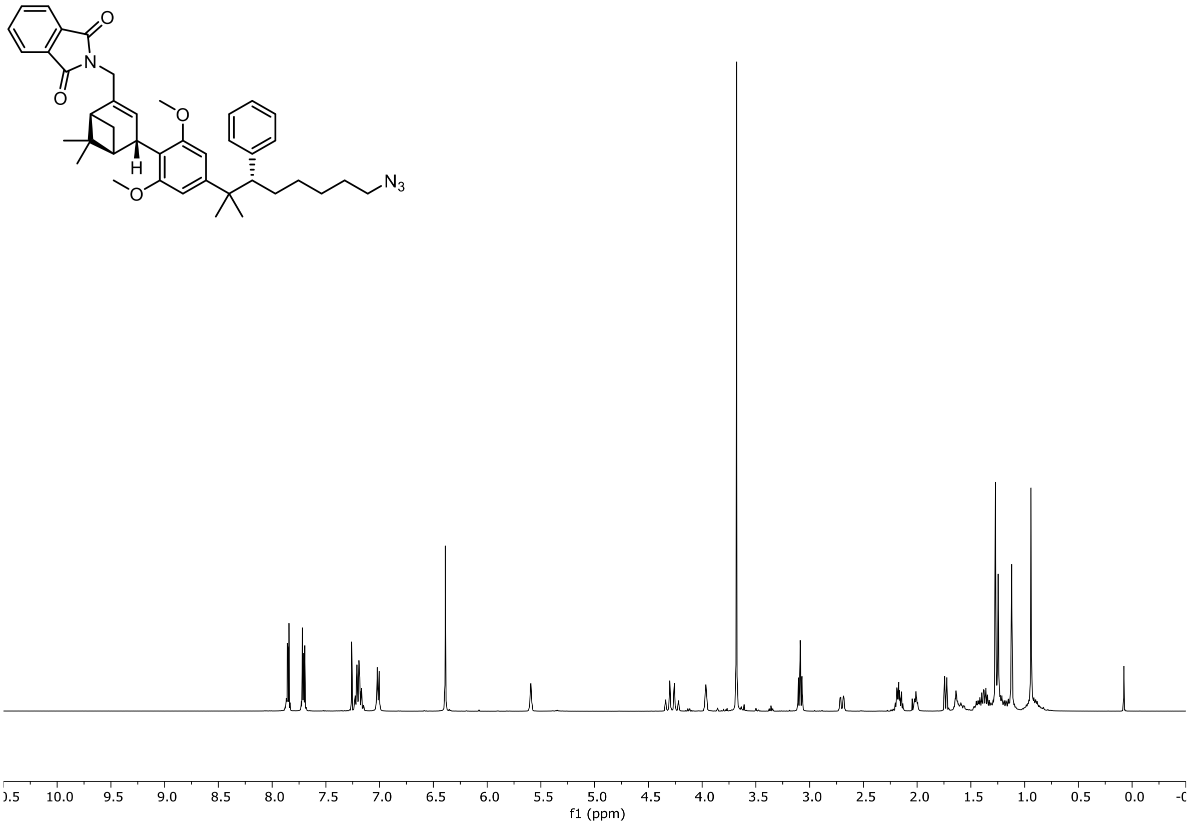

^{13}C NMR (101 MHz, $CDCl_3$) of (*S*)-**SI-3**

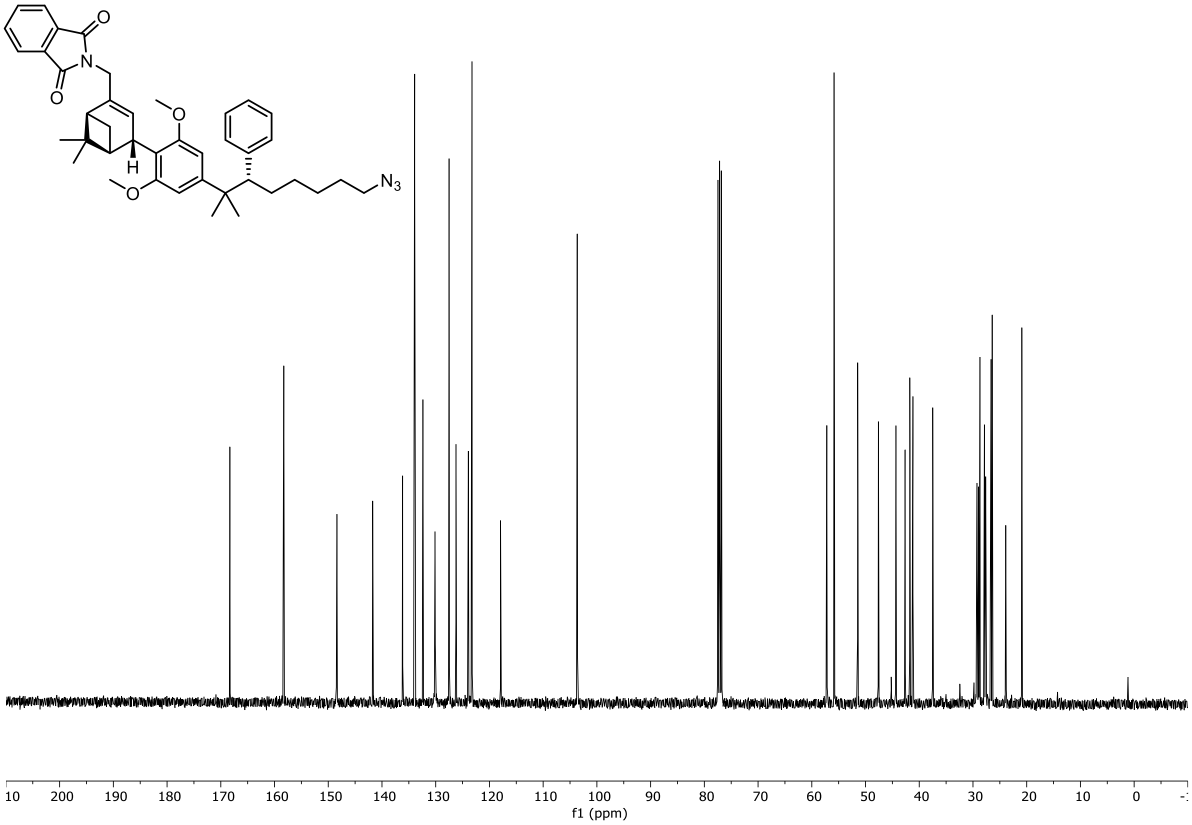

^{1}H NMR (400 MHz, $CDCl_3$) of (*R*)-**SI-3**

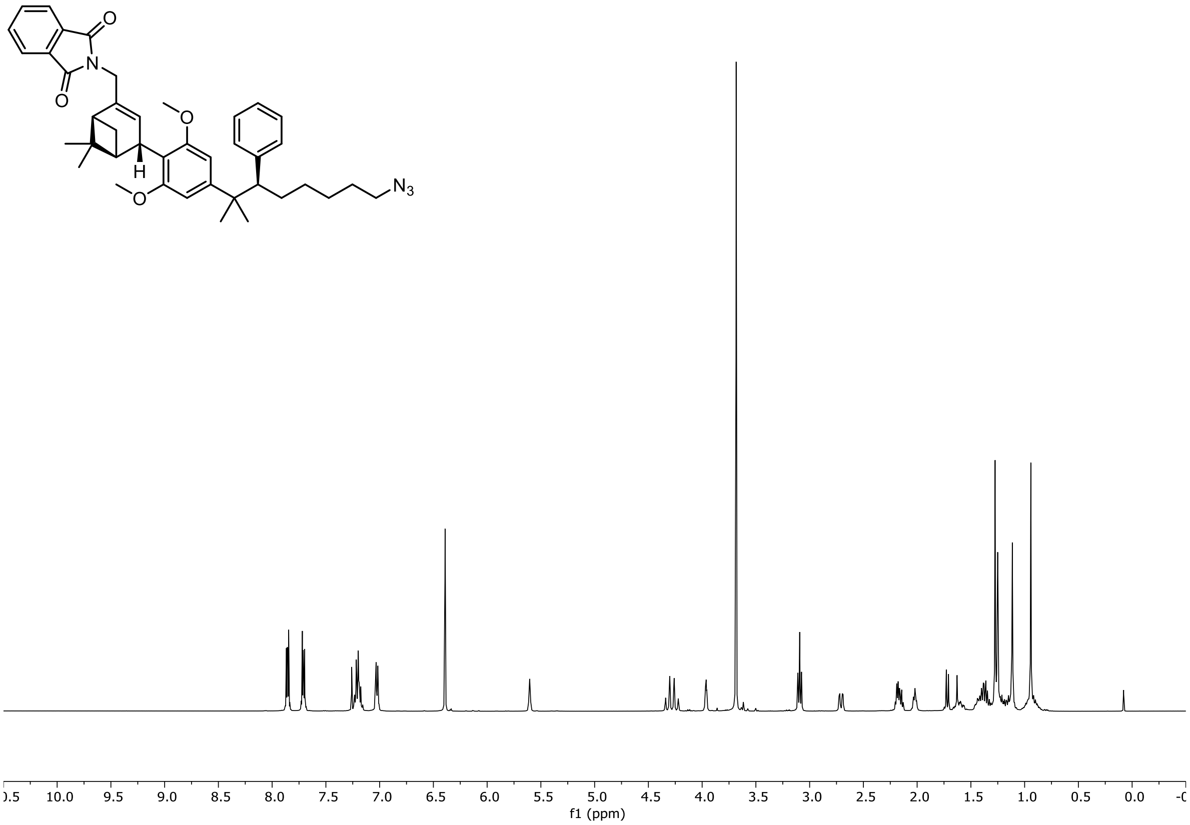

^{13}C NMR (101 MHz, $CDCl_3$) of (*R*)-**SI-3**

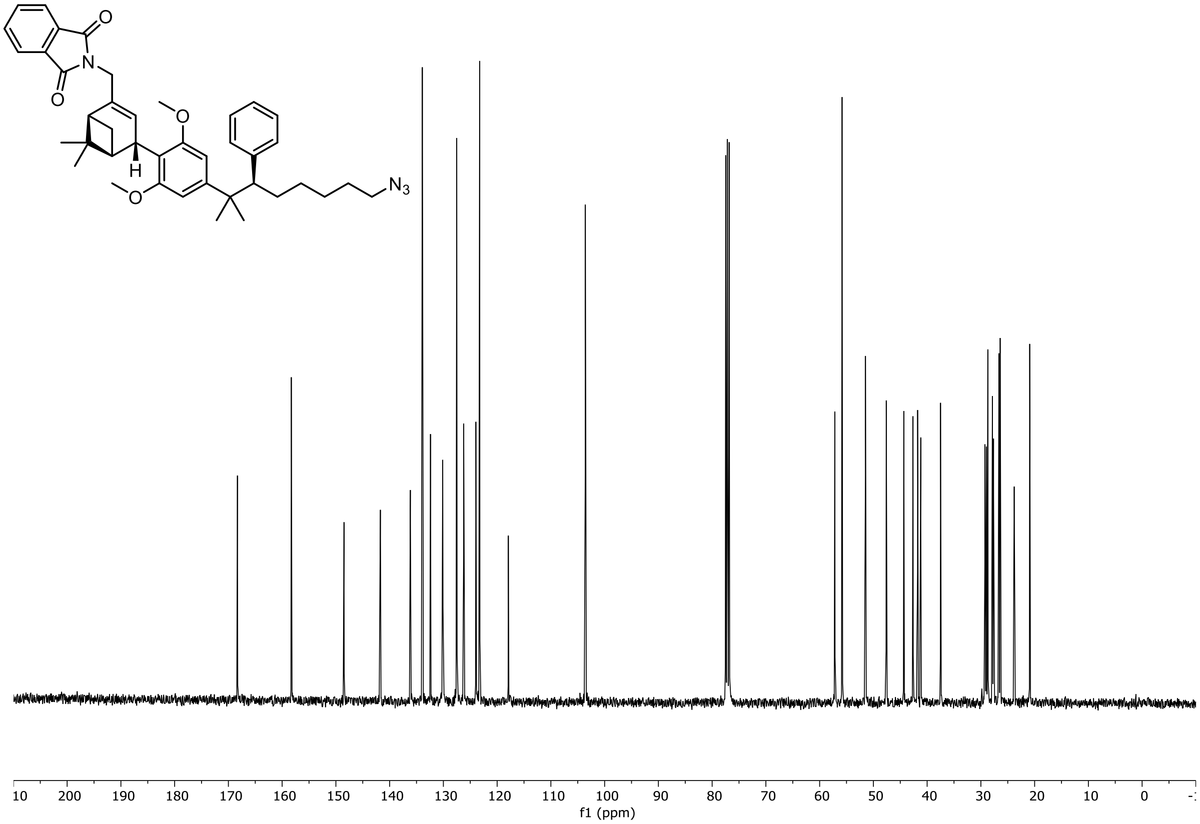

^{1}H NMR (400 MHz, $CDCl_3$) of (*S*)-**1**

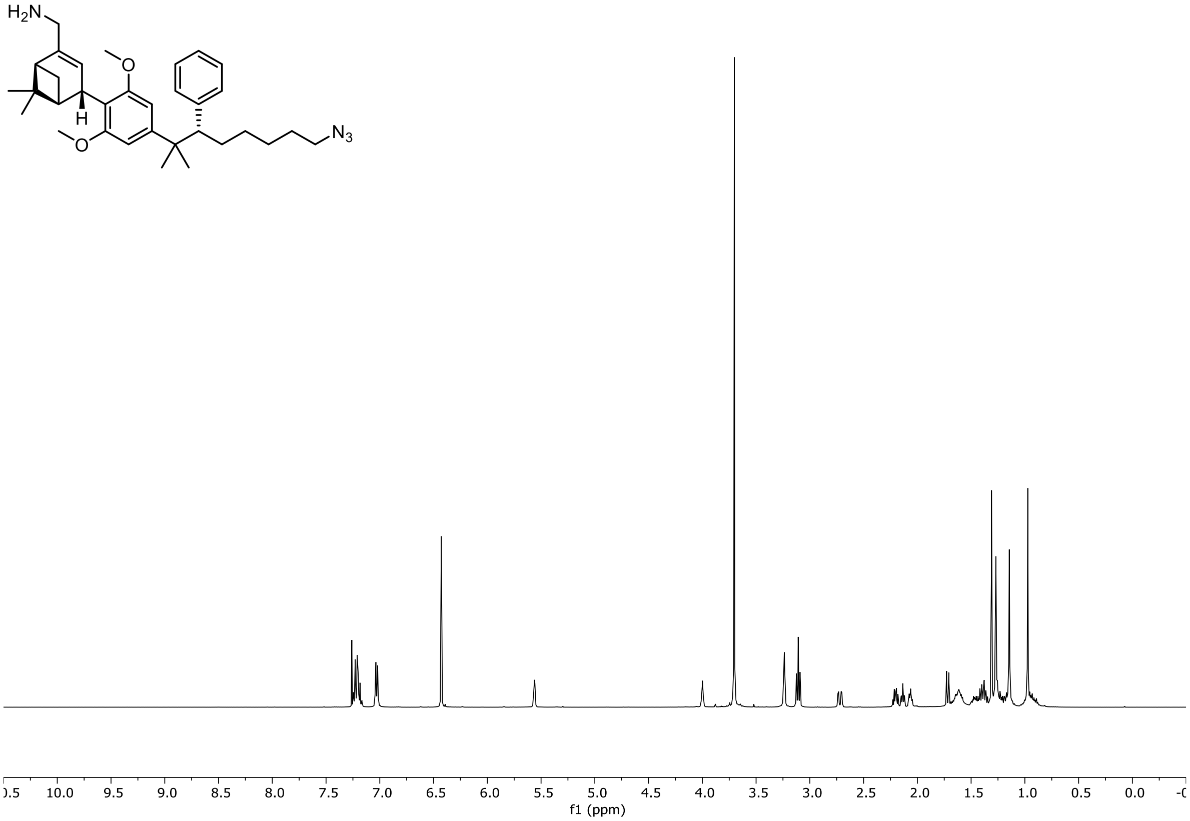

^{13}C NMR (101 MHz, $CDCl_3$) of (*S*)-**1**

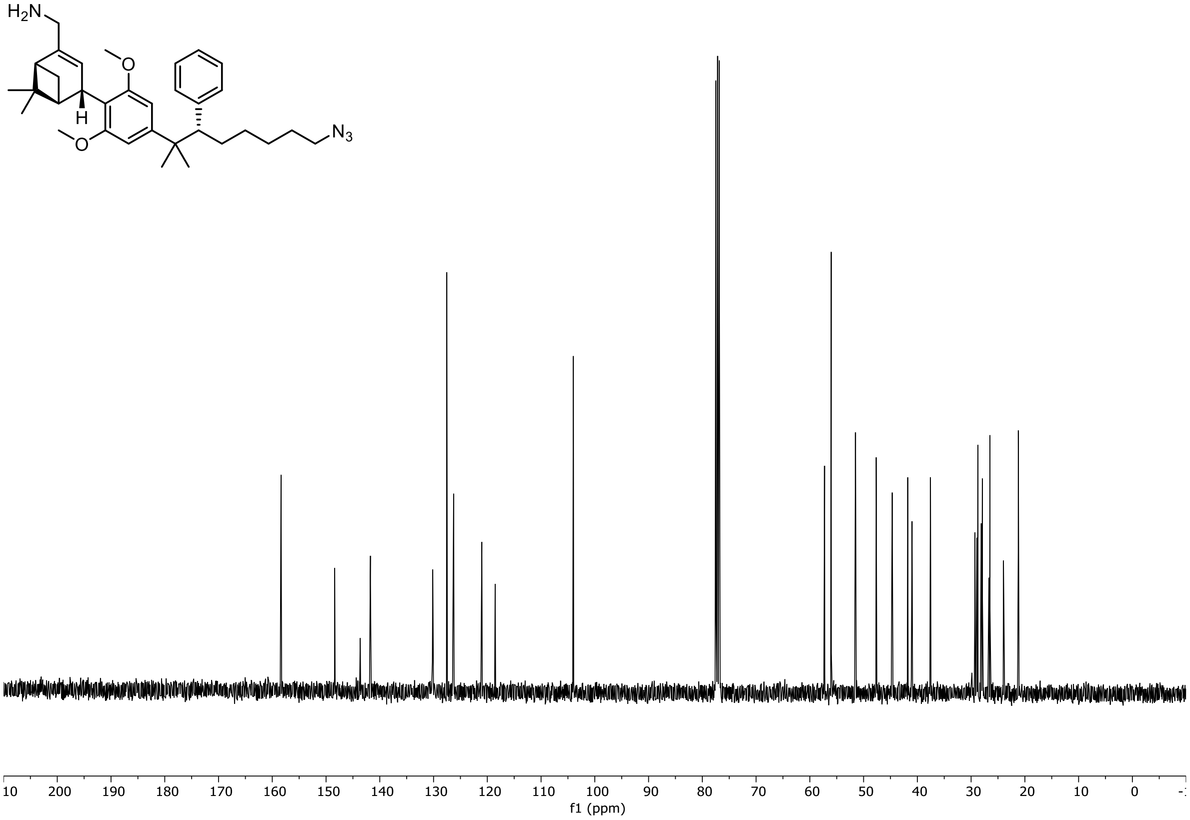

^{1}H NMR (400 MHz, $CDCl_3$) of (*R*)-**1**

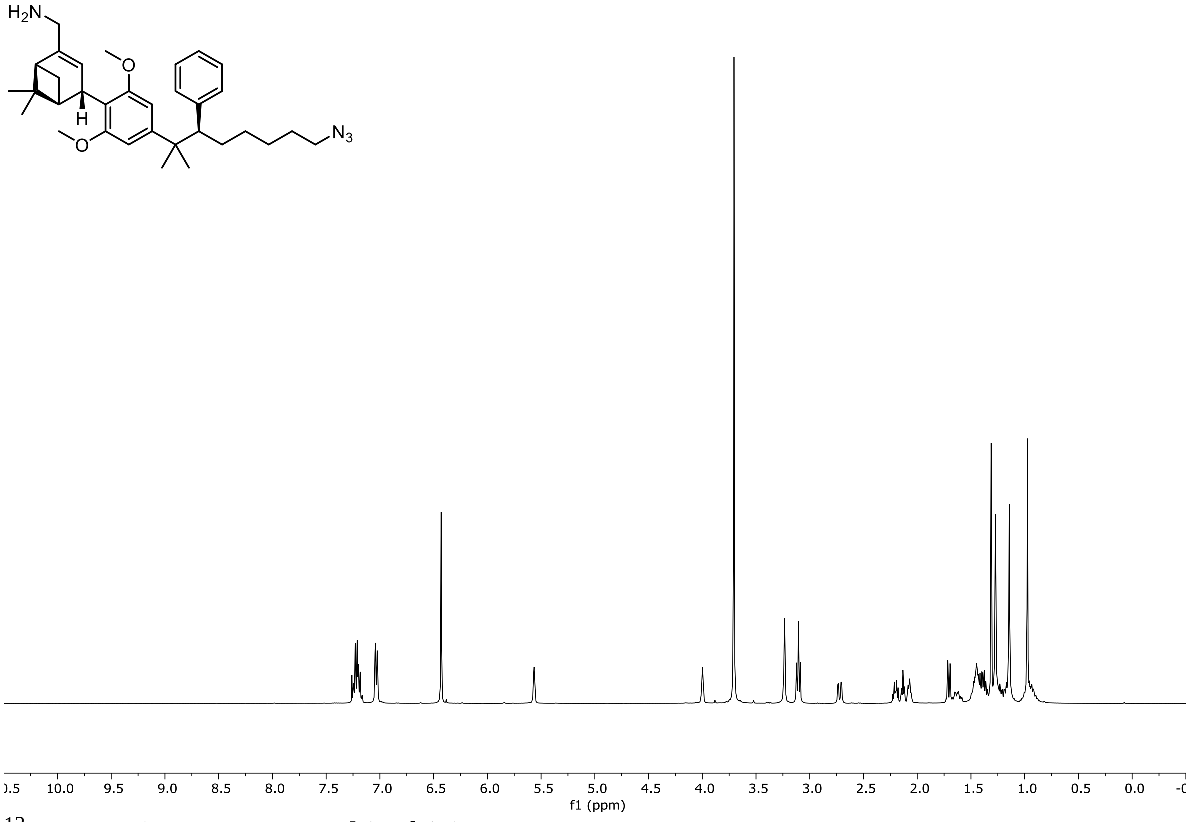

^{13}C NMR (101 MHz, $CDCl_3$) of (*R*)-**1**

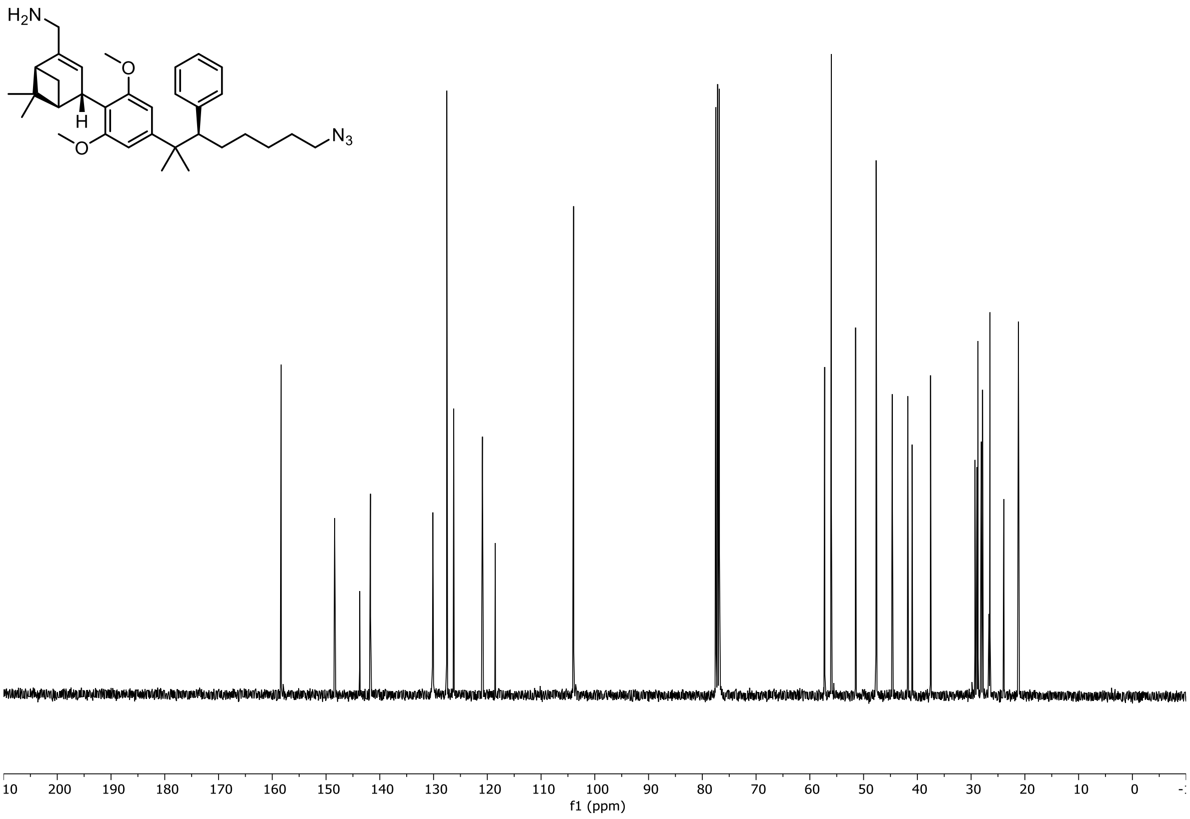

^{1}H NMR (500 MHz, CD_2Cl_2) of (*S*)-**2**

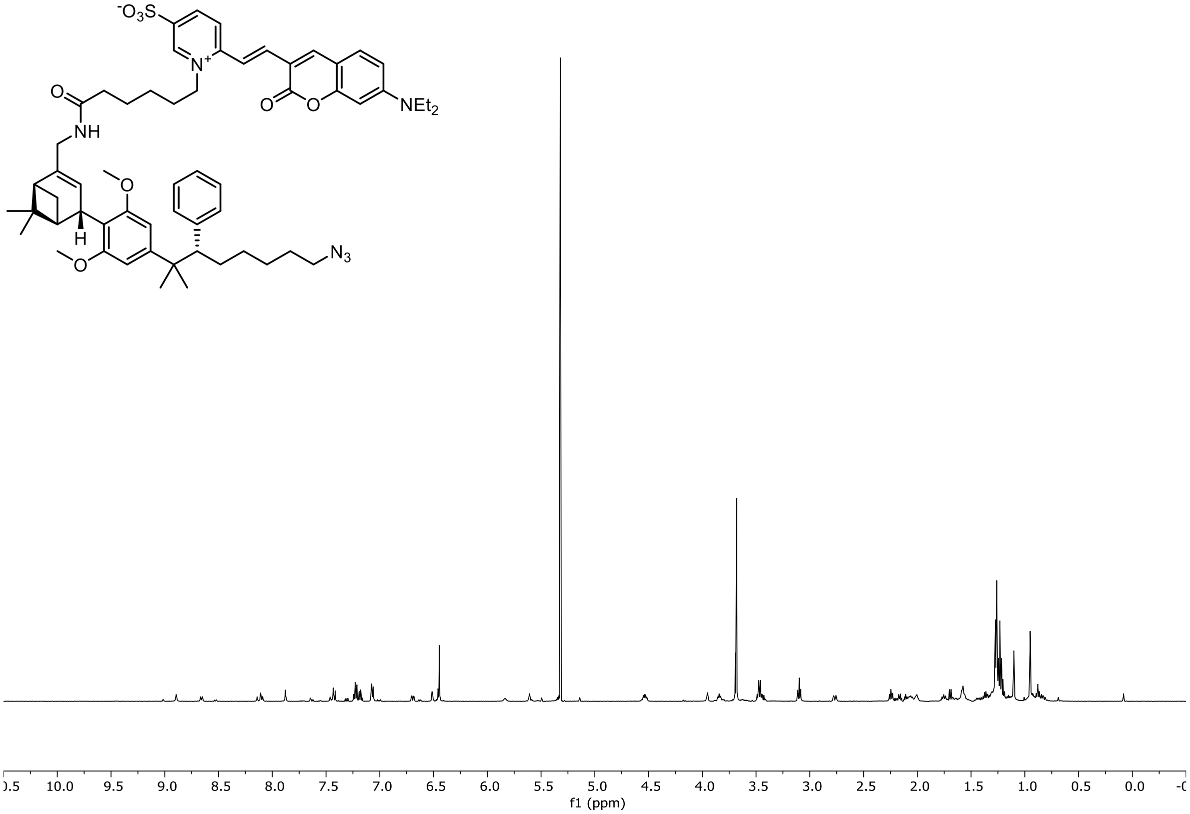

^{13}C NMR (126 MHz, CD_2Cl_2) of (*S*)-**2**

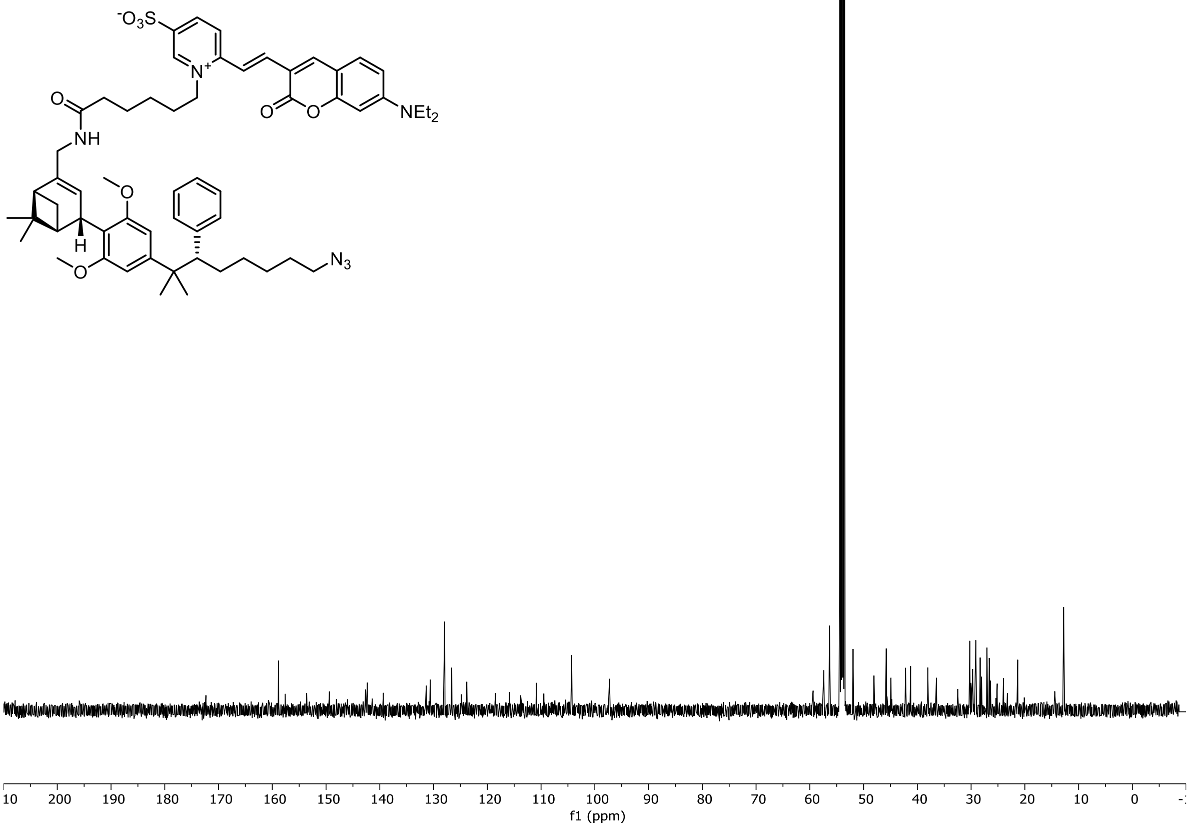

^{1}H NMR (500 MHz, CD_2Cl_2) of (*R*)-**2**

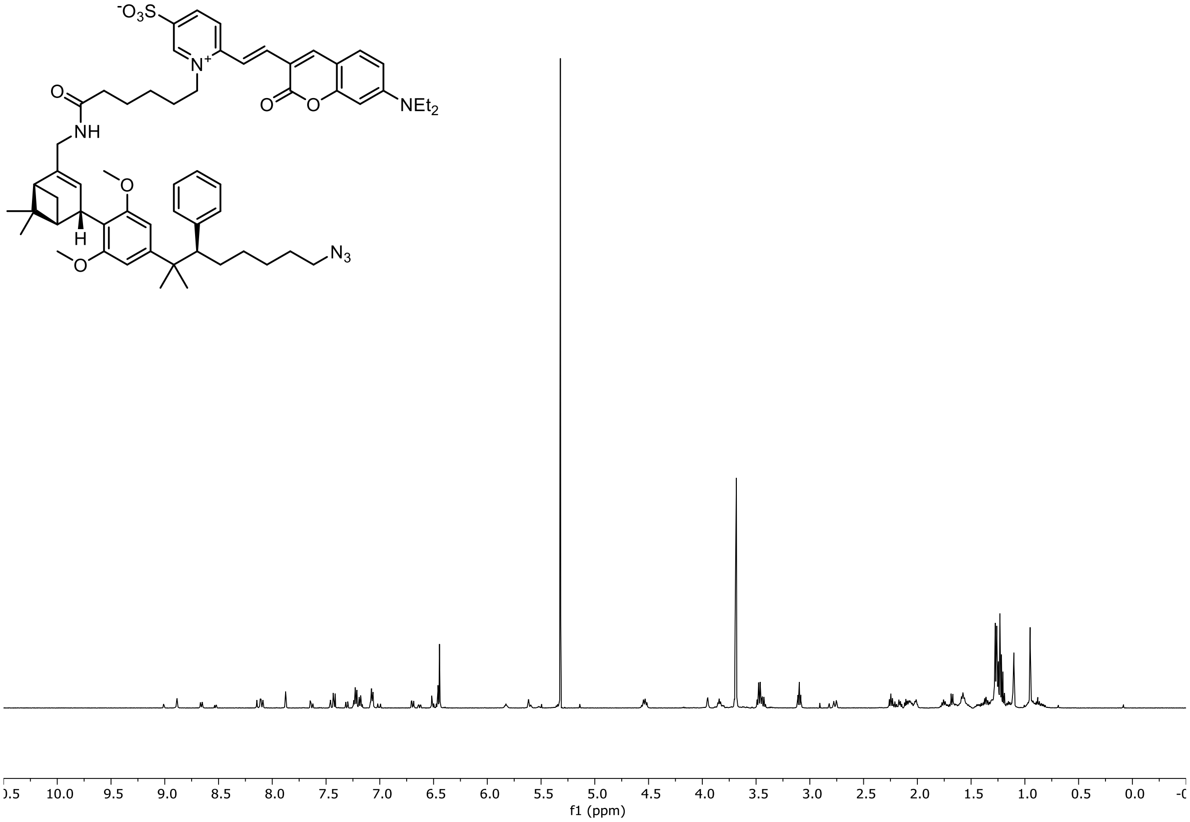

^{13}C NMR (126 MHz, CD_2Cl_2) of (*R*)-**2**

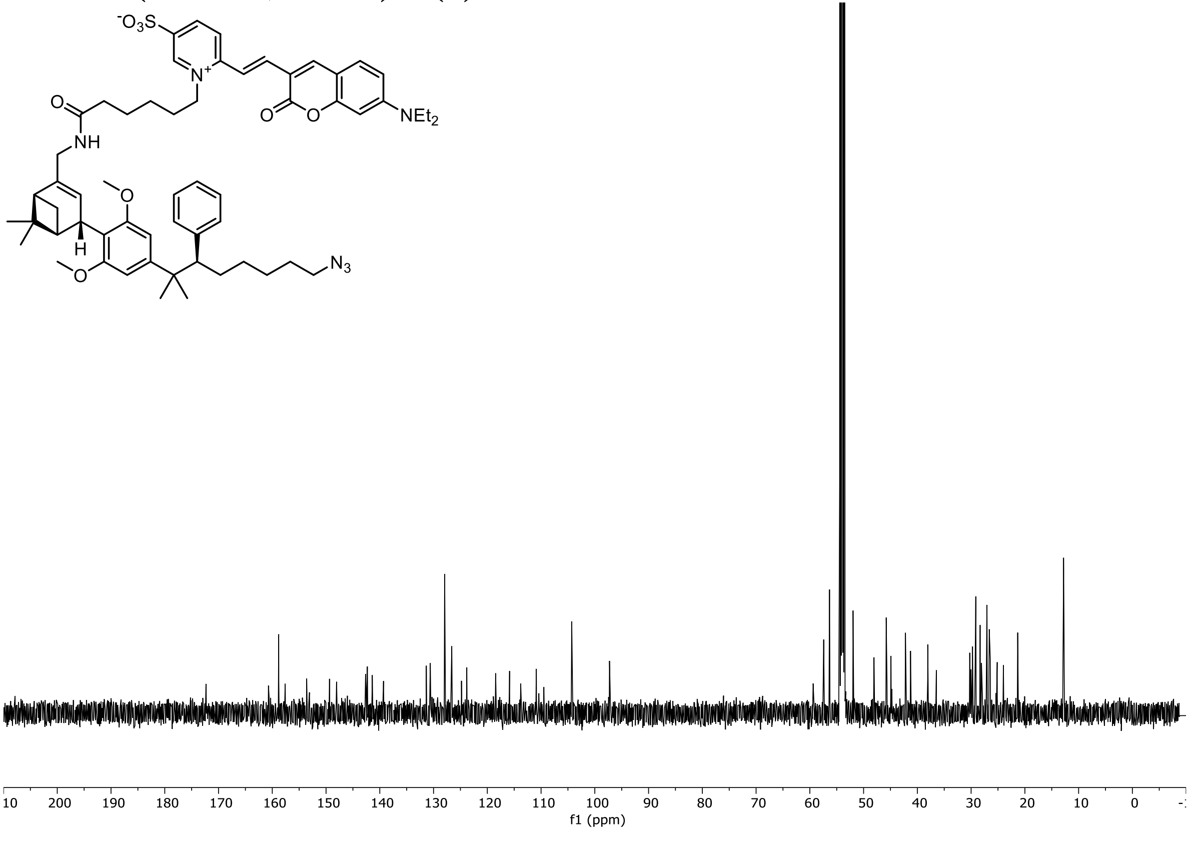

^{1}H NMR (400 MHz, $CDCl_3$) of (*S*)-**3**

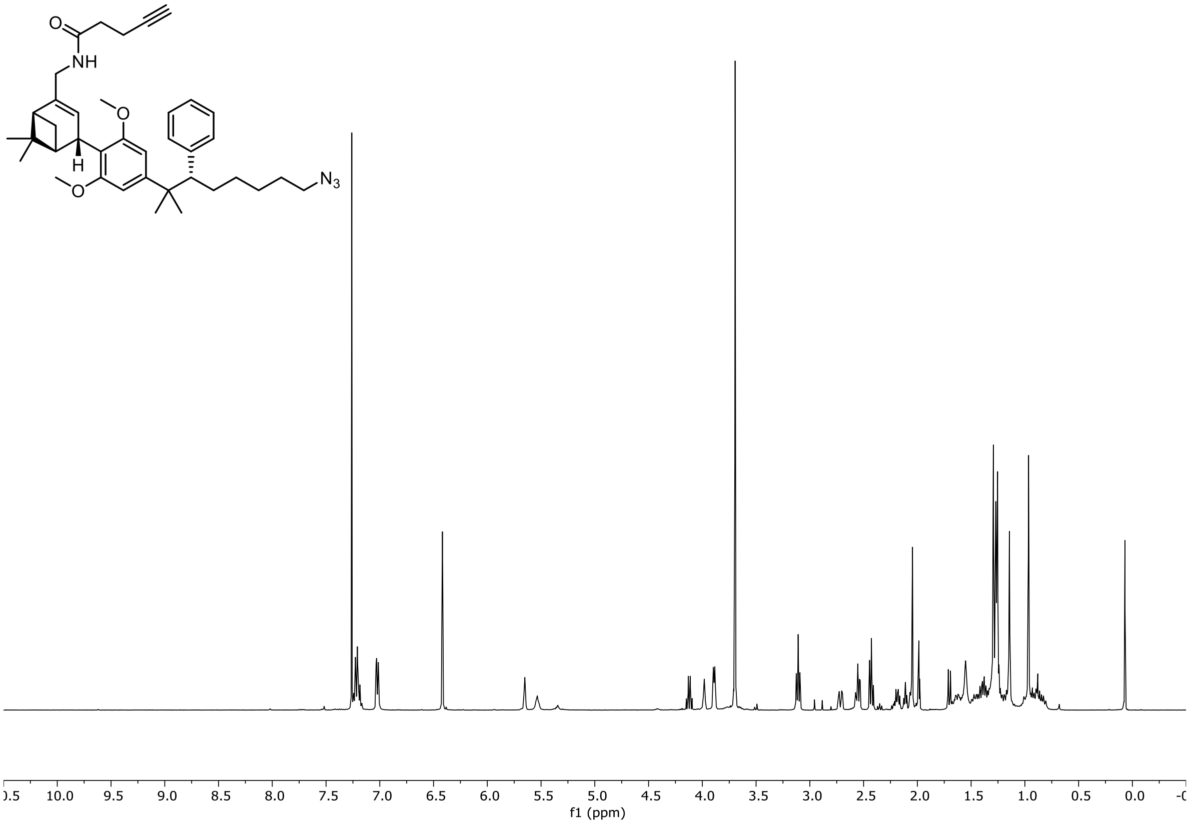

^{13}C NMR (101 MHz, $CDCl_3$) of (*S*)-**3**

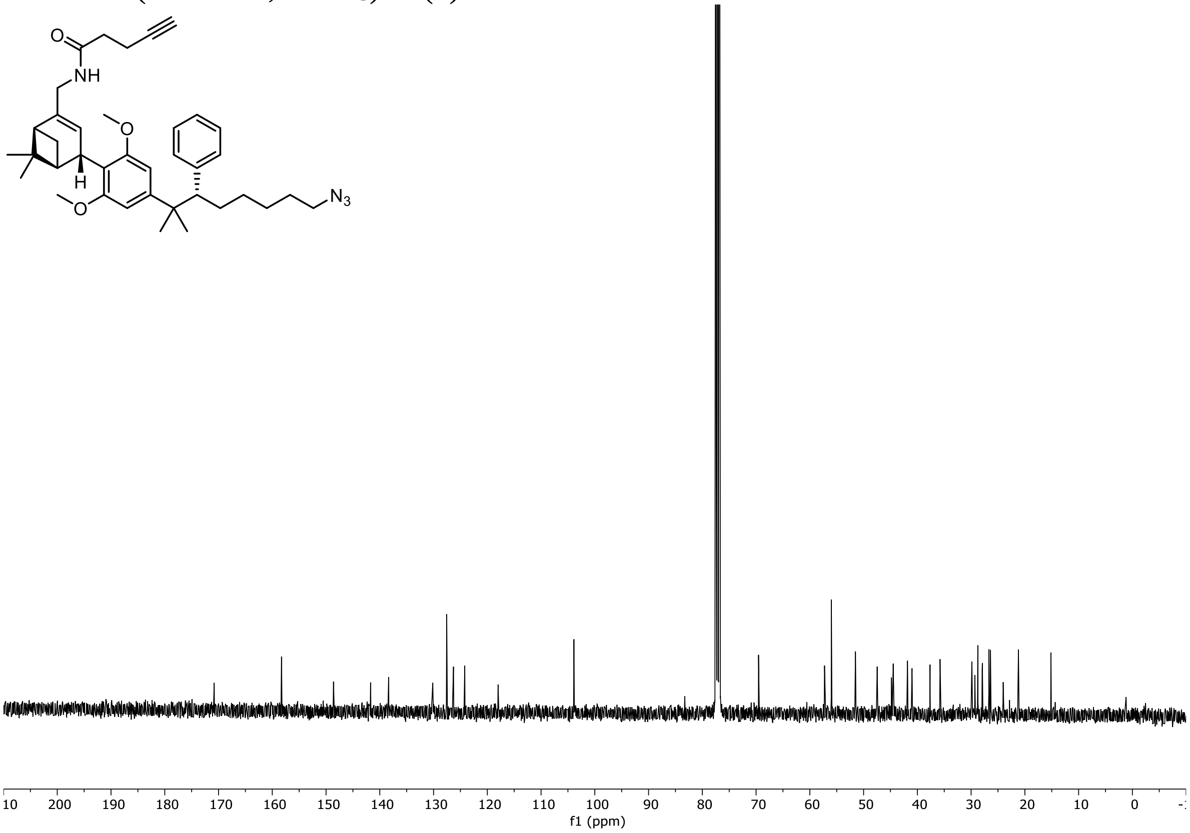

^{1}H NMR (400 MHz, $CDCl_3$) of (*R*)-**3**

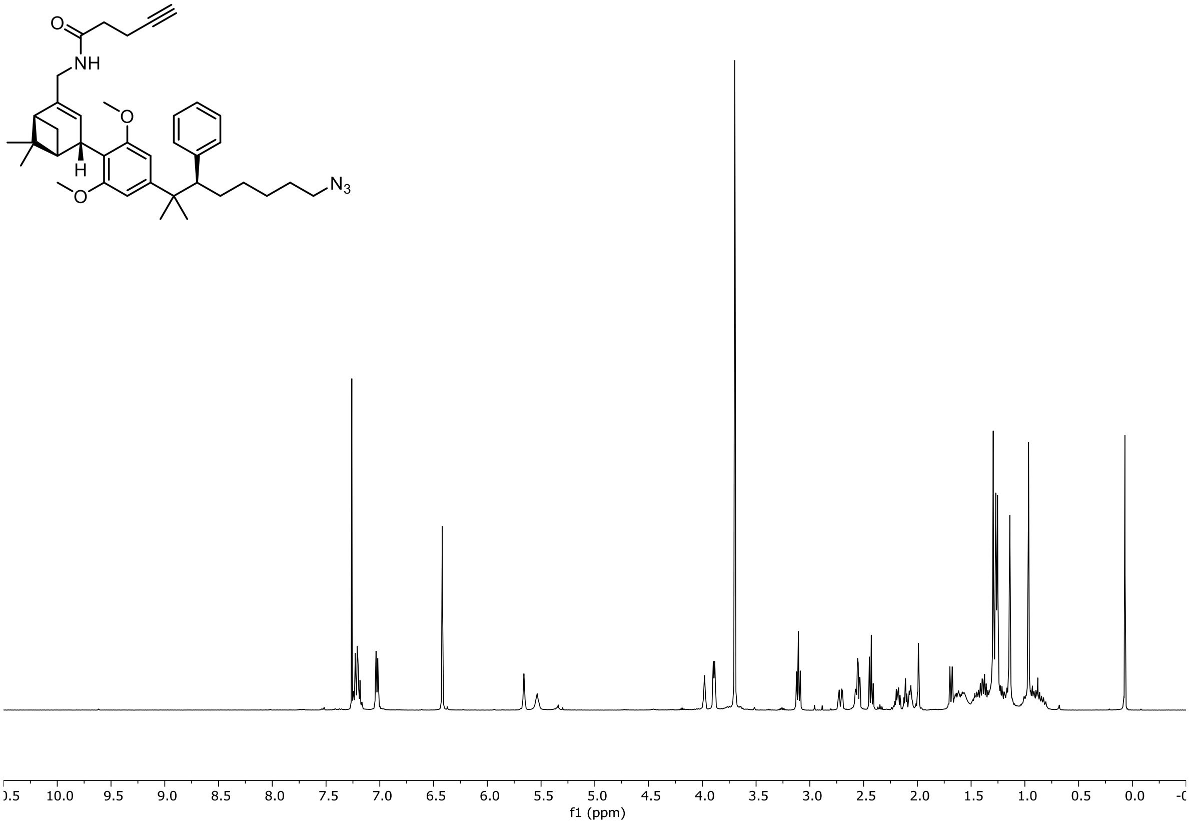

^{13}C NMR (101 MHz, $CDCl_3$) of (*R*)-**3**

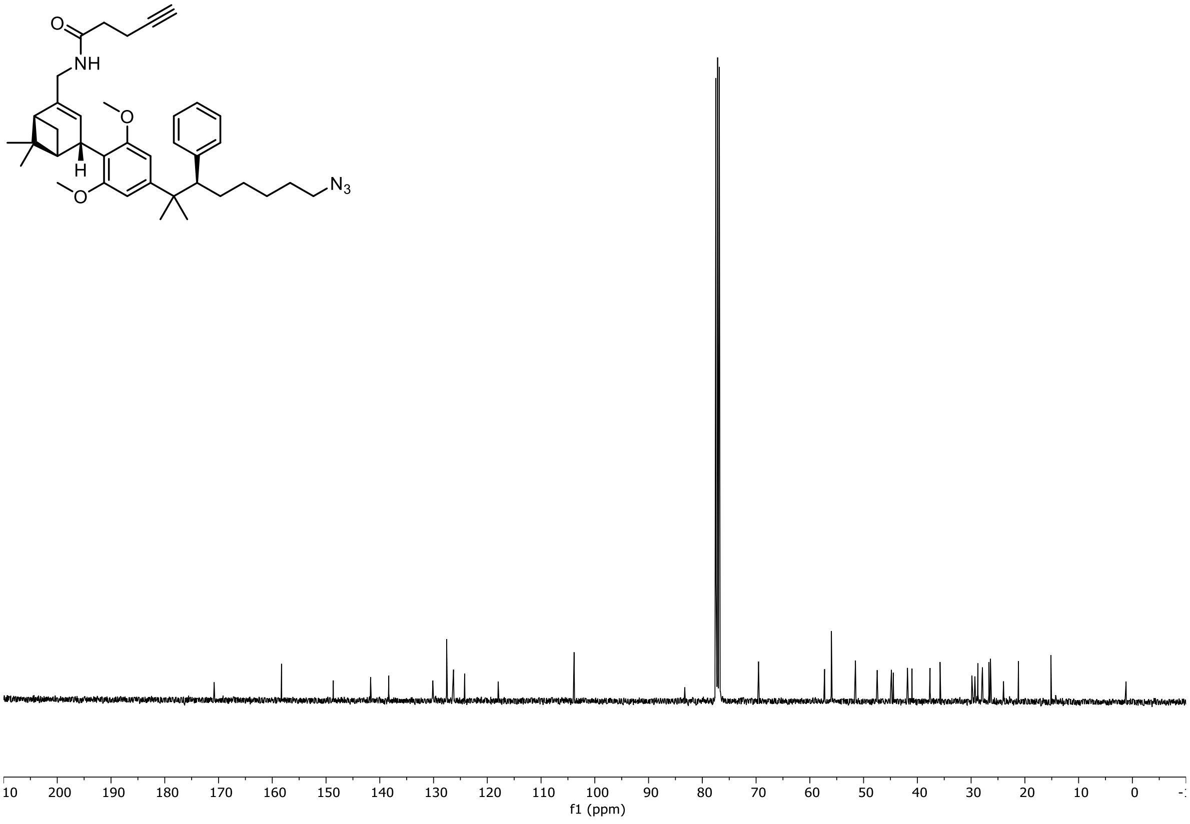

^{1}H NMR (500 MHz, CD_3OD) of (*S*)-**4**

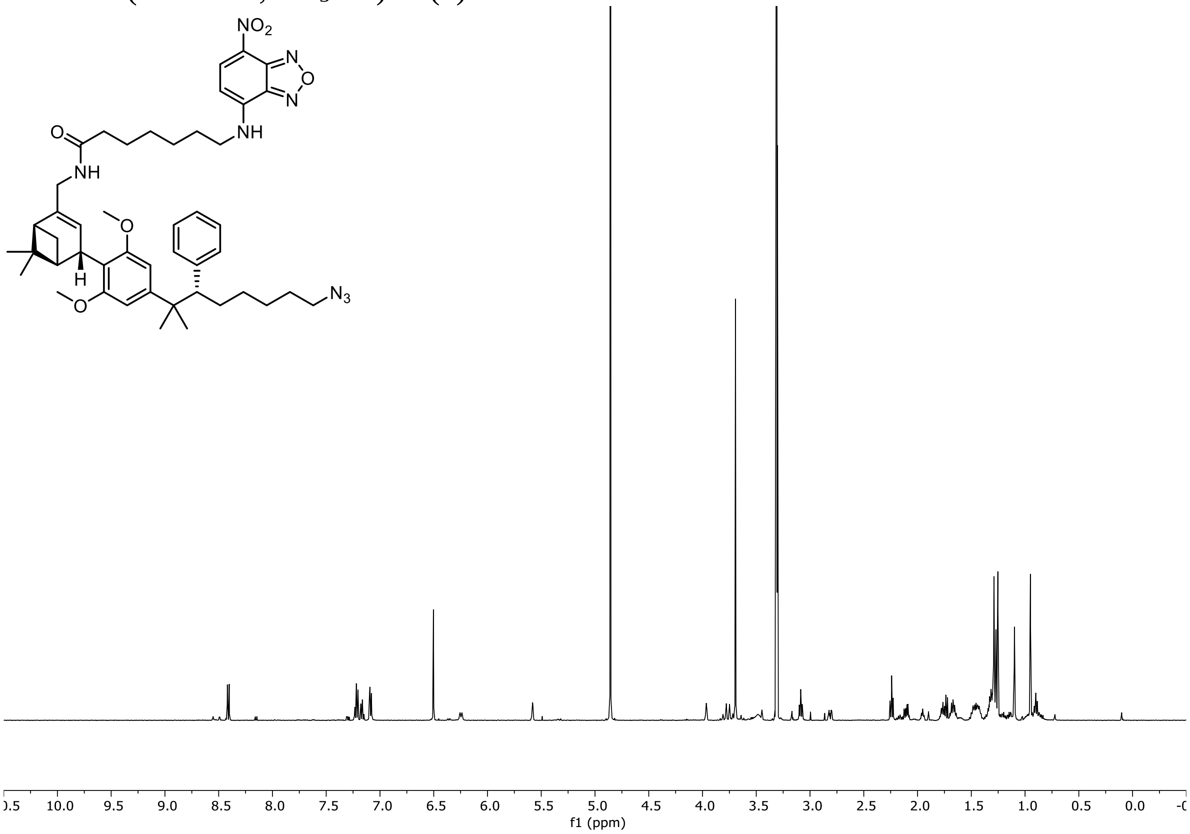

^{13}C NMR (126 MHz, CD_3OD) of (*S*)-**4**

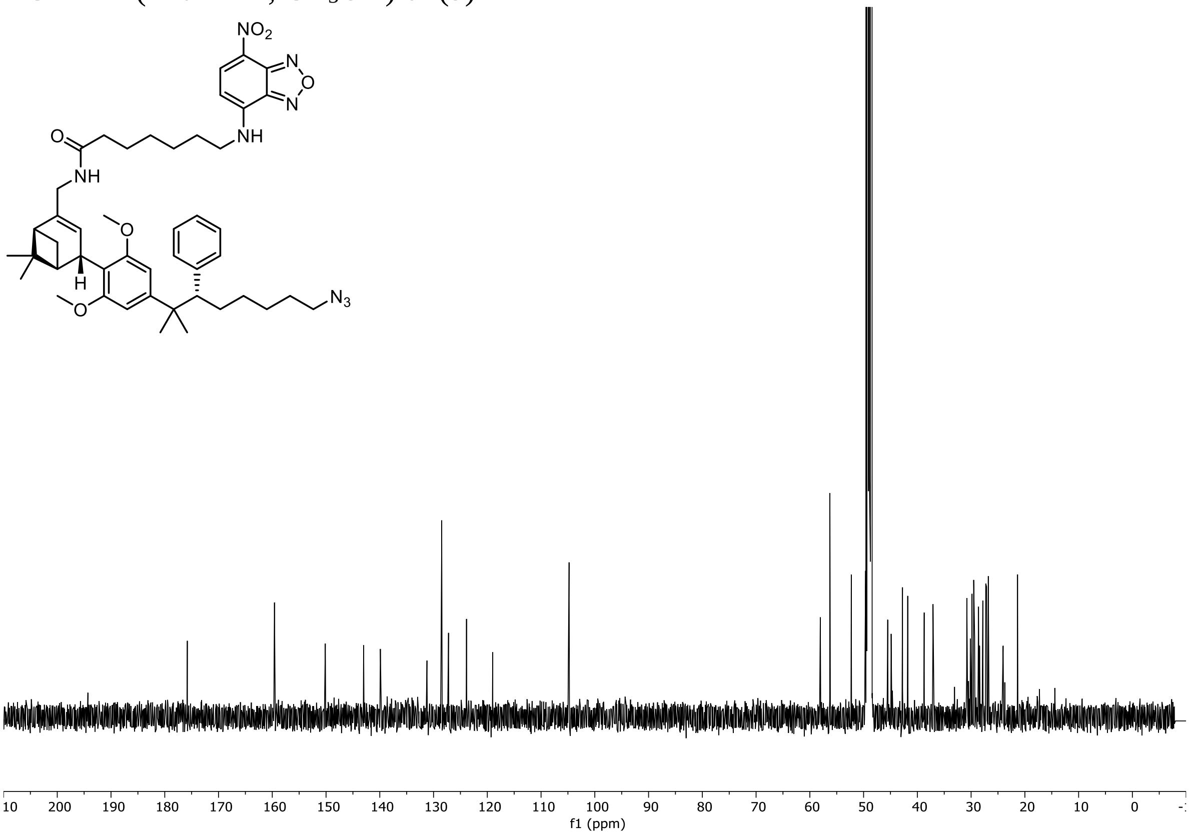

^{1}H NMR (500 MHz, CD_3OD) of (*R*)-**4**

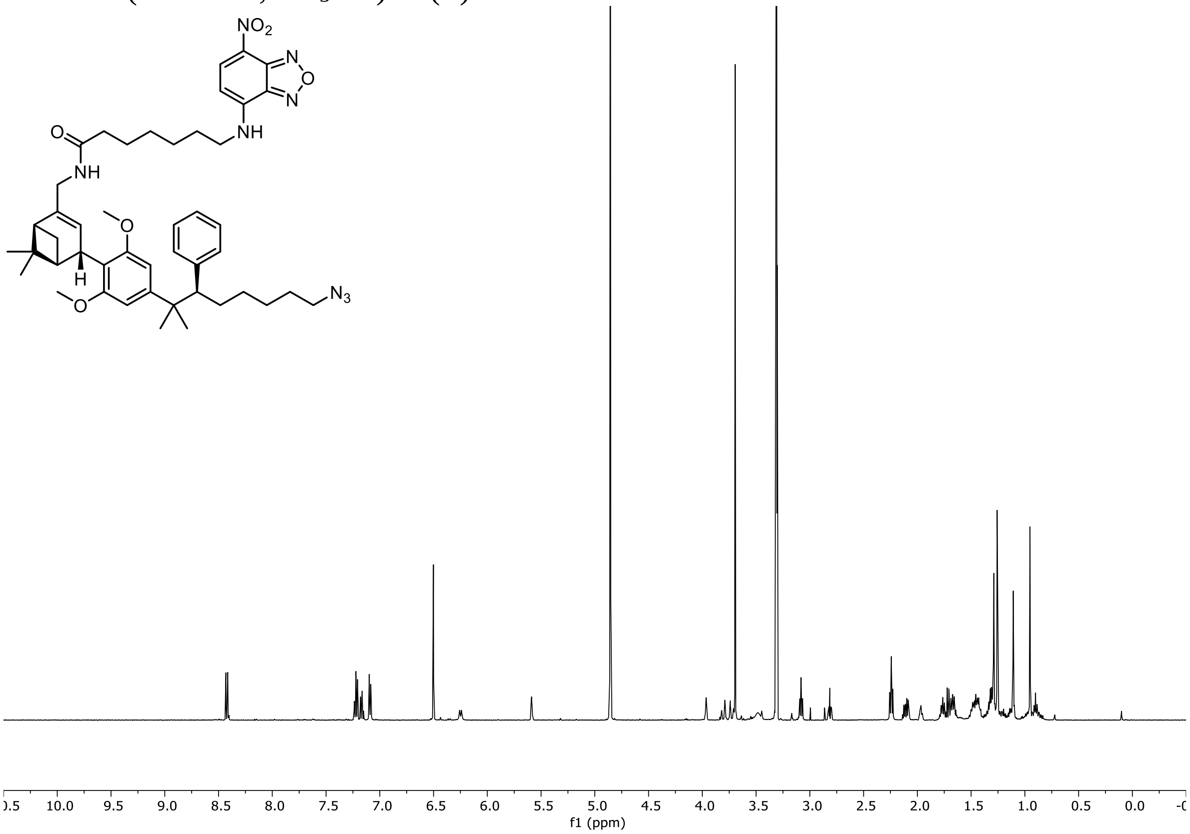

^{13}C NMR (126 MHz, CD_3OD) of (*R*)-**4**

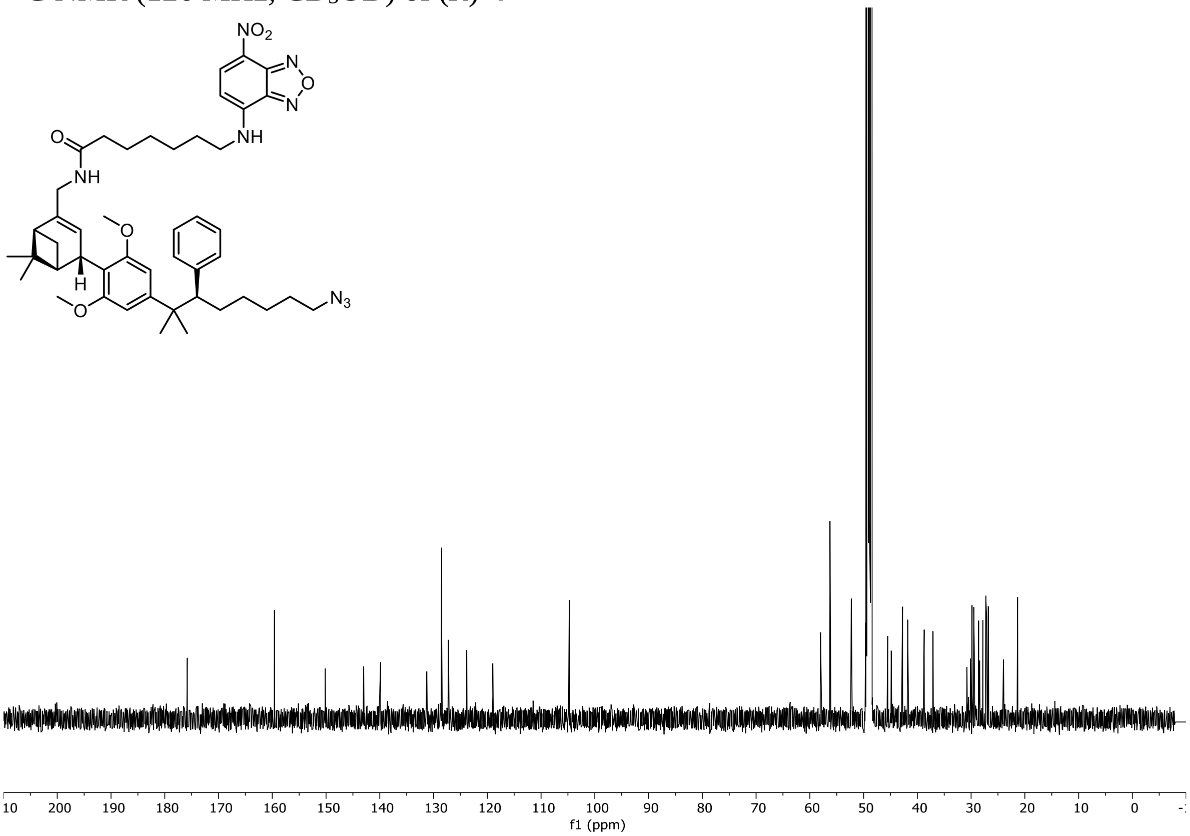

^{1}H NMR (400 MHz, $CDCl_3$) of (*R*)-**SI-4**

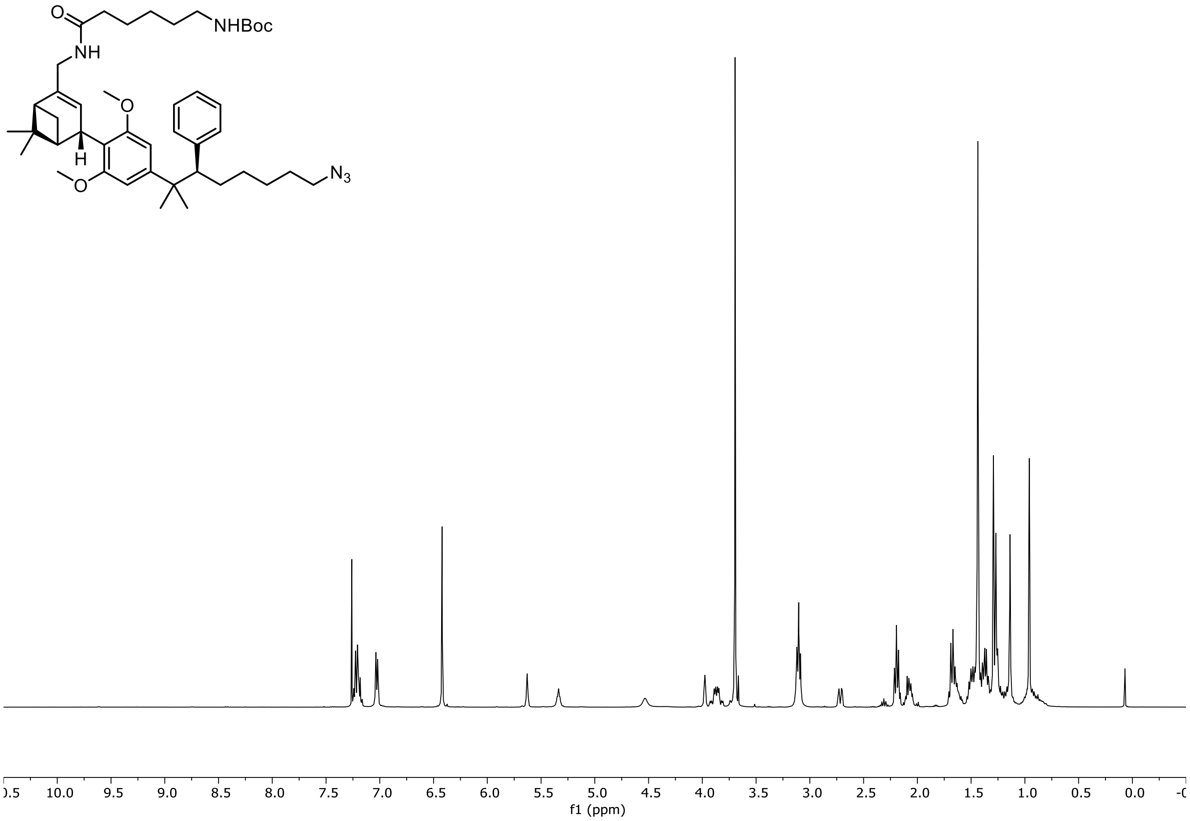

^{13}C NMR (101 MHz, $CDCl_3$) of (*R*)-**SI-4**

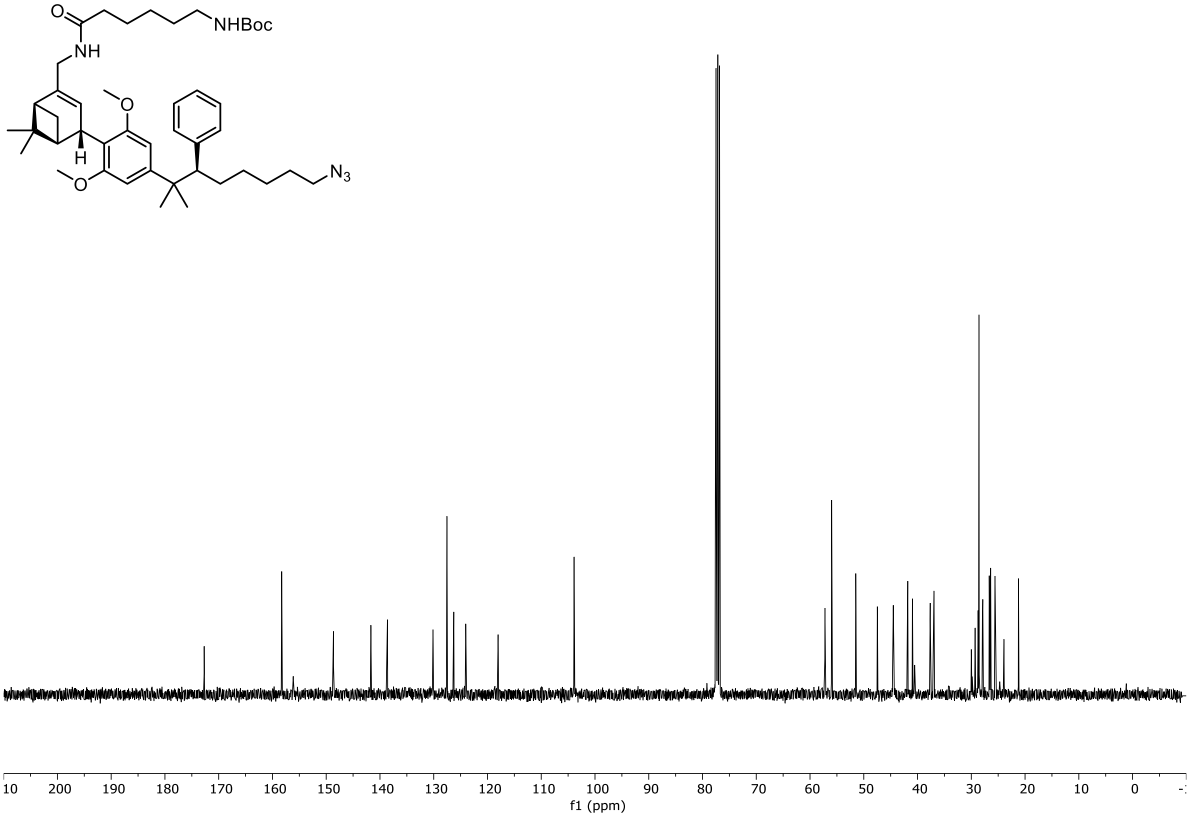

Analytical UPLC chromatogram of (*R*)-**5**

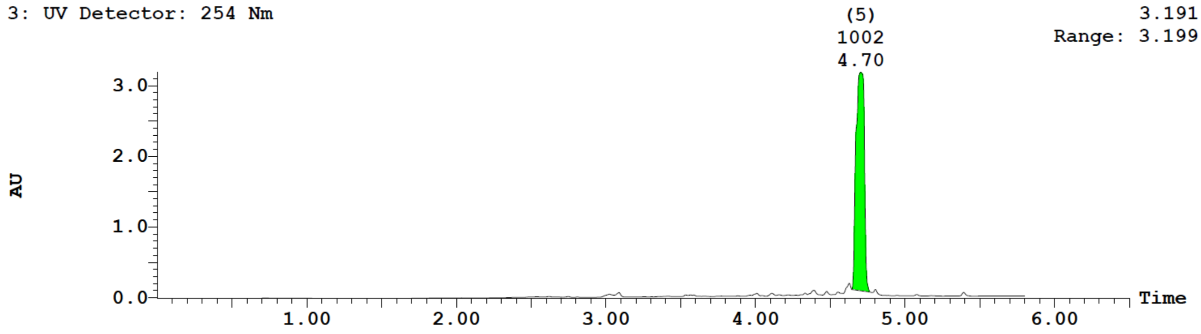

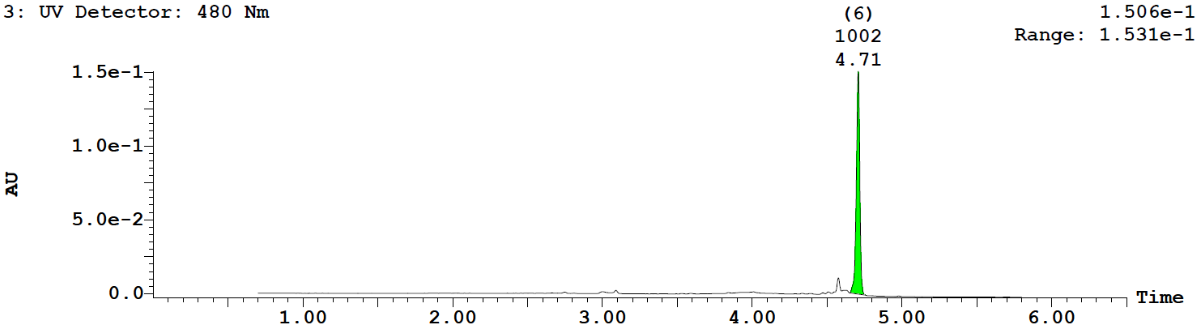

Peak Number	Time	Height	AreaAbs	Area %Total
6	4.71	150952	4051	100.00

Peak number
5

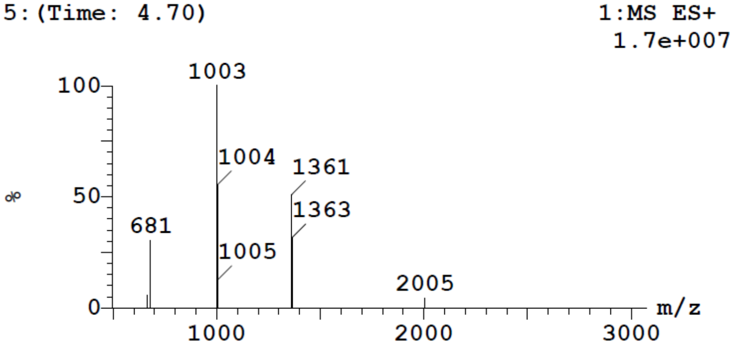

Analytical UPLC chromatogram of (*R*)-**6**

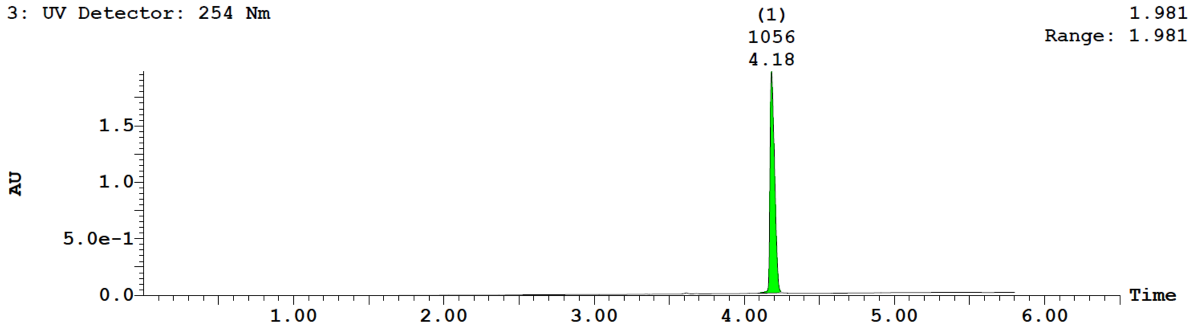

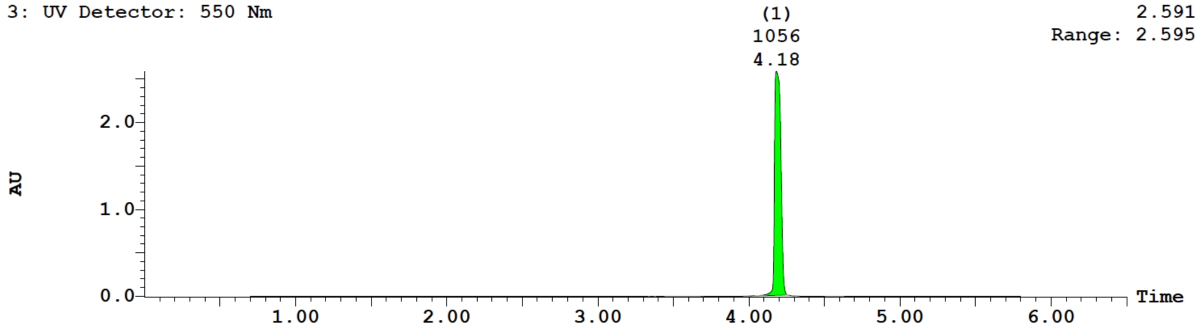

Peak Number	Time	Height	AreaAbs	Area %Total
1	4.18	2580549	125407	100.00

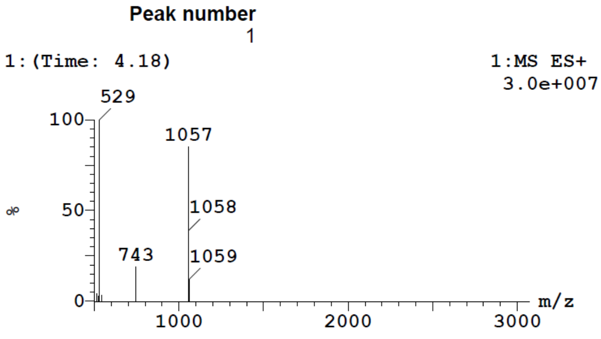

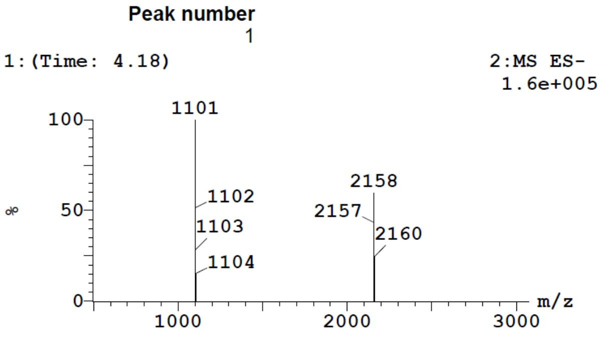

^{1}H NMR (600 MHz, $(CD_3)_2SO$) of (*R*)-7

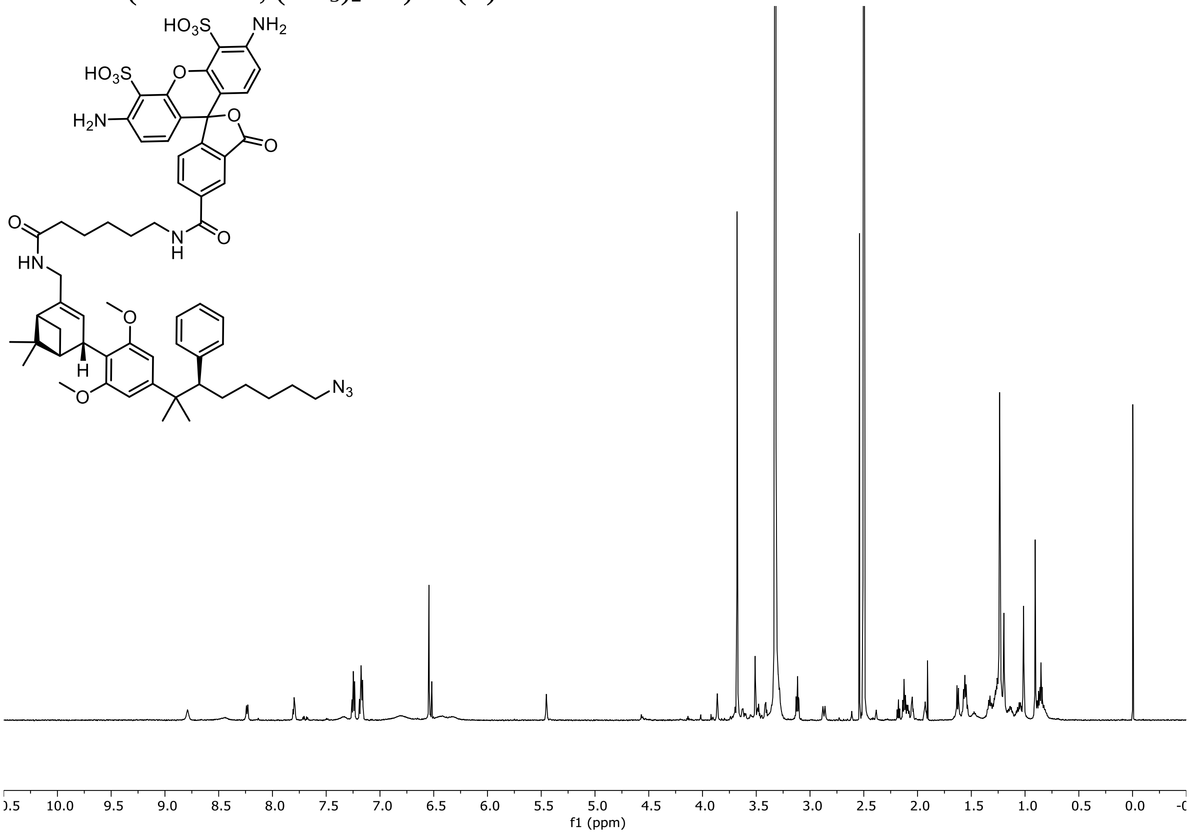

^{13}C NMR (151 MHz, $(CD_3)_2SO$) of (*R*)-7

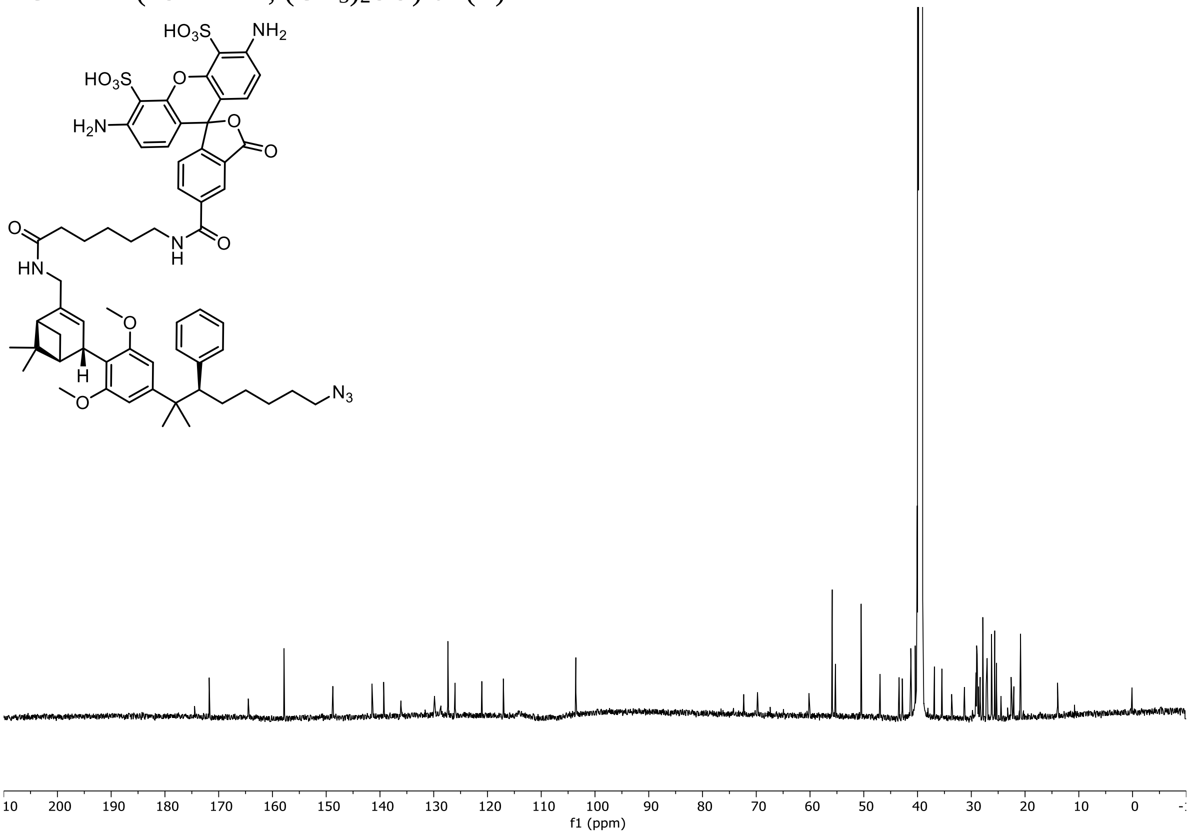

Analytical HPLC chromatogram of (*R*)-**7**

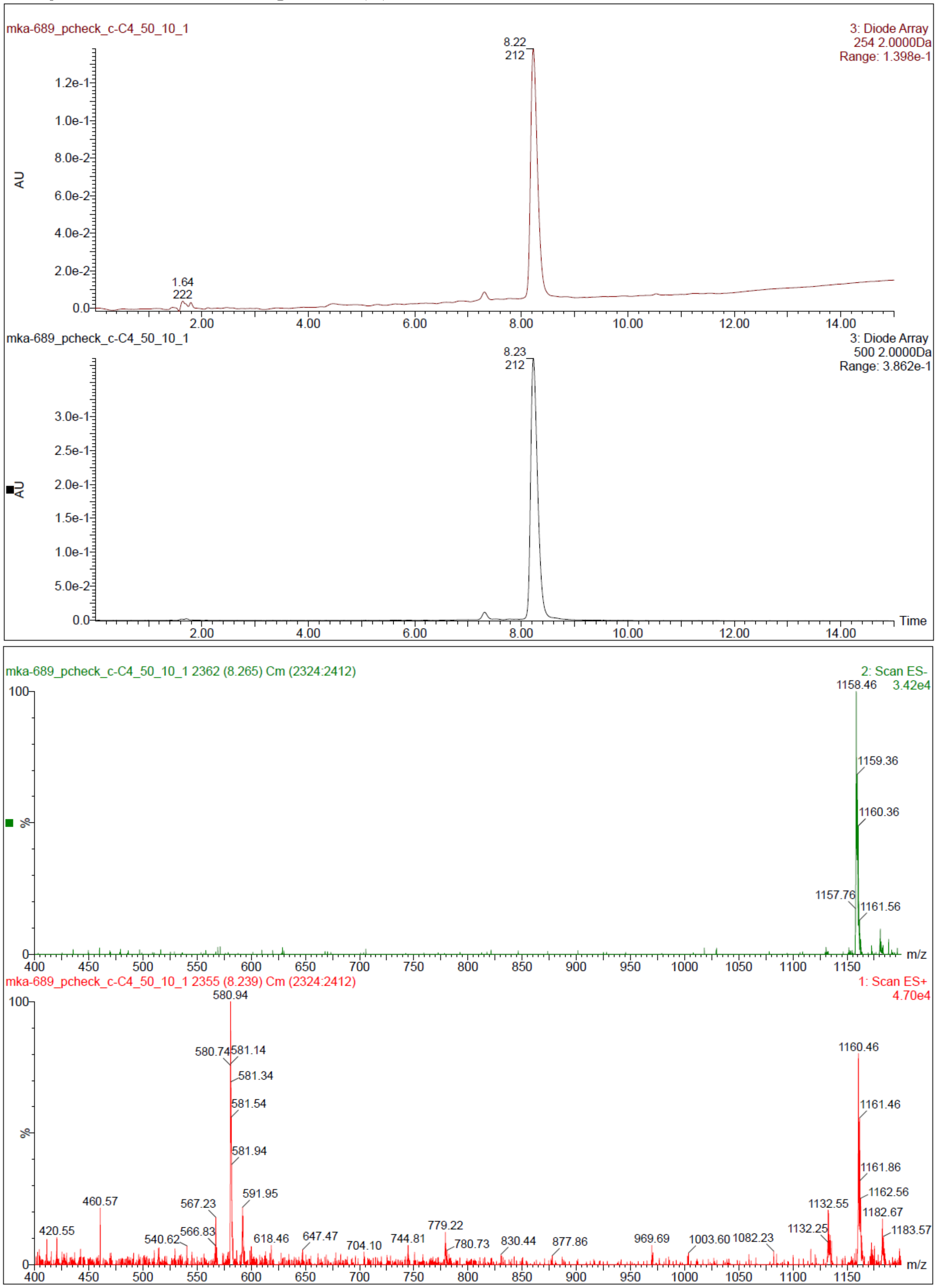

^{1}H NMR (500 MHz, CD_2Cl_2) of (*R*)-**8**

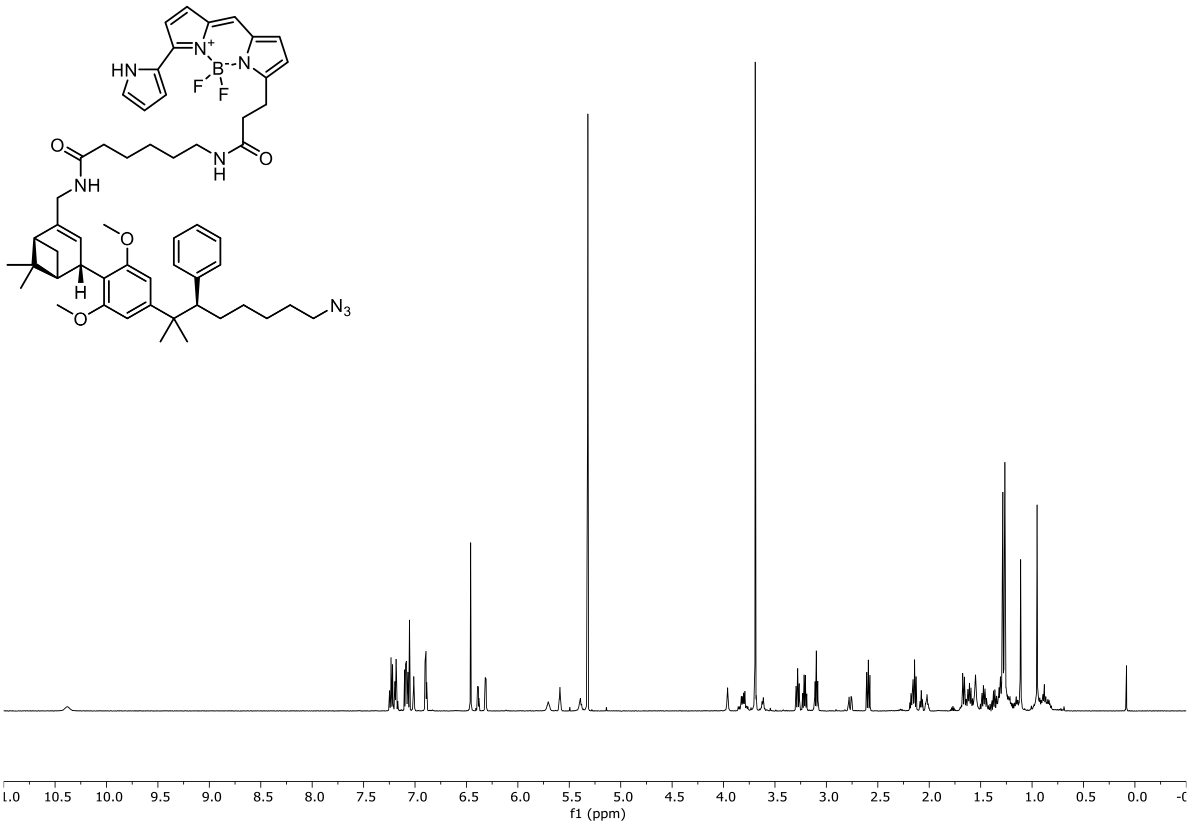

^{13}C NMR (126 MHz, CD_2Cl_2) of (*R*)-**8**

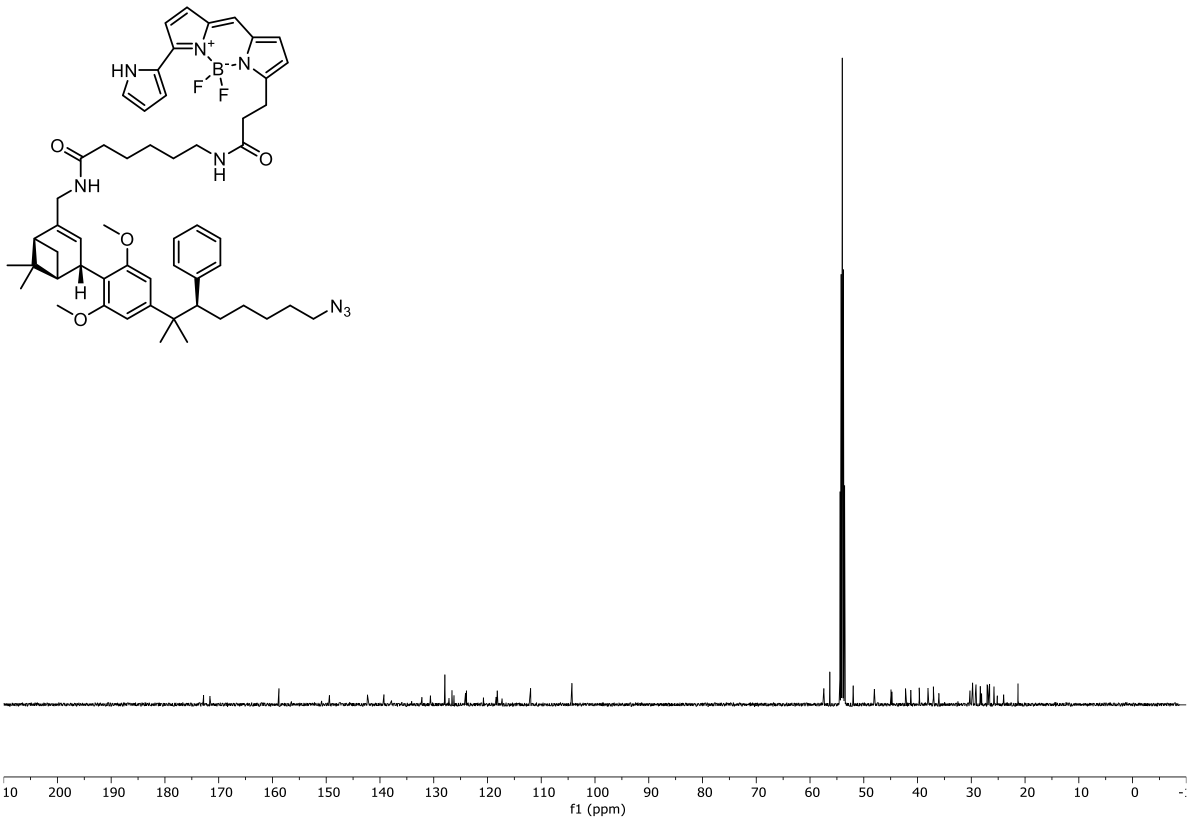

^{19}F NMR (471 MHz, CD_2Cl_2) of (*R*)-**8**

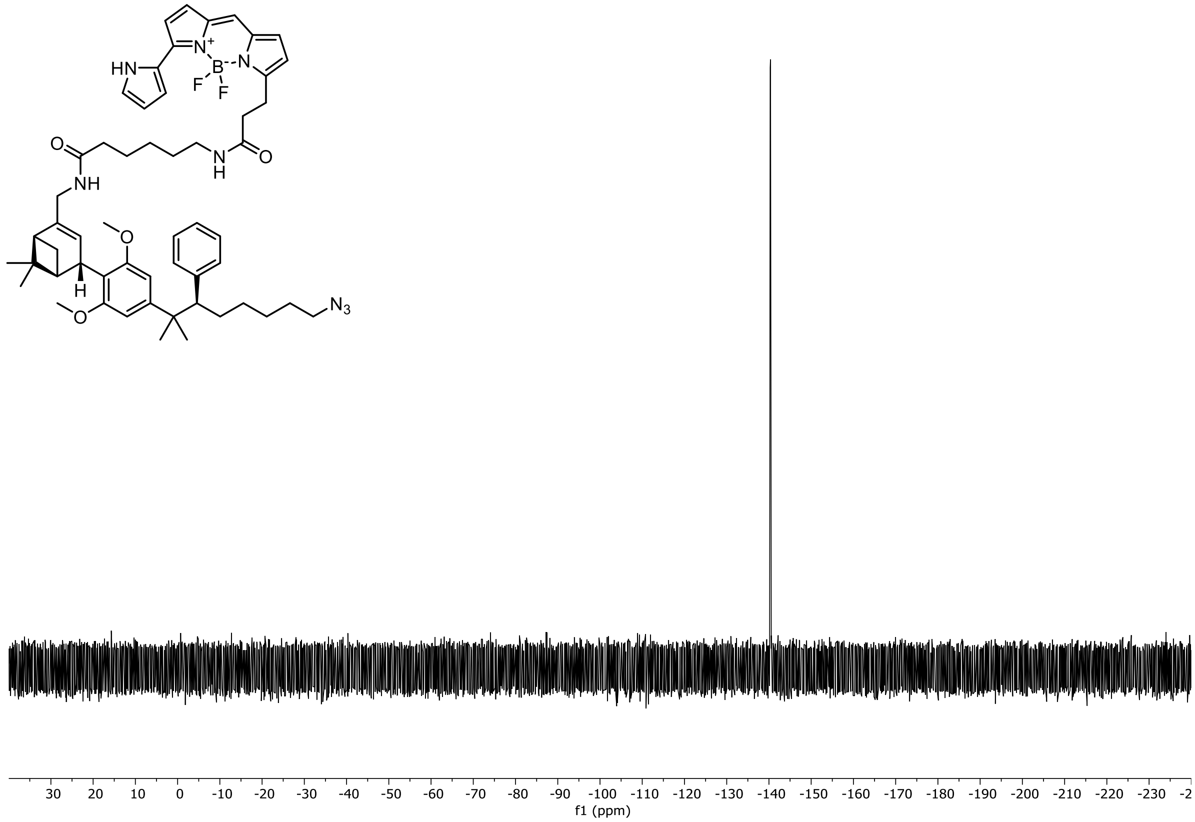

^{11}B NMR (160 MHz, CD_2Cl_2) of (*R*)-**8**

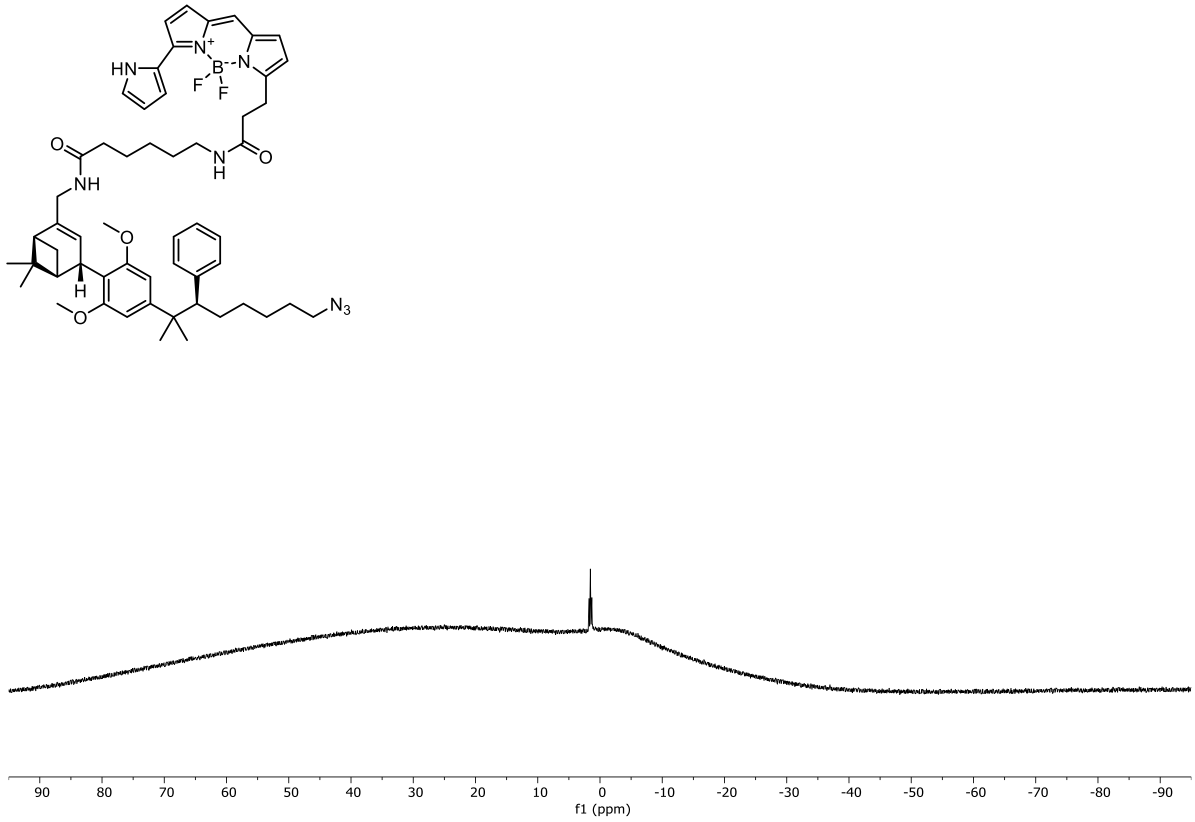

^{1}H NMR (500 MHz, CD_3OD) of (*R*)-**9**

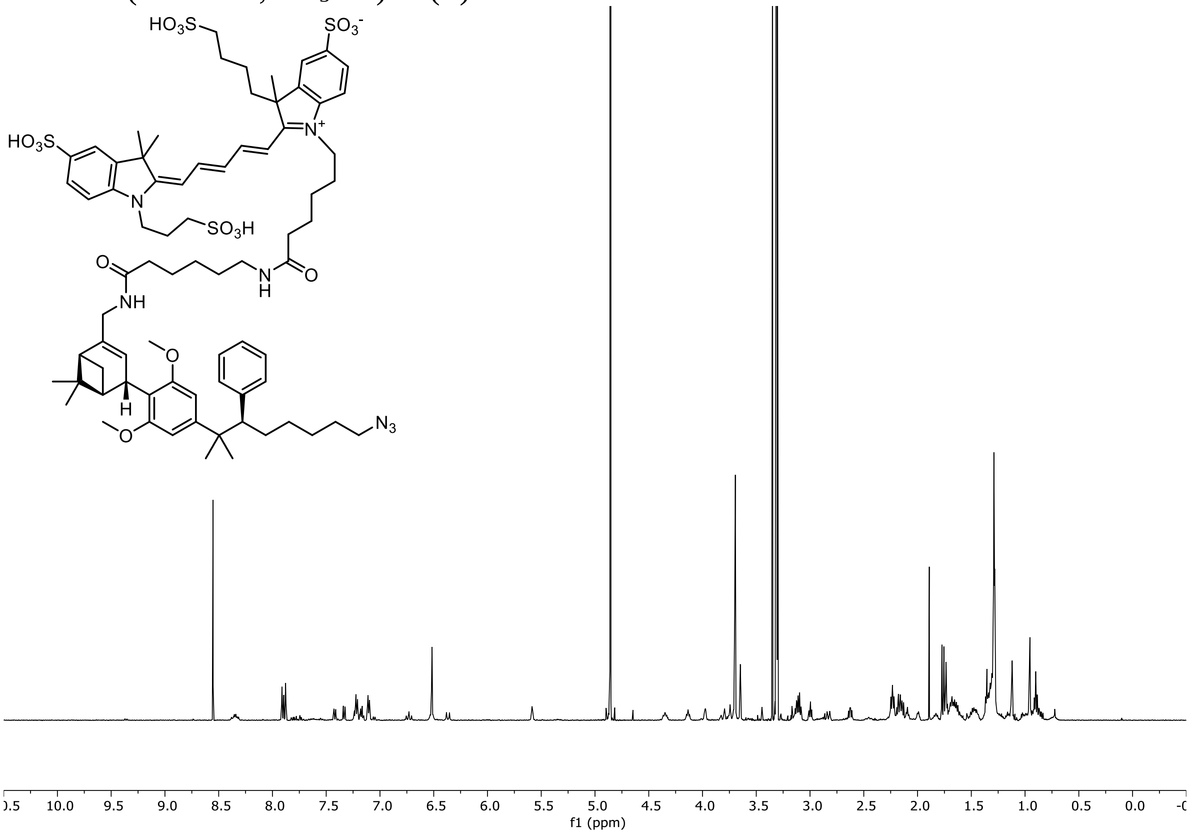

^{13}C NMR (126 MHz, CD_3OD) of (*R*)-**9**

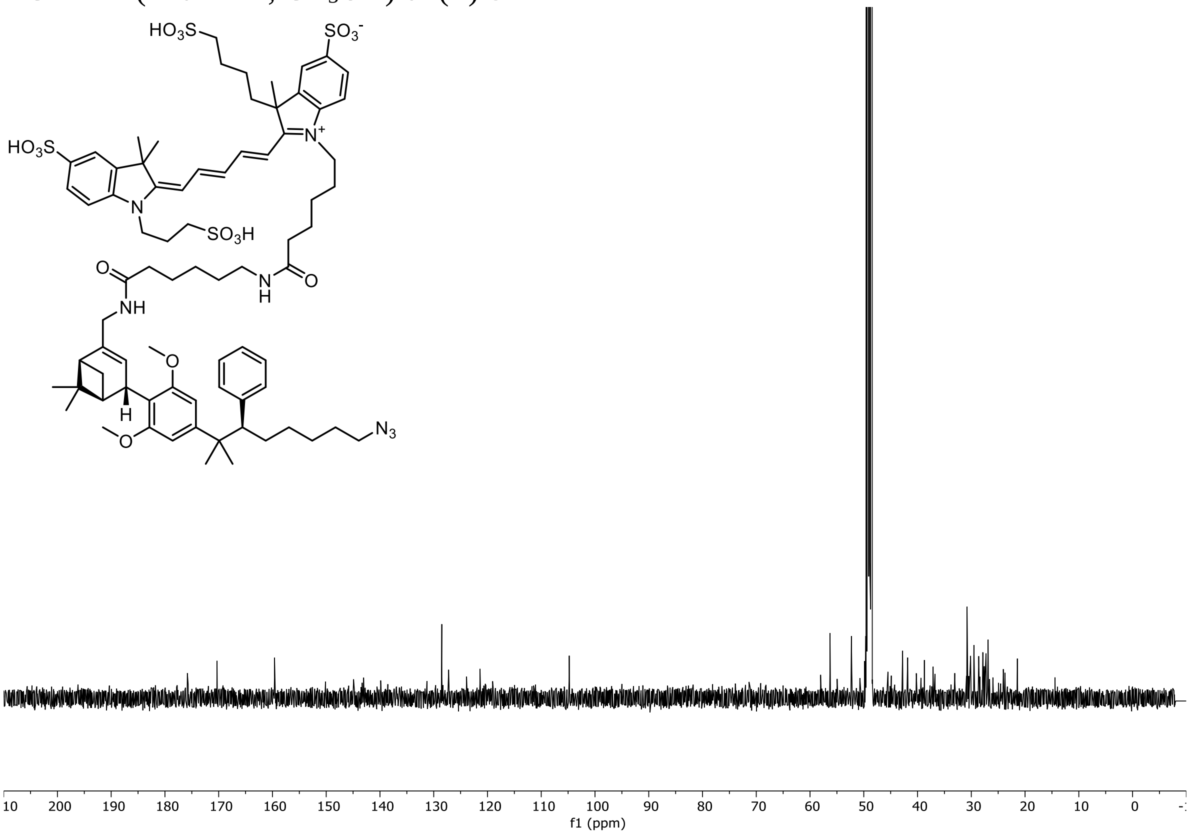

^{1}H NMR (400 MHz, $CDCl_3$) of (*S*)-**SI-6**

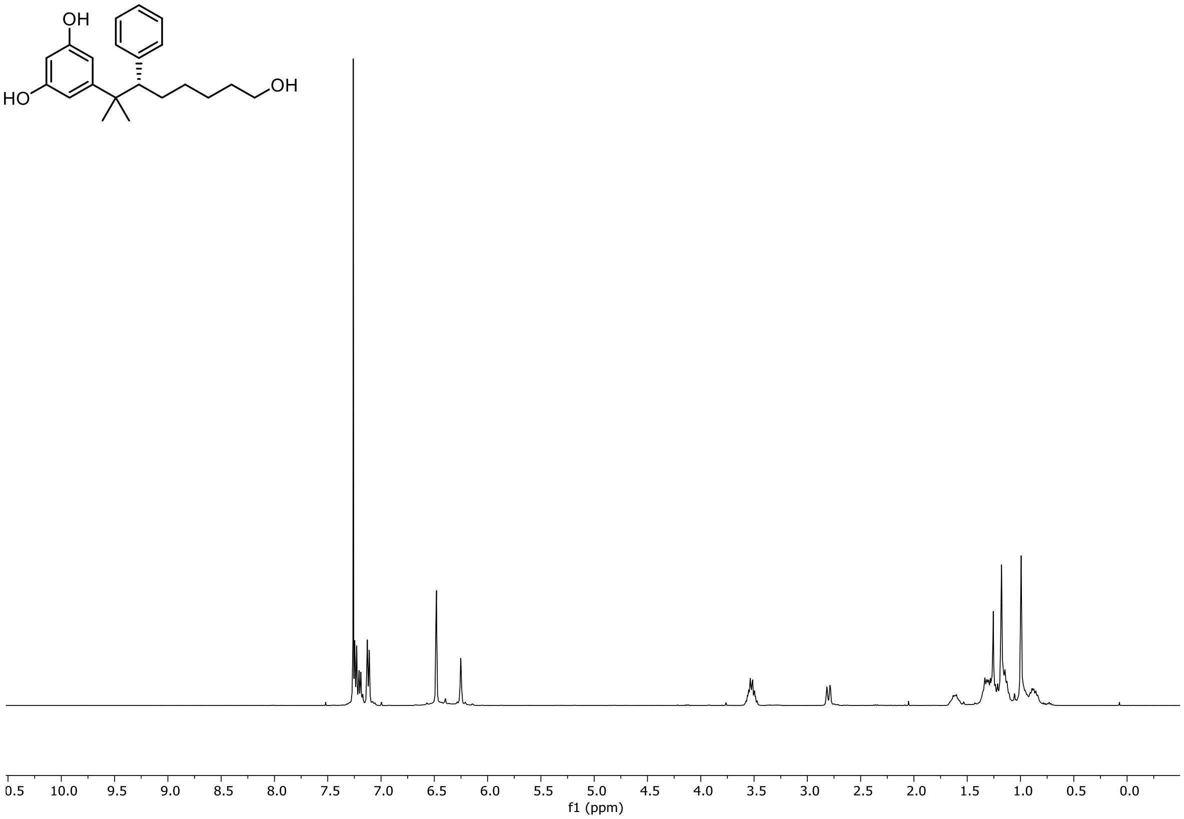

^{13}C NMR (101 MHz, $CDCl_3$) of (*S*)-**SI-6**

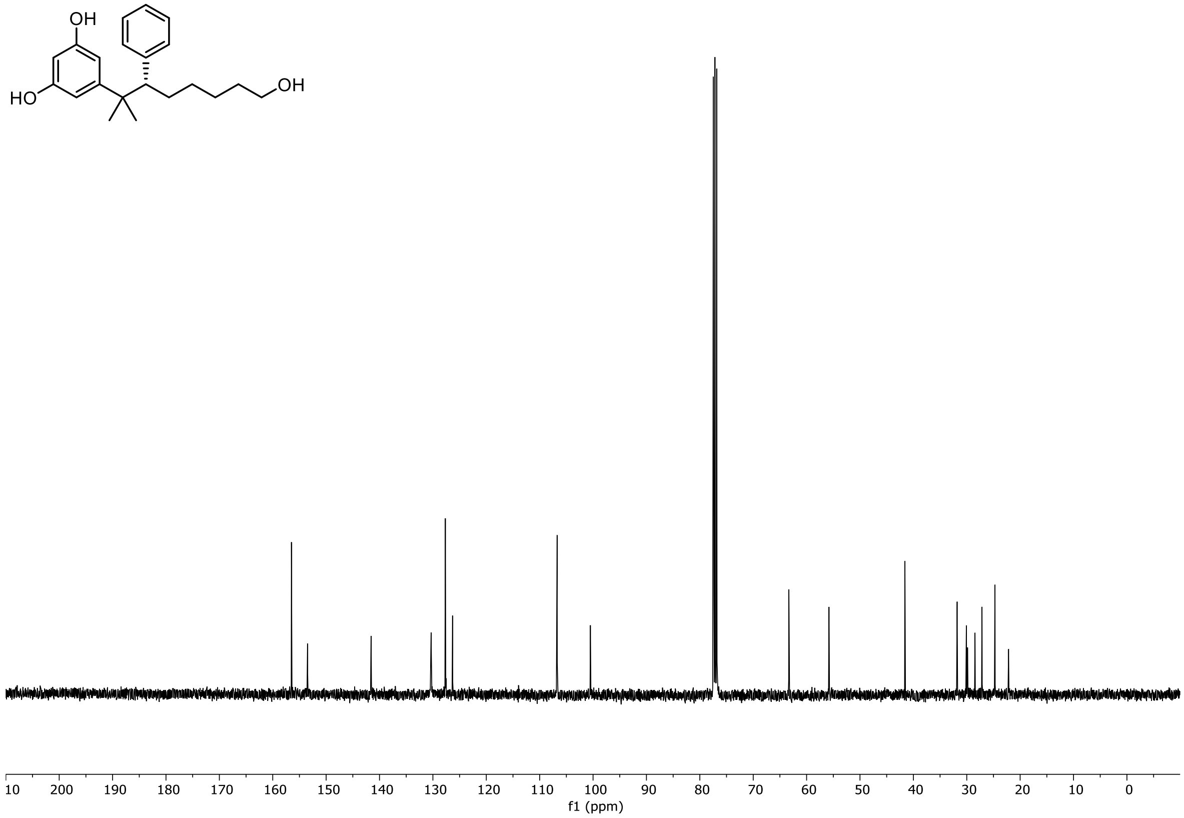

^{1}H NMR (500 MHz, $CDCl_3$) of (*S*)-**17**

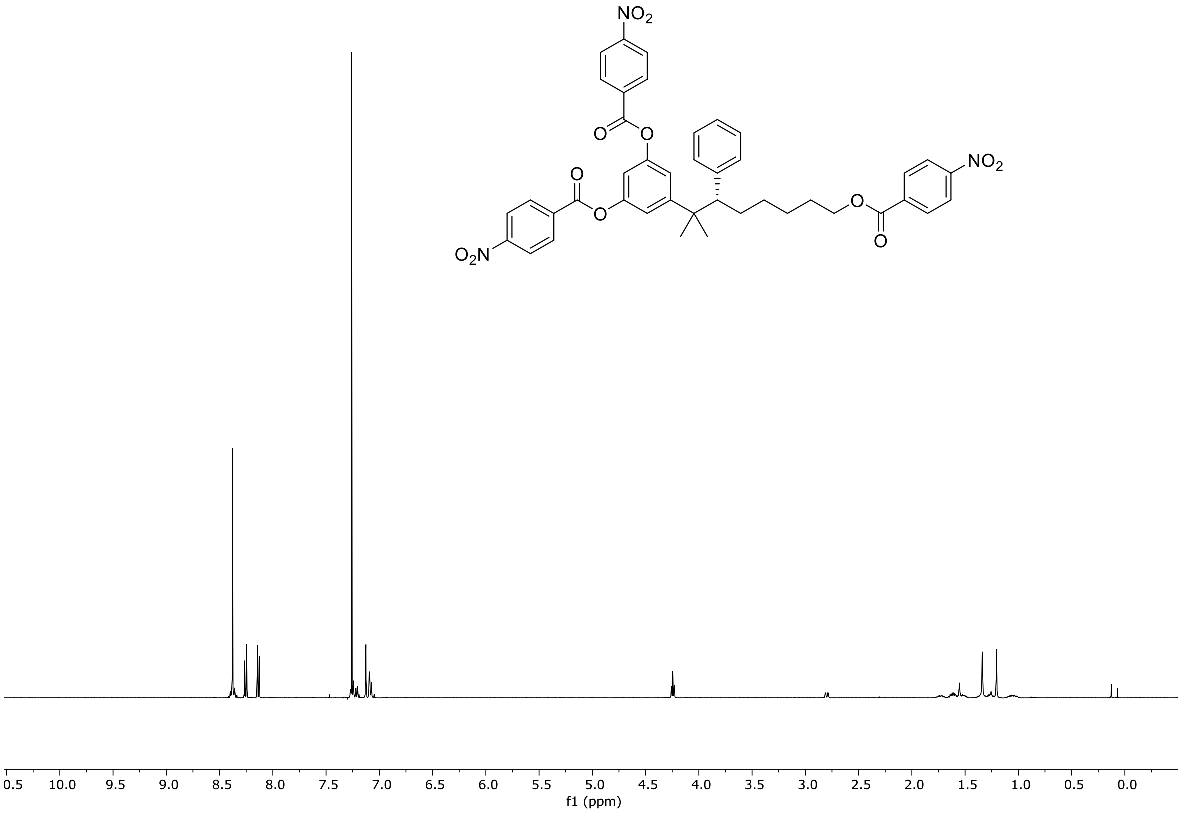

^{13}C NMR (126 MHz, $CDCl_3$) of (*S*)-**17**

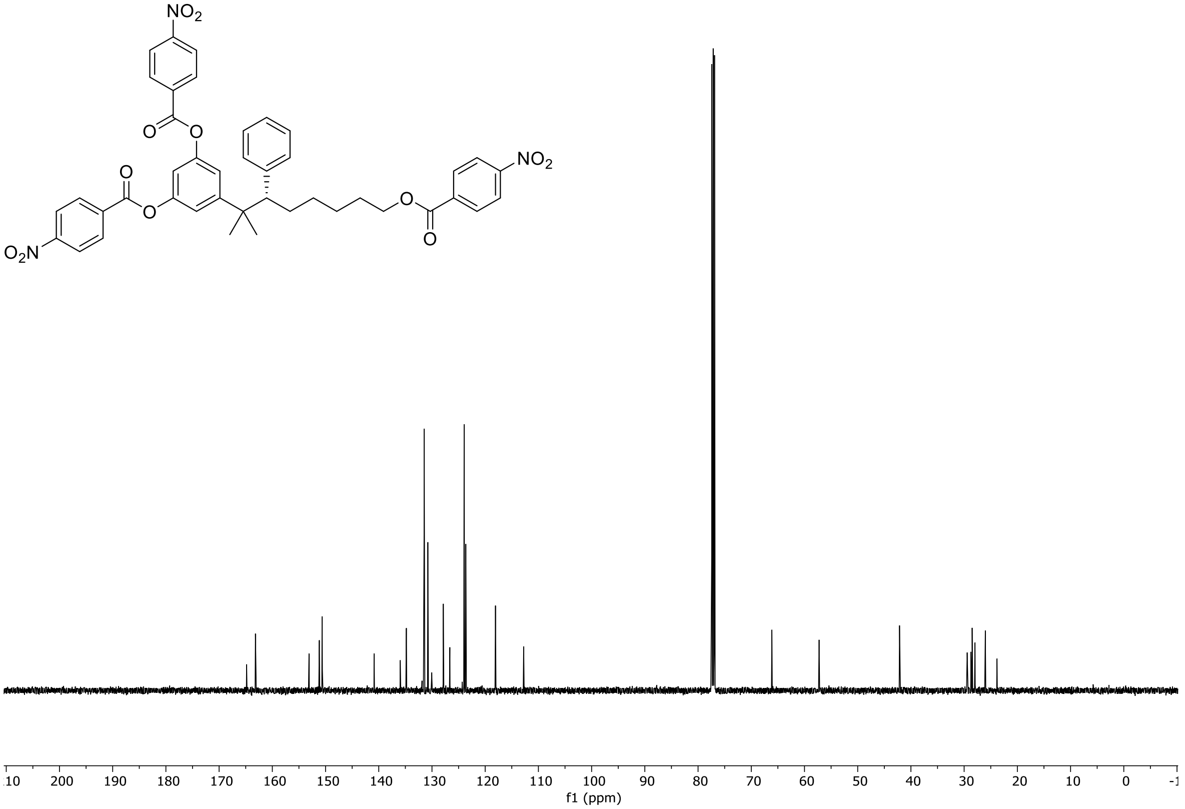

^{1}H NMR (500 MHz, $CDCl_3$) of **ago-3**

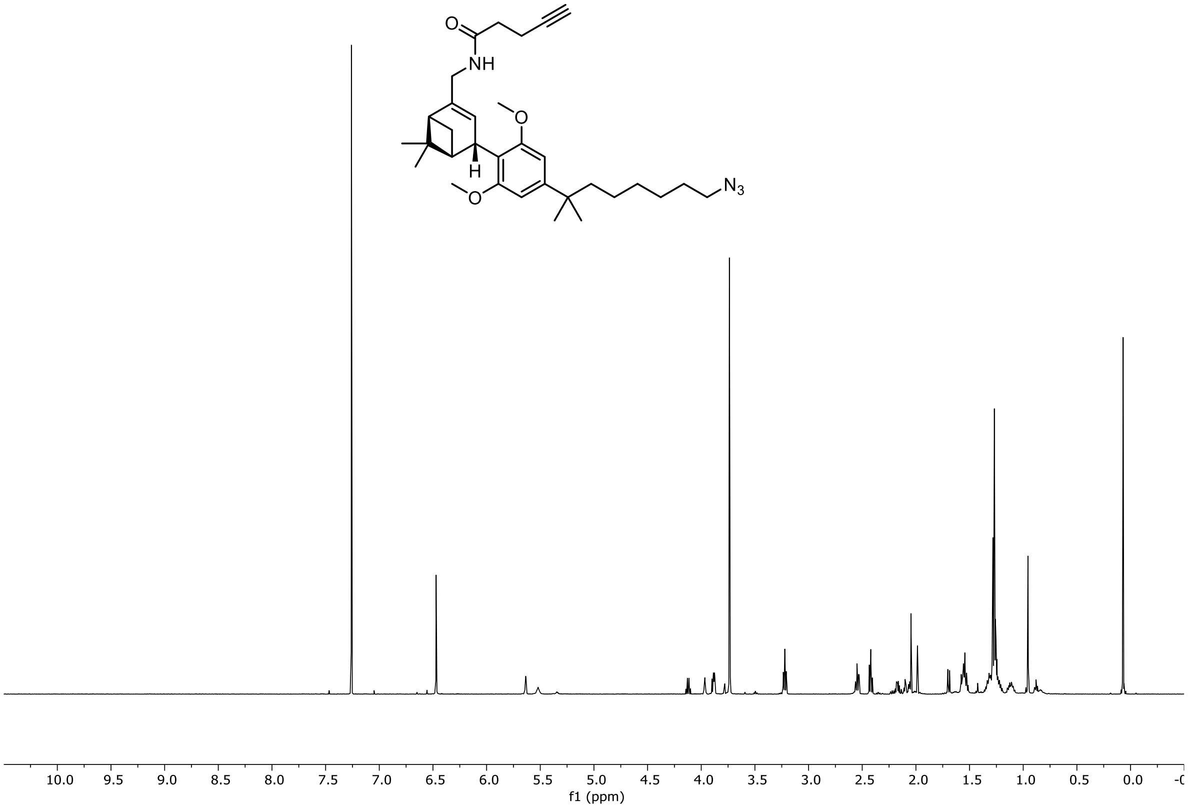

^{13}C NMR (126 MHz, $CDCl_3$) of **ago-3**

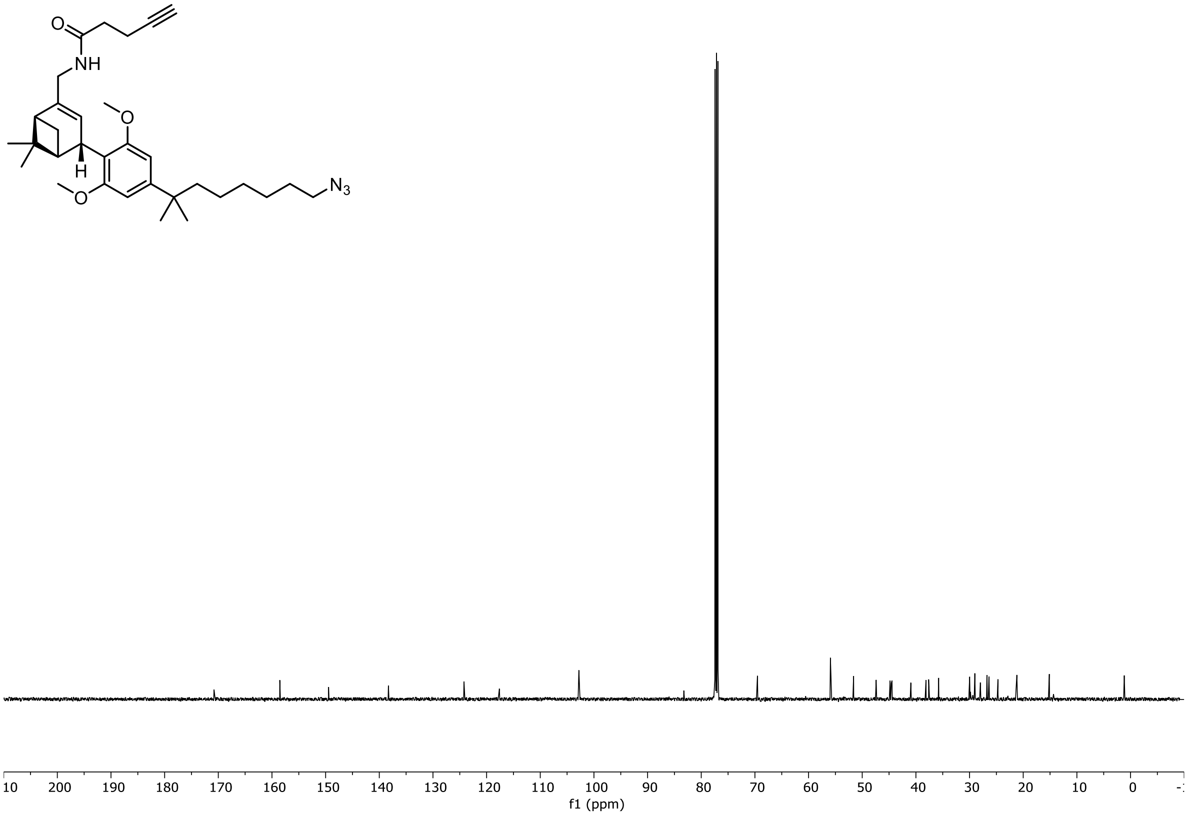

SFC TRACES

SFC trace of racemate (*S*)-**14**/(*R*)-**14**

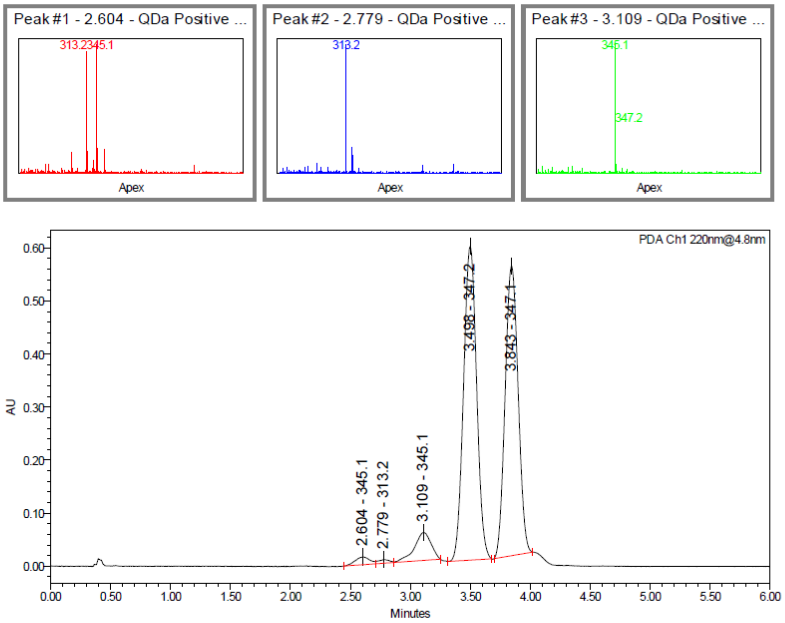

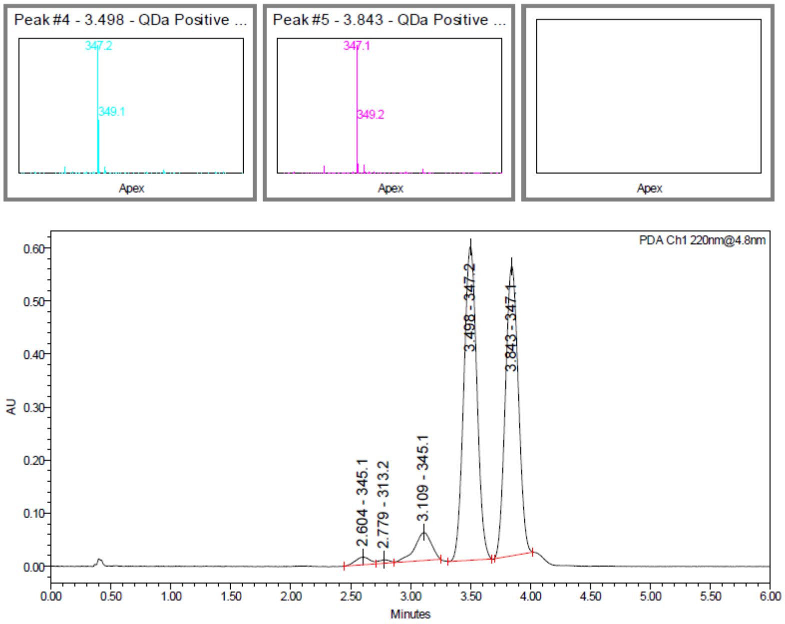

Peak Results

	RT	Area	% Area	Base Peak (m/z)
1	2.604	122241	1	345.1
2	2.779	43727	0	313.2
3	3.109	511059	6	345.1
4	3.498	4480531	48	347.2
5	3.843	4104558	44	347.1

SFC trace of (*S*)-**14**

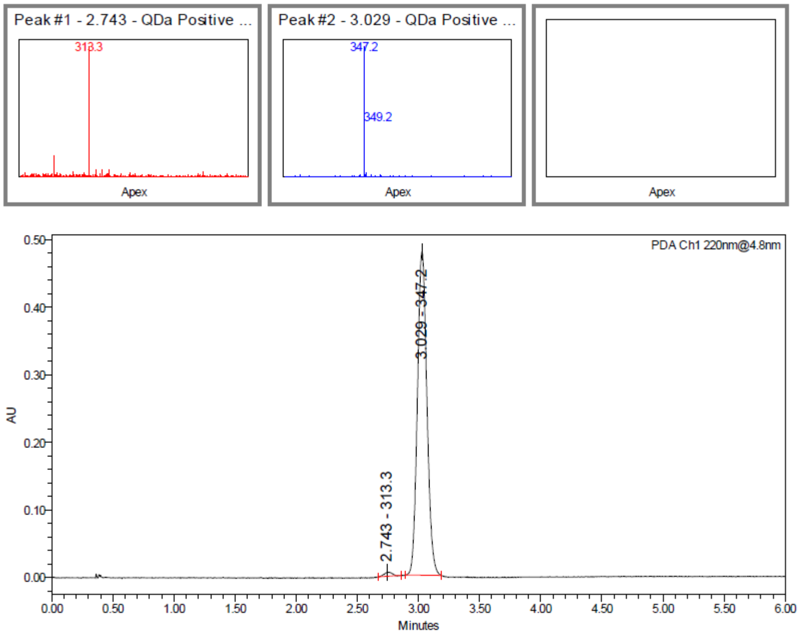

Peak Results

	RT	Area	% Area	Base Peak (m/z)
1	2.743	27606	1	313.3
2	3.029	2583724	99	347.2

SFC trace of (*R*)-**14**

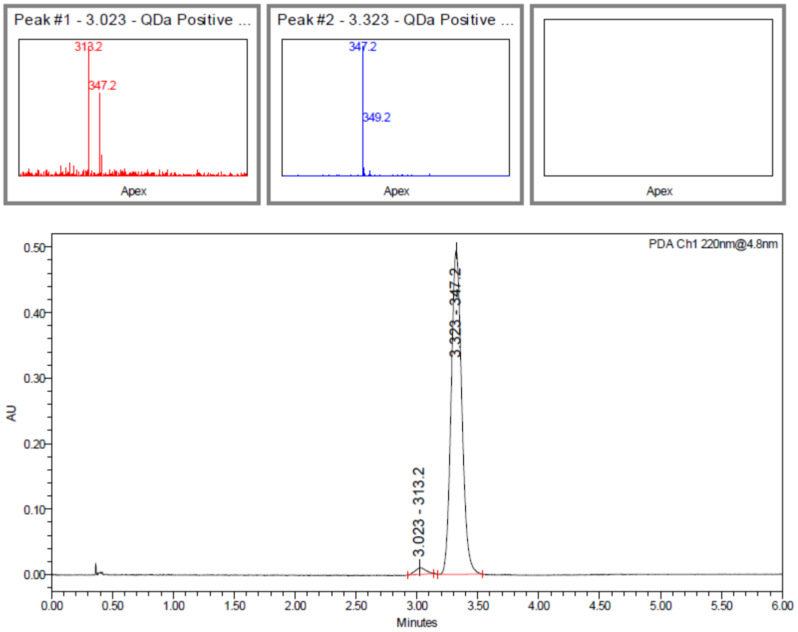

Peak Results

	RT	Area	% Area	Base Peak (m/z)
1	3.023	62092	2	313.2
2	3.323	2992202	98	347.2

CRYSTALLOGRAPHIC DATA

Compound (*S*)-14-[4-(NO_2)C_6H_4CO]$_3$: (*S*)-5-(2-methyl-8-((4-nitrobenzoyl)oxy)-3-phenyloctan-2-yl)-1,3-phenylene bis(4-nitrobenzoate)

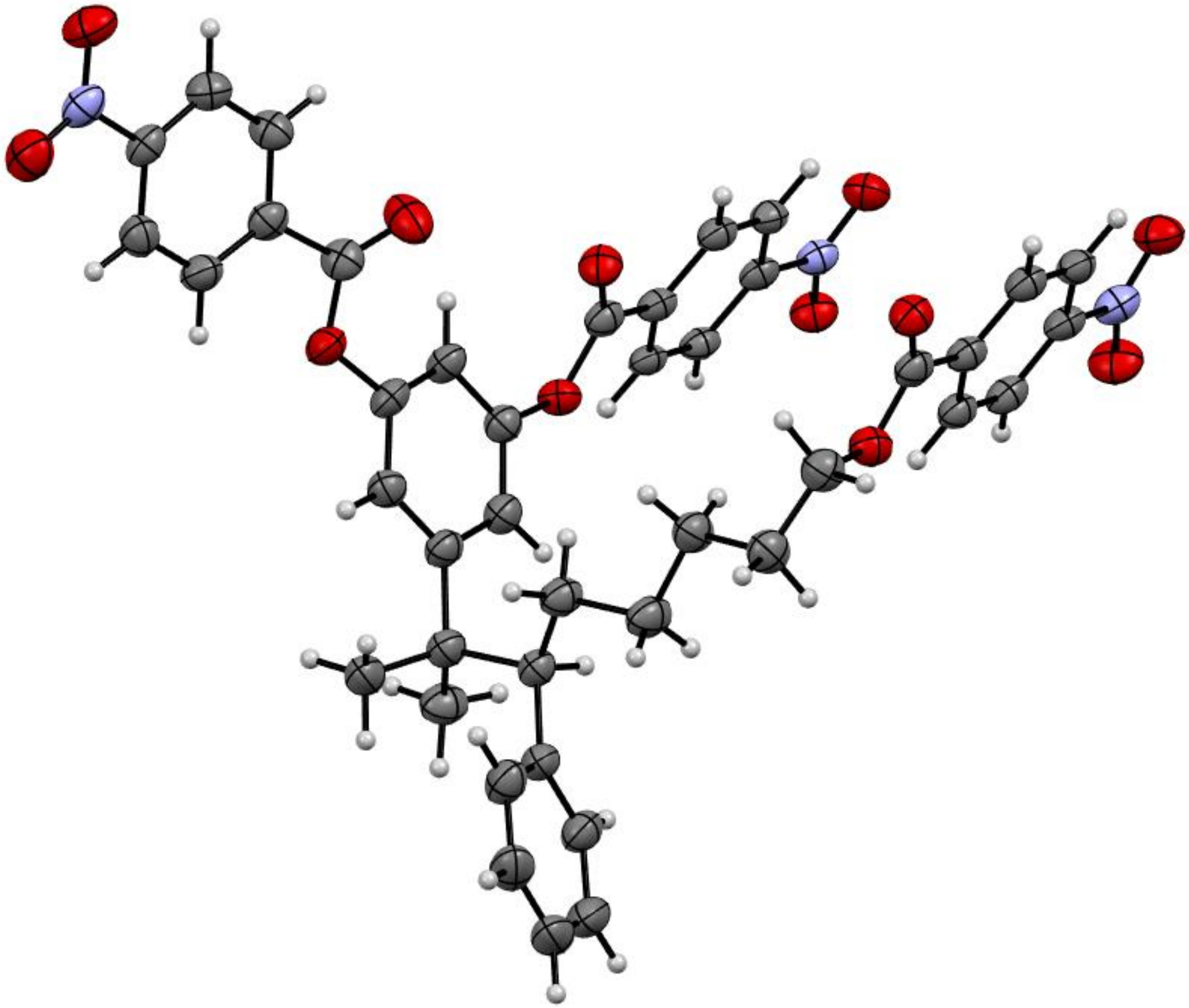

Bond precision:	C–C = 0.0064 A	Wavelength = 1.54184

Cell:	a=12.6822 (2)	b=6.9531 (1)	c=42.7059 (7)
	alpha=90	beta=95.895 (1)	gamma=90

Temperature:	100 K	
	Calculated	Reported
Volume	3745.92 (10)	3745.92 (10)
Space group	P 21	P 1 21 1
Hall group	P 2yb	P 2yb
Moiety formula	C42 H37 N3 O12	C42 H37 N3 O12
Sum formula	C42 H37 N3 O12	C42 H37 N3 O12
Mr	775.75	775.74
Dx,g cm–3	1.375	1.376
Z	4	4
Mu (mm–1)	0.851	0.851
F000	1624.0	1624.0
F000’	1629.54	
h,k,lmax	15,8,53	15,8,53
Nref	15418[8371]	14274
Tmin,Tmax	0.974,0.989	0.779,1.000
Tmin’	0.787	

Correction method= # Reported T Limits: Tmin=0.779 Tmax=1.000

AbsCorr = GAUSSIAN

Data completeness= 1.71/0.93	Theta(max)= 74.953
R(reflections)= 0.0543(10459)	wR2(reflections)= 0.1483(14274)
S = 1.028	Npar= 1031

CCDC Deposition number: 2310248
